# Development of a novel and rapid phenotype-based screening method to assess rice seedling growth

**DOI:** 10.1186/s13007-020-00682-6

**Published:** 2020-10-15

**Authors:** Lena Vlaminck, Chananchida Sang-Aram, Deborah Botterman, Christine Jewel C. Uy, Mary Kay Harper, Dirk Inzé, Godelieve Gheysen, Stephen Depuydt

**Affiliations:** 1 Present Address: Department of Plant Biotechnology and Bioinformatics, Ghent University, 9052 Ghent, Belgium; 2 Center for Plant Systems Biology, VIB, 9052 Ghent, Belgium; 3 Laboratory of Plant Growth Analysis, Ghent University Global Campus, Incheon, 21985 South Korea; 4 Department of Medical Chemistry, University of Utah, Salt Lake City, UT 84112 USA; 5 Department of Biotechnology, Ghent University, 9000 Ghent, Belgium

**Keywords:** *Oryza sativa*, Phenotype-based screening, Seedling growth, Automated computer analysis, Marine natural products, Plength, RIVA

## Abstract

**Background:**

Rice (*Oryza sativa*) is one of the most important model crops in plant research. Despite its considerable advantages, (phenotypic) bioassays for rice are not as well developed as for *Arabidopsis thaliana*. Here, we present a phenotype-based screening method to study shoot-related parameters of rice seedlings via an automated computer analysis.

**Results:**

The phenotype-based screening method was validated by testing several compounds in pharmacological experiments that interfered with hormone homeostasis, confirming that the assay was consistent with regard to the anticipated plant growth regulation and revealing the robustness of the set-up in terms of reproducibility. Moreover, abiotic stress tests using NaCl and DCMU, an electron transport blocker during the light dependent reactions of photosynthesis, confirmed the validity of the new method for a wide range of applications. Next, this method was used to screen the impact of semi-purified fractions of marine invertebrates on the initial stages of rice seedling growth. Certain fractions clearly stimulated growth, whereas others inhibited it, especially in the root, illustrating the possible applications of this novel, robust, and fast phenotype-based screening method for rice.

**Conclusions:**

The validated phenotype-based and cost-efficient screening method allows a quick and proper analysis of shoot growth and requires only small volumes of compounds and media. As a result, this method could potentially be used for a whole range of applications, ranging from discovery of novel biostimulants, plant growth regulators, and plant growth-promoting bacteria to analysis of CRISPR knockouts, molecular plant breeding, genome-wide association, and phytotoxicity studies. The assay system described here can contribute to a better understanding of plant development in general.

## Background

Rice (*Oryza sativa*) is one of the most important crops worldwide. It is also a main model species for plant research. It is an excellent model system for monocotyledonous crops, because it has many functionally conserved genes and shows synteny with other Poaceae, such as wheat (*Triticum* sp.), maize (*Zea mays*), barley (*Hordeum vulgare*), and sorghum (*Sorghum bicolor*) [1, 2]. Rice has a relatively short life cycle compared to other cereals and can be maintained via self-fertilization with hardly any cross-contamination risk [3]. It is diploid and possesses a high genetic variability in physiologically and agriculturally relevant features [4]. Moreover, efficient genetic transformation techniques [5] and the availability of the genome sequence [6] and of a large set of genetic, molecular, and genomic resources made rice an major model organism in plant biotechnological research [7]. Furthermore, rice will be of great importance in ensuring global food security, one of the main end goals of applied research of plant biology and biotechnology.

As modern agriculture must increase crop productivity to meet the demands of the growing population, besides the challenges of climate change and reduced availability of arable land [8], technologies from different disciplines will need to be integrated to meet the projected high demand of food by 2050 [9]. Phenotyping is very important in the breeding pipeline, because the identification of genes underlying agronomic traits is limited by the ability to phenotype them [10]. Although marker-assisted selection (MAS) and genomic selection (GS) mostly depend on genotypic information and statistical analysis, phenotypic information is necessary to detect markers and to train a yield prediction model, respectively [11–13]. Moreover, despite the advances and decreased costs in molecular techniques, information on the phenotype-genotype relationship remains limited. This so-called ‘genotype–phenotype gap’ [14] is mainly due to manual phenotyping that is labor-intensive, time-consuming, and error-prone. As phenomics or high-throughput phenotyping has gained more attention, the –omics technologies together with bioinformatics have not only led to an exponential generation of data, but also to means to mine and analyze them for information that will prove helpful in feeding the world [15].

Simple phenotypical assays to study plant growth and development are of great importance, especially to understand plant development in a physiological as well as agricultural relevant setting. Nowadays, applied plant research tends to focus more and more on improving the plant’s intrinsic yield and biomass for a biobased economy, whereas high productivity should be achieved without affecting the environment [16–20]. To this end, novel and natural growth-promoting substances have gained a lot of interest lately [21–23]. Rice can be used as a model crop to discover unknown natural compounds that stimulate plant growth and plant yield, but a rapid and reliable bioassay is needed to screen the impact of the natural compounds on rice growth. Increasingly, high-throughput plant phenotyping assays are established for the development of novel biostimulants, both large and small scale [24], which are also useful for toxicity testing in ecotoxicological research [25–30].

A well-described small-scale rice bioassay uses plants grown hydroponically [4]. The basic set-up of the hydroponic system consists of a container filled with solution for the nutrient acquisition by the rice roots. The container is covered by a lid with holes in which seedlings can be fixed by various support materials. The seedlings can either be directly sown or they can be pregerminated. As the nutrient composition of the root medium can be determined exactly, this method has been implemented widely, also for toxicity studies. However, most of these studies focused on the developing root only [31–35]. These hydroponics systems require special attention to avoid fungal and algal growths, making them less practical for highly controlled experiments. Another frequently used system is the wet-roll method that can also be used to pregerminate rice seeds before transfer to another system, such as the hydroponics system [4]. A filter paper cylinder with seeds between the sheets are incubated in a closed tap water-containing container. The seedling roots grow toward the water, while the leaves grow out of the paper into the humid air space [4, 34]. Culture dishes have been used also in small-scale set-ups. For example, in toxicity testing, the specimens are sterilized, the seeds are placed in a culture dish or an adapted seed tray device, and germination and radicle (root) and coleoptile (shoot) growths are checked [26, 30, 36]. In another system, seeds are sterilized, soaked, and then transferred to two sheets of sterile, solution-moistened filter paper into sterile Petri plates [37]. A different simple pregermination method for the study of rice seedling growth over a slightly extended time period employs sterile growth containers filled with Murashige and Skoog medium [38, 39]. A less environmentally controlled system is the use of soil, instead of agar [40]. The so-called sand + absorbent polymer (SAP) system was developed as a substrate for the xenic culture of plant-parasitic nematodes in laboratory settings [41], consisting of two devices, either the polyvinyl chloride (PVC) tube system to be used for a wide range of host plants and the polystyrene (PS) tube system suitable for rice only. Both systems could be utilized for the inoculation of seedlings with nematodes, as recent reported [42]. A drawback of these systems is that despite several washing steps, sand remains on the roots, preventing the precise determination of the root biomass. In 1970, a set-up had been established with some features that could be used in a broad range of applications [43], in which sterilized seeds were soaked in water, germinated in the dark, whereafter uniformly sized seeds were transferred to 1 mL of water in main bodies of Erlenmeyer flasks fitted with central wells containing 1 mL of water. As the aim was to check the effect of ethylene on rice growth, the Erlenmeyer flasks were fitted with vaccine caps and kept in the dark after an atmosphere had been introduced.

In vitro assays may be useful to speed up the process of preliminary screening to discover and characterize natural biostimulants [17]. Tests have been described in which seeds are germinated under sterile conditions in Petri dishes, flasks, or tubes, whereafter plants are grown on a liquid—i.e. hydroponically—or a solid medium in a controlled environment. In these set-ups, potential plant biostimulants can be added either to the solid or the liquid medium via foliar and/or root applications and even at different concentrations, to check for dose–response effects. Such experiments allow fast screening of growth responses and eliminate the influence of environmental parameters [17]. Nevertheless, the above-mentioned small-scale instances of phenotypical set-ups have some bottlenecks to make them useful for a broad range of applications. Pregermination is a crucial step in phenotypical studies, because the impact on seed germination needs to be uncoupled from that on seedling growth. Moreover, in most systems, the volumes in which the seedlings are grown, are quite large, possibly causing problems to discover novel biostimulants, because the molecules tested are often not available in large quantities. Accurate assessment of the effects of the test compound itself requires an assay system with minimal medium, without seedling competition, and a highly controlled growth environment [17, 44]. Large-scale methods have been developed, but most of them are laborious, require specialized facilities, and frequently result in wide variances in the obtained data [45]. Noteworthy, a small-scale set-up remains important to speed up the process of preliminary phenotypical screening before testing in large-scale set-ups. For overviews on the range of automated and semi-automated phenotyping platforms for the analysis of root growth and architecture, we refer to recent papers [46–48]. Regarding advancements in plant phenotyping and upcoming challenges and perspectives, recent reviews have been published [49, 50], but are beyond the scope of this article.

A reasonably fast system for the analysis of the rice seedling parameters of interest that could be computer-automated in a simple laboratory set-up would surely be an advancement in the field. To analyze image-based data, research groups need image analysis and storage tools that nowadays are becoming less of a problem, because more open-source software and resources are available [51]. However, the majority of the in-house developed software is made for specific experimental set-ups to address specific research questions [52].

In conclusion, a method is needed to screen rice seedlings in both a time- and cost-effective manner. Here, we propose a new phenotype-based methodology with a broad range of applications that allows a fast and reliable screening of rice seedling growth responses via an automated computer analysis. The automated computer vision analysis program was developed for the analysis of shoot-related parameters. This method was tested by pharmacological interference in the major hormonal pathways that influence rice growth and development as well as by subjecting seedlings to abiotic stresses (such as salt stress and inhibition of photosynthesis). The newly developed method was found to be valid and reliable. As proof of concept, we used our methodology to screen semi-purified fractions of marine invertebrates for their effect on rice seedling growth and discovered potential plant growth biostimulants, hence, providing an added value to this new method.

## Results

### Method development

#### Sterilization and pregermination

Disinfected rice seeds [53] were pregerminated in the dark under complete submergence in 2.0 mM sterile CaSO_4_ solution (cf. “Methods”). We opted for complete submergence, also for further manipulations, because rice seedlings, unlike other cereal crops, can germinate by elongation of their coleoptile and mesocotyl [54]. Moreover, 2.0 mM sterile CaSO_4_ was used, because sterile tap water resulted in too much variation (data not shown) and distilled water is unsuitable due to the lack of calcium ions that hampers germination and damages seedling roots [4, 55].

#### Set-up

After 3 days of pregermination, seedlings were selected and transferred under sterile conditions to 14-mL round-bottom tubes that contained 1 mL 2.0 mM CaSO_4_ to which a potential bioactive compound could be added for screening. Only seedlings at the same developmental stage (i.e. with radicles and coleoptiles of approximately the same size) were selected for further manipulations to avoid interference of growth effects with germination differences. Then, in each 14 mL round-bottom tube, only one seedling was placed, specifically to avoid competition within the tubes, as observed when numerous seedlings were added in large tubes (data not shown). The tubes were loosely capped to allow adequate aeration of the growing seedlings. As an optimal set-up of our experiments, we propose three biological repeats, each containing 15 technical repeats (i.e. 15 tubes with one seedling per tube), providing a total sample size of 45 seedlings for further analysis. However, the number of samples and repeats depends on the research question and experimental design.

Seedlings were grown at a day/night temperature of 28 °C/26 °C and a 12-h light/12-h dark regime (170 µmol m^−2^ s^−1^ light intensity). After 7 days, shoot and root parameters were measured by carefully placing the seedlings on square dishes and subsequently scanned for the analysis of the lengths of the total shoot, internode, first and second leaves, and coleoptile. To this end, the images were automatically imported into an in-house developed program, designated Plength, for the automated analysis of rice seedlings (see below) and the manual utilization of ImageJ, an open-source image processing program [56]. ImageJ was applied to measure the seminal root length and the crown root length. For a schematic overview of this workflow, which we labeled rice in vitro assay or RIVA, see Additional file 1: Figure S1.

### Automated computer vision analysis

**Fig. 1:**
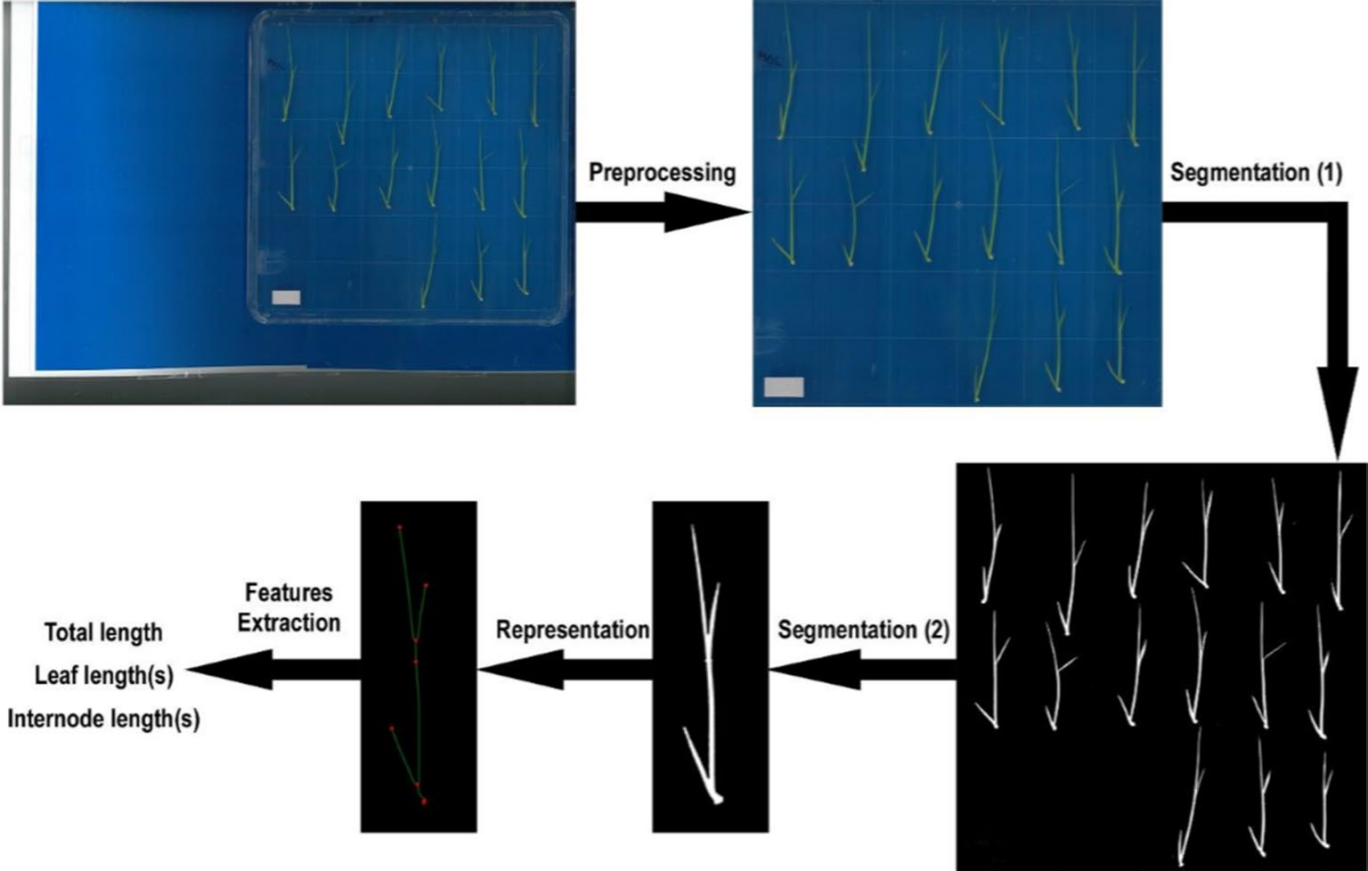
Overview of the image analysis pipeline. The sample image was preprocessed, segmented, and represented as a graph. Features of interest, namely lengths of total shoot, leaves, and internode, were extracted from the graph

The program Plength, (coding language: Python), can analyze multiple plants at once, providing the parameters of interest, which are shoot length, leaf lengths, internode length, as well as coleoptile lengths. In addition to returning the measurements for each seedling, also the mean, standard deviation, and standard error for each parameter are delivered for all the samples from the same image. The program follows a standard image processing pipeline for plant growth analysis, namely preprocessing, segmentation, and feature extraction [57] (Fig. 1).

Previous to the analysis, the image must be cropped manually to include the plate with plant samples and the scale bar. The subsequent steps are automatically executed by the program. In the following preprocessing step, the image is converted into a different color space, for which the most suitable for this purpose is hue, saturation, value (HSV) [58]. Next, the converted image is smoothed by median blurring, followed by bilateral filtering (Additional file 2: Figure S2). In the segmentation step, plant samples are separated from the background and partitioned for their individual analysis (Additional file 2: Figure S2). For this segmentation, color thresholding is used, because plants are positioned on the plates in such a manner that they do not overlap. The resulting image is black-and-white, i.e. binary. Before analysis, each connected white region must be recognized as an individual plant. To this end, the image needs to be partitioned, or segregated. Only the outline of each region, i.e. contour, is stored to save computation time. Note that the bottom corner of the image, where the scale bar is placed, is outlined separately to obtain a contour of the scale bar, of which the width (in pixels) is used for the later measurements. Then, before each contour is processed one at a time to determine individual shoot length, leaf lengths, and internode length, an extra representation step is done that skeletonizes the contour and converts it into a graph (Additional file 2: Figure S2). This graph is a structure consisting of nodes (i.e. vertices) connected to one another via edges. During the skeletonization, the binary objects are reduced to one-pixel-wide representations. For the graph transformation each point is designated as a terminal node, a branching node, or an edge, depending on whether they contain one, more than two, or exactly two neighbors, respectively. Before the conversion into the final graph, a pruning method must be implemented to eliminate unintended edges, such as the plate grid or the plant cut-off end. These unintended edges are usually quite short, so the pruning simply removes terminal edges that are shorter than 50 pixels, an empirically chosen threshold, with the exception of the edge below the lowest node.

In conclusion, Plength represents the plant as a graph, in which the ends and branching points are identified as nodes. By means of this graph, a path can be defined for each parameter, for instance, the shoot length as the path length from the lowest to the highest node. Finally, features can be extracted and each parameter of interest can be measured (i.e. total shoot length, internode length, and first and second leaf lengths).

Although the program was written for seedlings with two leaves, it will also be applicable for any other number of leaves, as long as the plants are carefully arranged on the agar plates prior scanning of the images, namely straight, nonoverlapping plants with open leaves (Additional file 3: Figure S3).

Plength can also analyze nonbranching tissues/organs, such as coleoptiles, but with some slight algorithmic differences when compared with the seedling analysis. For the coleoptile measurements, color information is not necessary, so the image can be converted to grayscale instead of HSV. The optimum value for the greyscale thresholding is determined as described [59], instead the manual selection of the seedlings. In this method, pixels are divided into two classes, background and foreground, and a threshold value is pursued that will maximize the variance between both classes. The scale bar is detected as well. Coleoptiles are not pruned after graph conversion to avoid difficulties when they are shorter than 50 pixels in length. Finally, the only parameter of interest in this set-up, is the total length. Dijkstra’s algorithm [60] was used to compute all the shortest paths from the lowest node to all the other nodes in the graph.

To make the program user friendly, Plength has a graphical user interface (GUI) (Additional file 4: Figure S4), so that it can be used without any prior coding knowledge. Both the source code as an installation guide can be found in Additional file 15. Some functionalities have been implemented in the GUI, including settings, preprocessing, and postprocessing steps (Additional file 4: Figure S4). The following four options can be changed under settings: plant type (coleoptile or seedling), scale bar position (left or right) and length (default set at 20 mm), and minimum detection area (used to remove noise and by default set empirically at 500 pixels). The preprocessing option can be used at the start of the analysis. The detected areas in the image can be cropped or checked. After analysis, the measured parameters are displayed in a text box. A new labeled image is obtained with all the detected areas (i.e. individual plants) framed and numbered that can be exported. Edges classified as leaves are traced with a yellow line. Importantly, based on the labeled image, the user can check whether the program made any mistakes in the detection or classification steps. Errors, such as misclassification, truncation, or underestimation can be fixed during the postprocessing or avoided entirely by placing the seedlings as recommended (see above). Unwanted or looked-for regions can either be removed or selected. Regions can also be merged, so that portions of the cut-off stem or leaf can be added to the main plant. When different treatments are tested on the same plate, they can be grouped and the mean, standard deviation, and standard error will be calculated separately for each group. During grouping, certain parameters can be removed from the plant, because the output text box can be edited directly. Finally, the results can be exported to a comma-separated value (csv) file.

The automated computer vision analysis system described above was validated by comparing the measurements made by Plength with manual measurements done with ImageJ (Table 1). In total, 127 samples were used. All parameters had a normal distribution (tested with the Shapiro–Wilk test, *P* > 0.05) and F-tests confirmed that variances of the manual and program-made measurements did not differ significantly (*P* > 0.05). The difference between the average measurements made by both methods was not statistically significant as determined by a Student’s two-sided *t*-test, indicating that Plength can be used for the accurate determination of growth-related parameters of rice seedlings.

**Table 1:** Comparisons between measurements (in mm) made by Plength (program) and ImageJ (manual)

Length	Mean	Standard deviation	Standard error	*P* value*
Total shoot				
Program	66.60	8.00	0.71	0.43
Manual	65.81	7.95	0.71	
Internode				
Program	30.76	3.19	0.28	0.37
Manual	30.41	2.99	0.27	
First leaf				
Program	16.82	1.50	0.13	0.07
Manual	16.49	1.43	0.13	
Second leaf				
Program	12.66	2.38	0.21	0.53
Manual	12.84	2.03	0.18	

^*^Student’s two-sided *t*-test (*n* = 127)

### Method validation by pharmacological interference in the major hormonal pathways

**Fig. 2:**
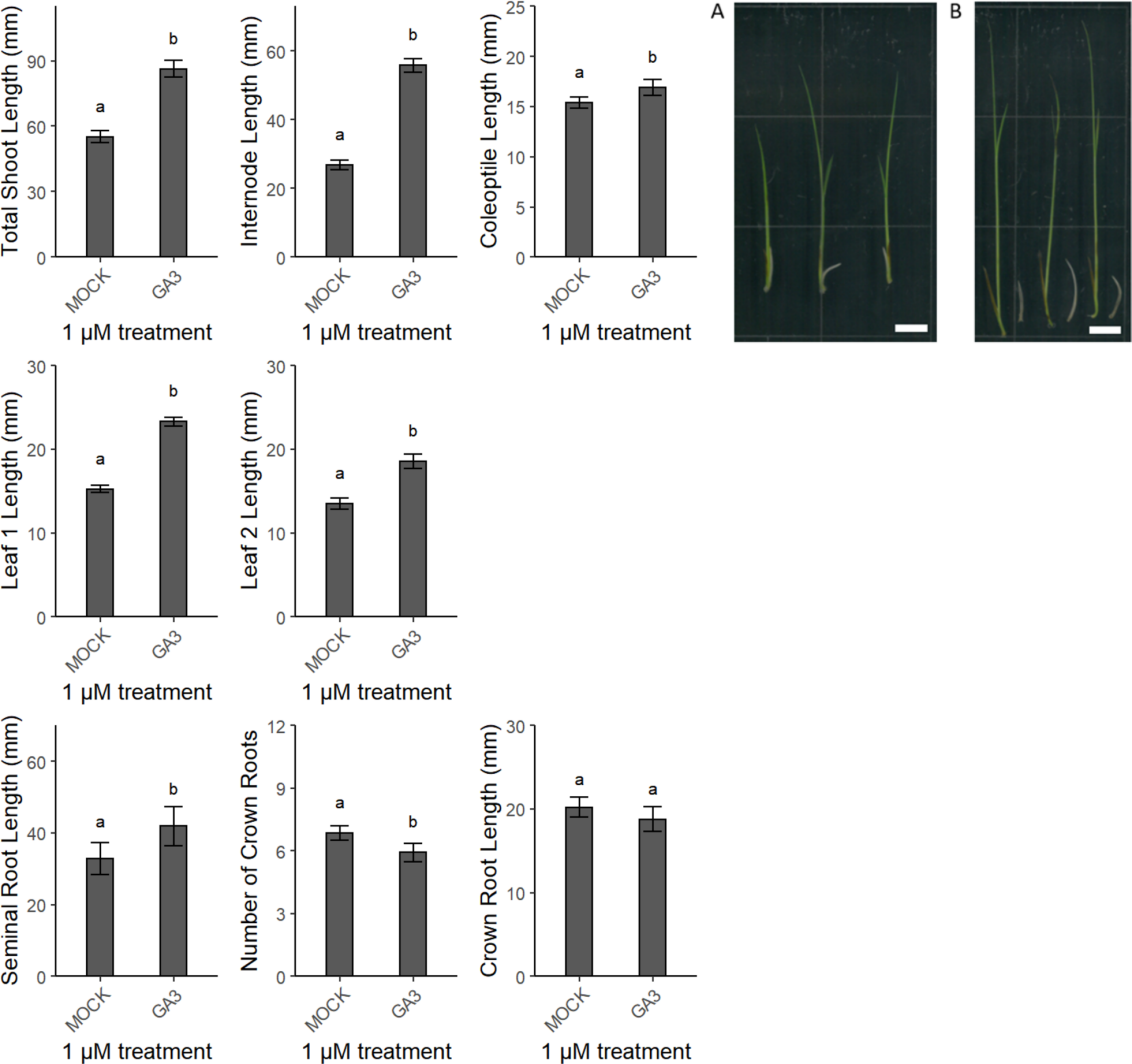
Validation of the screening method by means of 1 µM gibberellic acid (GA_3_). The shoot parameters (in mm) are lengths of total shoot, internode, coleoptile, and leaves 1 and 2. The root parameters are lengths (in mm) of the seminal and crown roots and the number of emerged crown roots. Different letters indicate statistically significant differences between the treatments (see “Methods”). The picture shows shoots of harvested rice seedlings with a clear difference between treatments, **a** Mock and **b** 1 µM gibberellic acid. Scale bar = 1 cm

The developed assay was validated by adding to the test tubes 1 µM of various plant hormones or molecules that interfere with their action mode. The effect of these molecules was assessed after 7 days of treatment (see above). Experiments were repeated up to 4 times for each treatment. Root parameters were manually measured (through ImageJ). To test the rice seedling response to auxin, the synthetic auxins naphthalene acetic acid (NAA) and dichlorophenoxyacetic acid (2,4-D), the main natural auxin in most plants, indole acetic acid (IAA), and an inhibitor of the auxin efflux, *N*-1-naphthylphthalamic acid (NPA) were used as treatment. Upon the 2,4-D treatment (Additional file 5: Figure S5), mainly the lengths of the total shoot, the internode, and leaf 2 decreased. The seminal root was reduced upon NAA and NPA treatments, whereas the number of crown roots decreased upon NPA treatment (Additional file 5: Figure S5). The root system of seedlings treated with 2,4-D could not be measured and showed a ‘stumpy’ phenotype (Additional file 5: Figure S5). Kinetin, *trans*-zeatin, and 6-(γ,γ-dimethylallylamino)purine (2iP), were added to test the effect of cytokinins. The effect on the shoot was the strongest for the leaf elongation upon *trans*-zeatin treatment (Additional file 6: Figure S6). Moreover, the seminal and crown roots were shortened upon kinetin and *trans*-zeatin treatments (Additional file 6: Figure S6). The addition of the brassinosteroids brassinolide and bikinin, a strong brassinosteroid signalling activator, mainly affected the roots (Additional file 7: Figure S7). Brassinolide decreased the lengths of the seminal and crown roots, but the number of crown roots increased. Treatment with gibberellic acid (GA_3_), a widely available gibberellin that has a very high relative activity on the shoot elongation in rice seedlings [61], increased the lengths of all the shoot-related parameters and of the seminal root, whereas the number of crown roots decreased (Fig. 2).

**Fig. 3:**
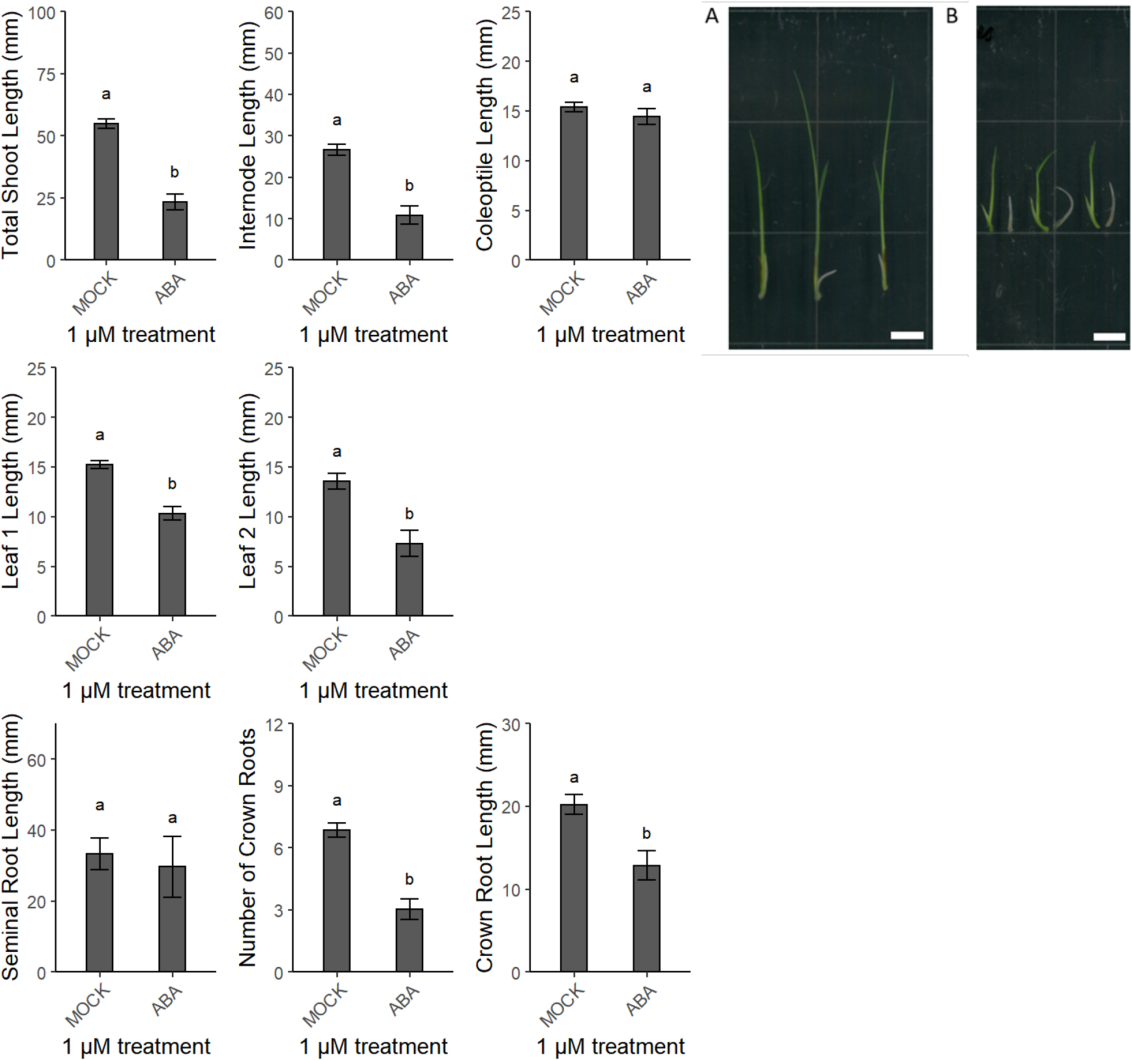
Validation of the screening method by means of 1 µM abscisic acid (ABA). The shoot parameters (in mm) are lengths of total shoot, internode, coleoptile, and leaves 1 and 2. The root parameters are lengths (in mm) of the seminal and crown roots and the number of emerged crown roots. Different letters indicate statistically significant differences between the treatments (see “Methods”). The picture shows shoots of harvested rice seedlings with a clear difference between treatments, **a** Mock and **b** 1 µM abscisic acid. Scale bar 1 = cm

In contrast, seedlings treated with abscisic acid (ABA) had shorter shoot-related parameters (except for the coleoptile length) than the mock-treated seedlings, whereas the length and number of emerged crown roots decreased as well (Fig. 3).

The effect of ethylene was investigated by adding 1-aminocyclopropane-1-carboxylic acid (ACC), a crucial intermediate in the ethylene production, and AgNO_3_, an ethylene perception inhibitor [62]. AgNO_3_ had a noticeable impact on the lengths of the seminal and crown roots (Additional file 8: Figure S8).

**Fig. 4:**
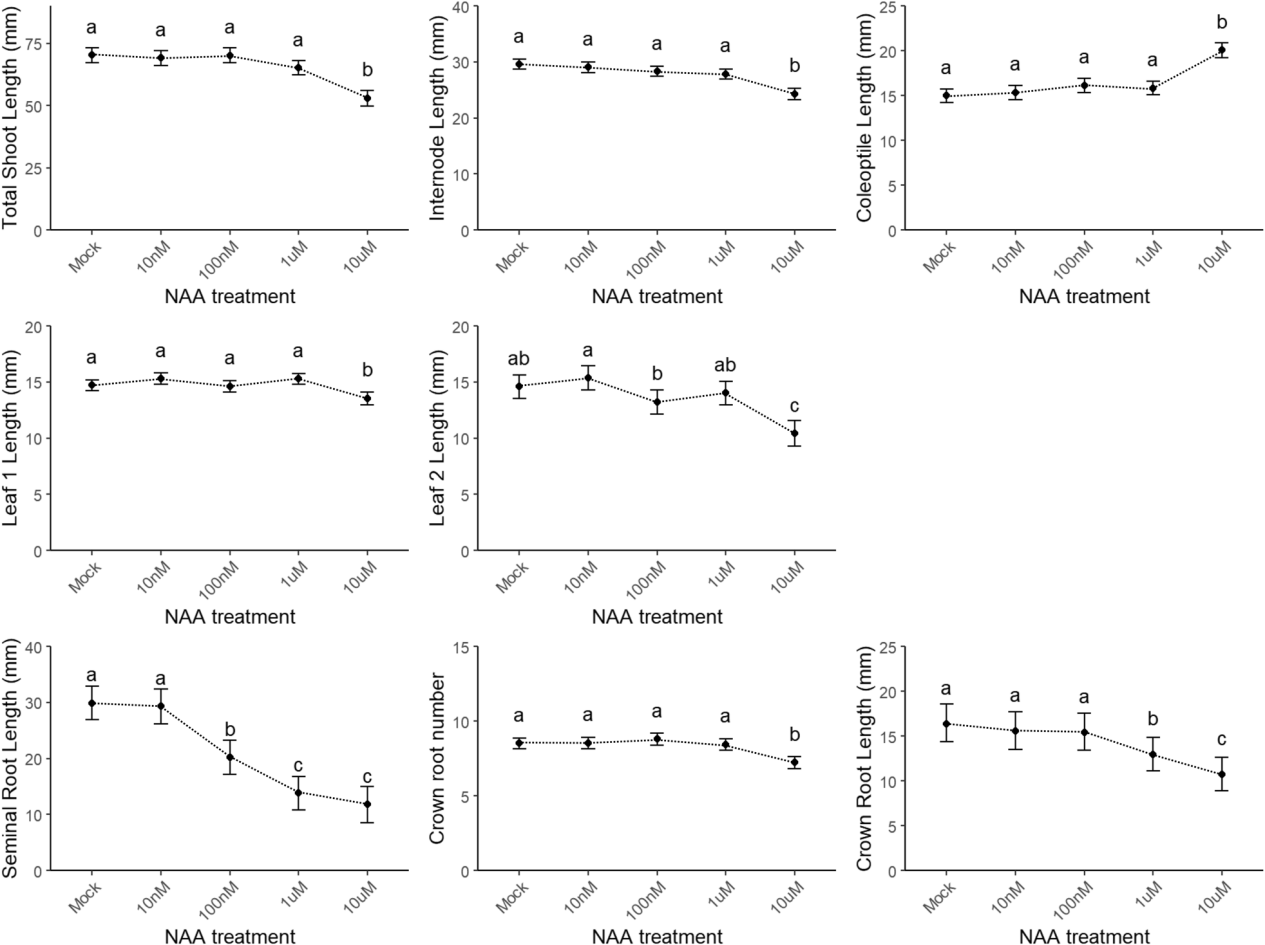
Validation of the screening method by means of a concentration range of 1-naphthaleneacetic acid (NAA). The shoot parameters (in mm) are lengths of total shoot, internode, coleoptile, and leaves 1 and 2. The root parameters are lengths (in mm) of the seminal and crown roots and the number of emerged crown roots. Different letters indicate statistically significant differences between the treatments (see “Methods”)

**Fig. 5:**
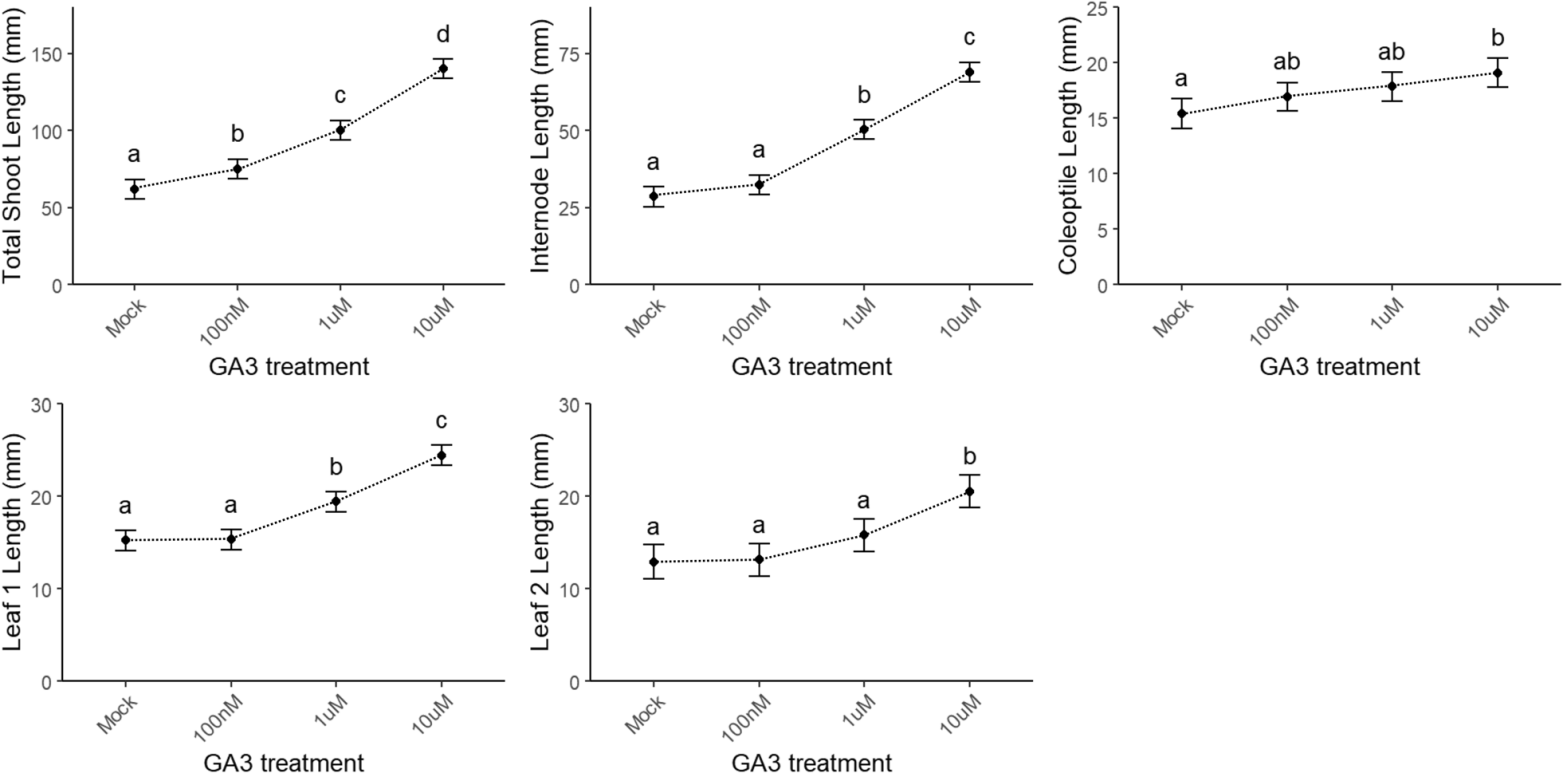
Validation of the screening method by means of a concentration range of gibberellic acid (GA_3_). The shoot parameters (in mm) are lengths of total shoot, internode, coleoptile, and leaves 1 and 2. The root parameters are lengths (in mm) of the seminal and crown roots and the number of emerged crown roots. Different letters indicate statistically significant differences between the treatments (*n* ≥ 14) (see “Methods”)

Besides testing the representatives of the major plant hormone classes, we validated and checked the robustness of the assay through a concentration range experiment with NAA and GA_3_ that were specifically chosen, because NAA inhibits (root) growth and GA_3_ stimulates shoot growth (Figs. 4 and 5, respectively). After NAA had been added in the test tubes at concentrations of 10 nM, 100 nM, 1 µM, and 10 µM, the shoot and root growth parameters were analyzed (Fig. 4).

For the shoot parameters, an increase in coleoptile length was detected at the highest NAA concentration, whereas the lengths of the internode, the total shoot, and leaves 1 and 2 decreased at high NAA concentrations. This decrease in length with increasing concentrations was even more clear when root parameters were analyzed, namely the lengths of the seminal and crown roots decreased significantly with increasing NAA concentrations, hinting at a concentration-dependent response to the treatment. Similarly, such a concentration-dependent response was also observed when 100 nM, 1 µM, and 10 µM GA_3_ was added in the set-up (Fig. 5). Although repeated only once (*n* ≥ 14), a clear concentration-dependent increase in the lengths of the total shoot, internode, and leaves 1 and 2 indicates that our developed method can also assess shoot parameters that vary in a concentration-dependent manner.

### Method validation by abiotic stress submission

Next, the effect of different abiotic stresses on rice seedlings of different cultivars [*Oryza sativa* (L.) cv. (New) Dongjin, cv. Chucheongbyeo and cv. Chilbo, a semi-dwarf variety] was assessed after 7 days of treatment (see above), which also allowed to check for cultivar dependent responses. Experiments were repeated up to 4 times for each treatment. Root parameters were manually measured (through ImageJ).

**Fig. 6:**
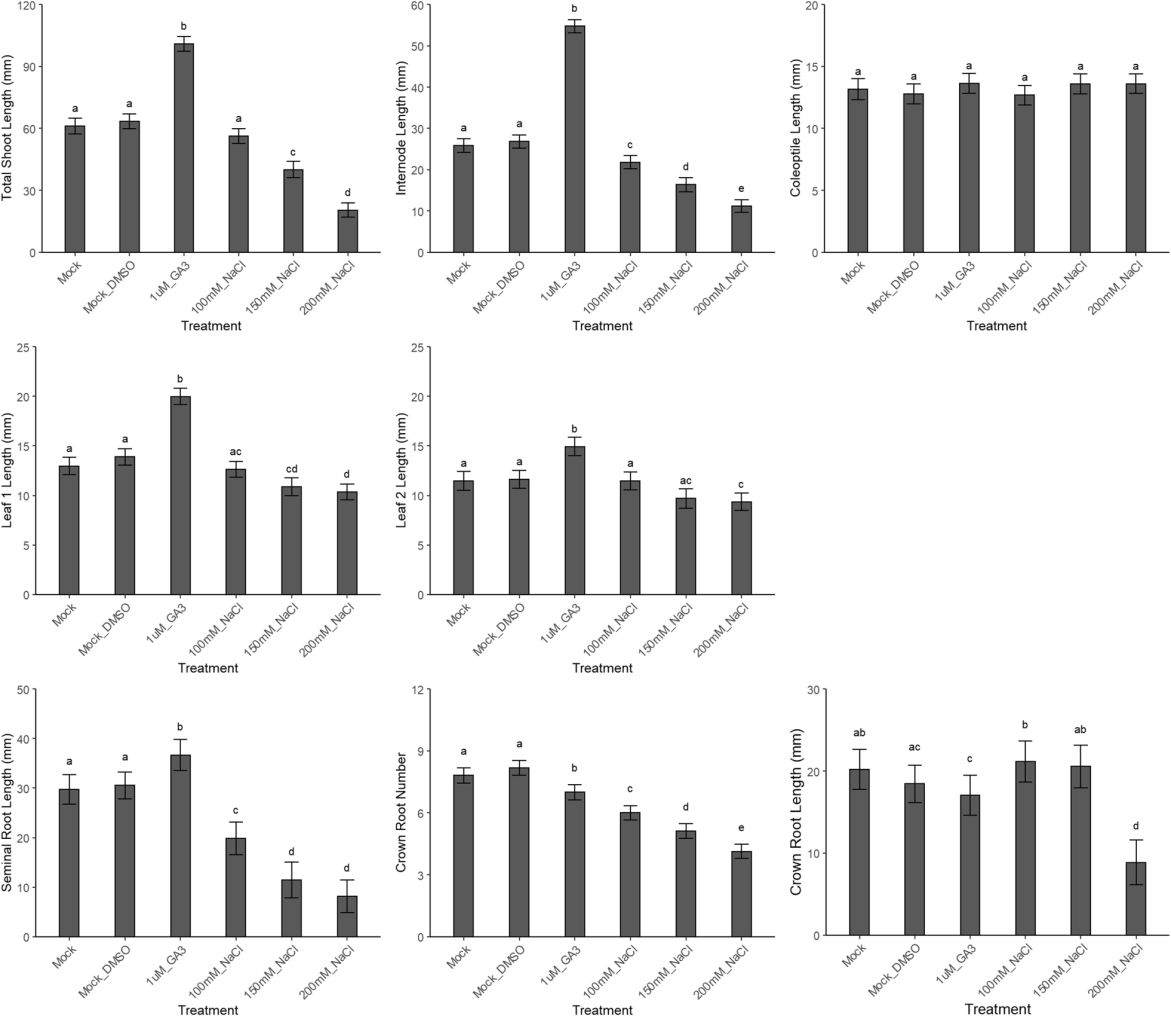
Validation of the screening method by means of a concentration range of NaCl in *Oryza sativa* (L.) cv. (New) Dongjin. Also 1 µM GA_3_ was added in the test tubes. Mock_DMSO corresponds to GA_3_ as this is also dissolved in DMSO, while NaCl is dissolved in sterile dH_2_O. The shoot parameters (in mm) are lengths of total shoot, internode, coleoptile, and leaves 1 and 2. The root parameters are lengths (in mm) of the seminal and crown roots and the number of emerged crown roots. Different letters indicate statistically significant differences between the treatments (see “Methods”)

*Oryza sativa* (L.) cv. (New) Dongjin, cv. Chucheongbyeo and cv. Chilbo all showed a concentration dependent decrease in total shoot length, internode length, leaf 1 and leaf 2 lengths, together with a decrease in seminal root length, crown root length and number of emerged crown roots upon treatment with 100, 150 and 200 mM of NaCl. Except for coleoptile length, seminal root length and crown root length, we did not observe a cultivar dependent response upon treatment with GA_3_ (Fig. 6, Additional file 9: Figure S9 and Additional file 10: Figure S10, respectively) (Additional files 11 and 12 for least squares means and pairwise comparisons for each parameter for the different cultivars tested, respectively).

We also added 100 µM, 150 µM, 200 µM and 500 µM DCMU, i.e. 3-(3,4-dichlorophenyl)-1,1-dimethylurea, a very specific and sensitive inhibitor of photosynthesis, to the test tubes. Also here, a concentration dependent decrease in total shoot length and internode length was detected together with shorter first leaf lengths. However, here was no inhibitory effect on leaf 2 lengths and we observed increasing coleoptile lengths. Moreover, the seedling roots showed a strong inhibitory response to the DCMU treatment (Additional file 13: Figure S11).

These concentration range experiments and lack of cultivar-specific shoot responses indicate the validity and robustness of the developed method also when seedlings are subjected to abiotic stresses.

### Method application by semi-purified fractions of marine invertebrates

Our newly developed and validated phenotype-based screening method RIVA, linked to an automated computer vision analysis system Plength, was used to screen possible effects of semi-purified fractions of marine invertebrates on rice seedling growth. These fractions were kindly provided by Chris Ireland’s group (University of Utah, Salt Lake City, UT 84112, USA), who had created a protocol for fractionating marine invertebrate extracts and had developed a marine natural product library (MICL), derived from an extensive collection of unique marine organisms from diverse locations around the world, *i.e*. sponges (85% over 150 genera), tunicates (12%), and 2% other phyla [63, 64]. The full MICL library currently contains over 30,000 wells of semi-purified marine materials, of which a representative sublibrary of 240 fully characterized marine natural products has been established (MICL240). Here, for the first time, we wanted to exploit part of the MICL library to find novel, unanticipated plant growth biostimulants from marine organisms. The HP20 fractions, obtained from an improved fractionation strategy by means of Diaion HP20SS, a porous polystyrene-based absorbent [64], were tested in one repeat in our set-up at a concentration of 0.5 µg/mL and were given a serial number for convenience purposes (Table 2).

**Table 2:** Fractions of marine invertebrates, obtained from the MICL library

Assigned serial number	Fraction code	Species
1–4	PNG11-1-001 F1-4	*Dysidea granulosa*
5–8	PNG11-2-009 F1-4	*Stylissa massa*
9–12	PNG11-3-026 F1-4	*Pipestela candelabra*
13–16	PNG11-3-031 F1-4	*Candidaspongia flabellata*
17–20	PNG11-4-034 F1-4	*Neamphius huxleyi*
21	PNG11-5-044 F1	*Agelas* sp.
22–24	PNG11-5-045 F1-4	*Carteriospongia lamellosa*
25–28	PNG11-6-056 F1-4	*Erythropodium* sp.
29–32	PNG11-9-091 F1-4	*Plakinastrella* sp.
33–36	PNG11-9-096 F1-4	Verongida
37–40	PNG11-18-131 F1-4	*Haliclona* sp. red

A total of 40 HP20 fractions were tested derived from 11 different marine invertebrates

**Fig. 7:**
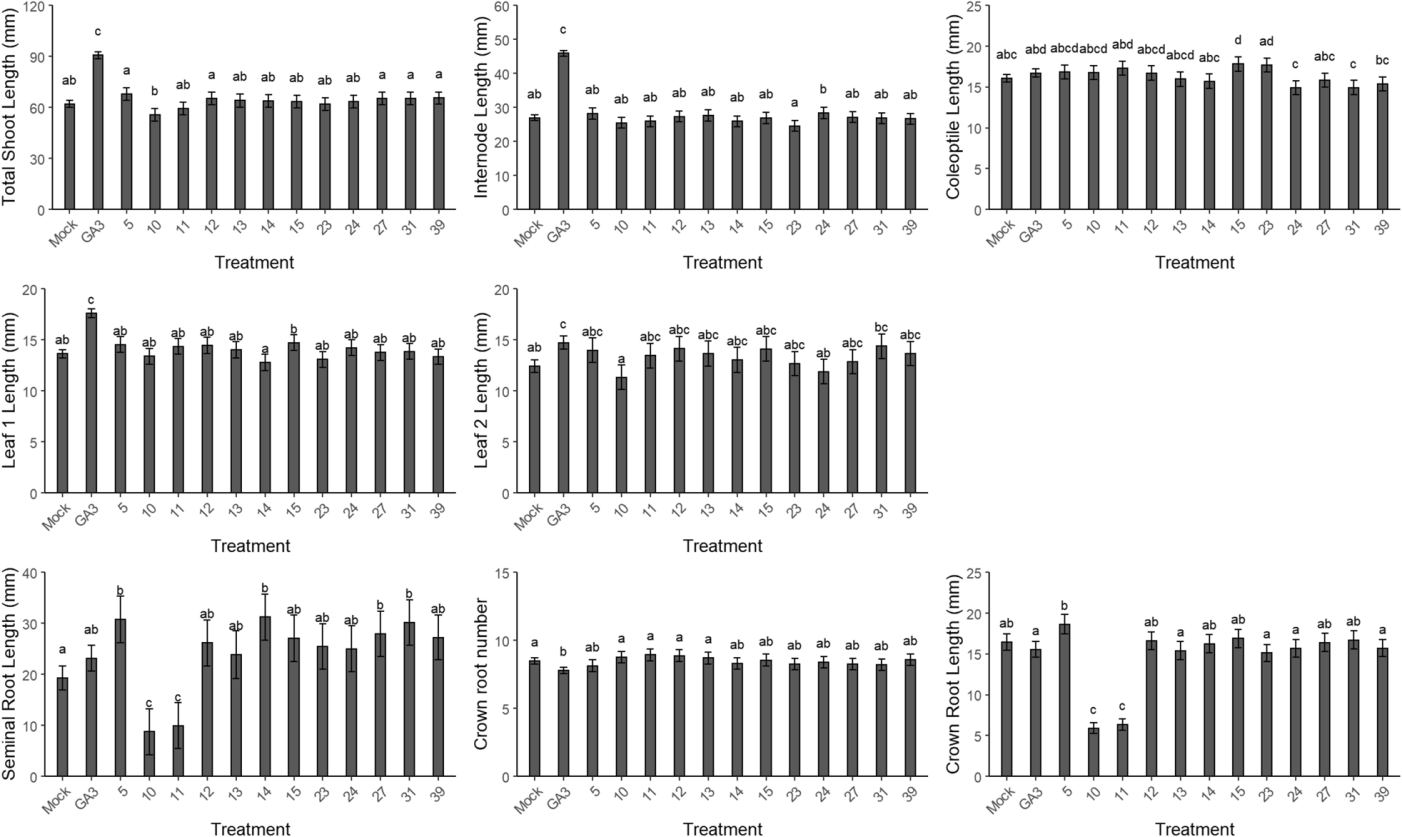
Application of the screening method by means of 0.5 µg/mL of the MICL library fractions. After one repeat, only the fractions that differed from the mock were repeated two more times and are presented. The shoot parameters (in mm) are lengths of total shoot, internode, coleoptile, and leaves 1 and 2. The root parameters are lengths (in mm) of the seminal and crown roots and the number of emerged crown roots. Different letters indicate statistically significant differences between the treatments (see “Methods”). As a control, 1 µM gibberellic acid (GA_3_) was used

For fractions with shoot and/or root parameters differing from the mock samples, as discussed above, the experiment was repeated two more times. For the selection of potential growth-altering fractions, no significant differences were found for the shoot parameters (Fig. 7) as well as for number of crown roots. However, at the concentration tested, seedlings treated with fractions 10 and 11, both originating from the same sponge (*Pipestela candelabra*), had significantly shorter seminal and crown root lengths than those of the mock samples, but, by contrast, seedlings treated with fractions 5, 14, 31, and 27, derived from the sponges *Stylissa massa*, *Candidaspongia flabellata*,* Plakinastrella* sp*.*, and the coral *Erythropodium* sp*.*, respectively, all had significantly longer seminal roots.

## Discussion

### Method development

As some of the published methods have proven to be rather difficult to adjust to specific laboratory settings and research questions, we developed a new small-scale in vitro method. The phenotype-based method developed and validated here was used to screen for biostimulants, but could potentially also be utilized for other specific research questions, such as mass phenotyping of mutant lines [65], for growth experiments under specific stresses, and might potentially even contribute to screening of agricultural traits [10, 66]. For the latter however, the specific lab-setting of the RIVA screening method needs to be taken into account, as results obtained in the lab and in controlled conditions do not necessarily translate into field-applications. Also abiotic stresses, such as salt, chilling, and drought stresses, and/or the effect of biotic stresses could be examined. However, for such experiments, the majority of the current methods are laborious, require specialized facilities, frequently exhibit wide variances in data, and are typically of a much larger scale [45].

Our proposed small-scale in vitro seedling screening method has a lot of advantages compared to existing methods and has a wide range of possible applications. First of all, it is a simple laboratory set-up, making it cost effective and also time effective, because it is quite rapid with a computer vision-aided analysis, and as such is user independent. Parallel testing of large amounts of samples in a whole range of concentrations is possible and, because the assay is miniaturized, the biological response evaluation is simple and fast [17, 67, 68]. Moreover, seedlings are grown in a highly controlled growth environment in a minimal medium without seedling competition and germination effects, hence eliminating the influence of soil and other environmental parameters [17, 44]. Field phenotyping systems have been claimed to be more advisable than phenotyping in controlled environments. A pipeline has been proposed, in which screens are done under real field conditions, whereafter, but only in a second instance, small-scale phenotyping platforms in controlled environments can be implemented for mode of action research [24]. Nevertheless, screening assays in a controlled environment are more time and cost efficient, because the number of compounds to be tested in a field trial for biostimulants can be drastically narrowed down [24]. Thus, in vitro assays may be useful to speed up the process of preliminary screening [17] and might be helpful. Moreover, bioassays with simple read-outs have been acknowledged [24] to be instrumental for the discovery of plant or tissue traits as well as for the mode of action research. Additionally, it allows one to define the best application method, timing, and rates and provides preliminary indications on the potential phyotoxicity of biostimulants [17]. Furthermore, the small volumes used in our set-up, only 1 mL of 2.0 mM CaSO_4_ needed per seedling, is advantageous. Nevertheless, a new commercial product developed for agriculture still requires large-scale field testing.

Although our automated computer vision analysis program Plength had been written for rice seedlings, we also tested it successfully for the analysis of wheat seedlings (Additional file 14: Figure S12), making it adequate for laboratory use. Nevertheless, the simple laboratory set-up can be potentially improved. Plength was developed for the automated computer analysis of shoot parameters, but not for root parameters. However, for in-depth characterization, the below-ground features, referring to the root system architecture and its function, should not be neglected [24]. Therefore, we used ImageJ [56]. Although specific software had been established for the automated analysis of the rice root system, it has mostly been used for root systems of older rice plants, such as the three-dimensional root phenotyping by the RootReader3D [69]. A more interesting tool that could be used in our set-up, pending some minor adaptations, is the RootReader2D software that is freely and publicly available and can be used for the thin, fibrous root system of rice [70]. However, in most cases, the automated measurement of the root system is still error-prone, especially for large crop species [71]. Our method analyzes rice seedlings in a destructive manner, which suits our goals, but may not be ideal for others. High-throughput phenotyping technologies have been developed that can non-destructively monitor plant traits, allowing time series measurements of individual plants at a high resolution [24, 49, 50].

### Method validation

We validated our method by evaluating the effect of disrupting hormonal balances through pharmacological approaches. Our results are mostly in line with data retrieved from the literature. For instance, the root system of rice seedlings treated with 2,4-D showed a ‘stumpy’ phenotype, as reported previously [30, 72]. For seedlings treated with 1 µM NPA and NAA, the seminal root length was significantly reduced. Previously, application of 20 µM NPA had been found to increase the root length [73], whereas the application of 300 nM NPA inhibited the root elongation [74]. For rice plants grown in the presence of 1 to 1000 nM NAA, the seminal root length decreased with increasing concentration [75], in agreement with our findings. Also, the concentration-dependent decrease of the different shoot organ lengths for NAA-treated seedlings concurs with previous work [76, 77]. Although NAA is generally considered to cause shoot elongation, results regarding growth upon NAA treatment in cereal plants, including rice, are variable [76], mainly due to the developmental stage by the start of the treatment or the time point of the analysis of the treatment impact. When the effect of the NAA treatment on early rice growth had been investigated, not only tillering and foliar production, but also shoot elongation were suppressed, whereas these effects were abolished to a great extent at later stages [77]. Moreover, NAA has been shown to inhibit rice primary root elongation in a dose-dependent manner [78], corresponding to previous findings in *Arabidopsis thaliana* [79] and the root phenotype observed in our NAA concentration range.

The genetic control of root development in rice and the gene regulatory network regulating the crown root initiation and development are well known [80]. A cytokinin response is known to inhibit crown root development and initiation, but we did not find a difference in crown root number upon addition of cytokinins, although the crown root length was significantly reduced after treatment with kinetin and *trans*-zeatin. Nevertheless, the seminal root length for both of these treatments was shortened, in line with the previously reported significantly decreased seminal roots upon treatment with 1 µM *trans*-zeatin [81]. Our data revealed that the lengths of the internode and the leaves had increased for seedlings treated with *trans*-zeatin. Treatments with various concentrations of kinetin or precursors had been found to quite effectively increase the root growth of older rice plants [82], in contrast to our results, probably because early seedling stages had been tested.

Brassinolide stimulates the elongation of mesocotyl and coleoptile, but inhibits the internode growth of rice seedlings [83]. In our experimental set-up, brassinosteroids stimulated growth of the coleoptile, but decreased the total shoot length and not the internode length. Previously, the brassinosteroid treatment had been observed to significantly promote coleoptile growth, whereas a relatively high concentration of brassinosteroids inhibited both root and seedling growths [84] and also reduced the root systems in the concentration we used [85]. For the root parameters, we found that brassinolide triggered a decrease in the seminal and crown roots, but apparently an increase in the number of crown roots.

The validation of shoot parameters and of the seminal root length revealed that GA_3_ stimulates the elongation growth in a concentration-dependent manner, corresponding with the overall consensus that gibberellins have a growth-promoting effect [61, 86–89]. On the contrary, ABA is generally accepted to inhibit shoot growth [86, 87]. In our experimental set-up, we also saw a negative effect of ABA on shoot and root growths, also in agreement with previous work [90]. Finally, we observed a significant increase in seminal root length upon treatment with the ethylene perception inhibitor AgNO_3_, also corresponding to previous findings [91]. An ACC treatment had been found to significantly increase the shoot and decrease the root [92], but in our experiment, the length of total shoot had significantly increased, but that of the internode and the root did not differ significantly.

We also validated our method by subjecting the seedlings to abiotic stresses and furthermore checked for cultivar-specific responses, which were in general not found. Although we only tested three cultivars, this confirms the robustness of the developed method. The results obtained for salt stress and photosynthesis inhibition are in line with data retrieved from the literature. Several authors found concentration dependent NaCl inhibitory effects on rice seedling growth in a similar concentration range as in our set-up [93, 94]. It is moreover generally accepted that significant reductions in mean root length, mean root numbers per plant and shoot length occur under increased salt stress, which is in line with our findings [95]. DCMU, i.e. 3-(3,4-dichlorophenyl)-1,1-dimethylurea, also called diuron, is a commonly used inhibitor in photosynthesis studies. In low application rates, it is used to selectively control grass weeds, while at higher rates, it is a nonselective weed killer [96]. In Arabidopsis, it was found that DCMU treatment strongly inhibited seedling root and vegetative growth, corresponding to our findings in rice [97].

Our data not only indicate that the method is valid, but also that it is consistent and robust, because the variance is low and, regarding reproducibility, every repeat gave similar and anticipated results.

### Method application by semi-purified fractions of marine invertebrates

Using the RIVA method, we tested a subfraction of the MICL, a marine natural product library and a unique resource for the discovery of novel small molecules with biological activities in a variety of systems [63, 64]. Screens by means of MICL have already identified novel modulators of diverse biological processes, such as Breast Cancer gene 2 (BRCA2)-deficient chemotherapy resistance, protein kinase C ζ (PKCζ), hypoxia-inducible factor 1 (HIF-1), wingless-related integration site (Wnt) signaling, phenotype induction in zebrafish and cystathionine β-synthase activity [63, 98–103]. Alkaloids from marine sponges have been demonstrated to stimulate initial development stages of agricultural plants [104]. Also, the stimulatory effect of merosesquiterpenoids from marine sponges and aaptamine alkaloids has been determined on the seedling root growth of agricultural plants, such as wheat, buckwheat (*Fagopyrum esculentum*), soybean (*Glycine max*), barley, and maize [105, 106]. Molecules of marine sponge origin have also been reported to regulate plant growth, namely two isolated indoles displayed a positive effect on plant growth in lettuce (*Lactuca sativa*) seedling root growth assays [107], demonstrating that marine organisms may contain molecules that stimulate plant growth.

Root growth was inhibited by fractions originating from *Pipestela candelabra* and seminal roots were stimulated by fractions from *Stylissa massa*, *Candidaspongia flabellata*, *Plakinastrella* sp., and *Erythropodium* sp*.* These data point out that the fractionation protocol used for the subfractions [64] sufficiently separates compounds, because not all the fractions from one organism were active; for instance, fractions 9–12 all originate from *Pipestela candelabra*, whereas only fractions 10 and 11 had an inhibitory effect on root growth. Furthermore, the selectivity of our newly developed phenotype-based screening method is demonstrated, for the reason that not all the fractions were active and that the activity was detected in fractions of different polarity.

Cytotoxic peptides from *Pipestela candelabra* have been documented in several cytotoxicity studies [108, 109]. *Stylissa massa* commonly produces alkaloids with a variety of biological activities [110, 111]. *Candidaspongia flabellata* contains cytotoxic candidaspongiolides, i.e., tedanolides analogs, with various biological activities [112, 113]. *Plakinastrella* has been reported to produce cyclic peroxides, polyketides, and peroxylactones with various activities [114–116]. *Erythropodium* apparently produces only compounds, called briarane diterpenes. Recently, fragilides have been reported to possess anti-inflammatory properties [117].

These data also indicate that the developed method can be used to detect the impact of natural compounds on rice seedling growth that are found in fractions or maybe even complex mixtures of marine invertebrates.

## Conclusions

A robust and simple phenotype-based screening method was established and validated. This new method, designated RIVA, allows the time-efficient and cost-efficient screening of rice seedling growth. An automated computer vision system, designated Plength, was developed that can be used as standard image analysis software for the measurement of shoot parameters of rice seedlings. We propose this set-up as a small-scale in vitro method in a controlled experimental environment for initial tests with a broad range of applications. Possibly, this method can be applied to screen for novel biostimulants, as illustrated with the fractions of the marine natural product library MICL.

## Methods

### Seed sterilization

Experiments were carried out with *Oryza sativa* (L.) cv. (New) Dongjin and with *Oryza sativa* (L.) cv. Chucheongbyeo and *Oryza sativa* (L.) cv. Chilbo, a semi-dwarf variety, for the salt stress experiment. All cultivars were obtained from the Gimpo Agricultural Technology Center (Gimpo, South Korea). Seeds were sterilized as described [53]. In short, the seeds were dehusked and the selected seeds were surface-sterilized with 70% (v/v) ethanol for 1 min, followed by a 1-min washing step in sterile distilled H_2_O. Then the seeds were subjected to 2.5% (v/v) bleach for 10 min, three times to sterile distilled H_2_O for 1 min, once to 0.1% (w/v) mercuric chloride for 3.5 min, and five times to sterile distilled H_2_O for 1 min. During these steps, seeds were continuously shaken.

### Seedling growth

The sterilized seeds were pregerminated in Petri dishes (90 × 20 mm; SPL Life Sciences) containing a sterile filter paper (90 mm diameter; Whatman) by complete submergence in 2.0 mM CaSO_4_ [4, 118]. The Petri dishes were closed with 3 M Surgical Tape (Micropore) and kept in continuous darkness at a 12-h/12-h day/night cycle and 28 °C/26 °C temperatures. After 3 days, seedlings with a similar radicle and coleoptile development were selected. These seedlings had an elongated coleoptile, but no apparent first leaf yet and a radicle of at least 5 mm. The selected seedlings were transferred under sterile conditions to 14-mL polypropylene round-bottom tubes (Falcon) containing 1 mL of 2.0 mM CaSO_4_ either supplemented with a test agent (pure compound or extract of interest) or none (control). Each test tube contained one seedling. Test tubes were loosely capped to allow adequate aeration of the rice seedlings and were placed in the growth chamber at a 12-h/12-h day/night cycle and 28 °C/26 °C temperatures and light intensity of 170 µmol m^−2^ s^−1^.

### Seedling analysis

After 7 days, seedlings were analyzed. Shoots, coleoptiles, and roots were dissected, put separately on a square dish (245 × 245 × 20 mm; SPL Life Sciences) filled with 0.66% (w/v) Plant Tissue Culture Agar (Neogen), and scanned (in color at 600 dpi), whereafter shoot and coleoptile images were analyzed with the in-house developed program for the automated analysis of rice seedlings, designated Plength. For contrast purposes, a blue background was placed behind the plate and a 2-cm scale bar was included in one of the lower corners as reference. Root parameters were measured manually with ImageJ [56, 119]. The Python source code of Plength can be found in Additional file 15 and a manual for the easy installation of this program’s graphical user interface in Additional file 16.

### Validation

The method described above was validated by interference with hormonal pathways (Table 3). A test compound (1 µM) was added to the tubes and the effect analyzed after 7 days of treatment. A concentration range for NAA and GA_3_ was done as well. The method was further validated by subjecting the seedlings to abiotic stresses by adding 100 mM, 150 mM and 200 mM NaCl or 100 µM, 150 µM, 200 µM and 500 µM DCMU. Each set-up consisted of 15 samples per treatment and was repeated up to 4 times.

**Table 3:** Compounds and concentrations added to interfere with the hormonal pathways to validate the method

Hormonal pathway	Interfering compound	Concentration
Auxin	2,4-D, IAA, NPA	1 µM
	NAA	10 nM, 100 nM, 1 µM, 10 µM
Cytokinin	Kinetin, 2iP, *trans*-zeatin	1 µM
Brassinosteroids	Bikinin, brassinolide	1 µM
Gibberellic acid	GA_3_	100 nM; 1 µM; 10 µM
Abscisic acid	ABA	1 µM
Ethylene	ACC, AgNO_3_	1 µM

### Semi-purified fractions of marine invertebrates

The fractions of marine invertebrates, kindly provided by Chris Ireland (University of Utah, Salt Lake City, UT 84112, USA) were selected because their parent organisms are rich sources of secondary metabolites and present a diverse suite of structural classes with a variety of biological activities. They were fractionated in the Chris Ireland’s laboratory by means of the available fractionation protocol that enhances screening by removing salts and other detrimental materials and concentrating metabolites [64]. The HP20 fractions selected are semi-purified, not crude, extracts, of varying polarity, whereas fractions that elute earlier are more water/methanol soluble. Forty HP20 fractions derived from 11 different marine invertebrates were tested at a concentration of 0.5 µg/mL (Table 2). For each fraction, an initial sample size of 15 seedlings was examined. Based on the results, the experiment was repeated two more times for fractions with shoot and/or root parameters that differed from the control samples.

### Statistical analysis

As statistical package, the ‘R version 3.6.0’ [120] was utilized. The least square means of the biological repeats were subjected to a statistical analysis, in which the sample size (*n*) is the total number of rice seedlings analyzed per treatment. To assess the statistical difference between two or more experimental groups, a multiple analysis of variance (ANOVA) (Linear Mixed-Effects model) was used [121, 122], whereas for discrete data, i.e. the number of emerged crown roots, a Generalized Linear Mixed-Effects Model with ‘Poisson’ as the family function was applied [123, 124]. A Tukey correction was always included as post-hoc analysis. The statistical assumptions of a normal distribution (via histogram) and of homogeneity of variances (by analysis of residuals, QQ-plot) were tested and, where needed, log-transformations were applied, when the data did not comply.

## Supplementary information

**Additional file 1: Figure S1.** Workflow overview of the RIVA method. Seeds were sterilized and pregerminated by complete submergence in 2.0 mM CaSO_4_ in the dark. After 3 days of pregermination, selected seeds were transferred to test tubes filled with 1 mL 2.0 mM CaSO_4_ and a compound of interest. After 7 days, rice seedlings were harvested. Shoots and coleoptiles were transferred to plates and scanned. Yield-related parameters were automatically generated from these images via an in-house developed software, designated Plength.

**Additional file 2: Figure S2.** Extra information about the image processing pipeline in Plength. Top panel: Smoothing improving thresholding by homogenizing the colors, removal of the noise, and connection of edges. Middle panel: Segmentation, i.e. thresholding and partitioning of the image into individual plants. Bottom panel: Prior to feature extraction, skeletonization of the contour, conversion to a graph, and pruning. The red and green dots indicate nodes and connecting edges, respectively. Note that the initial graph consists of nine nodes, but the pruned graph of only of seven.

**Additional file 3: Figure S3.** Theoretical example of the analysis of artificial plants with three or four leaves. Detected regions are numbered and framed. Leaves are traced in yellow.

**Additional file 4: Figure S4.** Graphical User Interface of Plength and its interference functionalities. Top panel: GUI with an uploaded image. Information on the file directory and image size are displayed. Bottom panel: Interference functionalities. (A) The four settings that can be changed, i.e. plant type, coleoptile or seedling, scale bar position and length, and minimum detection area. (B) Preprocessing. Before the analysis, the image can be cropped and the detected areas can be checked. (top) cropping tool; (bottom) color thresholding tool. (C) Postprocessing. The display after analysis completion. Detected regions are framed and numbered and the leaves are traced. For each detected area, the shoot, internode and leaf lengths are given.

**Additional file 5: Figure S5.** Validation of the screening method by interfering with auxins. Per treatment, 1 µM of NAA, 2,4-D, IAA, and NPA was added. The shoot parameters are the lengths (in mm) of the total shoot, internode, coleoptile, and leaves 1 and 2. The root parameters are the lengths (in mm) of the seminal and crown roots and the number of emerged crown roots. Different letters indicate statistically significant differences between treatments (see “Methods”). The root data for the 2,4-D treatment are not available, because the root system was unmeasurable. The picture shows the harvested rice seedling treated with 1 µM 2,4-D with the “stumpy” root phenotype. Scale bar, 1 cm.

**Additional file 6: Figure S6.** Validation of the screening method by interfering with cytokinins. Per treatment, 1 µM of kinetin, 2iP, and *trans*-zeatin was added. The shoot parameters are the lengths (in mm) of the total shoot, internode, coleoptile, and leaves 1 and 2. The root parameters are the lengths (in mm) of the seminal and crown roots and the number of emerged crown roots. Different letters indicate statistically significant differences between treatments (see “Methods”).

**Additional file 7: Figure S7.** Validation of the screening method by interfering with brassinosteroids. Per treatment, 1 µM of bikinin and brassinolide was added. The shoot parameters are the lengths (in mm) of the total shoot, internode, coleoptile, and leaves 1 and 2. The root parameters are the lengths (in mm) of the seminal and crown roots and the number of emerged crown roots. Different letters indicate statistically significant differences between treatments (see “Methods”).

**Additional file 8: Figure S8.** Validation of the screening method by interference with the ethylene pathway. Per treatment, 1 µM of ACC and AgNO_3_ was added. The shoot parameters are the lengths (in mm) of the total shoot, internode, coleoptile, and leaves 1 and 2. The root parameters are the lengths (in mm) of the seminal and crown roots and the number of emerged crown roots. Different letters indicate statistically significant differences between treatments (see “Methods”).

**Additional file 9: Figure S9.** Validation of the screening method by means of a concentration range of NaCl in *Oryza sativa* (L.) cv. Chucheongbyeo. Also 1 µM GA_3_ was added in the test tubes. Mock_DMSO corresponds to GA_3_ as this is also dissolved in DMSO, while NaCl is dissolved in sterile dH_2_O. The shoot parameters (in mm) are lengths of total shoot, internode, coleoptile, and leaves 1 and 2. The root parameters are lengths (in mm) of the seminal and crown roots and the number of emerged crown roots. Different letters indicate statistically significant differences between the treatments (see “Methods”).

**Additional file 10: Figure S10.** Validation of the screening method by means of a concentration range of NaCl in *Oryza sativa* (L.) cv. Chilbo. Also 1 µM GA_3_ was added in the test tubes. Mock_DMSO corresponds to GA_3_ as this is also dissolved in DMSO, while NaCl is dissolved in sterile dH_2_O. The shoot parameters (in mm) are lengths of total shoot, internode, coleoptile, and leaves 1 and 2. The root parameters are lengths (in mm) of the seminal and crown roots and the number of emerged crown roots. Different letters indicate statistically significant differences between the treatments (see “Methods”).

**Additional file 11.** Least squares means for each parameter in the NaCl experiment for the different cultivars tested (*Oryza sativa *(L.) cv. (New) Dongjin, cv. Chucheongbyeo and cv. Chilbo, a semi-dwarf variety).

**Additional file 12.** Pairwise comparisons for each parameter in the NaCl experiment for the different cultivars tested (*Oryza sativa *(L.) cv. (New) Dongjin, cv. Chucheongbyeo and cv. Chilbo, a semi-dwarf variety).

**Additional file 13: Figure S11.** Validation of the screening method by means of a concentration range of DCMU in *Oryza sativa* (L.) cv. (New) Dongjin. The shoot parameters (in mm) are lengths of total shoot, internode, coleoptile, and leaves 1 and 2. The root parameters are lengths (in mm) of the seminal and crown roots and the number of emerged crown roots. Different letters indicate statistically significant differences between the treatments (see “Methods”).

**Additional file 14: Figure S12.** Illustration of the use of Plength in another cereal crop, such as wheat.

**Additional file 15.** Python source code used to make Plength.

**Additional file 16.** Plength installation manual.

## Data Availability

All data generated or analyzed during this study are included in this publication and in Additional files 1, 2, 3, 4, 5, 6, 7, 8, 9, 10, 11, 12, 13, 14, 15, and 16.

## Abbreviations

2,4-D: Dichlorophenoxyacetic acid
2iP: 6-(γ,γ-Dimethylallylamino)purine
ABA: Abscisic acid
ACC: 1-Aminocyclopropane-1-carboxylic acid
GA_3_: Gibberellic acid
GC: Genomic selection
GUI: Graphical user interface
HSV: Hue, saturation, value
IAA: Indole acetic acid
MAS: Marker-assisted selection
MICL: Marine natural product library
NAA: Naphthalene acetic acid
NPA: *N*-1-Naphthylphthalamic acid
RIVA: Rice in vitro assay
SAP: Sand + absorbent polymer
