# Supplementary material for: Development of a novel and rapid phenotype-based screening method to assess rice seedling growth
Source: Plant Methods. 2020 Oct 15;16:139. doi: 10.1186/s13007-020-00682-6 (PMC7560306; doi:10.1186/s13007-020-00682-6)
Supplement: Supplementary file 1 — Additional file 1: Figure S1. Workflow overview of the RIVA method. Seeds were sterilized and pregerminated by complete submergence in 2.0 mM CaSO4 in the dark. After 3 days of pregermination, selected seeds were transferred to test tubes filled with 1 mL 2.0 mM CaSO4 and a compound of interest. After 7 days, rice seedlings were harvested. Shoots and coleoptiles were transferred to plates and scanned. Yield-related parameters were automatically generated from these images via an in-house developed software, designated Plength. [file 13007_2020_682_MOESM1_ESM.pdf]

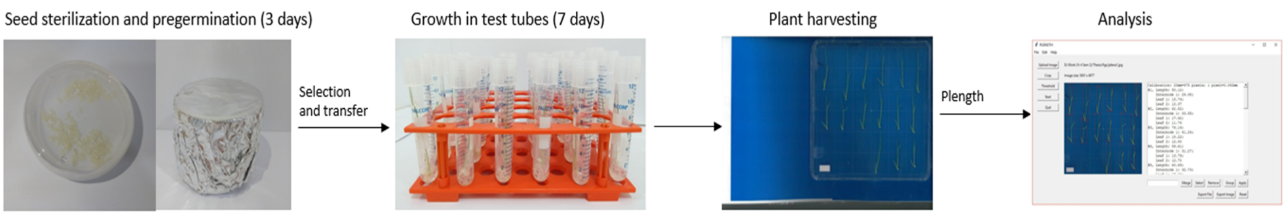
Seed sterilization and pregermination (3 days)
Growth in test tubes (7 days)
Plant harvesting
Analysis
Selection
and transfer
Plength
