# Supplementary material for: Development of a novel and rapid phenotype-based screening method to assess rice seedling growth
Source: Plant Methods. 2020 Oct 15;16:139. doi: 10.1186/s13007-020-00682-6 (PMC7560306; doi:10.1186/s13007-020-00682-6)
Supplement: Supplementary file 2 — Additional file 2: Figure S2. Extra information about the image processing pipeline in Plength. Top panel: Smoothing improving thresholding by homogenizing the colors, removal of the noise, and connection of edges. Middle panel: Segmentation, i.e. thresholding and partitioning of the image into individual plants. Bottom panel: Prior to feature extraction, skeletonization of the contour, conversion to a graph, and pruning. The red and green dots indicate nodes and connecting edges, respectively. Note that the initial graph consists of nine nodes, but the pruned graph of only of seven. [file 13007_2020_682_MOESM2_ESM.pdf]

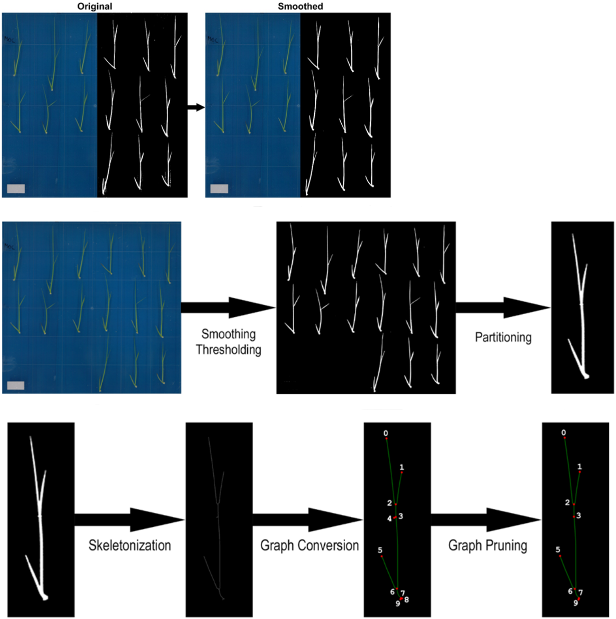
Original
Smoothed
Smoothing
Thresholding
Partitioning
Skeletonization
Graph Conversion
Graph Pruning
0
1
2
4
3
5
6
7
9
8
0
1
2
3
5
6
7
9
