# Supplementary material for: Development of a novel and rapid phenotype-based screening method to assess rice seedling growth
Source: Plant Methods. 2020 Oct 15;16:139. doi: 10.1186/s13007-020-00682-6 (PMC7560306; doi:10.1186/s13007-020-00682-6)
Supplement: Supplementary file 3 — Additional file 3: Figure S3. Theoretical example of the analysis of artificial plants with three or four leaves. Detected regions are numbered and framed. Leaves are traced in yellow. [file 13007_2020_682_MOESM3_ESM.pdf]

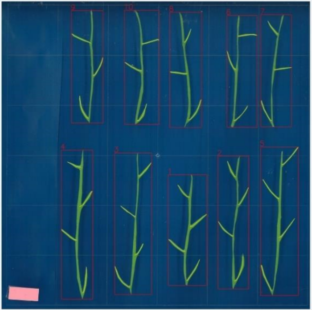

```
Calibration: 20mm=500 pixels; 1 pixel=0.040mm
#1, Length: 74.84;
    Internode 1: 21.99;
    Leaf 1: 19.78;
    Internode 2: 17.47;
    Leaf 2: 13.98;
    Internode 3: 19.36;
    Leaf 3: 14.09;
    Leaf 4: 11.94
#2, Length: 89.63;
    Internode 1: 34.01;
    Leaf 1: 23.25;
    Internode 2: 21.50;
    Leaf 2: 14.61;
    Internode 3: 18.63;
    Leaf 3: 14.19;
    Leaf 4: 12.91
#3, Length: 95.15;
    Internode 1: 25.67;
    Leaf 1: 22.91;
```
