# Supplementary figures and images for: Development of a novel and rapid phenotype-based screening method to assess rice seedling growth
Source: Plant Methods. 2020 Oct 15;16:139. doi: 10.1186/s13007-020-00682-6 (PMC7560306; doi:10.1186/s13007-020-00682-6)

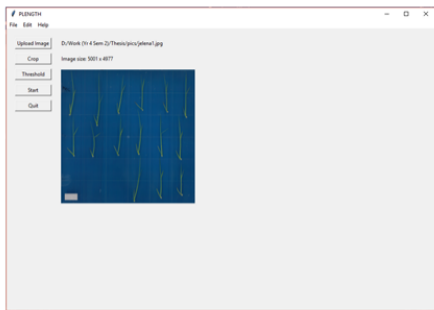

A

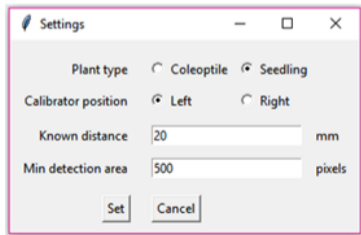

B

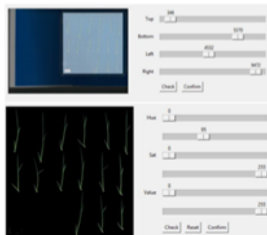

C

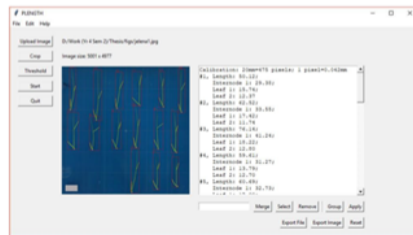

Supplement: Supplementary file 4 — Additional file 4: Figure S4. Graphical User Interface of Plength and its interference functionalities. Top panel: GUI with an uploaded image. Information on the file directory and image size are displayed. Bottom panel: Interference functionalities. (A) The four settings that can be changed, i.e. plant type, coleoptile or seedling, scale bar position and length, and minimum detection area. (B) Preprocessing. Before the analysis, the image can be cropped and the detected areas can be checked. (top) cropping tool; (bottom) color thresholding tool. (C) Postprocessing. The display after analysis completion. Detected regions are framed and numbered and the leaves are traced. For each detected area, the shoot, internode and leaf lengths are given. [file 13007_2020_682_MOESM4_ESM.pdf]

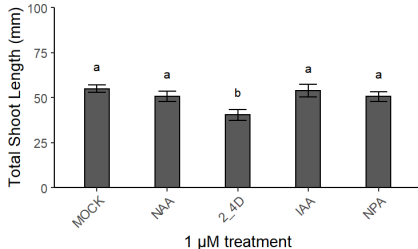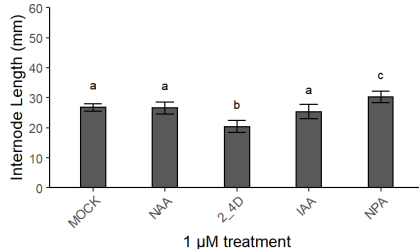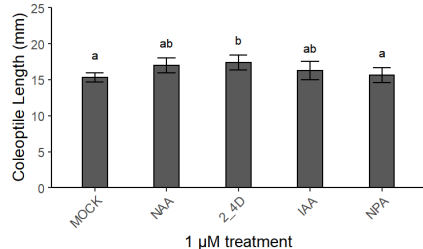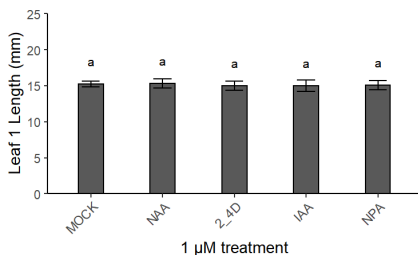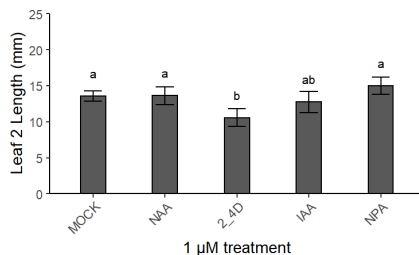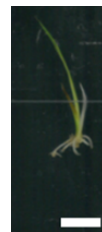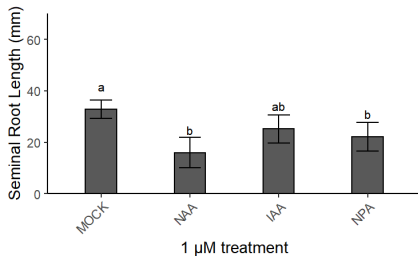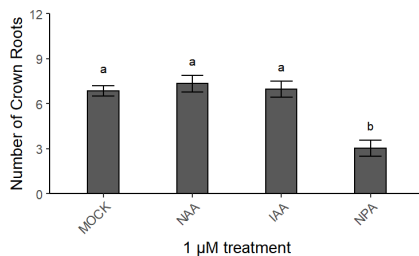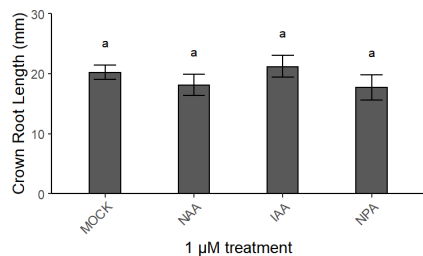

Supplement: Supplementary file 5 — Additional file 5: Figure S5. Validation of the screening method by interfering with auxins. Per treatment, 1 µM of NAA, 2,4-D, IAA, and NPA was added. The shoot parameters are the lengths (in mm) of the total shoot, internode, coleoptile, and leaves 1 and 2. The root parameters are the lengths (in mm) of the seminal and crown roots and the number of emerged crown roots. Different letters indicate statistically significant differences between treatments (see “Methods”). The root data for the 2,4-D treatment are not available, because the root system was unmeasurable. The picture shows the harvested rice seedling treated with 1 µM 2,4-D with the “stumpy” root phenotype. Scale bar, 1 cm. [file 13007_2020_682_MOESM5_ESM.pdf]

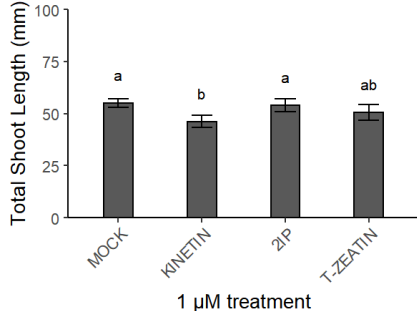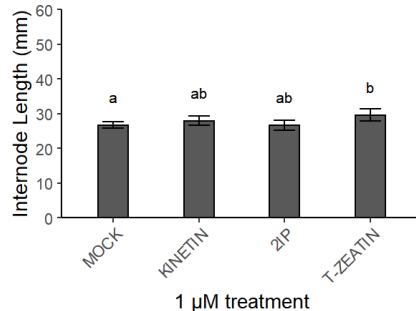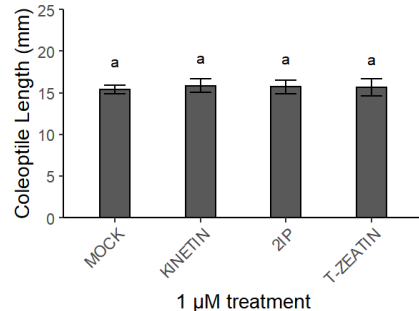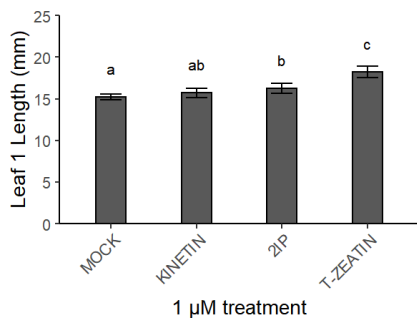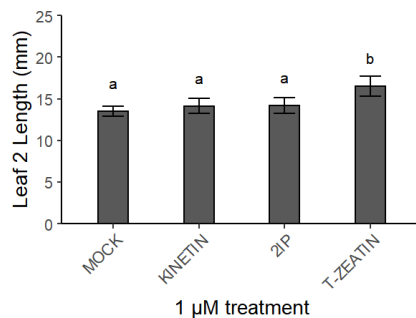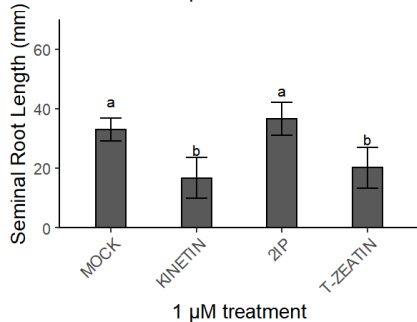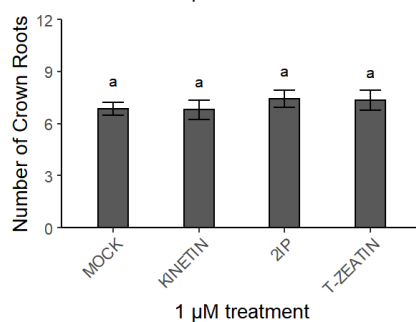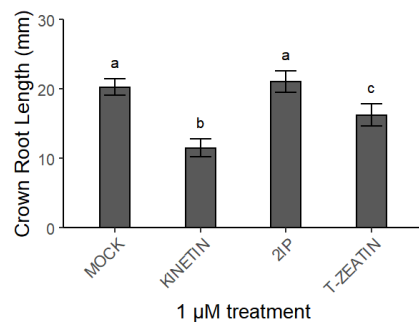

Supplement: Supplementary file 6 — Additional file 6: Figure S6. Validation of the screening method by interfering with cytokinins. Per treatment, 1 µM of kinetin, 2iP, and trans-zeatin was added. The shoot parameters are the lengths (in mm) of the total shoot, internode, coleoptile, and leaves 1 and 2. The root parameters are the lengths (in mm) of the seminal and crown roots and the number of emerged crown roots. Different letters indicate statistically significant differences between treatments (see “Methods”). [file 13007_2020_682_MOESM6_ESM.pdf]

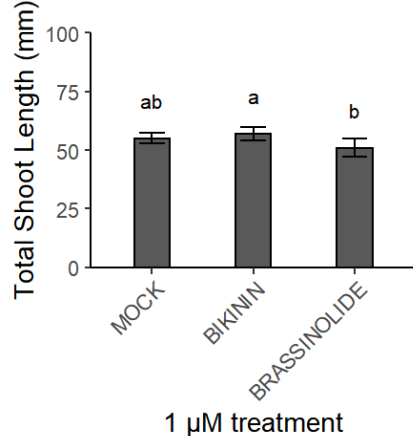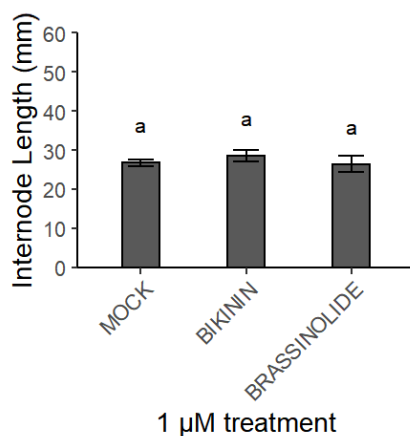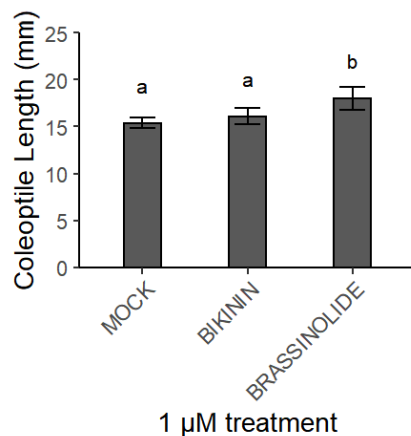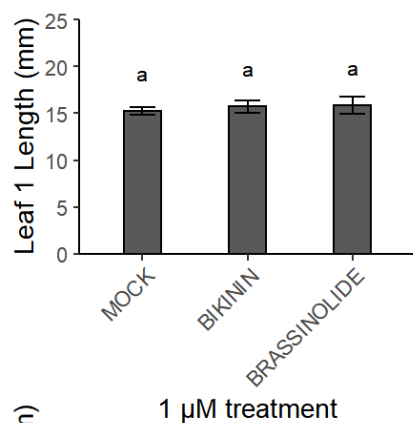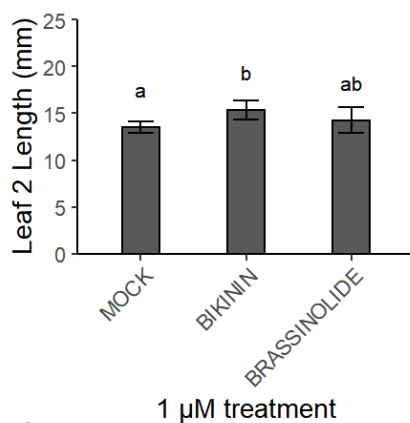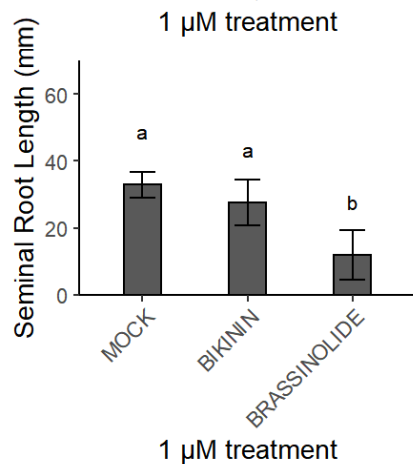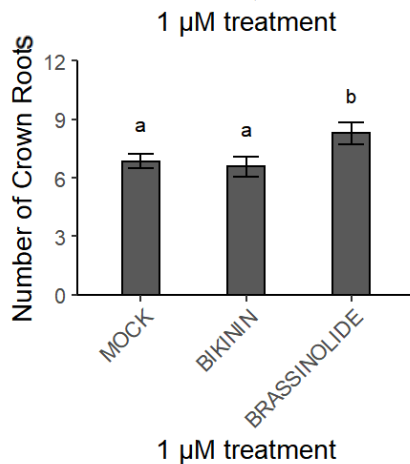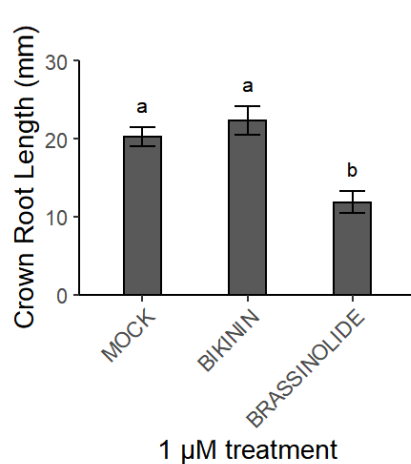

Supplement: Supplementary file 7 — Additional file 7: Figure S7. Validation of the screening method by interfering with brassinosteroids. Per treatment, 1 µM of bikinin and brassinolide was added. The shoot parameters are the lengths (in mm) of the total shoot, internode, coleoptile, and leaves 1 and 2. The root parameters are the lengths (in mm) of the seminal and crown roots and the number of emerged crown roots. Different letters indicate statistically significant differences between treatments (see “Methods”). [file 13007_2020_682_MOESM7_ESM.pdf]

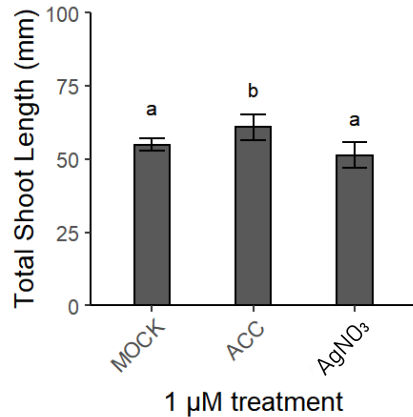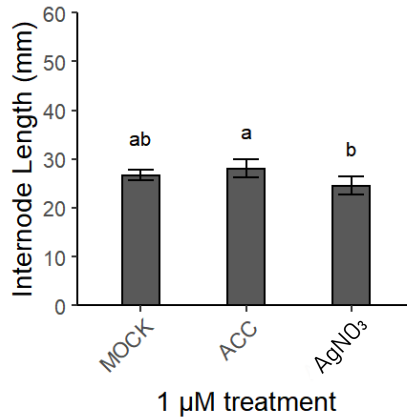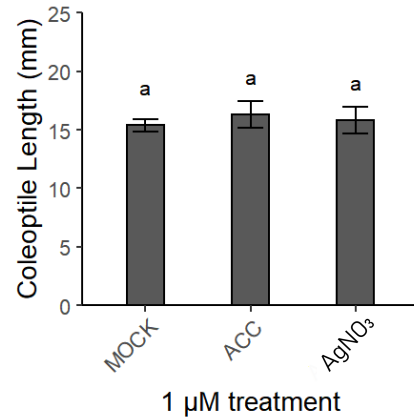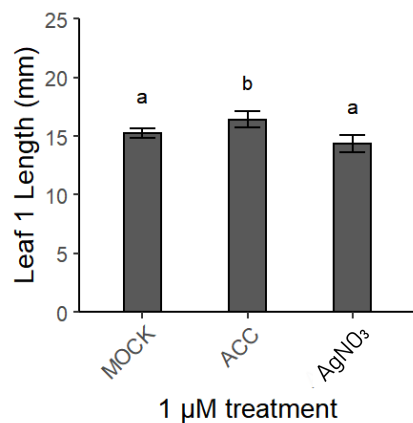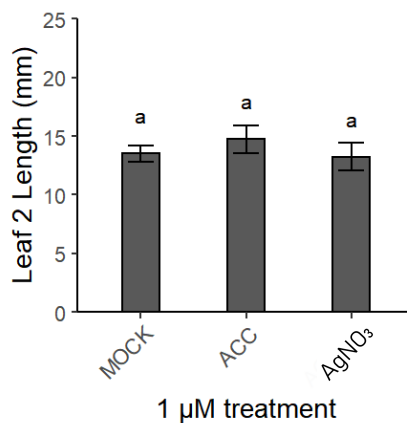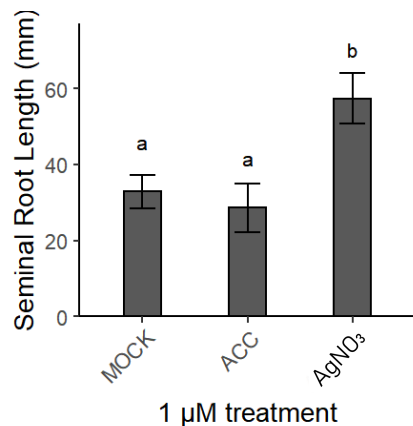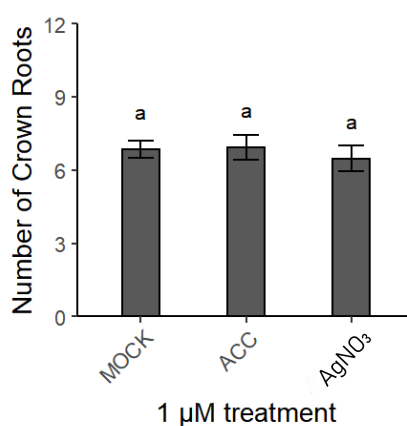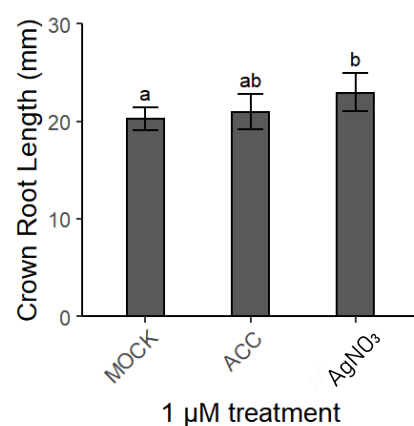

Supplement: Supplementary file 8 — Additional file 8: Figure S8. Validation of the screening method by interference with the ethylene pathway. Per treatment, 1 µM of ACC and AgNO3 was added. The shoot parameters are the lengths (in mm) of the total shoot, internode, coleoptile, and leaves 1 and 2. The root parameters are the lengths (in mm) of the seminal and crown roots and the number of emerged crown roots. Different letters indicate statistically significant differences between treatments (see “Methods”). [file 13007_2020_682_MOESM8_ESM.pdf]

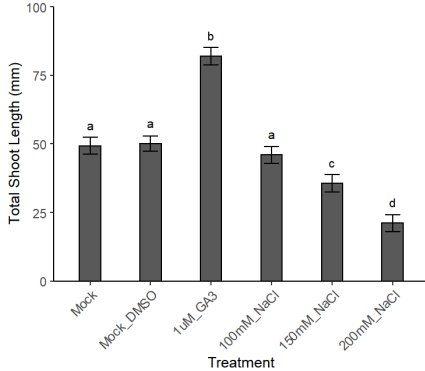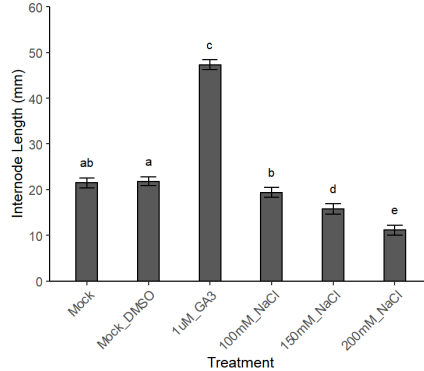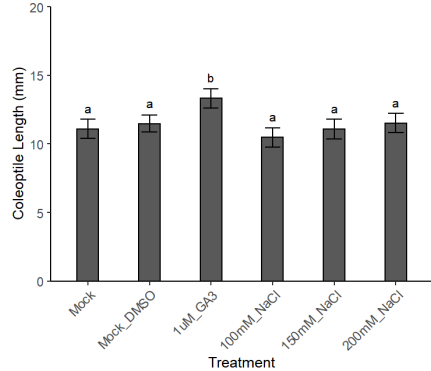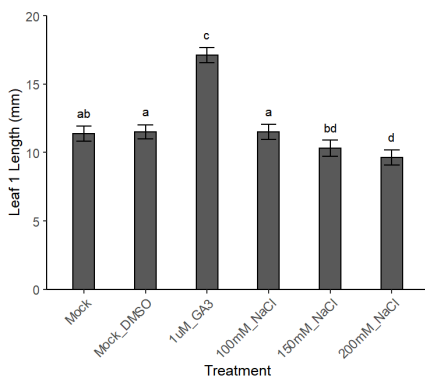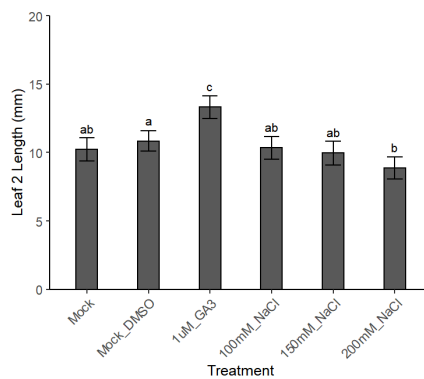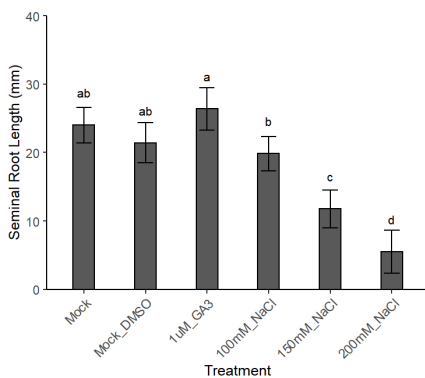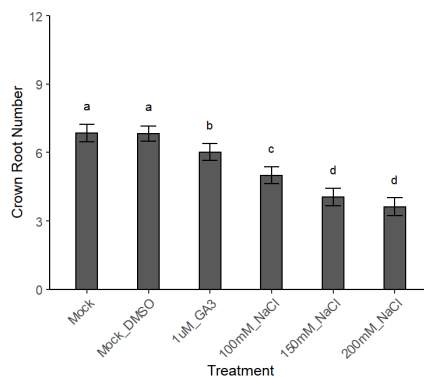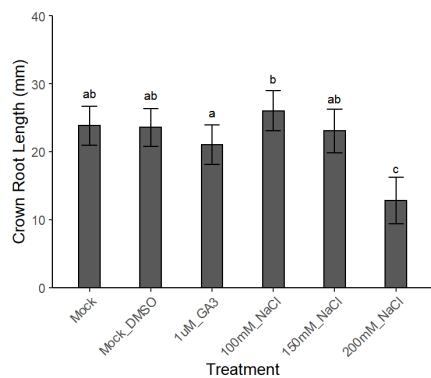

Supplement: Supplementary file 9 — Additional file 9: Figure S9. Validation of the screening method by means of a concentration range of NaCl in Oryza sativa (L.) cv. Chucheongbyeo. Also 1 µM GA3 was added in the test tubes. Mock_DMSO corresponds to GA3 as this is also dissolved in DMSO, while NaCl is dissolved in sterile dH2O. The shoot parameters (in mm) are lengths of total shoot, internode, coleoptile, and leaves 1 and 2. The root parameters are lengths (in mm) of the seminal and crown roots and the number of emerged crown roots. Different letters indicate statistically significant differences between the treatments (see “Methods”). [file 13007_2020_682_MOESM9_ESM.pdf]

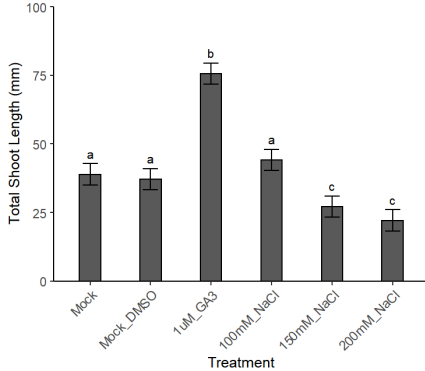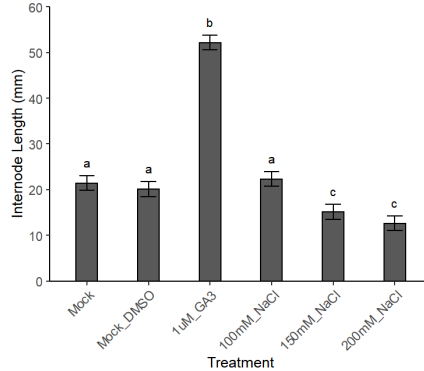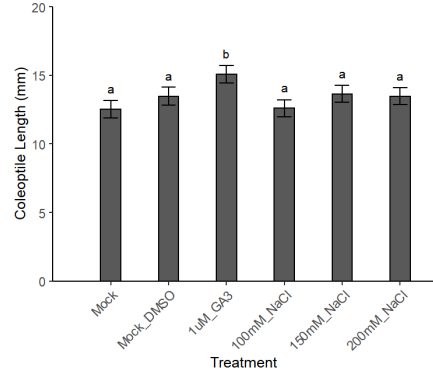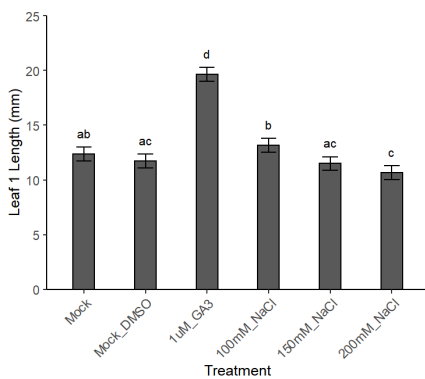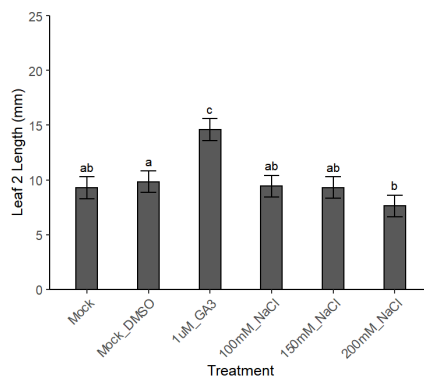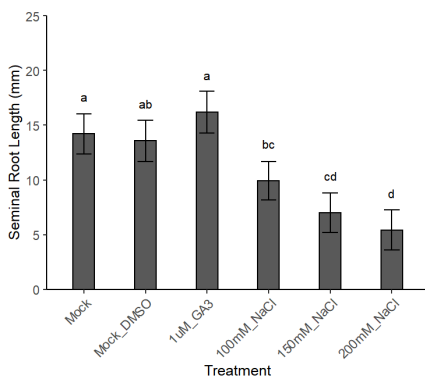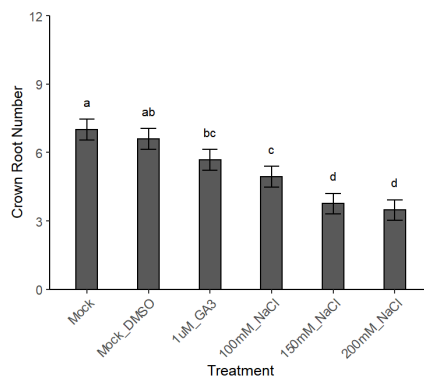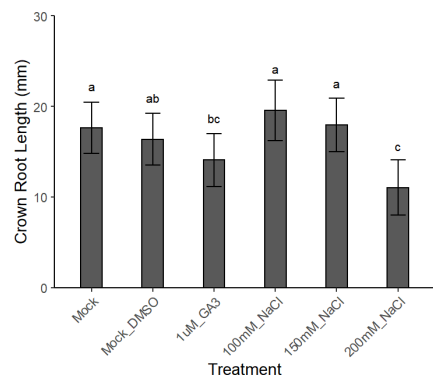

Supplement: Supplementary file 10 — Additional file 10: Figure S10. Validation of the screening method by means of a concentration range of NaCl in Oryza sativa (L.) cv. Chilbo. Also 1 µM GA3 was added in the test tubes. Mock_DMSO corresponds to GA3 as this is also dissolved in DMSO, while NaCl is dissolved in sterile dH2O. The shoot parameters (in mm) are lengths of total shoot, internode, coleoptile, and leaves 1 and 2. The root parameters are lengths (in mm) of the seminal and crown roots and the number of emerged crown roots. Different letters indicate statistically significant differences between the treatments (see “Methods”). [file 13007_2020_682_MOESM10_ESM.pdf]

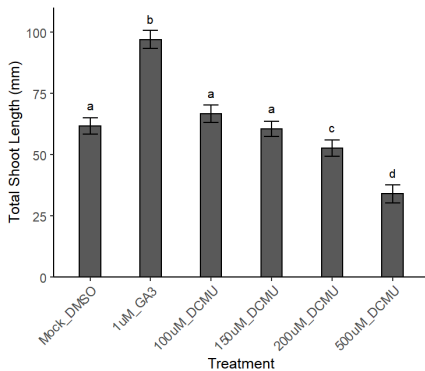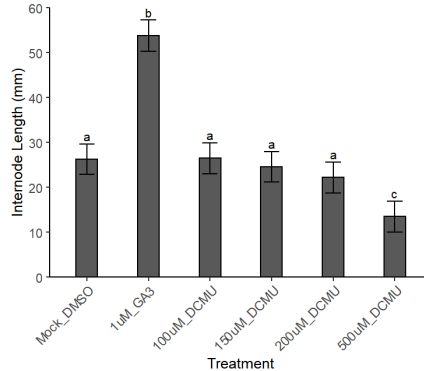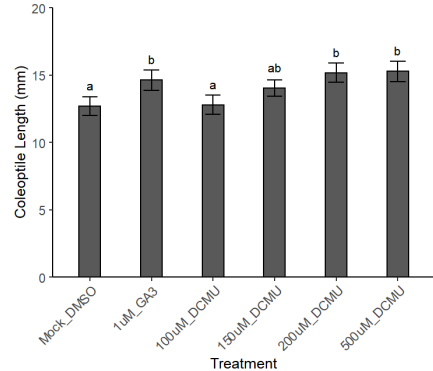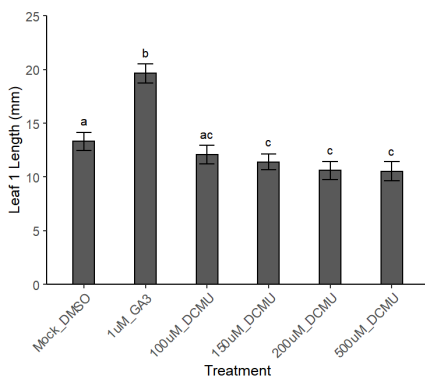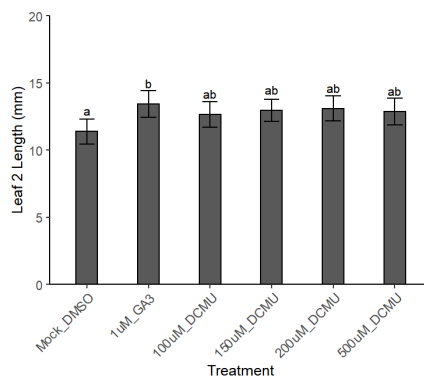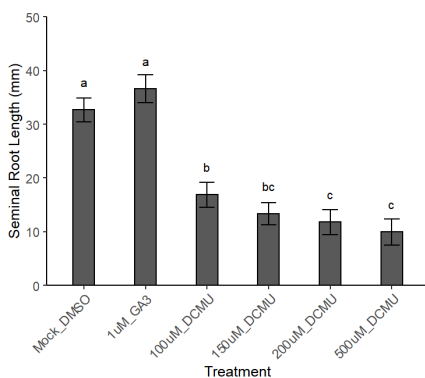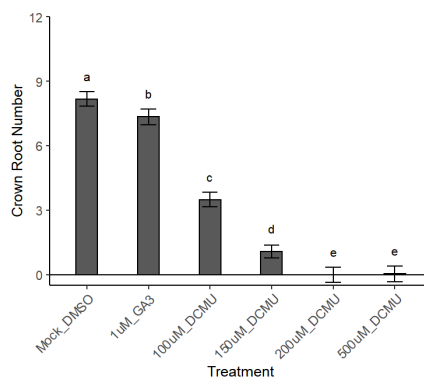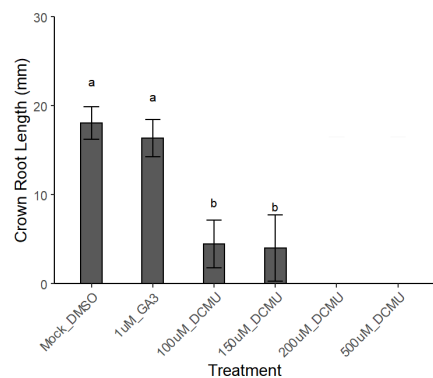

Supplement: Supplementary file 13 — Additional file 13: Figure S11. Validation of the screening method by means of a concentration range of DCMU in Oryza sativa (L.) cv. (New) Dongjin. The shoot parameters (in mm) are lengths of total shoot, internode, coleoptile, and leaves 1 and 2. The root parameters are lengths (in mm) of the seminal and crown roots and the number of emerged crown roots. Different letters indicate statistically significant differences between the treatments (see “Methods”). [file 13007_2020_682_MOESM13_ESM.pdf]
