# Supplementary material for: Development of a novel and rapid phenotype-based screening method to assess rice seedling growth
Source: Plant Methods. 2020 Oct 15;16:139. doi: 10.1186/s13007-020-00682-6 (PMC7560306; doi:10.1186/s13007-020-00682-6)
Supplement: Supplementary file 11 — Additional file 11. Least squares means for each parameter in the NaCl experiment for the different cultivars tested (Oryza sativa (L.) cv. (New) Dongjin, cv. Chucheongbyeo and cv. Chilbo, a semi-dwarf variety). [file 13007_2020_682_MOESM11_ESM.docx]

**Additional file 11:** Least-squares means or emmean for each parameter in the NaCl experiment for the different cultivars tested (*Oryza sativa* (L.) cv. (New) Dongjin, cv. Chucheongbyeo and cv. Chilbo, a semi-dwarf variety).

Total Shoot Lengh (mm)

| Treatment | Cultivar | emmean | SE | df | lower.CL | upper.CL | .group |
| --- | --- | --- | --- | --- | --- | --- | --- |
| Mock | New_Dongjin | 61.24617 | 3.466045 | 34.23906 | 54.20413 | 68.28821 | abc |
| Mock_DMSO | New_Dongjin | 63.41908 | 3.430016 | 32.84082 | 56.43938 | 70.39879 | ab |
| 1uM_GA3 | New_Dongjin | 100.9239 | 3.430016 | 32.84082 | 93.9442 | 107.9036 | d |
| 100mM_NaCl | New_Dongjin | 56.1689 | 3.408604 | 32.02992 | 49.22605 | 63.11174 | ace |
| 150mM_NaCl | New_Dongjin | 39.24114 | 4.10932 | 28.49796 | 30.8302 | 47.65208 | efghi |
| 200mM_NaCl | New_Dongjin | 20.42715 | 3.398603 | 31.6557 | 13.50147 | 27.35283 | f |
| Mock | Chucheongbyeo | 49.24584 | 3.431226 | 32.87105 | 42.26392 | 56.22777 | aceg |
| Mock_DMSO | Chucheongbyeo | 49.93476 | 3.346565 | 29.61673 | 43.09645 | 56.77306 | aceg |
| 1uM_GA3 | Chucheongbyeo | 81.89622 | 3.432667 | 32.90428 | 74.91163 | 88.8808 | j |
| 100mM_NaCl | Chucheongbyeo | 46.04772 | 3.408604 | 32.02992 | 39.10487 | 52.99056 | aceg |
| 150mM_NaCl | Chucheongbyeo | 36.80864 | 4.06757 | 27.48084 | 28.46951 | 45.14778 | efghi |
| 200mM_NaCl | Chucheongbyeo | 20.99026 | 3.409795 | 32.05829 | 14.04523 | 27.93529 | fh |
| Mock | Chilbo | 38.88152 | 3.418672 | 32.41477 | 31.92141 | 45.84164 | eghi |
| Mock_DMSO | Chilbo | 37.10612 | 3.419873 | 32.44411 | 30.14381 | 44.06844 | fghi |
| 1uM_GA3 | Chilbo | 75.4773 | 3.419873 | 32.44413 | 68.51499 | 82.43962 | bj |
| 100mM_NaCl | Chilbo | 44.20152 | 3.408604 | 32.02993 | 37.25868 | 51.14437 | cegi |
| 150mM_NaCl | Chilbo | 27.25677 | 3.398604 | 31.65575 | 20.33109 | 34.18246 | fhi |
| 200mM_NaCl | Chilbo | 22.18438 | 3.408604 | 32.02998 | 15.24153 | 29.12722 | fh |

Internode Length (mm)

| Treatment | Cultivar | emmean | SE | df | lower.CL | upper.CL | .group |
| --- | --- | --- | --- | --- | --- | --- | --- |
| Mock | New_Dongjin | 25.86170105 | 1.642584294 | 33.52272094 | 22.52181666 | 29.20158544 | ab |
| Mock_DMSO | New_Dongjin | 26.8586973 | 1.630739425 | 32.56781358 | 23.5392596 | 30.178135 | a |
| 1uM_GA3 | New_Dongjin | 54.71409378 | 1.630739425 | 32.56781358 | 51.39465609 | 58.03353148 | c |
| 100mM_NaCl | New_Dongjin | 21.83356991 | 1.623717714 | 32.01124802 | 18.52621073 | 25.14092908 | abd |
| 150mM_NaCl | New_Dongjin | 15.87300822 | 1.974819694 | 29.70283163 | 11.83819564 | 19.9078208 | def |
| 200mM_NaCl | New_Dongjin | 11.2002038 | 1.620444171 | 31.75387751 | 7.898463373 | 14.50194423 | e |
| Mock | Chucheongbyeo | 21.45949448 | 1.631168978 | 32.59460968 | 18.13928753 | 24.77970143 | abd |
| Mock_DMSO | Chucheongbyeo | 21.71409271 | 1.603764965 | 30.39969203 | 18.44057256 | 24.98761286 | abd |
| 1uM_GA3 | Chucheongbyeo | 47.28753445 | 1.631688577 | 32.62550021 | 43.96639087 | 50.60867804 | c |
| 100mM_NaCl | Chucheongbyeo | 19.38227277 | 1.623717714 | 32.01124802 | 16.0749136 | 22.68963195 | abdef |
| 150mM_NaCl | Chucheongbyeo | 16.15599897 | 1.960900654 | 28.93541566 | 12.14511797 | 20.16687997 | bdef |
| 200mM_NaCl | Chucheongbyeo | 11.0780024 | 1.624142878 | 32.03693301 | 7.769881189 | 14.38612362 | e |
| Mock | Chilbo | 21.44811905 | 1.627006345 | 32.27359934 | 18.13511709 | 24.761121 | abd |
| Mock_DMSO | Chilbo | 20.05149004 | 1.627433814 | 32.29985124 | 16.73772235 | 23.36525773 | abdf |
| 1uM_GA3 | Chilbo | 52.03571682 | 1.627433833 | 32.2998587 | 48.72194912 | 55.34948452 | c |
| 100mM_NaCl | Chilbo | 22.29560388 | 1.62371772 | 32.01125013 | 18.98824471 | 25.60296306 | abd |
| 150mM_NaCl | Chilbo | 15.17293693 | 1.620444293 | 31.7538997 | 11.87119634 | 18.47467751 | def |
| 200mM_NaCl | Chilbo | 12.66550809 | 1.623717841 | 32.01127272 | 9.35814876 | 15.97286743 | ef |

Coleoptile Length (mm)

| Treatment | Cultivar | emmean | SE | df | lower.CL | upper.CL | .group |
| --- | --- | --- | --- | --- | --- | --- | --- |
| Mock | New_Dongjin | 13.26401616 | 0.556260841 | 41.09616261 | 12.14070442 | 14.38732789 | abc |
| Mock_DMSO | New_Dongjin | 12.86486239 | 0.546487721 | 38.29362664 | 11.75883434 | 13.97089043 | abc |
| 1uM_GA3 | New_Dongjin | 13.69286022 | 0.546487721 | 38.29362664 | 12.58683218 | 14.79888827 | ab |
| 100mM_NaCl | New_Dongjin | 12.74450799 | 0.540646736 | 36.68689303 | 11.64873798 | 13.840278 | abc |
| 150mM_NaCl | New_Dongjin | 13.36145486 | 0.631412233 | 28.69360506 | 12.06947261 | 14.65343712 | abc |
| 200mM_NaCl | New_Dongjin | 13.70863091 | 0.537906136 | 35.94851682 | 12.61765245 | 14.79960936 | ab |
| Mock | Chucheongbyeo | 11.20536671 | 0.546745866 | 38.30815339 | 10.09882988 | 12.31190354 | ac |
| Mock_DMSO | Chucheongbyeo | 11.54019105 | 0.522818604 | 31.54859479 | 10.47464648 | 12.60573562 | ac |
| 1uM_GA3 | Chucheongbyeo | 13.42174236 | 0.547040282 | 38.31836761 | 12.31461928 | 14.52886543 | ab |
| 100mM_NaCl | Chucheongbyeo | 10.5688492 | 0.540646736 | 36.68689303 | 9.47307919 | 11.66461921 | c |
| 150mM_NaCl | Chucheongbyeo | 11.17205189 | 0.620490296 | 27.13810856 | 9.899214067 | 12.44488972 | ac |
| 200mM_NaCl | Chucheongbyeo | 11.59112243 | 0.54089517 | 36.6979851 | 10.49486018 | 12.68738468 | ac |
| Mock | Chilbo | 12.53385588 | 0.470258878 | 40.1550868 | 11.58354156 | 13.48417019 | ac |
| Mock_DMSO | Chilbo | 13.53150775 | 0.473673122 | 40.97891683 | 12.57489053 | 14.48812498 | ab |
| 1uM_GA3 | Chilbo | 15.1318247 | 0.465038957 | 38.65433481 | 14.19092561 | 16.07272379 | b |
| 100mM_NaCl | Chilbo | 12.6036728 | 0.458718692 | 36.69420463 | 11.67395892 | 13.53338668 | ac |
| 150mM_NaCl | Chilbo | 13.63623136 | 0.456892808 | 36.1094398 | 12.7097073 | 14.56275542 | ab |
| 200mM_NaCl | Chilbo | 13.47504049 | 0.458718875 | 36.69434446 | 12.54532636 | 14.40475462 | ab |

Leaf 1 Length (mm)

| Treatment | Cultivar | emmean | SE | df | lower.CL | upper.CL | .group |
| --- | --- | --- | --- | --- | --- | --- | --- |
| Mock | New_Dongjin | 12.98933173 | 0.629856611 | 36.65102058 | 11.71271071 | 14.26595275 | ab |
| Mock_DMSO | New_Dongjin | 13.8976097 | 0.622617013 | 34.99934582 | 1.26E+01 | 15.16159028 | ac |
| 1uM_GA3 | New_Dongjin | 19.95235342 | 0.622617013 | 34.99934582 | 18.68837284 | 21.216334 | d |
| 100mM_NaCl | New_Dongjin | 12.63906326 | 0.618310801 | 34.04310186 | 11.38256313 | 13.89556339 | abe |
| 150mM_NaCl | New_Dongjin | 10.7350872 | 0.734723633 | 29.66119803 | 9.23E+00 | 12.23631209 | abe |
| 200mM_NaCl | New_Dongjin | 10.35201678 | 0.616298101 | 33.60203517 | 9.099001738 | 11.60503182 | be |
| Mock | Chucheongbyeo | 11.37611575 | 0.622848354 | 35.02909356 | 10.11170391 | 12.64052759 | abe |
| Mock_DMSO | Chucheongbyeo | 11.51746376 | 0.605697587 | 31.14722241 | 1.03E+01 | 12.75255552 | abe |
| 1uM_GA3 | Chucheongbyeo | 17.0539287 | 0.623120292 | 35.06011123 | 15.78900478 | 18.31885262 | cd |
| 100mM_NaCl | Chucheongbyeo | 11.51857396 | 0.618310801 | 34.04310186 | 10.26207383 | 12.77507409 | abe |
| 150mM_NaCl | Chucheongbyeo | 10.58128536 | 0.72634186 | 28.50032968 | 9.09E+00 | 12.06795337 | abe |
| 200mM_NaCl | Chucheongbyeo | 9.614845096 | 0.618537802 | 34.07060809 | 8.357920992 | 10.8717692 | e |
| Mock | Chilbo | 12.35583333 | 0.620340019 | 34.49874917 | 11.09582172 | 13.61584495 | abe |
| Mock_DMSO | Chilbo | 11.73923354 | 0.620569056 | 34.52730967 | 1.05E+01 | 12.99967253 | abe |
| 1uM_GA3 | Chilbo | 19.64420547 | 0.620569056 | 34.52730967 | 18.38376648 | 20.90464446 | d |
| 100mM_NaCl | Chilbo | 13.13314547 | 0.618310801 | 34.04310186 | 1.19E+01 | 14.3896456 | ab |
| 150mM_NaCl | Chilbo | 11.4911758 | 0.616298101 | 33.60203517 | 1.02E+01 | 12.74419084 | abe |
| 200mM_NaCl | Chilbo | 10.686175 | 0.618310801 | 34.04310186 | 9.43E+00 | 11.94267513 | abe |

Leaf 2 Length (mm)

| Treatment | Cultivar | emmean | SE | df | lower.CL | upper.CL | .group |
| --- | --- | --- | --- | --- | --- | --- | --- |
| Mock | New_Dongjin | 11.45905756 | 0.487752856 | 746 | 10.501526 | 12.41658911 | abc |
| Mock_DMSO | New_Dongjin | 11.61568749 | 0.469568088 | 746 | 1.07E+01 | 12.53751964 | ab |
| 1uM_GA3 | New_Dongjin | 14.9454192 | 0.469568088 | 746 | 14.02358705 | 15.86725134 | d |
| 100mM_NaCl | New_Dongjin | 11.46071337 | 0.458516698 | 746 | 10.56057675 | 12.36084999 | abc |
| 150mM_NaCl | New_Dongjin | 9.548968015 | 0.484665709 | 746 | 8.60E+00 | 10.50043904 | acef |
| 200mM_NaCl | New_Dongjin | 9.362595162 | 0.453276497 | 746 | 8.472745838 | 10.25244449 | acef |
| Mock | Chucheongbyeo | 10.21130121 | 0.469611858 | 746 | 9.289383141 | 11.13321929 | ace |
| Mock_DMSO | Chucheongbyeo | 10.77613244 | 0.417705649 | 746 | 9.96E+00 | 11.59615088 | ace |
| 1uM_GA3 | Chucheongbyeo | 13.29489391 | 0.469668004 | 746 | 12.37286561 | 14.21692221 | bd |
| 100mM_NaCl | Chucheongbyeo | 10.33701569 | 0.458516698 | 746 | 9.436879075 | 11.23715231 | ace |
| 150mM_NaCl | Chucheongbyeo | 9.945299609 | 0.467529859 | 746 | 9.03E+00 | 10.86313041 | ace |
| 200mM_NaCl | Chucheongbyeo | 8.857659867 | 0.458559885 | 746 | 7.957438463 | 9.75788127 | ef |
| Mock | Chilbo | 9.279880952 | 0.46392921 | 746 | 8.369118765 | 10.19064314 | cef |
| Mock_DMSO | Chilbo | 9.823710414 | 0.463975576 | 746 | 8.91E+00 | 10.73456363 | acef |
| 1uM_GA3 | Chilbo | 14.60227856 | 0.46397709 | 746 | 13.69142237 | 15.51313474 | d |
| 100mM_NaCl | Chilbo | 9.418507276 | 0.458517185 | 746 | 8.52E+00 | 10.31864485 | acef |
| 150mM_NaCl | Chilbo | 9.311147448 | 0.453277961 | 746 | 8.42E+00 | 10.20099965 | cef |
| 200mM_NaCl | Chilbo | 7.62047703 | 0.458518701 | 746 | 6.72E+00 | 8.520617581 | f |

Seminal Root Length (mm)

| Treatment | Cultivar | emmean | SE | df | lower.CL | upper.CL | .group |
| --- | --- | --- | --- | --- | --- | --- | --- |
| Mock | New_Dongjin | 29.75490138 | 1.284311056 | 869 | 27.23418714 | 32.27561563 | ab |
| Mock_DMSO | New_Dongjin | 30.45702186 | 1.192878411 | 869 | 2.81E+01 | 32.79828147 | ac |
| 1uM_GA3 | New_Dongjin | 36.68465526 | 1.369162258 | 869 | 33.99740377 | 39.37190675 | c |
| 100mM_NaCl | New_Dongjin | 20.36308851 | 1.398305319 | 869 | 17.618638 | 23.10753902 | def |
| 150mM_NaCl | New_Dongjin | 11.61817017 | 1.478192684 | 869 | 8.72E+00 | 14.51941542 | ghi |
| 200mM_NaCl | New_Dongjin | 8.657553955 | 1.382812663 | 869 | 5.943510842 | 11.37159707 | gh |
| Mock | Chucheongbyeo | 24.0121482 | 1.235911925 | 869 | 21.58642681 | 26.43786958 | bdj |
| Mock_DMSO | Chucheongbyeo | 22.38753658 | 1.316347552 | 869 | 1.98E+01 | 24.97112878 | dej |
| 1uM_GA3 | Chucheongbyeo | 27.47778573 | 1.415669164 | 869 | 24.69925524 | 30.25631621 | abj |
| 100mM_NaCl | Chucheongbyeo | 19.85442985 | 1.202841217 | 869 | 17.49361626 | 22.21524343 | def |
| 150mM_NaCl | Chucheongbyeo | 13.12088186 | 1.235305181 | 869 | 1.07E+01 | 15.54541239 | gi |
| 200mM_NaCl | Chucheongbyeo | 6.552771204 | 1.432569086 | 869 | 3.741071279 | 9.364471129 | h |
| Mock | Chilbo | 14.14941926 | 1.260103189 | 869 | 11.67621774 | 16.62262078 | fgi |
| Mock_DMSO | Chilbo | 13.52121162 | 1.298400686 | 869 | 1.10E+01 | 16.06957954 | gi |
| 1uM_GA3 | Chilbo | 16.23249572 | 1.325566543 | 869 | 13.63080943 | 18.83418201 | efi |
| 100mM_NaCl | Chilbo | 9.902636737 | 1.192444738 | 869 | 7.56E+00 | 12.24304518 | gh |
| 150mM_NaCl | Chilbo | 6.955957107 | 1.23679023 | 869 | 4.53E+00 | 9.383402339 | h |
| 200mM_NaCl | Chilbo | 5.370708649 | 1.259708809 | 869 | 2.90E+00 | 7.843136121 | h |

Number of Crown Roots

| Treatment | Cultivar | emmean | SE | df | lower.CL | upper.CL | .group |
| --- | --- | --- | --- | --- | --- | --- | --- |
| Mock | New_Dongjin | 7.814969554 | 0.212885643 | 737 | 7.397035014 | 8.232904095 | ab |
| Mock_DMSO | New_Dongjin | 8.169971782 | 0.204948675 | 737 | 7.77E+00 | 8.572324564 | a |
| 1uM_GA3 | New_Dongjin | 7.00477868 | 0.207494639 | 737 | 6.597427692 | 7.412129667 | bc |
| 100mM_NaCl | New_Dongjin | 6.004445284 | 0.200125147 | 737 | 5.61E+00 | 6.397328573 | cde |
| 150mM_NaCl | New_Dongjin | 5.208153769 | 0.211625254 | 737 | 4.79E+00 | 5.623613929 | df |
| 200mM_NaCl | New_Dongjin | 4.135655524 | 0.197838009 | 737 | 3.747262318 | 4.52404873 | gh |
| Mock | Chucheongbyeo | 6.86298279 | 0.204967926 | 737 | 6.460592215 | 7.265373365 | bc |
| Mock_DMSO | Chucheongbyeo | 6.860605417 | 0.1823162 | 737 | 6.50E+00 | 7.218526395 | bc |
| 1uM_GA3 | Chucheongbyeo | 6.039896474 | 0.204993446 | 737 | 5.64E+00 | 6.44233715 | cde |
| 100mM_NaCl | Chucheongbyeo | 5.004445284 | 0.200125147 | 737 | 4.61E+00 | 5.397328573 | fg |
| 150mM_NaCl | Chucheongbyeo | 4.133479118 | 0.204148259 | 737 | 3.73E+00 | 4.534260532 | gh |
| 200mM_NaCl | Chucheongbyeo | 3.655986781 | 0.215975878 | 737 | 3.23E+00 | 4.079988035 | h |
| Mock | Chilbo | 7 | 0.202487453 | 737 | 6.60E+00 | 7.39752094 | bc |
| Mock_DMSO | Chilbo | 6.60920354 | 0.202508115 | 737 | 6.21E+00 | 7.006765043 | ce |
| 1uM_GA3 | Chilbo | 5.700375171 | 0.204992121 | 737 | 5.30E+00 | 6.102813244 | def |
| 100mM_NaCl | Chilbo | 4.930957138 | 0.200125372 | 737 | 4.54E+00 | 5.323840868 | fg |
| 150mM_NaCl | Chilbo | 3.755052367 | 0.197838768 | 737 | 3.37E+00 | 4.143447063 | h |
| 200mM_NaCl | Chilbo | 3.476190476 | 0.202487453 | 737 | 3.08E+00 | 3.873711416 | h |

Crown Root Length (mm)

| Treatment | Cultivar | emmean | SE | df | lower.CL | upper.CL | .group |
| --- | --- | --- | --- | --- | --- | --- | --- |
| Mock | New_Dongjin | 19.89665063 | 0.790572678 | 3 | 17.38069554 | 22.41260573 | abcd |
| Mock_DMSO | New_Dongjin | 18.0995224 | 0.757839498 | 3 | 15.68773889 | 20.51130591 | abe |
| 1uM_GA3 | New_Dongjin | 16.72651414 | 0.792083183 | 3 | 14.20575194 | 19.24727634 | aef |
| 100mM_NaCl | New_Dongjin | 20.8665395 | 0.796045269 | 3 | 18.33316817 | 23.39991082 | bcdg |
| 150mM_NaCl | New_Dongjin | 20.23133862 | 0.82868286 | 3 | 17.59409991 | 22.86857732 | bcdg |
| 200mM_NaCl | New_Dongjin | 8.523277958 | 0.847952524 | 3 | 5.82471458 | 11.22184134 | h |
| Mock | Chucheongbyeo | 23.61766311 | 0.797176063 | 3 | 21.0806931 | 26.15463313 | gi |
| Mock_DMSO | Chucheongbyeo | 23.10737252 | 0.742278255 | 3 | 20.74511183 | 25.4696332 | cgi |
| 1uM_GA3 | Chucheongbyeo | 20.65268983 | 0.800691285 | 3 | 18.10453281 | 23.20084685 | bcdg |
| 100mM_NaCl | Chucheongbyeo | 26.0376727 | 0.817163024 | 3 | 23.43709525 | 28.63825014 | i |
| 150mM_NaCl | Chucheongbyeo | 22.44897498 | 0.862648768 | 3 | 19.70364159 | 25.19430836 | cdgi |
| 200mM_NaCl | Chucheongbyeo | 12.35632399 | 0.935980374 | 3 | 9.377616705 | 15.33503127 | hj |
| Mock | Chilbo | 17.76915832 | 0.724440665 | 3 | 15.4636648 | 20.07465184 | abe |
| Mock_DMSO | Chilbo | 16.51724001 | 0.73827743 | 3 | 14.16771173 | 18.86676829 | ef |
| 1uM_GA3 | Chilbo | 14.21976936 | 0.759817653 | 3 | 11.80169048 | 16.63784824 | fj |
| 100mM_NaCl | Chilbo | 19.36715184 | 0.887811888 | 3 | 16.54173818 | 22.19256551 | abde |
| 150mM_NaCl | Chilbo | 18.12466381 | 0.785505194 | 3 | 15.6248357 | 20.62449191 | abe |
| 200mM_NaCl | Chilbo | 11.29002495 | 0.840901199 | 3 | 8.613902036 | 13.96614786 | hj |
