# Supplementary material for: Development of a novel and rapid phenotype-based screening method to assess rice seedling growth
Source: Plant Methods. 2020 Oct 15;16:139. doi: 10.1186/s13007-020-00682-6 (PMC7560306; doi:10.1186/s13007-020-00682-6)
Supplement: Supplementary file 12 — Additional file 12. Pairwise comparisons for each parameter in the NaCl experiment for the different cultivars tested (Oryza sativa (L.) cv. (New) Dongjin, cv. Chucheongbyeo and cv. Chilbo, a semi-dwarf variety). [file 13007_2020_682_MOESM12_ESM.docx]

**Additional file 12:** Pairwise comparisons for each parameter in the NaCl experiment for the different cultivars tested (*Oryza sativa* (L.) cv. (New) Dongjin, cv. Chucheongbyeo and cv. Chilbo, a semi-dwarf variety).

Total Shoot Lengh (mm)

| contrast | estimate | SE | df | t.ratio | p.value |
| --- | --- | --- | --- | --- | --- |
| Mock,New_Dongjin - Mock_DMSO,New_Dongjin | -2.172913328 | 4.876305744 | 33.53574515 | -0.445606457 | 0.999999999 |
| Mock,New_Dongjin - 1uM_GA3,New_Dongjin | -39.67773828 | 4.876305744 | 33.53574515 | -8.136843825 | 2.64E-07 |
| Mock,New_Dongjin - 100mM_NaCl,New_Dongjin | 5.077274144 | 4.86127547 | 33.12545706 | 1.044432511 | 0.999733502 |
| Mock,New_Dongjin - 150mM_NaCl,New_Dongjin | 22.00502637 | 5.376481037 | 30.71480427 | 4.092830648 | 0.024580714 |
| Mock,New_Dongjin - 200mM_NaCl,New_Dongjin | 40.81901894 | 4.854262608 | 32.93469304 | 8.408902079 | 1.44E-07 |
| Mock,New_Dongjin - Mock,Chucheongbyeo | 12.00032672 | 4.877158572 | 33.55104229 | 2.460516004 | 0.571862849 |
| Mock,New_Dongjin - Mock_DMSO,Chucheongbyeo | 11.31141416 | 4.817964252 | 31.88429113 | 2.347758009 | 0.64667124 |
| Mock,New_Dongjin - 1uM_GA3,Chucheongbyeo | -20.65004589 | 4.878192136 | 33.56891211 | -4.233135005 | 0.015684866 |
| Mock,New_Dongjin - 100mM_NaCl,Chucheongbyeo | 15.19845514 | 4.86127547 | 33.12545706 | 3.126433636 | 0.202649726 |
| Mock,New_Dongjin - 150mM_NaCl,Chucheongbyeo | 24.4375274 | 5.344618646 | 30.07553878 | 4.572361289 | 0.007516702 |
| Mock,New_Dongjin - 200mM_NaCl,Chucheongbyeo | 40.25590842 | 4.862092966 | 33.13865689 | 8.279543131 | 1.95E-07 |
| Mock,New_Dongjin - Mock,Chilbo | 22.36464655 | 4.868344942 | 33.32102469 | 4.59389111 | 0.006071117 |
| Mock,New_Dongjin - Mock_DMSO,Chilbo | 24.14004776 | 4.869188041 | 33.33574319 | 4.95771524 | 0.002231111 |
| Mock,New_Dongjin - 1uM_GA3,Chilbo | -14.2311308 | 4.869170553 | 33.33469721 | -2.922701236 | 0.292840362 |
| Mock,New_Dongjin - 100mM_NaCl,Chilbo | 17.04464882 | 4.861291092 | 33.12641698 | 3.506197942 | 0.092408508 |
| Mock,New_Dongjin - 150mM_NaCl,Chilbo | 33.98939764 | 4.8542796 | 32.93573215 | 7.001944767 | 6.97E-06 |
| Mock,New_Dongjin - 200mM_NaCl,Chilbo | 39.06179214 | 4.861274817 | 33.12541408 | 8.035298067 | 3.80E-07 |
| Mock_DMSO,New_Dongjin - 1uM_GA3,New_Dongjin | -37.50482495 | 4.850764291 | 32.84017365 | -7.731735187 | 9.27E-07 |
| Mock_DMSO,New_Dongjin - 100mM_NaCl,New_Dongjin | 7.250187472 | 4.835653677 | 32.4339099 | 1.499319008 | 0.984475001 |
| Mock_DMSO,New_Dongjin - 150mM_NaCl,New_Dongjin | 24.1779397 | 5.35324739 | 30.18721061 | 4.5164996 | 0.008632206 |
| Mock_DMSO,New_Dongjin - 200mM_NaCl,New_Dongjin | 42.99193227 | 4.828604366 | 32.24509691 | 8.903593877 | 4.66E-08 |
| Mock_DMSO,New_Dongjin - Mock,Chucheongbyeo | 14.17324005 | 4.851621377 | 32.85538173 | 2.921340919 | 0.294170105 |
| Mock_DMSO,New_Dongjin - Mock_DMSO,Chucheongbyeo | 13.48432749 | 4.79211276 | 31.20610762 | 2.813858555 | 0.353211712 |
| Mock_DMSO,New_Dongjin - 1uM_GA3,Chucheongbyeo | -18.47713256 | 4.852657821 | 32.8730374 | -3.807631455 | 0.046341816 |
| Mock_DMSO,New_Dongjin - 100mM_NaCl,Chucheongbyeo | 17.37136847 | 4.835653677 | 32.4339099 | 3.592351652 | 0.077071091 |
| Mock_DMSO,New_Dongjin - 150mM_NaCl,Chucheongbyeo | 26.61044073 | 5.321248408 | 29.5527335 | 5.000789043 | 0.002534118 |
| Mock_DMSO,New_Dongjin - 200mM_NaCl,Chucheongbyeo | 42.42882175 | 4.836477841 | 32.44718422 | 8.772669522 | 6.21E-08 |
| Mock_DMSO,New_Dongjin - Mock,Chilbo | 24.53755988 | 4.842759923 | 32.62747011 | 5.066854494 | 0.001721026 |
| Mock_DMSO,New_Dongjin - Mock_DMSO,Chilbo | 26.31296109 | 4.843607537 | 32.64212409 | 5.432513036 | 0.000618142 |
| Mock_DMSO,New_Dongjin - 1uM_GA3,Chilbo | -12.05821747 | 4.843592239 | 32.6412128 | -2.489519529 | 0.55288928 |
| Mock_DMSO,New_Dongjin - 100mM_NaCl,Chilbo | 19.21756215 | 4.835667354 | 32.4347475 | 3.974128232 | 0.031273721 |
| Mock_DMSO,New_Dongjin - 150mM_NaCl,Chilbo | 36.16231097 | 4.828619289 | 32.24600589 | 7.489161768 | 2.02E-06 |
| Mock_DMSO,New_Dongjin - 200mM_NaCl,Chilbo | 41.23470547 | 4.835653158 | 32.43387565 | 8.527225613 | 1.19E-07 |
| 1uM_GA3,New_Dongjin - 100mM_NaCl,New_Dongjin | 44.75501243 | 4.835653677 | 32.4339099 | 9.255214583 | 1.79E-08 |
| 1uM_GA3,New_Dongjin - 150mM_NaCl,New_Dongjin | 61.68276465 | 5.35324739 | 30.18721061 | 11.52249469 | 1.98E-10 |
| 1uM_GA3,New_Dongjin - 200mM_NaCl,New_Dongjin | 80.49675722 | 4.828604366 | 32.24509691 | 16.67081233 | 1.19E-13 |
| 1uM_GA3,New_Dongjin - Mock,Chucheongbyeo | 51.678065 | 4.851621377 | 32.85538173 | 10.65171022 | 4.91E-10 |
| 1uM_GA3,New_Dongjin - Mock_DMSO,Chucheongbyeo | 50.98915244 | 4.79211276 | 31.20610762 | 10.64022384 | 9.31E-10 |
| 1uM_GA3,New_Dongjin - 1uM_GA3,Chucheongbyeo | 19.02769239 | 4.852657821 | 32.8730374 | 3.921086772 | 0.035218547 |
| 1uM_GA3,New_Dongjin - 100mM_NaCl,Chucheongbyeo | 54.87619343 | 4.835653677 | 32.4339099 | 11.34824723 | 1.13E-10 |
| 1uM_GA3,New_Dongjin - 150mM_NaCl,Chucheongbyeo | 64.11526569 | 5.321248408 | 29.5527335 | 12.04891423 | 8.83E-11 |
| 1uM_GA3,New_Dongjin - 200mM_NaCl,Chucheongbyeo | 79.9336467 | 4.836477841 | 32.44718422 | 16.52724345 | 9.96E-14 |
| 1uM_GA3,New_Dongjin - Mock,Chilbo | 62.04238483 | 4.842759923 | 32.62747011 | 12.8113691 | 4.18E-12 |
| 1uM_GA3,New_Dongjin - Mock_DMSO,Chilbo | 63.81778604 | 4.843607537 | 32.64212409 | 13.17567238 | 1.99E-12 |
| 1uM_GA3,New_Dongjin - 1uM_GA3,Chilbo | 25.44660749 | 4.843592239 | 32.6412128 | 5.253664271 | 0.001021455 |
| 1uM_GA3,New_Dongjin - 100mM_NaCl,Chilbo | 56.72238711 | 4.835667354 | 32.4347475 | 11.73000187 | 4.75E-11 |
| 1uM_GA3,New_Dongjin - 150mM_NaCl,Chilbo | 73.66713593 | 4.828619289 | 32.24600589 | 15.25635622 | 1.53E-13 |
| 1uM_GA3,New_Dongjin - 200mM_NaCl,Chilbo | 78.73953042 | 4.835653158 | 32.43387565 | 16.28312202 | 9.83E-14 |
| 100mM_NaCl,New_Dongjin - 150mM_NaCl,New_Dongjin | 16.92775222 | 5.338686992 | 29.85981909 | 3.170770688 | 0.191490794 |
| 100mM_NaCl,New_Dongjin - 200mM_NaCl,New_Dongjin | 35.7417448 | 4.813423402 | 31.84232197 | 7.425431301 | 2.60E-06 |
| 100mM_NaCl,New_Dongjin - Mock,Chucheongbyeo | 6.923052578 | 4.83649842 | 32.4481439 | 1.431418348 | 0.990117085 |
| 100mM_NaCl,New_Dongjin - Mock_DMSO,Chucheongbyeo | 6.234140015 | 4.776779825 | 30.80781289 | 1.305092603 | 0.996112517 |
| 100mM_NaCl,New_Dongjin - 1uM_GA3,Chucheongbyeo | -25.72732004 | 4.837519244 | 32.46463529 | -5.318287895 | 0.000863742 |
| 100mM_NaCl,New_Dongjin - 100mM_NaCl,Chucheongbyeo | 10.121181 | 4.820485227 | 32.0294164 | 2.099618716 | 0.797636962 |
| 100mM_NaCl,New_Dongjin - 150mM_NaCl,Chucheongbyeo | 19.36025326 | 5.306602282 | 29.22836709 | 3.648333195 | 0.072291969 |
| 100mM_NaCl,New_Dongjin - 200mM_NaCl,Chucheongbyeo | 35.17863428 | 4.821328086 | 32.04362925 | 7.2964614 | 3.56E-06 |
| 100mM_NaCl,New_Dongjin - Mock,Chilbo | 17.28737241 | 4.827617818 | 32.22206414 | 3.580932264 | 0.079358682 |
| 100mM_NaCl,New_Dongjin - Mock_DMSO,Chilbo | 19.06277362 | 4.828482365 | 32.23755954 | 3.947984517 | 0.033519687 |
| 100mM_NaCl,New_Dongjin - 1uM_GA3,Chilbo | -19.30840494 | 4.828468892 | 32.23675511 | -3.998867006 | 0.029585215 |
| 100mM_NaCl,New_Dongjin - 100mM_NaCl,Chilbo | 11.96737468 | 4.820497284 | 32.03015703 | 2.482601685 | 0.557690124 |
| 100mM_NaCl,New_Dongjin - 150mM_NaCl,Chilbo | 28.9121235 | 4.813423016 | 31.84229764 | 6.006561942 | 0.000132762 |
| 100mM_NaCl,New_Dongjin - 200mM_NaCl,Chilbo | 33.984518 | 4.82049844 | 32.03023036 | 7.050000829 | 7.04E-06 |
| 150mM_NaCl,New_Dongjin - 200mM_NaCl,New_Dongjin | 18.81399257 | 5.333110513 | 29.73517406 | 3.527770993 | 0.093073494 |
| 150mM_NaCl,New_Dongjin - Mock,Chucheongbyeo | -10.00469965 | 5.352784344 | 30.17323798 | -1.869064584 | 0.90210243 |
| 150mM_NaCl,New_Dongjin - Mock_DMSO,Chucheongbyeo | -10.69361221 | 5.297117035 | 28.89006313 | -2.018760797 | 0.837488335 |
| 150mM_NaCl,New_Dongjin - 1uM_GA3,Chucheongbyeo | -42.65507226 | 5.352152031 | 30.15001244 | -7.969704899 | 8.71E-07 |
| 150mM_NaCl,New_Dongjin - 100mM_NaCl,Chucheongbyeo | -6.806571224 | 5.338686992 | 29.85981909 | -1.274952293 | 0.99692764 |
| 150mM_NaCl,New_Dongjin - 150mM_NaCl,Chucheongbyeo | 2.432501035 | 5.717849114 | 27.86642219 | 0.42542239 | 0.999999999 |
| 150mM_NaCl,New_Dongjin - 200mM_NaCl,Chucheongbyeo | 18.25088205 | 5.340783997 | 29.90310324 | 3.417266466 | 0.11724486 |
| 150mM_NaCl,New_Dongjin - Mock,Chilbo | 0.359620182 | 5.345449434 | 30.01133593 | 0.067275949 | 1 |
| 150mM_NaCl,New_Dongjin - Mock_DMSO,Chilbo | 2.135021392 | 5.347394369 | 30.04690057 | 0.399263874 | 1 |
| 150mM_NaCl,New_Dongjin - 1uM_GA3,Chilbo | -36.23615716 | 5.347559311 | 30.05543563 | -6.776204817 | 2.04E-05 |
| 150mM_NaCl,New_Dongjin - 100mM_NaCl,Chilbo | -4.960377545 | 5.338540786 | 29.85212033 | -0.929163557 | 0.999936462 |
| 150mM_NaCl,New_Dongjin - 150mM_NaCl,Chilbo | 11.98437128 | 5.331835841 | 29.70436948 | 2.247700723 | 0.710086638 |
| 150mM_NaCl,New_Dongjin - 200mM_NaCl,Chilbo | 17.05676577 | 5.339820981 | 29.88338612 | 3.194257979 | 0.183085771 |
| 200mM_NaCl,New_Dongjin - Mock,Chucheongbyeo | -28.81869222 | 4.829465193 | 32.26022705 | -5.967263676 | 0.000141689 |
| 200mM_NaCl,New_Dongjin - Mock_DMSO,Chucheongbyeo | -29.50760478 | 4.769681091 | 30.62607522 | -6.186494279 | 9.31E-05 |
| 200mM_NaCl,New_Dongjin - 1uM_GA3,Chucheongbyeo | -61.46906483 | 4.830504288 | 32.2777025 | -12.72518585 | 5.93E-12 |
| 200mM_NaCl,New_Dongjin - 100mM_NaCl,Chucheongbyeo | -25.6205638 | 4.813423402 | 31.84232197 | -5.322732212 | 0.00089506 |
| 200mM_NaCl,New_Dongjin - 150mM_NaCl,Chucheongbyeo | -16.38149154 | 5.300992052 | 29.10484627 | -3.090269024 | 0.223756383 |
| 200mM_NaCl,New_Dongjin - 200mM_NaCl,Chucheongbyeo | -0.563110518 | 4.814253294 | 31.8556527 | -0.116967364 | 1 |
| 200mM_NaCl,New_Dongjin - Mock,Chilbo | -18.45437239 | 4.820561903 | 32.03414554 | -3.828261676 | 0.044962587 |
| 200mM_NaCl,New_Dongjin - Mock_DMSO,Chilbo | -16.67897118 | 4.82141347 | 32.04874188 | -3.459353006 | 0.103966558 |
| 200mM_NaCl,New_Dongjin - 1uM_GA3,Chilbo | -55.05014973 | 4.821399977 | 32.04794084 | -11.41787655 | 1.13E-10 |
| 200mM_NaCl,New_Dongjin - 100mM_NaCl,Chilbo | -23.77437012 | 4.813435476 | 31.84305948 | -4.939168757 | 0.002569574 |
| 200mM_NaCl,New_Dongjin - 150mM_NaCl,Chilbo | -6.829621296 | 4.806355331 | 31.65599929 | -1.420956385 | 0.990706353 |
| 200mM_NaCl,New_Dongjin - 200mM_NaCl,Chilbo | -1.757226799 | 4.813422995 | 31.84229485 | -0.365068019 | 1 |
| Mock,Chucheongbyeo - Mock_DMSO,Chucheongbyeo | -0.688912563 | 4.792894829 | 31.2157786 | -0.143736215 | 1 |
| Mock,Chucheongbyeo - 1uM_GA3,Chucheongbyeo | -32.65037261 | 4.853463709 | 32.8851287 | -6.727231225 | 1.53E-05 |
| Mock,Chucheongbyeo - 100mM_NaCl,Chucheongbyeo | 3.198128422 | 4.83649842 | 32.4481439 | 0.661248727 | 0.999999552 |
| Mock,Chucheongbyeo - 150mM_NaCl,Chucheongbyeo | 12.43720068 | 5.320767524 | 29.53818722 | 2.337482445 | 0.653274294 |
| Mock,Chucheongbyeo - 200mM_NaCl,Chucheongbyeo | 28.2555817 | 4.837338834 | 32.46242747 | 5.841141725 | 0.000198137 |
| Mock,Chucheongbyeo - Mock,Chilbo | 10.36431983 | 4.843616853 | 32.64256195 | 2.139789365 | 0.775520079 |
| Mock,Chucheongbyeo - Mock_DMSO,Chilbo | 12.13972104 | 4.844494352 | 32.65907233 | 2.505879903 | 0.542061822 |
| Mock,Chucheongbyeo - 1uM_GA3,Chilbo | -26.23145752 | 4.844465467 | 32.65732839 | -5.414726908 | 0.000649093 |
| Mock,Chucheongbyeo - 100mM_NaCl,Chilbo | 5.044322101 | 4.836524152 | 32.44973404 | 1.042964315 | 0.999733801 |
| Mock,Chucheongbyeo - 150mM_NaCl,Chilbo | 21.98907092 | 4.829463932 | 32.26014734 | 4.553108012 | 0.007110392 |
| Mock,Chucheongbyeo - 200mM_NaCl,Chilbo | 27.06146542 | 4.836526182 | 32.44986392 | 5.595227731 | 0.00039724 |
| Mock_DMSO,Chucheongbyeo - 1uM_GA3,Chucheongbyeo | -31.96146005 | 4.793901912 | 31.23083407 | -6.667107637 | 2.29E-05 |
| Mock_DMSO,Chucheongbyeo - 100mM_NaCl,Chucheongbyeo | 3.887040985 | 4.776779825 | 30.80781289 | 0.813736686 | 0.99999011 |
| Mock_DMSO,Chucheongbyeo - 150mM_NaCl,Chucheongbyeo | 13.12611324 | 5.264716822 | 28.26482973 | 2.493223033 | 0.552325696 |
| Mock_DMSO,Chucheongbyeo - 200mM_NaCl,Chucheongbyeo | 28.94449426 | 4.777645649 | 30.82280571 | 6.058317504 | 0.000129137 |
| Mock_DMSO,Chucheongbyeo - Mock,Chilbo | 11.05323239 | 4.784016297 | 30.99911475 | 2.310450405 | 0.670769369 |
| Mock_DMSO,Chucheongbyeo - Mock_DMSO,Chilbo | 12.8286336 | 4.784960245 | 31.01886795 | 2.681032432 | 0.431095844 |
| Mock_DMSO,Chucheongbyeo - 1uM_GA3,Chilbo | -25.54254495 | 4.784890441 | 31.01458927 | -5.338167147 | 0.000916342 |
| Mock_DMSO,Chucheongbyeo - 100mM_NaCl,Chilbo | 5.733234664 | 4.776841831 | 30.81168957 | 1.200214465 | 0.998476933 |
| Mock_DMSO,Chucheongbyeo - 150mM_NaCl,Chilbo | 22.67798348 | 4.769664092 | 30.62507706 | 4.754629057 | 0.004532631 |
| Mock_DMSO,Chucheongbyeo - 200mM_NaCl,Chilbo | 27.75037798 | 4.77685965 | 30.81274827 | 5.809335006 | 0.00025628 |
| 1uM_GA3,Chucheongbyeo - 100mM_NaCl,Chucheongbyeo | 35.84850104 | 4.837519244 | 32.46463529 | 7.41051337 | 2.41E-06 |
| 1uM_GA3,Chucheongbyeo - 150mM_NaCl,Chucheongbyeo | 45.08757329 | 5.320148525 | 29.51607111 | 8.474871158 | 2.85E-07 |
| 1uM_GA3,Chucheongbyeo - 200mM_NaCl,Chucheongbyeo | 60.90595431 | 4.838393467 | 32.48094652 | 12.58805319 | 7.15E-12 |
| 1uM_GA3,Chucheongbyeo - Mock,Chilbo | 43.01469244 | 4.844637731 | 32.65915789 | 8.87882538 | 4.45E-08 |
| 1uM_GA3,Chucheongbyeo - Mock_DMSO,Chilbo | 44.79009365 | 4.845533672 | 32.67679146 | 9.243583202 | 1.72E-08 |
| 1uM_GA3,Chucheongbyeo - 1uM_GA3,Chilbo | 6.418915096 | 4.845520245 | 32.6759794 | 1.324711233 | 0.995587424 |
| 1uM_GA3,Chucheongbyeo - 100mM_NaCl,Chilbo | 37.69469471 | 4.837531259 | 32.46538298 | 7.792134602 | 8.47E-07 |
| 1uM_GA3,Chucheongbyeo - 150mM_NaCl,Chilbo | 54.63944353 | 4.830471023 | 32.27570573 | 11.3114111 | 1.31E-10 |
| 1uM_GA3,Chucheongbyeo - 200mM_NaCl,Chilbo | 59.71183803 | 4.83756538 | 32.46745527 | 12.34336559 | 1.21E-11 |
| 100mM_NaCl,Chucheongbyeo - 150mM_NaCl,Chucheongbyeo | 9.239072259 | 5.306602282 | 29.22836709 | 1.741052328 | 0.94149474 |
| 100mM_NaCl,Chucheongbyeo - 200mM_NaCl,Chucheongbyeo | 25.05745328 | 4.821328086 | 32.04362925 | 5.197209737 | 0.001248138 |
| 100mM_NaCl,Chucheongbyeo - Mock,Chilbo | 7.166191406 | 4.827617818 | 32.22206414 | 1.484415643 | 0.98584822 |
| 100mM_NaCl,Chucheongbyeo - Mock_DMSO,Chilbo | 8.941592617 | 4.828482365 | 32.23755954 | 1.851843279 | 0.909427291 |
| 100mM_NaCl,Chucheongbyeo - 1uM_GA3,Chilbo | -29.42958594 | 4.828468892 | 32.23675511 | -6.095014092 | 9.91E-05 |
| 100mM_NaCl,Chucheongbyeo - 100mM_NaCl,Chilbo | 1.846193679 | 4.820497284 | 32.03015703 | 0.382988221 | 1 |
| 100mM_NaCl,Chucheongbyeo - 150mM_NaCl,Chilbo | 18.7909425 | 4.813423016 | 31.84229764 | 3.903862685 | 0.037693339 |
| 100mM_NaCl,Chucheongbyeo - 200mM_NaCl,Chilbo | 23.863337 | 4.82049844 | 32.03023036 | 4.950387869 | 0.00246346 |
| 150mM_NaCl,Chucheongbyeo - 200mM_NaCl,Chucheongbyeo | 15.81838102 | 5.308694957 | 29.27049767 | 2.979711802 | 0.27151843 |
| 150mM_NaCl,Chucheongbyeo - Mock,Chilbo | -2.072880853 | 5.313420551 | 29.37924385 | -0.390121737 | 1 |
| 150mM_NaCl,Chucheongbyeo - Mock_DMSO,Chilbo | -0.297479642 | 5.315394056 | 29.41535321 | -0.05596568 | 1 |
| 150mM_NaCl,Chucheongbyeo - 1uM_GA3,Chilbo | -38.6686582 | 5.315526096 | 29.42217197 | -7.274662469 | 6.13E-06 |
| 150mM_NaCl,Chucheongbyeo - 100mM_NaCl,Chilbo | -7.39287858 | 5.306485278 | 29.22222025 | -1.393178006 | 0.99206363 |
| 150mM_NaCl,Chucheongbyeo - 150mM_NaCl,Chilbo | 9.55187024 | 5.299724747 | 29.07506342 | 1.802333271 | 0.923942831 |
| 150mM_NaCl,Chucheongbyeo - 200mM_NaCl,Chilbo | 14.62426474 | 5.307758072 | 29.25245066 | 2.755262116 | 0.388920292 |
| 200mM_NaCl,Chucheongbyeo - Mock,Chilbo | -17.89126187 | 4.828459131 | 32.23626856 | -3.705377096 | 0.059741469 |
| 200mM_NaCl,Chucheongbyeo - Mock_DMSO,Chilbo | -16.11586066 | 4.829308901 | 32.25084578 | -3.337094601 | 0.13428372 |
| 200mM_NaCl,Chucheongbyeo - 1uM_GA3,Chilbo | -54.48703922 | 4.829280064 | 32.24913487 | -11.28264224 | 1.41E-10 |
| 200mM_NaCl,Chucheongbyeo - 100mM_NaCl,Chilbo | -23.2112596 | 4.821353777 | 32.04518946 | -4.814261859 | 0.003564972 |
| 200mM_NaCl,Chucheongbyeo - 150mM_NaCl,Chilbo | -6.266510778 | 4.814281013 | 31.85732784 | -1.301650394 | 0.996296525 |
| 200mM_NaCl,Chucheongbyeo - 200mM_NaCl,Chilbo | -1.194116281 | 4.821326754 | 32.0435409 | -0.247673792 | 1 |
| Mock,Chilbo - Mock_DMSO,Chilbo | 1.775401211 | 4.83558106 | 32.42943694 | 0.367153645 | 1 |
| Mock,Chilbo - 1uM_GA3,Chilbo | -36.59577735 | 4.835581147 | 32.42944839 | -7.568020519 | 1.57E-06 |
| Mock,Chilbo - 100mM_NaCl,Chilbo | -5.319997727 | 4.827617843 | 32.22206743 | -1.101992308 | 0.999467456 |
| Mock,Chilbo - 150mM_NaCl,Chilbo | 11.62475109 | 4.820562285 | 32.03416956 | 2.411492769 | 0.604751709 |
| Mock,Chilbo - 200mM_NaCl,Chilbo | 16.69714559 | 4.827618227 | 32.22209198 | 3.458671503 | 0.103863919 |
| Mock_DMSO,Chilbo - 1uM_GA3,Chilbo | -38.37117856 | 4.836415753 | 32.44318905 | -7.933804808 | 5.79E-07 |
| Mock_DMSO,Chilbo - 100mM_NaCl,Chilbo | -7.095398938 | 4.828468877 | 32.23675366 | -1.469492528 | 0.98716558 |
| Mock_DMSO,Chilbo - 150mM_NaCl,Chilbo | 9.849349883 | 4.821428395 | 32.04966863 | 2.042828198 | 0.827450891 |
| Mock_DMSO,Chilbo - 200mM_NaCl,Chilbo | 14.92174438 | 4.828454681 | 32.23586363 | 3.090376811 | 0.218330336 |
| 1uM_GA3,Chilbo - 100mM_NaCl,Chilbo | 31.27577962 | 4.828482582 | 32.23758816 | 6.47735165 | 3.38E-05 |
| 1uM_GA3,Chilbo - 150mM_NaCl,Chilbo | 48.22052844 | 4.821428539 | 32.04968717 | 10.00129485 | 3.14E-09 |
| 1uM_GA3,Chilbo - 200mM_NaCl,Chilbo | 53.29292294 | 4.828454713 | 32.23586773 | 11.03726267 | 2.50E-10 |
| 100mM_NaCl,Chilbo - 150mM_NaCl,Chilbo | 16.94474882 | 4.813422992 | 31.84229443 | 3.520311605 | 0.091339566 |
| 100mM_NaCl,Chilbo - 200mM_NaCl,Chilbo | 22.01714332 | 4.820498513 | 32.03024018 | 4.56739967 | 0.006919103 |
| 150mM_NaCl,Chilbo - 200mM_NaCl,Chilbo | 5.072394497 | 4.813437014 | 31.84315598 | 1.053798872 | 0.999691894 |

Internode Length (mm)

| contrast | estimate | SE | df | t.ratio | p.value |
| --- | --- | --- | --- | --- | --- |
| Mock,New_Dongjin - Mock_DMSO,New_Dongjin | -0.99699625 | 2.314601773 | 33.04327187 | -0.430742023 | 0.999999999 |
| Mock,New_Dongjin - 1uM_GA3,New_Dongjin | -28.85239274 | 2.314601773 | 33.04327187 | -12.4653809 | 7.12E-12 |
| Mock,New_Dongjin - 100mM_NaCl,New_Dongjin | 4.028131141 | 2.309661723 | 32.76269811 | 1.744035111 | 0.942433675 |
| Mock,New_Dongjin - 150mM_NaCl,New_Dongjin | 9.988692828 | 2.568847826 | 31.19368691 | 3.888394138 | 0.039776259 |
| Mock,New_Dongjin - 200mM_NaCl,New_Dongjin | 14.66149725 | 2.307360227 | 32.6322838 | 6.354229859 | 4.54E-05 |
| Mock,New_Dongjin - Mock,Chucheongbyeo | 4.40220657 | 2.314904845 | 33.0567897 | 1.901679276 | 0.891415389 |
| Mock,New_Dongjin - Mock_DMSO,Chucheongbyeo | 4.147608338 | 2.295675416 | 31.94168639 | 1.806705037 | 0.924177256 |
| Mock,New_Dongjin - 1uM_GA3,Chucheongbyeo | -21.42583341 | 2.315275526 | 33.07288155 | -9.254118211 | 1.50E-08 |
| Mock,New_Dongjin - 100mM_NaCl,Chucheongbyeo | 6.479428276 | 2.309661723 | 32.76269811 | 2.805358122 | 0.356042009 |
| Mock,New_Dongjin - 150mM_NaCl,Chucheongbyeo | 9.705702079 | 2.558158521 | 30.71728626 | 3.794019018 | 0.050264624 |
| Mock,New_Dongjin - 200mM_NaCl,Chucheongbyeo | 14.78369865 | 2.309956527 | 32.77511817 | 6.399989987 | 3.92E-05 |
| Mock,New_Dongjin - Mock,Chilbo | 4.413582 | 2.311975954 | 32.89535254 | 1.909008609 | 0.888438907 |
| Mock,New_Dongjin - Mock_DMSO,Chilbo | 5.810211006 | 2.312276708 | 32.90854772 | 2.512766308 | 0.537407399 |
| Mock,New_Dongjin - 1uM_GA3,Chilbo | -26.17401577 | 2.312272692 | 32.90804012 | -11.31960597 | 9.95E-11 |
| Mock,New_Dongjin - 100mM_NaCl,Chilbo | 3.566097165 | 2.309665276 | 32.76315548 | 1.543988734 | 0.979651836 |
| Mock,New_Dongjin - 150mM_NaCl,Chilbo | 10.68876412 | 2.307364089 | 32.63277929 | 4.632456652 | 5.65E-03 |
| Mock,New_Dongjin - 200mM_NaCl,Chilbo | 13.19619295 | 2.309661578 | 32.76267843 | 5.713474685 | 2.76E-04 |
| Mock_DMSO,New_Dongjin - 1uM_GA3,New_Dongjin | -27.85539649 | 2.306211394 | 32.56750446 | -12.07842289 | 2.06E-11 |
| Mock_DMSO,New_Dongjin - 100mM_NaCl,New_Dongjin | 5.025127392 | 2.301253108 | 32.28883717 | 2.18364828 | 0.749822496 |
| Mock_DMSO,New_Dongjin - 150mM_NaCl,New_Dongjin | 10.98568908 | 2.561264855 | 30.82701304 | 4.289165588 | 0.01499808 |
| Mock_DMSO,New_Dongjin - 200mM_NaCl,New_Dongjin | 15.6584935 | 2.298943372 | 32.1593474 | 6.811169726 | 1.34E-05 |
| Mock_DMSO,New_Dongjin - Mock,Chucheongbyeo | 5.39920282 | 2.306515513 | 32.58094699 | 2.340848257 | 0.651196308 |
| Mock_DMSO,New_Dongjin - Mock_DMSO,Chucheongbyeo | 5.144604589 | 2.287215807 | 31.47382398 | 2.249286916 | 0.7095689 |
| Mock_DMSO,New_Dongjin - 1uM_GA3,Chucheongbyeo | -20.42883716 | 2.306886936 | 32.59689035 | -8.85558665 | 4.81E-08 |
| Mock_DMSO,New_Dongjin - 100mM_NaCl,Chucheongbyeo | 7.476424526 | 2.301253108 | 32.28883717 | 3.24884929 | 0.160653065 |
| Mock_DMSO,New_Dongjin - 150mM_NaCl,Chucheongbyeo | 10.70269833 | 2.550544363 | 30.35316148 | 4.196240804 | 0.019277964 |
| Mock_DMSO,New_Dongjin - 200mM_NaCl,Chucheongbyeo | 15.7806949 | 2.30154954 | 32.30126265 | 6.856552346 | 1.16E-05 |
| Mock_DMSO,New_Dongjin - Mock,Chilbo | 5.410578251 | 2.303575638 | 32.42055019 | 2.348773863 | 0.646013284 |
| Mock_DMSO,New_Dongjin - Mock_DMSO,Chilbo | 6.807207257 | 2.3038775 | 32.43368138 | 2.954674133 | 0.278359067 |
| Mock_DMSO,New_Dongjin - 1uM_GA3,Chilbo | -25.17701952 | 2.30387401 | 32.43324137 | -10.92812342 | 2.99E-10 |
| Mock_DMSO,New_Dongjin - 100mM_NaCl,Chilbo | 4.563093415 | 2.301256198 | 32.28923412 | 1.982870668 | 0.856472353 |
| Mock_DMSO,New_Dongjin - 150mM_NaCl,Chilbo | 11.68576037 | 2.298946742 | 32.15977866 | 5.083093121 | 1.70E-03 |
| Mock_DMSO,New_Dongjin - 200mM_NaCl,Chilbo | 14.1931892 | 2.301252993 | 32.28882164 | 6.16759185 | 8.03E-05 |
| 1uM_GA3,New_Dongjin - 100mM_NaCl,New_Dongjin | 32.88052388 | 2.301253108 | 32.28883717 | 14.28809537 | 3.51E-13 |
| 1uM_GA3,New_Dongjin - 150mM_NaCl,New_Dongjin | 38.84108556 | 2.561264855 | 30.82701304 | 15.16480636 | 1.93E-13 |
| 1uM_GA3,New_Dongjin - 200mM_NaCl,New_Dongjin | 43.51388999 | 2.298943372 | 32.1593474 | 18.92777809 | 1.03E-13 |
| 1uM_GA3,New_Dongjin - Mock,Chucheongbyeo | 33.25459931 | 2.306515513 | 32.58094699 | 14.41767858 | 2.61E-13 |
| 1uM_GA3,New_Dongjin - Mock_DMSO,Chucheongbyeo | 33.00000107 | 2.287215807 | 31.47382398 | 14.42802248 | 4.05E-13 |
| 1uM_GA3,New_Dongjin - 1uM_GA3,Chucheongbyeo | 7.42655933 | 2.306886936 | 32.59689035 | 3.219299227 | 0.169890739 |
| 1uM_GA3,New_Dongjin - 100mM_NaCl,Chucheongbyeo | 35.33182101 | 2.301253108 | 32.28883717 | 15.35329638 | 1.36E-13 |
| 1uM_GA3,New_Dongjin - 150mM_NaCl,Chucheongbyeo | 38.55809482 | 2.550544363 | 30.35316148 | 15.11759426 | 2.25E-13 |
| 1uM_GA3,New_Dongjin - 200mM_NaCl,Chucheongbyeo | 43.63609138 | 2.30154954 | 32.30126265 | 18.95944042 | 1.12E-13 |
| 1uM_GA3,New_Dongjin - Mock,Chilbo | 33.26597474 | 2.303575638 | 32.42055019 | 14.44101691 | 2.75E-13 |
| 1uM_GA3,New_Dongjin - Mock_DMSO,Chilbo | 34.66260374 | 2.3038775 | 32.43368138 | 15.04533281 | 1.57E-13 |
| 1uM_GA3,New_Dongjin - 1uM_GA3,Chilbo | 2.678376965 | 2.30387401 | 32.43324137 | 1.162553574 | 0.998988543 |
| 1uM_GA3,New_Dongjin - 100mM_NaCl,Chilbo | 32.4184899 | 2.301256198 | 32.28923412 | 14.0873015 | 4.79E-13 |
| 1uM_GA3,New_Dongjin - 150mM_NaCl,Chilbo | 39.54115686 | 2.298946742 | 32.15977866 | 17.19968372 | 1.01E-13 |
| 1uM_GA3,New_Dongjin - 200mM_NaCl,Chilbo | 42.04858569 | 2.301252993 | 32.28882164 | 18.27203954 | 1.06E-13 |
| 100mM_NaCl,New_Dongjin - 150mM_NaCl,New_Dongjin | 5.960561687 | 2.556518499 | 30.59903817 | 2.33151518 | 0.657173652 |
| 100mM_NaCl,New_Dongjin - 200mM_NaCl,New_Dongjin | 10.63336611 | 2.293969194 | 31.88233128 | 4.635356978 | 5.82E-03 |
| 100mM_NaCl,New_Dongjin - Mock,Chucheongbyeo | 0.374075429 | 2.301554496 | 32.30180131 | 0.162531641 | 1 |
| 100mM_NaCl,New_Dongjin - Mock_DMSO,Chucheongbyeo | 0.119477197 | 2.282208069 | 31.19875228 | 0.052351579 | 1 |
| 100mM_NaCl,New_Dongjin - 1uM_GA3,Chucheongbyeo | -25.45396455 | 2.301922416 | 32.31715834 | -11.05769872 | 2.31E-10 |
| 100mM_NaCl,New_Dongjin - 100mM_NaCl,Chucheongbyeo | 2.451297135 | 2.296281752 | 32.01100864 | 1.067507126 | 0.999639127 |
| 100mM_NaCl,New_Dongjin - 150mM_NaCl,Chucheongbyeo | 5.677570938 | 2.545778502 | 30.12679349 | 2.230190463 | 0.720984011 |
| 100mM_NaCl,New_Dongjin - 200mM_NaCl,Chucheongbyeo | 10.7555675 | 2.296582467 | 32.02386107 | 4.683292527 | 5.08E-03 |
| 100mM_NaCl,New_Dongjin - Mock,Chilbo | 0.385450859 | 2.298610202 | 32.14229388 | 0.167688658 | 1 |
| 100mM_NaCl,New_Dongjin - Mock_DMSO,Chilbo | 1.782079865 | 2.298915926 | 32.15580111 | 0.775182705 | 0.999995278 |
| 100mM_NaCl,New_Dongjin - 1uM_GA3,Chilbo | -30.20214691 | 2.298912867 | 32.15541494 | -13.13757791 | 2.71E-12 |
| 100mM_NaCl,New_Dongjin - 100mM_NaCl,Chilbo | -0.462033976 | 2.296284462 | 32.01135747 | -0.201209381 | 1 |
| 100mM_NaCl,New_Dongjin - 150mM_NaCl,Chilbo | 6.660632982 | 2.293969107 | 31.88232002 | 2.903540837 | 0.304522514 |
| 100mM_NaCl,New_Dongjin - 200mM_NaCl,Chilbo | 9.168061813 | 2.296284719 | 32.01139136 | 3.992563176 | 3.02E-02 |
| 150mM_NaCl,New_Dongjin - 200mM_NaCl,New_Dongjin | 4.672804421 | 2.554701638 | 30.51211943 | 1.829099865 | 0.916208408 |
| 150mM_NaCl,New_Dongjin - Mock,Chucheongbyeo | -5.586486258 | 2.561126675 | 30.81863283 | -2.181261206 | 0.750677921 |
| 150mM_NaCl,New_Dongjin - Mock_DMSO,Chucheongbyeo | -5.84108449 | 2.543165902 | 29.9409574 | -2.296776819 | 0.679362111 |
| 150mM_NaCl,New_Dongjin - 1uM_GA3,Chucheongbyeo | -31.41452624 | 2.560953303 | 30.80587463 | -12.26673138 | 3.10E-11 |
| 150mM_NaCl,New_Dongjin - 100mM_NaCl,Chucheongbyeo | -3.509264553 | 2.556518499 | 30.59903817 | -1.372673249 | 0.993346718 |
| 150mM_NaCl,New_Dongjin - 150mM_NaCl,Chucheongbyeo | -0.28299075 | 2.752973943 | 29.24319057 | -0.102794562 | 1 |
| 150mM_NaCl,New_Dongjin - 200mM_NaCl,Chucheongbyeo | 4.795005817 | 2.55721568 | 30.63060434 | 1.875088541 | 0.900165556 |
| 150mM_NaCl,New_Dongjin - Mock,Chilbo | -5.575110828 | 2.55872282 | 30.70469003 | -2.178864699 | 0.752046262 |
| 150mM_NaCl,New_Dongjin - Mock_DMSO,Chilbo | -4.178481822 | 2.559386161 | 30.73260049 | -1.63261093 | 0.965850706 |
| 150mM_NaCl,New_Dongjin - 1uM_GA3,Chilbo | -36.1627086 | 2.559422971 | 30.73667185 | -14.12924281 | 8.77E-13 |
| 150mM_NaCl,New_Dongjin - 100mM_NaCl,Chilbo | -6.422595663 | 2.556486098 | 30.59541089 | -2.512274825 | 0.538789351 |
| 150mM_NaCl,New_Dongjin - 150mM_NaCl,Chilbo | 0.700071294 | 2.55429464 | 30.49155987 | 0.274076171 | 1 |
| 150mM_NaCl,New_Dongjin - 200mM_NaCl,Chilbo | 3.207500126 | 2.556894317 | 30.6161838 | 1.254451584 | 0.997473302 |
| 200mM_NaCl,New_Dongjin - Mock,Chucheongbyeo | -10.25929068 | 2.299248408 | 32.17272483 | -4.462019259 | 0.009082815 |
| 200mM_NaCl,New_Dongjin - Mock_DMSO,Chucheongbyeo | -10.51388891 | 2.279887392 | 31.07250247 | -4.61158255 | 6.45E-03 |
| 200mM_NaCl,New_Dongjin - 1uM_GA3,Chucheongbyeo | -36.08733066 | 2.299620511 | 32.18854321 | -15.69273299 | 1.25E-13 |
| 200mM_NaCl,New_Dongjin - 100mM_NaCl,Chucheongbyeo | -8.182068973 | 2.293969194 | 31.88233128 | -3.566773694 | 0.08237723 |
| 200mM_NaCl,New_Dongjin - 150mM_NaCl,Chucheongbyeo | -4.95579517 | 2.543953971 | 30.04047155 | -1.948067939 | 0.870602063 |
| 200mM_NaCl,New_Dongjin - 200mM_NaCl,Chucheongbyeo | 0.122201397 | 2.294267015 | 31.89475751 | 0.053263808 | 1 |
| 200mM_NaCl,New_Dongjin - Mock,Chilbo | -10.24791525 | 2.29629897 | 32.01323077 | -4.462796606 | 0.009126814 |
| 200mM_NaCl,New_Dongjin - Mock_DMSO,Chilbo | -8.851286243 | 2.296601799 | 32.02630615 | -3.854079644 | 0.042285569 |
| 200mM_NaCl,New_Dongjin - 1uM_GA3,Chilbo | -40.83551302 | 2.296598737 | 32.0259211 | -17.7808654 | 9.26E-14 |
| 200mM_NaCl,New_Dongjin - 100mM_NaCl,Chilbo | -11.09540008 | 2.293971906 | 31.8826791 | -4.836763718 | 0.00338538 |
| 200mM_NaCl,New_Dongjin - 150mM_NaCl,Chilbo | -3.972733126 | 2.291655231 | 31.75401934 | -1.733564924 | 0.944658489 |
| 200mM_NaCl,New_Dongjin - 200mM_NaCl,Chilbo | -1.465304294 | 2.293969104 | 31.88231912 | -0.638763744 | 0.999999729 |
| Mock,Chucheongbyeo - Mock_DMSO,Chucheongbyeo | -0.254598232 | 2.28750346 | 31.48462779 | -0.111299605 | 1 |
| Mock,Chucheongbyeo - 1uM_GA3,Chucheongbyeo | -25.82803998 | 2.307179419 | 32.60885144 | -11.19463869 | 1.50E-10 |
| Mock,Chucheongbyeo - 100mM_NaCl,Chucheongbyeo | 2.077221706 | 2.301554496 | 32.30180131 | 0.902529881 | 0.999960254 |
| Mock,Chucheongbyeo - 150mM_NaCl,Chucheongbyeo | 5.303495509 | 2.550402217 | 30.34449061 | 2.07947416 | 0.807574051 |
| Mock,Chucheongbyeo - 200mM_NaCl,Chucheongbyeo | 10.38149208 | 2.30185459 | 32.31470488 | 4.510055554 | 0.007951482 |
| Mock,Chucheongbyeo - Mock,Chilbo | 0.01137543 | 2.303879745 | 32.43392647 | 0.00493751 | 1 |
| Mock,Chucheongbyeo - Mock_DMSO,Chilbo | 1.408004436 | 2.304188353 | 32.4479361 | 0.611063082 | 0.999999865 |
| Mock,Chucheongbyeo - 1uM_GA3,Chilbo | -30.57622234 | 2.304181786 | 32.44710052 | -13.26988284 | 1.83E-12 |
| Mock,Chucheongbyeo - 100mM_NaCl,Chilbo | -0.836109405 | 2.301560296 | 32.3025511 | -0.363279383 | 1 |
| Mock,Chucheongbyeo - 150mM_NaCl,Chilbo | 6.286557553 | 2.299248126 | 32.17268782 | 2.734179701 | 0.397866597 |
| Mock,Chucheongbyeo - 200mM_NaCl,Chilbo | 8.793986384 | 2.30156075 | 32.30261111 | 3.820879542 | 0.045477022 |
| Mock_DMSO,Chucheongbyeo - 1uM_GA3,Chucheongbyeo | -25.57344174 | 2.287868389 | 31.49921114 | -11.17784654 | 2.43E-10 |
| Mock_DMSO,Chucheongbyeo - 100mM_NaCl,Chucheongbyeo | 2.331819938 | 2.282208069 | 31.19875228 | 1.021738539 | 0.999788319 |
| Mock_DMSO,Chucheongbyeo - 150mM_NaCl,Chucheongbyeo | 5.558093741 | 2.532355583 | 29.47194381 | 2.194831475 | 0.742097945 |
| Mock_DMSO,Chucheongbyeo - 200mM_NaCl,Chucheongbyeo | 10.63609031 | 2.282514042 | 31.21191802 | 4.659813745 | 0.00564012 |
| Mock_DMSO,Chucheongbyeo - Mock,Chilbo | 0.265973662 | 2.284559413 | 31.32948102 | 0.1164223 | 1 |
| Mock_DMSO,Chucheongbyeo - Mock_DMSO,Chilbo | 1.662602668 | 2.284882891 | 31.34491383 | 0.72765334 | 0.999998065 |
| Mock_DMSO,Chucheongbyeo - 1uM_GA3,Chilbo | -30.32162411 | 2.284867353 | 31.34291607 | -13.27062776 | 3.14E-12 |
| Mock_DMSO,Chucheongbyeo - 100mM_NaCl,Chilbo | -0.581511173 | 2.282221767 | 31.20053777 | -0.254800467 | 1 |
| Mock_DMSO,Chucheongbyeo - 150mM_NaCl,Chilbo | 6.541155785 | 2.279883582 | 31.07202666 | 2.869074472 | 0.323529436 |
| Mock_DMSO,Chucheongbyeo - 200mM_NaCl,Chilbo | 9.048584616 | 2.282225756 | 31.20104 | 3.964806983 | 0.033098877 |
| 1uM_GA3,Chucheongbyeo - 100mM_NaCl,Chucheongbyeo | 27.90526168 | 2.301922416 | 32.31715834 | 12.12259001 | 2.10E-11 |
| 1uM_GA3,Chucheongbyeo - 150mM_NaCl,Chucheongbyeo | 31.13153549 | 2.550231983 | 30.33225578 | 12.20733474 | 4.39E-11 |
| 1uM_GA3,Chucheongbyeo - 200mM_NaCl,Chucheongbyeo | 36.20953205 | 2.302230201 | 32.3310398 | 15.72802409 | 1.22E-13 |
| 1uM_GA3,Chucheongbyeo - Mock,Chilbo | 25.83941541 | 2.304247656 | 32.44934749 | 11.21381868 | 1.53E-10 |
| 1uM_GA3,Chucheongbyeo - Mock_DMSO,Chilbo | 27.23604441 | 2.304560453 | 32.46389729 | 11.818325 | 3.85E-11 |
| 1uM_GA3,Chucheongbyeo - 1uM_GA3,Chilbo | -4.748182364 | 2.304557402 | 32.46350848 | -2.060344585 | 0.818714823 |
| 1uM_GA3,Chucheongbyeo - 100mM_NaCl,Chilbo | 24.99193057 | 2.301925119 | 32.31750956 | 10.8569694 | 3.69E-10 |
| 1uM_GA3,Chucheongbyeo - 150mM_NaCl,Chilbo | 32.11459753 | 2.299612964 | 32.18758714 | 13.96521851 | 5.83E-13 |
| 1uM_GA3,Chucheongbyeo - 200mM_NaCl,Chilbo | 34.62202636 | 2.301932851 | 32.31849697 | 15.04041543 | 1.67E-13 |
| 100mM_NaCl,Chucheongbyeo - 150mM_NaCl,Chucheongbyeo | 3.226273803 | 2.545778502 | 30.12679349 | 1.26730342 | 0.997141014 |
| 100mM_NaCl,Chucheongbyeo - 200mM_NaCl,Chucheongbyeo | 8.30427037 | 2.296582467 | 32.02386107 | 3.61592518 | 0.073603248 |
| 100mM_NaCl,Chucheongbyeo - Mock,Chilbo | -2.065846276 | 2.298610202 | 32.14229388 | -0.898737104 | 0.999962294 |
| 100mM_NaCl,Chucheongbyeo - Mock_DMSO,Chilbo | -0.669217269 | 2.298915926 | 32.15580111 | -0.291101237 | 1 |
| 100mM_NaCl,Chucheongbyeo - 1uM_GA3,Chilbo | -32.65344405 | 2.298912867 | 32.15541494 | -14.20386328 | 4.04E-13 |
| 100mM_NaCl,Chucheongbyeo - 100mM_NaCl,Chilbo | -2.913331111 | 2.296284462 | 32.01135747 | -1.268715248 | 0.997213775 |
| 100mM_NaCl,Chucheongbyeo - 150mM_NaCl,Chilbo | 4.209335847 | 2.293969107 | 31.88232002 | 1.834957512 | 0.91501507 |
| 100mM_NaCl,Chucheongbyeo - 200mM_NaCl,Chilbo | 6.716764679 | 2.296284719 | 32.01139136 | 2.925057429 | 0.293492953 |
| 150mM_NaCl,Chucheongbyeo - 200mM_NaCl,Chucheongbyeo | 5.077996567 | 2.546474775 | 30.15775635 | 1.994127968 | 0.850014513 |
| 150mM_NaCl,Chucheongbyeo - Mock,Chilbo | -5.292120078 | 2.547995491 | 30.23207769 | -2.076973879 | 0.808832082 |
| 150mM_NaCl,Chucheongbyeo - Mock_DMSO,Chilbo | -3.895491072 | 2.54866545 | 30.26021993 | -1.528443473 | 0.980866166 |
| 150mM_NaCl,Chucheongbyeo - 1uM_GA3,Chilbo | -35.87971785 | 2.548694733 | 30.26344962 | -14.07768353 | 1.23E-12 |
| 150mM_NaCl,Chucheongbyeo - 100mM_NaCl,Chilbo | -6.139604914 | 2.545752731 | 30.12391716 | -2.411705127 | 0.604936301 |
| 150mM_NaCl,Chucheongbyeo - 150mM_NaCl,Chilbo | 0.983062044 | 2.543548645 | 30.02041707 | 0.386492331 | 1 |
| 150mM_NaCl,Chucheongbyeo - 200mM_NaCl,Chilbo | 3.490490876 | 2.546159274 | 30.14418029 | 1.370884733 | 0.993382023 |
| 200mM_NaCl,Chucheongbyeo - Mock,Chilbo | -10.37011665 | 2.298910554 | 32.15515399 | -4.510883048 | 0.007990697 |
| 200mM_NaCl,Chucheongbyeo - Mock_DMSO,Chilbo | -8.973487639 | 2.299212962 | 32.16823433 | -3.902851884 | 0.037473732 |
| 200mM_NaCl,Chucheongbyeo - 1uM_GA3,Chilbo | -40.95771442 | 2.299206403 | 32.16740972 | -17.81384845 | 1.04E-13 |
| 200mM_NaCl,Chucheongbyeo - 100mM_NaCl,Chilbo | -11.21760148 | 2.29658826 | 32.02460106 | -4.884463479 | 0.0029497 |
| 200mM_NaCl,Chucheongbyeo - 150mM_NaCl,Chilbo | -4.094934523 | 2.294273262 | 31.89555267 | -1.784850388 | 0.930759099 |
| 200mM_NaCl,Chucheongbyeo - 200mM_NaCl,Chilbo | -1.587505691 | 2.296582173 | 32.02382124 | -0.691247067 | 0.999999116 |
| Mock,Chilbo - Mock_DMSO,Chilbo | 1.396629006 | 2.301236725 | 32.2867247 | 0.606903667 | 0.999999878 |
| Mock,Chilbo - 1uM_GA3,Chilbo | -30.58759777 | 2.301236739 | 32.28672843 | -13.29180838 | 1.89E-12 |
| Mock,Chilbo - 100mM_NaCl,Chilbo | -0.847484835 | 2.298610206 | 32.14229493 | -0.368694454 | 1 |
| Mock,Chilbo - 150mM_NaCl,Chilbo | 6.275182123 | 2.296299056 | 32.01324193 | 2.732737318 | 0.398891211 |
| Mock,Chilbo - 200mM_NaCl,Chilbo | 8.782610954 | 2.298610292 | 32.14230626 | 3.82083513 | 0.045649208 |
| Mock_DMSO,Chilbo - 1uM_GA3,Chilbo | -31.98422678 | 2.301535702 | 32.29942269 | -13.89690664 | 6.45E-13 |
| Mock_DMSO,Chilbo - 100mM_NaCl,Chilbo | -2.244113841 | 2.298912865 | 32.15541441 | -0.976163071 | 0.999885946 |
| Mock_DMSO,Chilbo - 150mM_NaCl,Chilbo | 4.878553116 | 2.296605145 | 32.02674002 | 2.124245488 | 0.784035059 |
| Mock_DMSO,Chilbo - 200mM_NaCl,Chilbo | 7.385981948 | 2.298909684 | 32.15499922 | 3.212819537 | 0.172785534 |
| 1uM_GA3,Chilbo - 100mM_NaCl,Chilbo | 29.74011294 | 2.298915959 | 32.15581039 | 12.93658118 | 4.06E-12 |
| 1uM_GA3,Chilbo - 150mM_NaCl,Chilbo | 36.86277989 | 2.296605167 | 32.02674608 | 16.05098709 | 1.10E-13 |
| 1uM_GA3,Chilbo - 200mM_NaCl,Chilbo | 39.37020873 | 2.298909689 | 32.15500057 | 17.12560041 | 1.01E-13 |
| 100mM_NaCl,Chilbo - 150mM_NaCl,Chilbo | 7.122666958 | 2.293969104 | 31.88231898 | 3.104953308 | 0.213028164 |
| 100mM_NaCl,Chilbo - 200mM_NaCl,Chilbo | 9.630095789 | 2.296284731 | 32.01139453 | 4.193772514 | 0.018290324 |
| 150mM_NaCl,Chilbo - 200mM_NaCl,Chilbo | 2.507428832 | 2.293972249 | 31.88272388 | 1.093051074 | 0.999514315 |

Coleoptile Length (mm)

| contrast | estimate | SE | df | t.ratio | p.value |
| --- | --- | --- | --- | --- | --- |
| Mock,New_Dongjin - Mock_DMSO,New_Dongjin | 0.39915377 | 0.763611036 | 39.61042094 | 0.522718702 | 0.999999991 |
| Mock,New_Dongjin - 1uM_GA3,New_Dongjin | -0.428844066 | 0.763611036 | 39.61042094 | -0.561600141 | 1.00E+00 |
| Mock,New_Dongjin - 100mM_NaCl,New_Dongjin | 0.519508169 | 0.759444915 | 38.75707019 | 0.684063002 | 0.999999385 |
| Mock,New_Dongjin - 150mM_NaCl,New_Dongjin | -0.097438709 | 0.826694422 | 33.11306296 | -0.117865449 | 1 |
| Mock,New_Dongjin - 200mM_NaCl,New_Dongjin | -0.444614751 | 0.757493879 | 38.36024399 | -0.586954909 | 1.00E+00 |
| Mock,New_Dongjin - Mock,Chucheongbyeo | 2.058649442 | 0.763795771 | 39.61761197 | 2.695287823 | 0.415299571 |
| Mock,New_Dongjin - Mock_DMSO,Chucheongbyeo | 1.723825104 | 0.746848283 | 35.93851114 | 2.308132915 | 0.672776697 |
| Mock,New_Dongjin - 1uM_GA3,Chucheongbyeo | -0.157726201 | 0.764015417 | 39.62629674 | -0.206443742 | 1.00E+00 |
| Mock,New_Dongjin - 100mM_NaCl,Chucheongbyeo | 2.695166956 | 0.759444915 | 38.75707019 | 3.54886431 | 0.077740479 |
| Mock,New_Dongjin - 150mM_NaCl,Chucheongbyeo | 2.091964261 | 0.818372914 | 32.08128873 | 2.556248166 | 0.509298444 |
| Mock,New_Dongjin - 200mM_NaCl,Chucheongbyeo | 1.672893724 | 0.759613544 | 38.75866636 | 2.202295809 | 7.41E-01 |
| Mock,New_Dongjin - Mock,Chilbo | 0.730160277 | 0.726265026 | 40.26481329 | 1.005363402 | 0.999859972 |
| Mock,New_Dongjin - Mock_DMSO,Chilbo | -0.267491599 | 0.727787623 | 40.49548831 | -0.367540737 | 1 |
| Mock,New_Dongjin - 1uM_GA3,Chilbo | -1.867808548 | 0.724091049 | 39.85249934 | -2.579521663 | 4.90E-01 |
| Mock,New_Dongjin - 100mM_NaCl,Chilbo | 0.660343353 | 0.720871902 | 39.19462734 | 0.916034251 | 0.999958996 |
| Mock,New_Dongjin - 150mM_NaCl,Chilbo | -0.372215203 | 0.719573102 | 38.9090922 | -0.517272258 | 1.00E+00 |
| Mock,New_Dongjin - 200mM_NaCl,Chilbo | -0.211024337 | 0.720865891 | 39.19173876 | -0.292737303 | 1.00E+00 |
| Mock_DMSO,New_Dongjin - 1uM_GA3,New_Dongjin | -0.827997836 | 0.756522013 | 38.16465151 | -1.094479502 | 1.00E+00 |
| Mock_DMSO,New_Dongjin - 100mM_NaCl,New_Dongjin | 0.120354399 | 0.752316213 | 37.32609014 | 0.159978473 | 1 |
| Mock_DMSO,New_Dongjin - 150mM_NaCl,New_Dongjin | -0.496592479 | 0.82012826 | 32.07135876 | -0.605505874 | 0.999999881 |
| Mock_DMSO,New_Dongjin - 200mM_NaCl,New_Dongjin | -0.843768521 | 0.750346988 | 36.93642488 | -1.124504441 | 9.99E-01 |
| Mock_DMSO,New_Dongjin - Mock,Chucheongbyeo | 1.659495672 | 0.756708378 | 38.17219099 | 2.193045196 | 0.746012969 |
| Mock_DMSO,New_Dongjin - Mock_DMSO,Chucheongbyeo | 1.324671334 | 0.739599105 | 34.56407942 | 1.791066709 | 0.930166516 |
| Mock_DMSO,New_Dongjin - 1uM_GA3,Chucheongbyeo | -0.556879971 | 0.75692895 | 38.18095808 | -0.735709701 | 1.00E+00 |
| Mock_DMSO,New_Dongjin - 100mM_NaCl,Chucheongbyeo | 2.296013186 | 0.752316213 | 37.32609014 | 3.0519257 | 0.227412599 |
| Mock_DMSO,New_Dongjin - 150mM_NaCl,Chucheongbyeo | 1.692810491 | 0.811740572 | 31.05166805 | 2.085408255 | 0.804802153 |
| Mock_DMSO,New_Dongjin - 200mM_NaCl,Chucheongbyeo | 1.273739954 | 0.752487485 | 37.32854527 | 1.692705832 | 9.56E-01 |
| Mock_DMSO,New_Dongjin - Mock,Chilbo | 0.331006507 | 0.718807061 | 38.64186558 | 0.460494234 | 0.999999999 |
| Mock_DMSO,New_Dongjin - Mock_DMSO,Chilbo | -0.666645369 | 0.720345456 | 38.87008819 | -0.925452314 | 0.999952523 |
| Mock_DMSO,New_Dongjin - 1uM_GA3,Chilbo | -2.266962318 | 0.716611304 | 38.23658143 | -3.163447613 | 1.82E-01 |
| Mock_DMSO,New_Dongjin - 100mM_NaCl,Chilbo | 0.261189583 | 0.713357055 | 37.59020784 | 0.366141446 | 1 |
| Mock_DMSO,New_Dongjin - 150mM_NaCl,Chilbo | -0.771368973 | 0.712044774 | 37.31043929 | -1.083315266 | 1.00E+00 |
| Mock_DMSO,New_Dongjin - 200mM_NaCl,Chilbo | -0.610178107 | 0.713351762 | 37.58768973 | -0.855367772 | 1.00E+00 |
| 1uM_GA3,New_Dongjin - 100mM_NaCl,New_Dongjin | 0.948352235 | 0.752316213 | 37.32609014 | 1.260576627 | 9.98E-01 |
| 1uM_GA3,New_Dongjin - 150mM_NaCl,New_Dongjin | 0.331405357 | 0.82012826 | 32.07135876 | 0.404089669 | 1.00E+00 |
| 1uM_GA3,New_Dongjin - 200mM_NaCl,New_Dongjin | -0.015770685 | 0.750346988 | 36.93642488 | -0.021017856 | 1.00E+00 |
| 1uM_GA3,New_Dongjin - Mock,Chucheongbyeo | 2.487493508 | 0.756708378 | 38.17219099 | 3.287255145 | 1.41E-01 |
| 1uM_GA3,New_Dongjin - Mock_DMSO,Chucheongbyeo | 2.15266917 | 0.739599105 | 34.56407942 | 2.910589205 | 2.97E-01 |
| 1uM_GA3,New_Dongjin - 1uM_GA3,Chucheongbyeo | 0.271117865 | 0.75692895 | 38.18095808 | 0.358181391 | 1 |
| 1uM_GA3,New_Dongjin - 100mM_NaCl,Chucheongbyeo | 3.124011022 | 0.752316213 | 37.32609014 | 4.152523854 | 1.73E-02 |
| 1uM_GA3,New_Dongjin - 150mM_NaCl,Chucheongbyeo | 2.520808327 | 0.811740572 | 31.05166805 | 3.105435915 | 2.14E-01 |
| 1uM_GA3,New_Dongjin - 200mM_NaCl,Chucheongbyeo | 2.10173779 | 0.752487485 | 37.32854527 | 2.793053482 | 3.58E-01 |
| 1uM_GA3,New_Dongjin - Mock,Chilbo | 1.159004343 | 0.718807061 | 38.64186558 | 1.612399774 | 9.72E-01 |
| 1uM_GA3,New_Dongjin - Mock_DMSO,Chilbo | 0.161352467 | 0.720345456 | 38.87008819 | 0.223993177 | 1.00E+00 |
| 1uM_GA3,New_Dongjin - 1uM_GA3,Chilbo | -1.438964482 | 0.716611304 | 38.23658143 | -2.008012537 | 0.847518282 |
| 1uM_GA3,New_Dongjin - 100mM_NaCl,Chilbo | 1.089187419 | 0.713357055 | 37.59020784 | 1.526847477 | 9.83E-01 |
| 1uM_GA3,New_Dongjin - 150mM_NaCl,Chilbo | 0.056628863 | 0.712044774 | 37.31043929 | 0.079529918 | 1.00E+00 |
| 1uM_GA3,New_Dongjin - 200mM_NaCl,Chilbo | 0.217819729 | 0.713351762 | 37.58768973 | 0.305346872 | 1.00E+00 |
| 100mM_NaCl,New_Dongjin - 150mM_NaCl,New_Dongjin | -0.616946878 | 0.815999832 | 31.42834776 | -0.756062506 | 0.999996626 |
| 100mM_NaCl,New_Dongjin - 200mM_NaCl,New_Dongjin | -0.96412292 | 0.746106025 | 36.11071983 | -1.292206319 | 9.97E-01 |
| 100mM_NaCl,New_Dongjin - Mock,Chucheongbyeo | 1.539141273 | 0.752495941 | 37.330397 | 2.045381497 | 0.828643492 |
| 100mM_NaCl,New_Dongjin - Mock_DMSO,Chucheongbyeo | 1.204316935 | 0.73527695 | 33.75885301 | 1.637909274 | 0.965937605 |
| 100mM_NaCl,New_Dongjin - 1uM_GA3,Chucheongbyeo | -0.67723437 | 0.752708433 | 37.33553798 | -0.899730016 | 1.00E+00 |
| 100mM_NaCl,New_Dongjin - 100mM_NaCl,Chucheongbyeo | 2.175658787 | 0.748081441 | 36.49403652 | 2.908318089 | 0.296259811 |
| 100mM_NaCl,New_Dongjin - 150mM_NaCl,Chucheongbyeo | 1.572456092 | 0.807570214 | 30.41636838 | 1.947144737 | 0.871233905 |
| 100mM_NaCl,New_Dongjin - 200mM_NaCl,Chucheongbyeo | 1.153385555 | 0.748261841 | 36.50028262 | 1.541419716 | 9.81E-01 |
| 100mM_NaCl,New_Dongjin - Mock,Chilbo | 0.210652109 | 0.714376358 | 37.70076639 | 0.294875532 | 1 |
| 100mM_NaCl,New_Dongjin - Mock_DMSO,Chilbo | -0.786999768 | 0.71593029 | 37.93050615 | -1.099268713 | 0.999541326 |
| 100mM_NaCl,New_Dongjin - 1uM_GA3,Chilbo | -2.387316717 | 0.712173543 | 37.30261509 | -3.352155863 | 1.23E-01 |
| 100mM_NaCl,New_Dongjin - 100mM_NaCl,Chilbo | 0.140835184 | 0.708890051 | 36.65922556 | 0.198669997 | 1 |
| 100mM_NaCl,New_Dongjin - 150mM_NaCl,Chilbo | -0.891723372 | 0.707567828 | 36.38202723 | -1.260265571 | 0.997604387 |
| 100mM_NaCl,New_Dongjin - 200mM_NaCl,Chilbo | -0.730532506 | 0.708890972 | 36.65966095 | -1.030528719 | 1.00E+00 |
| 150mM_NaCl,New_Dongjin - 200mM_NaCl,New_Dongjin | -0.347176042 | 0.814416585 | 31.18452297 | -0.42628803 | 1 |
| 150mM_NaCl,New_Dongjin - Mock,Chucheongbyeo | 2.156088151 | 0.819967941 | 32.03476915 | 2.629478599 | 0.462290973 |
| 150mM_NaCl,New_Dongjin - Mock_DMSO,Chucheongbyeo | 1.821263813 | 0.803728569 | 29.39683491 | 2.266018508 | 0.698628445 |
| 150mM_NaCl,New_Dongjin - 1uM_GA3,Chucheongbyeo | -0.060287492 | 0.819714697 | 31.96611871 | -0.073546921 | 1.00E+00 |
| 150mM_NaCl,New_Dongjin - 100mM_NaCl,Chucheongbyeo | 2.792605665 | 0.815999832 | 31.42834776 | 3.422311568 | 0.113513103 |
| 150mM_NaCl,New_Dongjin - 150mM_NaCl,Chucheongbyeo | 2.18940297 | 0.860414541 | 27.27096327 | 2.544590852 | 0.519887133 |
| 150mM_NaCl,New_Dongjin - 200mM_NaCl,Chucheongbyeo | 1.770332433 | 0.816563905 | 31.50403434 | 2.168026803 | 0.758732167 |
| 150mM_NaCl,New_Dongjin - Mock,Chilbo | 0.827598986 | 0.78531016 | 31.85732392 | 1.05384984 | 0.999691818 |
| 150mM_NaCl,New_Dongjin - Mock_DMSO,Chilbo | -0.17005289 | 0.786976039 | 32.05910911 | -0.216083949 | 1 |
| 150mM_NaCl,New_Dongjin - 1uM_GA3,Chilbo | -1.770369839 | 0.783627906 | 31.6345332 | -2.259197031 | 7.03E-01 |
| 150mM_NaCl,New_Dongjin - 100mM_NaCl,Chilbo | 0.757782062 | 0.780202325 | 31.09300489 | 0.971263527 | 0.999889965 |
| 150mM_NaCl,New_Dongjin - 150mM_NaCl,Chilbo | -0.274776494 | 0.778945526 | 30.89615565 | -0.352754442 | 1 |
| 150mM_NaCl,New_Dongjin - 200mM_NaCl,Chilbo | -0.113585628 | 0.780505443 | 31.14940905 | -0.145528297 | 1 |
| 200mM_NaCl,New_Dongjin - Mock,Chucheongbyeo | 2.503264193 | 0.750534801 | 36.9442515 | 3.33530729 | 0.128327767 |
| 200mM_NaCl,New_Dongjin - Mock_DMSO,Chucheongbyeo | 2.168439855 | 0.733282003 | 33.39761809 | 2.957170429 | 2.76E-01 |
| 200mM_NaCl,New_Dongjin - 1uM_GA3,Chucheongbyeo | 0.286888549 | 0.750756236 | 36.95308694 | 0.382132755 | 1.00E+00 |
| 200mM_NaCl,New_Dongjin - 100mM_NaCl,Chucheongbyeo | 3.139781706 | 0.746106025 | 36.11071983 | 4.208224572 | 0.015468769 |
| 200mM_NaCl,New_Dongjin - 150mM_NaCl,Chucheongbyeo | 2.536579011 | 0.805970408 | 30.17541779 | 3.14723591 | 0.199590551 |
| 200mM_NaCl,New_Dongjin - 200mM_NaCl,Chucheongbyeo | 2.117508475 | 0.746279602 | 36.11388624 | 2.83741974 | 0.334454437 |
| 200mM_NaCl,New_Dongjin - Mock,Chilbo | 1.174775028 | 0.712304549 | 37.26553714 | 1.649259477 | 0.964904226 |
| 200mM_NaCl,New_Dongjin - Mock_DMSO,Chilbo | 0.177123152 | 0.713856985 | 37.49155303 | 0.24812134 | 1 |
| 200mM_NaCl,New_Dongjin - 1uM_GA3,Chilbo | -1.423193798 | 0.710089396 | 36.86639495 | -2.004245953 | 8.49E-01 |
| 200mM_NaCl,New_Dongjin - 100mM_NaCl,Chilbo | 1.104958104 | 0.706803982 | 36.23007477 | 1.563316183 | 0.978009686 |
| 200mM_NaCl,New_Dongjin - 150mM_NaCl,Chilbo | 0.072399547 | 0.705479703 | 35.95531164 | 0.102624565 | 1 |
| 200mM_NaCl,New_Dongjin - 200mM_NaCl,Chilbo | 0.233590414 | 0.706799299 | 36.22786225 | 0.330490443 | 1 |
| Mock,Chucheongbyeo - Mock_DMSO,Chucheongbyeo | -0.334824338 | 0.739744073 | 34.55180921 | -0.45262186 | 0.999999999 |
| Mock,Chucheongbyeo - 1uM_GA3,Chucheongbyeo | -2.216375644 | 0.757089718 | 38.17719573 | -2.92749405 | 2.85E-01 |
| Mock,Chucheongbyeo - 100mM_NaCl,Chucheongbyeo | 0.636517513 | 0.752495941 | 37.330397 | 0.845875013 | 0.999985868 |
| Mock,Chucheongbyeo - 150mM_NaCl,Chucheongbyeo | 0.033314819 | 0.811570881 | 31.01330416 | 0.041049795 | 1 |
| Mock,Chucheongbyeo - 200mM_NaCl,Chucheongbyeo | -0.385755718 | 0.752675527 | 37.33658423 | -0.512512636 | 0.999999993 |
| Mock,Chucheongbyeo - Mock,Chilbo | -1.328489165 | 0.719003247 | 38.65012338 | -1.847681732 | 0.91377284 |
| Mock,Chucheongbyeo - Mock_DMSO,Chilbo | -2.326141041 | 0.72055371 | 38.88454971 | -3.228268773 | 0.158477628 |
| Mock,Chucheongbyeo - 1uM_GA3,Chilbo | -3.926457991 | 0.716814874 | 38.24832957 | -5.477645807 | 3.58E-04 |
| Mock,Chucheongbyeo - 100mM_NaCl,Chilbo | -1.398306089 | 0.71355428 | 37.59860206 | -1.959635208 | 0.869513716 |
| Mock,Chucheongbyeo - 150mM_NaCl,Chilbo | -2.430864646 | 0.712236907 | 37.31631498 | -3.413000115 | 0.107858883 |
| Mock,Chucheongbyeo - 200mM_NaCl,Chilbo | -2.269673779 | 0.713555558 | 37.5992297 | -3.180794757 | 0.176326292 |
| Mock_DMSO,Chucheongbyeo - 1uM_GA3,Chucheongbyeo | -1.881551306 | 0.739948853 | 34.55367426 | -2.542812652 | 5.17E-01 |
| Mock_DMSO,Chucheongbyeo - 100mM_NaCl,Chucheongbyeo | 0.971341851 | 0.73527695 | 33.75885301 | 1.321055762 | 0.995793612 |
| Mock_DMSO,Chucheongbyeo - 150mM_NaCl,Chucheongbyeo | 0.368139157 | 0.795134961 | 28.40137835 | 0.462989523 | 0.999999998 |
| Mock_DMSO,Chucheongbyeo - 200mM_NaCl,Chucheongbyeo | -0.05093138 | 0.735468524 | 33.76924317 | -0.069250252 | 1 |
| Mock_DMSO,Chucheongbyeo - Mock,Chilbo | -0.993664827 | 0.700980518 | 34.6176481 | -1.417535582 | 0.991308123 |
| Mock_DMSO,Chucheongbyeo - Mock_DMSO,Chilbo | -1.991316703 | 0.702595418 | 34.85646446 | -2.834229559 | 0.337571806 |
| Mock_DMSO,Chucheongbyeo - 1uM_GA3,Chilbo | -3.591633653 | 0.698742087 | 34.23780162 | -5.140142149 | 1.26E-03 |
| Mock_DMSO,Chucheongbyeo - 100mM_NaCl,Chilbo | -1.063481751 | 0.695393662 | 33.62109733 | -1.529323332 | 0.981559412 |
| Mock_DMSO,Chucheongbyeo - 150mM_NaCl,Chilbo | -2.096040308 | 0.694028997 | 33.3484659 | -3.020104802 | 0.246627497 |
| Mock_DMSO,Chucheongbyeo - 200mM_NaCl,Chilbo | -1.934849441 | 0.69540149 | 33.62451172 | -2.782348712 | 0.368155368 |
| 1uM_GA3,Chucheongbyeo - 100mM_NaCl,Chucheongbyeo | 2.852893157 | 0.752708433 | 37.33553798 | 3.790170314 | 4.43E-02 |
| 1uM_GA3,Chucheongbyeo - 150mM_NaCl,Chucheongbyeo | 2.249690462 | 0.811323677 | 30.94844065 | 2.772864304 | 3.77E-01 |
| 1uM_GA3,Chucheongbyeo - 200mM_NaCl,Chucheongbyeo | 1.830619925 | 0.752904852 | 37.34885651 | 2.431409387 | 5.91E-01 |
| 1uM_GA3,Chucheongbyeo - Mock,Chilbo | 0.887886479 | 0.719227076 | 38.65585285 | 1.234500909 | 9.98E-01 |
| 1uM_GA3,Chucheongbyeo - Mock_DMSO,Chilbo | -0.109765398 | 0.720784639 | 38.89383312 | -0.152285983 | 1.00E+00 |
| 1uM_GA3,Chucheongbyeo - 1uM_GA3,Chilbo | -1.710082347 | 0.717053024 | 38.26061384 | -2.384875719 | 0.621871826 |
| 1uM_GA3,Chucheongbyeo - 100mM_NaCl,Chilbo | 0.818069555 | 0.713773785 | 37.60199861 | 1.146118801 | 9.99E-01 |
| 1uM_GA3,Chucheongbyeo - 150mM_NaCl,Chilbo | -0.214489002 | 0.712456279 | 37.31965609 | -0.301055669 | 1.00E+00 |
| 1uM_GA3,Chucheongbyeo - 200mM_NaCl,Chilbo | -0.053298136 | 0.713787847 | 37.60853143 | -0.074669436 | 1.00E+00 |
| 100mM_NaCl,Chucheongbyeo - 150mM_NaCl,Chucheongbyeo | -0.603202695 | 0.807570214 | 30.41636838 | -0.746935293 | 0.999997066 |
| 100mM_NaCl,Chucheongbyeo - 200mM_NaCl,Chucheongbyeo | -1.022273232 | 0.748261841 | 36.50028262 | -1.366197199 | 0.99421638 |
| 100mM_NaCl,Chucheongbyeo - Mock,Chilbo | -1.965006678 | 0.714376358 | 37.70076639 | -2.750660288 | 0.382860312 |
| 100mM_NaCl,Chucheongbyeo - Mock_DMSO,Chilbo | -2.962658555 | 0.71593029 | 37.93050615 | -4.138194175 | 0.017746698 |
| 100mM_NaCl,Chucheongbyeo - 1uM_GA3,Chilbo | -4.562975504 | 0.712173543 | 37.30261509 | -6.407111792 | 2.26E-05 |
| 100mM_NaCl,Chucheongbyeo - 100mM_NaCl,Chilbo | -2.034823603 | 0.708890051 | 36.65922556 | -2.870436113 | 0.315935924 |
| 100mM_NaCl,Chucheongbyeo - 150mM_NaCl,Chilbo | -3.067382159 | 0.707567828 | 36.38202723 | -4.335106881 | 0.010896232 |
| 100mM_NaCl,Chucheongbyeo - 200mM_NaCl,Chilbo | -2.906191293 | 0.708890972 | 36.65966095 | -4.09963084 | 0.02031725 |
| 150mM_NaCl,Chucheongbyeo - 200mM_NaCl,Chucheongbyeo | -0.419070537 | 0.808131626 | 30.48886526 | -0.518567178 | 0.999999989 |
| 150mM_NaCl,Chucheongbyeo - Mock,Chilbo | -1.361803983 | 0.776555728 | 30.75451707 | -1.753646178 | 0.938954794 |
| 150mM_NaCl,Chucheongbyeo - Mock_DMSO,Chilbo | -2.35945586 | 0.778247186 | 30.95697641 | -3.031756366 | 0.245154072 |
| 150mM_NaCl,Chucheongbyeo - 1uM_GA3,Chilbo | -3.959772809 | 0.774847655 | 30.53165494 | -5.110388843 | 1.76E-03 |
| 150mM_NaCl,Chucheongbyeo - 100mM_NaCl,Chilbo | -1.431620908 | 0.771396043 | 30.00088077 | -1.855883136 | 0.906734035 |
| 150mM_NaCl,Chucheongbyeo - 150mM_NaCl,Chilbo | -2.464179464 | 0.770118823 | 29.80466087 | -3.199739302 | 0.181319954 |
| 150mM_NaCl,Chucheongbyeo - 200mM_NaCl,Chilbo | -2.302988598 | 0.771696486 | 30.05452146 | -2.984319145 | 0.26803896 |
| 200mM_NaCl,Chucheongbyeo - Mock,Chilbo | -0.942733447 | 0.714564458 | 37.70693632 | -1.319311975 | 0.996090707 |
| 200mM_NaCl,Chucheongbyeo - Mock_DMSO,Chilbo | -1.940385323 | 0.716111755 | 37.93339191 | -2.709612446 | 0.407603572 |
| 200mM_NaCl,Chucheongbyeo - 1uM_GA3,Chilbo | -3.540702272 | 0.71234998 | 37.30312156 | -4.970453246 | 1.73E-03 |
| 200mM_NaCl,Chucheongbyeo - 100mM_NaCl,Chilbo | -1.012550371 | 0.709085193 | 36.66830643 | -1.427967162 | 0.990877856 |
| 200mM_NaCl,Chucheongbyeo - 150mM_NaCl,Chilbo | -2.045108927 | 0.707763476 | 36.39128157 | -2.889537251 | 0.306116997 |
| 200mM_NaCl,Chucheongbyeo - 200mM_NaCl,Chilbo | -1.883918061 | 0.709074649 | 36.66337634 | -2.656868447 | 0.441531194 |
| Mock,Chilbo - Mock_DMSO,Chilbo | -0.997651876 | 0.667112104 | 40.40248775 | -1.495478601 | 0.986108092 |
| Mock,Chilbo - 1uM_GA3,Chilbo | -2.597968826 | 0.661248844 | 39.34446053 | -3.928882218 | 2.99E-02 |
| Mock,Chilbo - 100mM_NaCl,Chilbo | -0.069816924 | 0.656920082 | 38.40110897 | -0.106279175 | 1 |
| Mock,Chilbo - 150mM_NaCl,Chilbo | -1.102375481 | 0.655629603 | 38.093919 | -1.681399796 | 0.95887911 |
| Mock,Chilbo - 200mM_NaCl,Chilbo | -0.941184614 | 0.656920261 | 38.40122864 | -1.432722768 | 0.990751378 |
| Mock_DMSO,Chilbo - 1uM_GA3,Chilbo | -1.600316949 | 0.663637599 | 39.73412634 | -2.411432011 | 6.04E-01 |
| Mock_DMSO,Chilbo - 100mM_NaCl,Chilbo | 0.927834952 | 0.659363079 | 38.80613411 | 1.407168495 | 0.992343639 |
| Mock_DMSO,Chilbo - 150mM_NaCl,Chilbo | -0.104723604 | 0.658076555 | 38.49803456 | -0.159135899 | 1 |
| Mock_DMSO,Chilbo - 200mM_NaCl,Chilbo | 0.056467262 | 0.659358195 | 38.80333799 | 0.085639736 | 1 |
| 1uM_GA3,Chilbo - 100mM_NaCl,Chilbo | 2.528151901 | 0.653207862 | 37.6673469 | 3.870363552 | 3.60E-02 |
| 1uM_GA3,Chilbo - 150mM_NaCl,Chilbo | 1.495593345 | 0.65191968 | 37.36712745 | 2.29413744 | 6.82E-01 |
| 1uM_GA3,Chilbo - 200mM_NaCl,Chilbo | 1.656784211 | 0.653198473 | 37.66232392 | 2.536417766 | 5.20E-01 |
| 100mM_NaCl,Chilbo - 150mM_NaCl,Chilbo | -1.032558556 | 0.647432701 | 36.39929123 | -1.594850792 | 0.973682039 |
| 100mM_NaCl,Chilbo - 200mM_NaCl,Chilbo | -0.87136769 | 0.648726806 | 36.69451418 | -1.343196676 | 0.995185799 |
| 150mM_NaCl,Chilbo - 200mM_NaCl,Chilbo | 0.161190866 | 0.647437444 | 36.40176074 | 0.248967476 | 1 |

Leaf 1 Length (mm)

| contrast | estimate | SE | df | t.ratio | p.value |
| --- | --- | --- | --- | --- | --- |
| Mock,New_Dongjin - Mock_DMSO,New_Dongjin | -0.908277966 | 0.885647387 | 35.8206884 | -1.025552584 | 0.999801739 |
| Mock,New_Dongjin - 1uM_GA3,New_Dongjin | -6.963021684 | 0.885647387 | 35.8206884 | -7.862069919 | 3.62E-07 |
| Mock,New_Dongjin - 100mM_NaCl,New_Dongjin | 0.350268474 | 0.8826254 | 35.33568628 | 0.396848396 | 1 |
| Mock,New_Dongjin - 150mM_NaCl,New_Dongjin | 2.25424453 | 0.967749021 | 32.36031695 | 2.32936896 | 0.658664585 |
| Mock,New_Dongjin - 200mM_NaCl,New_Dongjin | 2.637314954 | 0.881216602 | 35.11086306 | 2.992811243 | 2.57E-01 |
| Mock,New_Dongjin - Mock,Chucheongbyeo | 1.613215981 | 0.885810037 | 35.83561011 | 1.821176001 | 0.921422963 |
| Mock,New_Dongjin - Mock_DMSO,Chucheongbyeo | 1.471867974 | 0.873835749 | 33.84204356 | 1.684376012 | 0.956899903 |
| Mock,New_Dongjin - 1uM_GA3,Chucheongbyeo | -4.064596966 | 0.886001269 | 35.851134 | -4.587574655 | 5.54E-03 |
| Mock,New_Dongjin - 100mM_NaCl,Chucheongbyeo | 1.470757774 | 0.8826254 | 35.33568628 | 1.666344265 | 0.961090329 |
| Mock,New_Dongjin - 150mM_NaCl,Chucheongbyeo | 2.408046377 | 0.961400982 | 31.63413537 | 2.504726356 | 0.54325991 |
| Mock,New_Dongjin - 200mM_NaCl,Chucheongbyeo | 3.374486637 | 0.882784437 | 35.34949318 | 3.822548852 | 4.24E-02 |
| Mock,New_Dongjin - Mock,Chilbo | 0.6334984 | 0.884048126 | 35.56724475 | 0.716588137 | 0.999998656 |
| Mock,New_Dongjin - Mock_DMSO,Chilbo | 1.250098197 | 0.884208857 | 35.5815772 | 1.413804201 | 0.991644924 |
| Mock,New_Dongjin - 1uM_GA3,Chilbo | -6.654873738 | 0.884208857 | 35.5815772 | -7.526359507 | 1.00E-06 |
| Mock,New_Dongjin - 100mM_NaCl,Chilbo | -0.143813736 | 0.8826254 | 35.33568628 | -0.162938588 | 1 |
| Mock,New_Dongjin - 150mM_NaCl,Chilbo | 1.498155935 | 0.881216602 | 35.11086306 | 1.700099535 | 9.54E-01 |
| Mock,New_Dongjin - 200mM_NaCl,Chilbo | 2.303156732 | 0.8826254 | 35.33568628 | 2.609438536 | 4.73E-01 |
| Mock_DMSO,New_Dongjin - 1uM_GA3,New_Dongjin | -6.054743718 | 0.880513424 | 34.99934582 | -6.87637866 | 7.36E-06 |
| Mock_DMSO,New_Dongjin - 100mM_NaCl,New_Dongjin | 1.258546441 | 0.877473756 | 34.51965493 | 1.434283855 | 0.990202039 |
| Mock_DMSO,New_Dongjin - 150mM_NaCl,New_Dongjin | 3.162522497 | 0.963052834 | 31.73644367 | 3.283851502 | 0.150595993 |
| Mock_DMSO,New_Dongjin - 200mM_NaCl,New_Dongjin | 3.54559292 | 0.876056673 | 34.29731321 | 4.047218668 | 2.48E-02 |
| Mock_DMSO,New_Dongjin - Mock,Chucheongbyeo | 2.521493948 | 0.880677022 | 35.0142203 | 2.863131302 | 0.321672706 |
| Mock_DMSO,New_Dongjin - Mock_DMSO,Chucheongbyeo | 2.38014594 | 0.868631977 | 33.04380506 | 2.740108589 | 0.393446079 |
| Mock_DMSO,New_Dongjin - 1uM_GA3,Chucheongbyeo | -3.156318999 | 0.880869368 | 35.02973244 | -3.583186241 | 7.55E-02 |
| Mock_DMSO,New_Dongjin - 100mM_NaCl,Chucheongbyeo | 2.379035741 | 0.877473756 | 34.51965493 | 2.711232929 | 0.409399032 |
| Mock_DMSO,New_Dongjin - 150mM_NaCl,Chucheongbyeo | 3.316324344 | 0.956673634 | 31.01603862 | 3.466515879 | 0.103941403 |
| Mock_DMSO,New_Dongjin - 200mM_NaCl,Chucheongbyeo | 4.282764603 | 0.877633726 | 34.53342936 | 4.879899753 | 2.59E-03 |
| Mock_DMSO,New_Dongjin - Mock,Chilbo | 1.541776366 | 0.878904821 | 34.74863271 | 1.75420174 | 0.940599459 |
| Mock_DMSO,New_Dongjin - Mock_DMSO,Chilbo | 2.158376163 | 0.879066492 | 34.7629257 | 2.455304783 | 0.575026854 |
| Mock_DMSO,New_Dongjin - 1uM_GA3,Chilbo | -5.746595772 | 0.879066492 | 34.7629257 | -6.537157112 | 2.06E-05 |
| Mock_DMSO,New_Dongjin - 100mM_NaCl,Chilbo | 0.76446423 | 0.877473756 | 34.51965493 | 0.871210364 | 0.99997704 |
| Mock_DMSO,New_Dongjin - 150mM_NaCl,Chilbo | 2.406433901 | 0.876056673 | 34.29731321 | 2.746892953 | 3.88E-01 |
| Mock_DMSO,New_Dongjin - 200mM_NaCl,Chilbo | 3.211434698 | 0.877473756 | 34.51965493 | 3.659864102 | 6.37E-02 |
| 1uM_GA3,New_Dongjin - 100mM_NaCl,New_Dongjin | 7.313290158 | 0.877473756 | 34.51965493 | 8.334483067 | 1.23E-07 |
| 1uM_GA3,New_Dongjin - 150mM_NaCl,New_Dongjin | 9.217266214 | 0.963052834 | 31.73644367 | 9.570883222 | 9.98E-09 |
| 1uM_GA3,New_Dongjin - 200mM_NaCl,New_Dongjin | 9.600336638 | 0.876056673 | 34.29731321 | 10.95857943 | 1.38E-10 |
| 1uM_GA3,New_Dongjin - Mock,Chucheongbyeo | 8.576237665 | 0.880677022 | 35.0142203 | 9.738232581 | 2.41E-09 |
| 1uM_GA3,New_Dongjin - Mock_DMSO,Chucheongbyeo | 8.434889658 | 0.868631977 | 33.04380506 | 9.710544721 | 4.70E-09 |
| 1uM_GA3,New_Dongjin - 1uM_GA3,Chucheongbyeo | 2.898424718 | 0.880869368 | 35.02973244 | 3.290413794 | 0.14371331 |
| 1uM_GA3,New_Dongjin - 100mM_NaCl,Chucheongbyeo | 8.433779458 | 0.877473756 | 34.51965493 | 9.611432142 | 3.89E-09 |
| 1uM_GA3,New_Dongjin - 150mM_NaCl,Chucheongbyeo | 9.371068061 | 0.956673634 | 31.01603862 | 9.795470188 | 7.28E-09 |
| 1uM_GA3,New_Dongjin - 200mM_NaCl,Chucheongbyeo | 10.33750832 | 0.877633726 | 34.53342936 | 11.77884123 | 1.74E-11 |
| 1uM_GA3,New_Dongjin - Mock,Chilbo | 7.596520084 | 0.878904821 | 34.74863271 | 8.643165795 | 4.98E-08 |
| 1uM_GA3,New_Dongjin - Mock_DMSO,Chilbo | 8.213119881 | 0.879066492 | 34.7629257 | 9.34300187 | 7.41E-09 |
| 1uM_GA3,New_Dongjin - 1uM_GA3,Chilbo | 0.308147946 | 0.879066492 | 34.7629257 | 0.350539975 | 1 |
| 1uM_GA3,New_Dongjin - 100mM_NaCl,Chilbo | 6.819207948 | 0.877473756 | 34.51965493 | 7.771409577 | 6.00E-07 |
| 1uM_GA3,New_Dongjin - 150mM_NaCl,Chilbo | 8.461177619 | 0.876056673 | 34.29731321 | 9.65825372 | 3.68E-09 |
| 1uM_GA3,New_Dongjin - 200mM_NaCl,Chilbo | 9.266178416 | 0.877473756 | 34.51965493 | 10.56006331 | 3.41E-10 |
| 100mM_NaCl,New_Dongjin - 150mM_NaCl,New_Dongjin | 1.903976056 | 0.960274473 | 31.37158831 | 1.982741507 | 0.856009413 |
| 100mM_NaCl,New_Dongjin - 200mM_NaCl,New_Dongjin | 2.287046479 | 0.873001487 | 33.8222261 | 2.619750955 | 4.67E-01 |
| 100mM_NaCl,New_Dongjin - Mock,Chucheongbyeo | 1.262947507 | 0.87763792 | 34.53450012 | 1.43903024 | 0.989874543 |
| 100mM_NaCl,New_Dongjin - Mock_DMSO,Chucheongbyeo | 1.1215995 | 0.865550584 | 32.57774896 | 1.295822013 | 0.996519377 |
| 100mM_NaCl,New_Dongjin - 1uM_GA3,Chucheongbyeo | -4.41486544 | 0.877830932 | 34.55000343 | -5.029288987 | 1.70E-03 |
| 100mM_NaCl,New_Dongjin - 100mM_NaCl,Chucheongbyeo | 1.1204893 | 0.874423521 | 34.04310186 | 1.281403431 | 0.997000911 |
| 100mM_NaCl,New_Dongjin - 150mM_NaCl,Chucheongbyeo | 2.057777903 | 0.953876692 | 30.65460304 | 2.157278734 | 0.764620037 |
| 100mM_NaCl,New_Dongjin - 200mM_NaCl,Chucheongbyeo | 3.024218162 | 0.874584049 | 34.05685568 | 3.457893115 | 1.01E-01 |
| 100mM_NaCl,New_Dongjin - Mock,Chilbo | 0.283229925 | 0.87585957 | 34.27055547 | 0.323373672 | 1 |
| 100mM_NaCl,New_Dongjin - Mock_DMSO,Chilbo | 0.899829723 | 0.876021803 | 34.28482374 | 1.027177314 | 0.999790503 |
| 100mM_NaCl,New_Dongjin - 1uM_GA3,Chilbo | -7.005142213 | 0.876021803 | 34.28482374 | -7.996538654 | 3.33E-07 |
| 100mM_NaCl,New_Dongjin - 100mM_NaCl,Chilbo | -0.494082211 | 0.874423521 | 34.04310186 | -0.565037649 | 0.999999962 |
| 100mM_NaCl,New_Dongjin - 150mM_NaCl,Chilbo | 1.14788746 | 0.873001487 | 33.8222261 | 1.314874577 | 0.996005935 |
| 100mM_NaCl,New_Dongjin - 200mM_NaCl,Chilbo | 1.952888258 | 0.874423521 | 34.04310186 | 2.233343696 | 7.20E-01 |
| 150mM_NaCl,New_Dongjin - 200mM_NaCl,New_Dongjin | 0.383070423 | 0.958979753 | 31.2023597 | 0.399456216 | 1 |
| 150mM_NaCl,New_Dongjin - Mock,Chucheongbyeo | -0.641028549 | 0.963202413 | 31.74811048 | -0.665518006 | 0.999999494 |
| 150mM_NaCl,New_Dongjin - Mock_DMSO,Chucheongbyeo | -0.782376556 | 0.952201861 | 30.24980511 | -0.821649892 | 0.999988437 |
| 150mM_NaCl,New_Dongjin - 1uM_GA3,Chucheongbyeo | -6.318841496 | 0.963378283 | 31.7603931 | -6.559044988 | 2.87E-05 |
| 150mM_NaCl,New_Dongjin - 100mM_NaCl,Chucheongbyeo | -0.783486756 | 0.960274473 | 31.37158831 | -0.815898764 | 0.999989928 |
| 150mM_NaCl,New_Dongjin - 150mM_NaCl,Chucheongbyeo | 0.153801847 | 1.033146318 | 29.078751 | 0.14886744 | 1 |
| 150mM_NaCl,New_Dongjin - 200mM_NaCl,Chucheongbyeo | 1.120242106 | 0.960420652 | 31.38243107 | 1.166407765 | 0.998923138 |
| 150mM_NaCl,New_Dongjin - Mock,Chilbo | -1.620746131 | 0.961582319 | 31.54567589 | -1.685499098 | 0.955780112 |
| 150mM_NaCl,New_Dongjin - Mock_DMSO,Chilbo | -1.004146333 | 0.961730092 | 31.55690721 | -1.044104101 | 0.999723798 |
| 150mM_NaCl,New_Dongjin - 1uM_GA3,Chilbo | -8.909118269 | 0.961730092 | 31.55690721 | -9.263636796 | 2.26E-08 |
| 150mM_NaCl,New_Dongjin - 100mM_NaCl,Chilbo | -2.398058267 | 0.960274473 | 31.37158831 | -2.49726337 | 0.548281123 |
| 150mM_NaCl,New_Dongjin - 150mM_NaCl,Chilbo | -0.756088596 | 0.958979753 | 31.2023597 | -0.788430197 | 0.999993778 |
| 150mM_NaCl,New_Dongjin - 200mM_NaCl,Chilbo | 0.048912202 | 0.960274473 | 31.37158831 | 0.050935647 | 1 |
| 200mM_NaCl,New_Dongjin - Mock,Chucheongbyeo | -1.024098972 | 0.876221103 | 34.31214431 | -1.168767757 | 0.998963569 |
| 200mM_NaCl,New_Dongjin - Mock_DMSO,Chucheongbyeo | -1.16544698 | 0.864113948 | 32.36177273 | -1.348719092 | 9.95E-01 |
| 200mM_NaCl,New_Dongjin - 1uM_GA3,Chucheongbyeo | -6.701911919 | 0.876414427 | 34.32764295 | -7.646966679 | 8.85E-07 |
| 200mM_NaCl,New_Dongjin - 100mM_NaCl,Chucheongbyeo | -1.166557179 | 0.873001487 | 33.8222261 | -1.336260244 | 0.995247979 |
| 200mM_NaCl,New_Dongjin - 150mM_NaCl,Chucheongbyeo | -0.229268576 | 0.952573276 | 30.48697405 | -0.240683402 | 1 |
| 200mM_NaCl,New_Dongjin - 200mM_NaCl,Chucheongbyeo | 0.737171683 | 0.873162277 | 33.83596986 | 0.844255075 | 0.999984906 |
| 200mM_NaCl,New_Dongjin - Mock,Chilbo | -2.003816554 | 0.874439871 | 34.04896778 | -2.291542987 | 0.683304109 |
| 200mM_NaCl,New_Dongjin - Mock_DMSO,Chilbo | -1.387216757 | 0.874602368 | 34.06322409 | -1.586111366 | 0.974383444 |
| 200mM_NaCl,New_Dongjin - 1uM_GA3,Chilbo | -9.292188692 | 0.874602368 | 34.06322409 | -10.62447237 | 3.41E-10 |
| 200mM_NaCl,New_Dongjin - 100mM_NaCl,Chilbo | -2.78112869 | 0.873001487 | 33.8222261 | -3.185708996 | 0.179636505 |
| 200mM_NaCl,New_Dongjin - 150mM_NaCl,Chilbo | -1.139159019 | 0.871577133 | 33.60203517 | -1.307008842 | 0.996244396 |
| 200mM_NaCl,New_Dongjin - 200mM_NaCl,Chilbo | -0.334158222 | 0.873001487 | 33.8222261 | -0.382769361 | 1 |
| Mock,Chucheongbyeo - Mock_DMSO,Chucheongbyeo | -0.141348007 | 0.868797812 | 33.05853068 | -0.162693788 | 1 |
| Mock,Chucheongbyeo - 1uM_GA3,Chucheongbyeo | -5.677812947 | 0.8810329 | 35.04460376 | -6.444495943 | 2.61E-05 |
| Mock,Chucheongbyeo - 100mM_NaCl,Chucheongbyeo | -0.142458207 | 0.87763792 | 34.53450012 | -0.162320023 | 1 |
| Mock,Chucheongbyeo - 150mM_NaCl,Chucheongbyeo | 0.794830396 | 0.95682421 | 31.02767737 | 0.830696368 | 0.999986871 |
| Mock,Chucheongbyeo - 200mM_NaCl,Chucheongbyeo | 1.761270655 | 0.877797861 | 34.54827323 | 2.006464967 | 0.846604052 |
| Mock,Chucheongbyeo - Mock,Chilbo | -0.979717582 | 0.879068718 | 34.76349275 | -1.114494876 | 0.999421827 |
| Mock,Chucheongbyeo - Mock_DMSO,Chilbo | -0.363117784 | 0.879230359 | 34.77778447 | -0.412995048 | 1 |
| Mock,Chucheongbyeo - 1uM_GA3,Chilbo | -8.26808972 | 0.879230359 | 34.77778447 | -9.403780969 | 6.27E-09 |
| Mock,Chucheongbyeo - 100mM_NaCl,Chilbo | -1.757029718 | 0.87763792 | 34.53450012 | -2.001998407 | 0.848724065 |
| Mock,Chucheongbyeo - 150mM_NaCl,Chilbo | -0.115060047 | 0.876221103 | 34.31214431 | -0.131313941 | 1 |
| Mock,Chucheongbyeo - 200mM_NaCl,Chilbo | 0.689940751 | 0.87763792 | 34.53450012 | 0.786133706 | 0.999994637 |
| Mock_DMSO,Chucheongbyeo - 1uM_GA3,Chucheongbyeo | -5.53646494 | 0.868992788 | 33.07397507 | -6.371128758 | 4.10E-05 |
| Mock_DMSO,Chucheongbyeo - 100mM_NaCl,Chucheongbyeo | -0.0011102 | 0.865550584 | 32.57774896 | -0.001282652 | 1 |
| Mock_DMSO,Chucheongbyeo - 150mM_NaCl,Chucheongbyeo | 0.936178403 | 0.945749472 | 29.54434061 | 0.989879911 | 0.999852245 |
| Mock_DMSO,Chucheongbyeo - 200mM_NaCl,Chucheongbyeo | 1.902618663 | 0.865712758 | 32.59141168 | 2.197748209 | 0.741492085 |
| Mock_DMSO,Chucheongbyeo - Mock,Chilbo | -0.838369575 | 0.86700133 | 32.80011246 | -0.966976112 | 0.999901065 |
| Mock_DMSO,Chucheongbyeo - Mock_DMSO,Chilbo | -0.221769777 | 0.86716522 | 32.81427616 | -0.255741088 | 1 |
| Mock_DMSO,Chucheongbyeo - 1uM_GA3,Chilbo | -8.126741712 | 0.86716522 | 32.81427616 | -9.371618604 | 1.19E-08 |
| Mock_DMSO,Chucheongbyeo - 100mM_NaCl,Chilbo | -1.615681711 | 0.865550584 | 32.57774896 | -1.866651979 | 0.904345421 |
| Mock_DMSO,Chucheongbyeo - 150mM_NaCl,Chilbo | 0.026287961 | 0.864113948 | 32.36177273 | 0.030421868 | 1 |
| Mock_DMSO,Chucheongbyeo - 200mM_NaCl,Chilbo | 0.831288758 | 0.865550584 | 32.57774896 | 0.960416148 | 0.999909042 |
| 1uM_GA3,Chucheongbyeo - 100mM_NaCl,Chucheongbyeo | 5.53535474 | 0.877830932 | 34.55000343 | 6.305718489 | 4.16E-05 |
| 1uM_GA3,Chucheongbyeo - 150mM_NaCl,Chucheongbyeo | 6.472643343 | 0.957001252 | 31.0399556 | 6.763463821 | 1.81E-05 |
| 1uM_GA3,Chucheongbyeo - 200mM_NaCl,Chucheongbyeo | 7.439083602 | 0.877990838 | 34.56377453 | 8.472848786 | 8.32E-08 |
| 1uM_GA3,Chucheongbyeo - Mock,Chilbo | 4.698095365 | 0.879261416 | 34.7790012 | 5.343229306 | 6.79E-04 |
| 1uM_GA3,Chucheongbyeo - Mock_DMSO,Chilbo | 5.314695163 | 0.879423022 | 34.79329095 | 6.043388711 | 8.75E-05 |
| 1uM_GA3,Chucheongbyeo - 1uM_GA3,Chilbo | -2.590276773 | 0.879423022 | 34.79329095 | -2.945427523 | 0.279666867 |
| 1uM_GA3,Chucheongbyeo - 100mM_NaCl,Chilbo | 3.920783229 | 0.877830932 | 34.55000343 | 4.466444603 | 8.14E-03 |
| 1uM_GA3,Chucheongbyeo - 150mM_NaCl,Chilbo | 5.5627529 | 0.876414427 | 34.32764295 | 6.34717176 | 3.78E-05 |
| 1uM_GA3,Chucheongbyeo - 200mM_NaCl,Chilbo | 6.367753698 | 0.877830932 | 34.55000343 | 7.253963678 | 2.63E-06 |
| 100mM_NaCl,Chucheongbyeo - 150mM_NaCl,Chucheongbyeo | 0.937288603 | 0.953876692 | 30.65460304 | 0.982609818 | 0.999870221 |
| 100mM_NaCl,Chucheongbyeo - 200mM_NaCl,Chucheongbyeo | 1.903728863 | 0.874584049 | 34.05685568 | 2.176724883 | 0.754563539 |
| 100mM_NaCl,Chucheongbyeo - Mock,Chilbo | -0.837259375 | 0.87585957 | 34.27055547 | -0.955928785 | 0.999918266 |
| 100mM_NaCl,Chucheongbyeo - Mock_DMSO,Chilbo | -0.220659577 | 0.876021803 | 34.28482374 | -0.251888225 | 1 |
| 100mM_NaCl,Chucheongbyeo - 1uM_GA3,Chilbo | -8.125631513 | 0.876021803 | 34.28482374 | -9.275604193 | 1.01E-08 |
| 100mM_NaCl,Chucheongbyeo - 100mM_NaCl,Chilbo | -1.614571511 | 0.874423521 | 34.04310186 | -1.84644108 | 0.912214258 |
| 100mM_NaCl,Chucheongbyeo - 150mM_NaCl,Chilbo | 0.027398161 | 0.873001487 | 33.8222261 | 0.031383865 | 1 |
| 100mM_NaCl,Chucheongbyeo - 200mM_NaCl,Chilbo | 0.832398958 | 0.874423521 | 34.04310186 | 0.951940265 | 0.99992225 |
| 150mM_NaCl,Chucheongbyeo - 200mM_NaCl,Chucheongbyeo | 0.966440259 | 0.954023852 | 30.66542738 | 1.013014777 | 0.999807496 |
| 150mM_NaCl,Chucheongbyeo - Mock,Chilbo | -1.774547978 | 0.955193298 | 30.82702752 | -1.857789394 | 0.906548801 |
| 150mM_NaCl,Chucheongbyeo - Mock_DMSO,Chilbo | -1.15794818 | 0.955342059 | 30.83823585 | -1.212077045 | 0.998295415 |
| 150mM_NaCl,Chucheongbyeo - 1uM_GA3,Chilbo | -9.062920116 | 0.955342059 | 30.83823585 | -9.486570834 | 1.63E-08 |
| 150mM_NaCl,Chucheongbyeo - 100mM_NaCl,Chilbo | -2.551860114 | 0.953876692 | 30.65460304 | -2.675251565 | 0.4349928 |
| 150mM_NaCl,Chucheongbyeo - 150mM_NaCl,Chilbo | -0.909890443 | 0.952573276 | 30.48697405 | -0.955192073 | 0.999910069 |
| 150mM_NaCl,Chucheongbyeo - 200mM_NaCl,Chilbo | -0.104889645 | 0.953876692 | 30.65460304 | -0.10996143 | 1 |
| 200mM_NaCl,Chucheongbyeo - Mock,Chilbo | -2.740988237 | 0.876019835 | 34.28431995 | -3.128911159 | 0.199985694 |
| 200mM_NaCl,Chucheongbyeo - Mock_DMSO,Chilbo | -2.12438844 | 0.876182039 | 34.29858685 | -2.42459711 | 0.595663597 |
| 200mM_NaCl,Chucheongbyeo - 1uM_GA3,Chilbo | -10.02936038 | 0.876182039 | 34.29858685 | -11.44666283 | 4.23E-11 |
| 200mM_NaCl,Chucheongbyeo - 100mM_NaCl,Chilbo | -3.518300373 | 0.874584049 | 34.05685568 | -4.022827052 | 0.026520644 |
| 200mM_NaCl,Chucheongbyeo - 150mM_NaCl,Chilbo | -1.876330702 | 0.873162277 | 33.83596986 | -2.148891165 | 0.77078106 |
| 200mM_NaCl,Chucheongbyeo - 200mM_NaCl,Chilbo | -1.071329905 | 0.874584049 | 34.05685568 | -1.224959345 | 0.998196882 |
| Mock,Chilbo - Mock_DMSO,Chilbo | 0.616599797 | 0.877455236 | 34.51303008 | 0.702713679 | 0.999998962 |
| Mock,Chilbo - 1uM_GA3,Chilbo | -7.288372138 | 0.877455236 | 34.51303008 | -8.306260918 | 1.34E-07 |
| Mock,Chilbo - 100mM_NaCl,Chilbo | -0.777312136 | 0.87585957 | 34.27055547 | -0.887484892 | 0.999970143 |
| Mock,Chilbo - 150mM_NaCl,Chilbo | 0.864657535 | 0.874439871 | 34.04896778 | 0.988813026 | 0.99987147 |
| Mock,Chilbo - 200mM_NaCl,Chilbo | 1.669658333 | 0.87585957 | 34.27055547 | 1.90630826 | 0.890221286 |
| Mock_DMSO,Chilbo - 1uM_GA3,Chilbo | -7.904971935 | 0.877617175 | 34.52730967 | -9.007312254 | 1.94E-08 |
| Mock_DMSO,Chilbo - 100mM_NaCl,Chilbo | -1.393911933 | 0.876021803 | 34.28482374 | -1.591184064 | 0.973699906 |
| Mock_DMSO,Chilbo - 150mM_NaCl,Chilbo | 0.248057738 | 0.874602368 | 34.06322409 | 0.283623446 | 1 |
| Mock_DMSO,Chilbo - 200mM_NaCl,Chilbo | 1.053058535 | 0.876021803 | 34.28482374 | 1.202091696 | 0.998556276 |
| 1uM_GA3,Chilbo - 100mM_NaCl,Chilbo | 6.511060002 | 0.876021803 | 34.28482374 | 7.432531904 | 1.64E-06 |
| 1uM_GA3,Chilbo - 150mM_NaCl,Chilbo | 8.153029673 | 0.874602368 | 34.06322409 | 9.321984449 | 9.50E-09 |
| 1uM_GA3,Chilbo - 200mM_NaCl,Chilbo | 8.95803047 | 0.876021803 | 34.28482374 | 10.22580766 | 8.57E-10 |
| 100mM_NaCl,Chilbo - 150mM_NaCl,Chilbo | 1.641969671 | 0.873001487 | 33.8222261 | 1.880832617 | 0.899779236 |
| 100mM_NaCl,Chilbo - 200mM_NaCl,Chilbo | 2.446970469 | 0.874423521 | 34.04310186 | 2.798381345 | 0.358536828 |
| 150mM_NaCl,Chilbo - 200mM_NaCl,Chilbo | 0.805000797 | 0.873001487 | 33.8222261 | 0.922107018 | 0.999949033 |

Leaf 2 Length (mm)

| contrast | estimate | SE | df | t.ratio | p.value |
| --- | --- | --- | --- | --- | --- |
| Mock,New_Dongjin - Mock_DMSO,New_Dongjin | -0.156629935 | 0.677027366 | 746 | -0.231349488 | 1 |
| Mock,New_Dongjin - 1uM_GA3,New_Dongjin | -3.486361642 | 0.677027366 | 746 | -5.149513616 | 4.93E-05 |
| Mock,New_Dongjin - 100mM_NaCl,New_Dongjin | -0.001655813 | 0.669423422 | 746 | -0.002473491 | 1 |
| Mock,New_Dongjin - 150mM_NaCl,New_Dongjin | 1.910089542 | 0.688101564 | 746 | 2.775883158 | 0.330688225 |
| Mock,New_Dongjin - 200mM_NaCl,New_Dongjin | 2.096462394 | 0.665832982 | 746 | 3.148631037 | 1.40E-01 |
| Mock,New_Dongjin - Mock,Chucheongbyeo | 1.247756343 | 0.677060944 | 746 | 1.842901077 | 0.930921536 |
| Mock,New_Dongjin - Mock_DMSO,Chucheongbyeo | 0.68292512 | 0.64211434 | 746 | 1.063556874 | 0.999864121 |
| Mock,New_Dongjin - 1uM_GA3,Chucheongbyeo | -1.835836354 | 0.677135816 | 746 | -2.711178927 | 3.74E-01 |
| Mock,New_Dongjin - 100mM_NaCl,Chucheongbyeo | 1.122041862 | 0.669423422 | 746 | 1.676131765 | 0.970564621 |
| Mock,New_Dongjin - 150mM_NaCl,Chucheongbyeo | 1.513757947 | 0.676109305 | 746 | 2.238924885 | 0.726763139 |
| Mock,New_Dongjin - 200mM_NaCl,Chucheongbyeo | 2.601397689 | 0.669418355 | 746 | 3.88605671 | 1.33E-02 |
| Mock,New_Dongjin - Mock,Chilbo | 2.179176604 | 0.673151662 | 746 | 3.237274343 | 0.109843777 |
| Mock,New_Dongjin - Mock_DMSO,Chilbo | 1.635347142 | 0.673180457 | 746 | 2.429284933 | 0.585652298 |
| Mock,New_Dongjin - 1uM_GA3,Chilbo | -3.143221001 | 0.673149384 | 746 | -4.66942565 | 5.05E-04 |
| Mock,New_Dongjin - 100mM_NaCl,Chilbo | 2.04055028 | 0.6694553 | 746 | 3.048075474 | 0.180453888 |
| Mock,New_Dongjin - 150mM_NaCl,Chilbo | 2.147910108 | 0.665868023 | 746 | 3.225729474 | 1.13E-01 |
| Mock,New_Dongjin - 200mM_NaCl,Chilbo | 3.838580527 | 0.669421689 | 746 | 5.734174122 | 2.14E-06 |
| Mock_DMSO,New_Dongjin - 1uM_GA3,New_Dongjin | -3.329731707 | 0.664047937 | 746 | -5.01429418 | 9.71E-05 |
| Mock_DMSO,New_Dongjin - 100mM_NaCl,New_Dongjin | 0.154974122 | 0.656292608 | 746 | 0.236135712 | 1 |
| Mock_DMSO,New_Dongjin - 150mM_NaCl,New_Dongjin | 2.066719477 | 0.675296485 | 746 | 3.060462365 | 0.175003479 |
| Mock_DMSO,New_Dongjin - 200mM_NaCl,New_Dongjin | 2.253092329 | 0.652630841 | 746 | 3.452322797 | 5.83E-02 |
| Mock_DMSO,New_Dongjin - Mock,Chucheongbyeo | 1.404386278 | 0.664081931 | 746 | 2.114778633 | 0.807063749 |
| Mock_DMSO,New_Dongjin - Mock_DMSO,Chucheongbyeo | 0.839555055 | 0.628416494 | 746 | 1.335985071 | 0.99746445 |
| Mock_DMSO,New_Dongjin - 1uM_GA3,Chucheongbyeo | -1.679206419 | 0.664155586 | 746 | -2.528332902 | 5.09E-01 |
| Mock_DMSO,New_Dongjin - 100mM_NaCl,Chucheongbyeo | 1.278671797 | 0.656292608 | 746 | 1.948325763 | 0.892052878 |
| Mock_DMSO,New_Dongjin - 150mM_NaCl,Chucheongbyeo | 1.670387882 | 0.663075063 | 746 | 2.519153524 | 0.516307177 |
| Mock_DMSO,New_Dongjin - 200mM_NaCl,Chucheongbyeo | 2.758027624 | 0.656290026 | 746 | 4.202452446 | 3.86E-03 |
| Mock_DMSO,New_Dongjin - Mock,Chilbo | 2.335806539 | 0.660094312 | 746 | 3.538595161 | 0.044334684 |
| Mock_DMSO,New_Dongjin - Mock_DMSO,Chilbo | 1.791977077 | 0.660123913 | 746 | 2.714607124 | 0.372000308 |
| Mock_DMSO,New_Dongjin - 1uM_GA3,Chilbo | -2.986591066 | 0.660094622 | 746 | -4.524489319 | 9.74E-04 |
| Mock_DMSO,New_Dongjin - 100mM_NaCl,Chilbo | 2.197180215 | 0.65632277 | 746 | 3.347712919 | 0.080042987 |
| Mock_DMSO,New_Dongjin - 150mM_NaCl,Chilbo | 2.304540043 | 0.652664049 | 746 | 3.530974393 | 4.54E-02 |
| Mock_DMSO,New_Dongjin - 200mM_NaCl,Chilbo | 3.995210462 | 0.656291072 | 746 | 6.087558751 | 2.73E-07 |
| 1uM_GA3,New_Dongjin - 100mM_NaCl,New_Dongjin | 3.484705829 | 0.656292608 | 746 | 5.30968319 | 2.16E-05 |
| 1uM_GA3,New_Dongjin - 150mM_NaCl,New_Dongjin | 5.396451184 | 0.675296485 | 746 | 7.991232454 | 0.00E+00 |
| 1uM_GA3,New_Dongjin - 200mM_NaCl,New_Dongjin | 5.582824036 | 0.652630841 | 746 | 8.554336831 | 0.00E+00 |
| 1uM_GA3,New_Dongjin - Mock,Chucheongbyeo | 4.734117985 | 0.664081931 | 746 | 7.128816136 | 0.00E+00 |
| 1uM_GA3,New_Dongjin - Mock_DMSO,Chucheongbyeo | 4.169286762 | 0.628416494 | 746 | 6.634591547 | 4.17E-09 |
| 1uM_GA3,New_Dongjin - 1uM_GA3,Chucheongbyeo | 1.650525288 | 0.664155586 | 746 | 2.485148546 | 0.542519344 |
| 1uM_GA3,New_Dongjin - 100mM_NaCl,Chucheongbyeo | 4.608403504 | 0.656292608 | 746 | 7.021873241 | 0.00E+00 |
| 1uM_GA3,New_Dongjin - 150mM_NaCl,Chucheongbyeo | 5.00011959 | 0.663075063 | 746 | 7.540804754 | 0.00E+00 |
| 1uM_GA3,New_Dongjin - 200mM_NaCl,Chucheongbyeo | 6.087759332 | 0.656290026 | 746 | 9.276019889 | 0.00E+00 |
| 1uM_GA3,New_Dongjin - Mock,Chilbo | 5.665538246 | 0.660094312 | 746 | 8.582922382 | 0.00E+00 |
| 1uM_GA3,New_Dongjin - Mock_DMSO,Chilbo | 5.121708784 | 0.660123913 | 746 | 7.758708151 | 0.00E+00 |
| 1uM_GA3,New_Dongjin - 1uM_GA3,Chilbo | 0.343140641 | 0.660094622 | 746 | 0.519835535 | 0.999999997 |
| 1uM_GA3,New_Dongjin - 100mM_NaCl,Chilbo | 5.526911923 | 0.65632277 | 746 | 8.421027242 | 0.00E+00 |
| 1uM_GA3,New_Dongjin - 150mM_NaCl,Chilbo | 5.63427175 | 0.652664049 | 746 | 8.632728832 | 0.00E+00 |
| 1uM_GA3,New_Dongjin - 200mM_NaCl,Chilbo | 7.324942169 | 0.656291072 | 746 | 11.1611181 | 0.00E+00 |
| 100mM_NaCl,New_Dongjin - 150mM_NaCl,New_Dongjin | 1.911745355 | 0.667166862 | 746 | 2.865468093 | 0.274934922 |
| 100mM_NaCl,New_Dongjin - 200mM_NaCl,New_Dongjin | 2.098118207 | 0.644737298 | 746 | 3.254221859 | 1.05E-01 |
| 100mM_NaCl,New_Dongjin - Mock,Chucheongbyeo | 1.249412156 | 0.656292597 | 746 | 1.90374257 | 0.90990149 |
| 100mM_NaCl,New_Dongjin - Mock_DMSO,Chucheongbyeo | 0.684580933 | 0.620110639 | 746 | 1.103965793 | 0.999775067 |
| 100mM_NaCl,New_Dongjin - 1uM_GA3,Chucheongbyeo | -1.834180541 | 0.656330554 | 746 | -2.794598745 | 3.19E-01 |
| 100mM_NaCl,New_Dongjin - 100mM_NaCl,Chucheongbyeo | 1.123697674 | 0.648421091 | 746 | 1.732975207 | 0.959759495 |
| 100mM_NaCl,New_Dongjin - 150mM_NaCl,Chucheongbyeo | 1.51541376 | 0.654795847 | 746 | 2.314330136 | 0.672688564 |
| 100mM_NaCl,New_Dongjin - 200mM_NaCl,Chucheongbyeo | 2.603053502 | 0.648453774 | 746 | 4.014246821 | 8.16E-03 |
| 100mM_NaCl,New_Dongjin - Mock,Chilbo | 2.180832417 | 0.652278985 | 746 | 3.343404384 | 0.081066311 |
| 100mM_NaCl,New_Dongjin - Mock_DMSO,Chilbo | 1.637002955 | 0.652342732 | 746 | 2.509421618 | 0.523796364 |
| 100mM_NaCl,New_Dongjin - 1uM_GA3,Chilbo | -3.141565188 | 0.652315222 | 746 | -4.816023119 | 2.54E-04 |
| 100mM_NaCl,New_Dongjin - 100mM_NaCl,Chilbo | 2.042206093 | 0.648449526 | 746 | 3.149367857 | 0.139361624 |
| 100mM_NaCl,New_Dongjin - 150mM_NaCl,Chilbo | 2.149565921 | 0.64473622 | 746 | 3.334023828 | 0.083331533 |
| 100mM_NaCl,New_Dongjin - 200mM_NaCl,Chilbo | 3.84023634 | 0.648452741 | 746 | 5.922152992 | 7.29E-07 |
| 150mM_NaCl,New_Dongjin - 200mM_NaCl,New_Dongjin | 0.186372852 | 0.664038975 | 746 | 0.280665532 | 1 |
| 150mM_NaCl,New_Dongjin - Mock,Chucheongbyeo | -0.662333199 | 0.674818459 | 746 | -0.981498342 | 0.999955393 |
| 150mM_NaCl,New_Dongjin - Mock_DMSO,Chucheongbyeo | -1.227164422 | 0.639282807 | 746 | -1.919595534 | 0.903794938 |
| 150mM_NaCl,New_Dongjin - 1uM_GA3,Chucheongbyeo | -3.745925896 | 0.673902059 | 746 | -5.558561284 | 5.68E-06 |
| 150mM_NaCl,New_Dongjin - 100mM_NaCl,Chucheongbyeo | -0.78804768 | 0.667166862 | 746 | -1.181185286 | 0.999452216 |
| 150mM_NaCl,New_Dongjin - 150mM_NaCl,Chucheongbyeo | -0.396331594 | 0.660496424 | 746 | -0.600051083 | 0.999999974 |
| 150mM_NaCl,New_Dongjin - 200mM_NaCl,Chucheongbyeo | 0.691308148 | 0.66811675 | 746 | 1.034711594 | 0.999906817 |
| 150mM_NaCl,New_Dongjin - Mock,Chilbo | 0.269087062 | 0.670918148 | 746 | 0.401072863 | 1 |
| 150mM_NaCl,New_Dongjin - Mock_DMSO,Chilbo | -0.2747424 | 0.67144943 | 746 | -0.409178097 | 1 |
| 150mM_NaCl,New_Dongjin - 1uM_GA3,Chilbo | -5.053310543 | 0.671888094 | 746 | -7.521059817 | 0.00E+00 |
| 150mM_NaCl,New_Dongjin - 100mM_NaCl,Chilbo | 0.130460739 | 0.666736733 | 746 | 0.195670543 | 1 |
| 150mM_NaCl,New_Dongjin - 150mM_NaCl,Chilbo | 0.237820566 | 0.663135167 | 746 | 0.358630605 | 1 |
| 150mM_NaCl,New_Dongjin - 200mM_NaCl,Chilbo | 1.928490985 | 0.667658614 | 746 | 2.888438707 | 0.261603024 |
| 200mM_NaCl,New_Dongjin - Mock,Chucheongbyeo | -0.848706051 | 0.652665218 | 746 | -1.300369665 | 0.998168916 |
| 200mM_NaCl,New_Dongjin - Mock_DMSO,Chucheongbyeo | -1.413537274 | 0.616341715 | 746 | -2.293431128 | 6.88E-01 |
| 200mM_NaCl,New_Dongjin - 1uM_GA3,Chucheongbyeo | -3.932298748 | 0.652737806 | 746 | -6.024315911 | 3.99E-07 |
| 200mM_NaCl,New_Dongjin - 100mM_NaCl,Chucheongbyeo | -0.974420532 | 0.644737298 | 746 | -1.511345064 | 0.989721903 |
| 200mM_NaCl,New_Dongjin - 150mM_NaCl,Chucheongbyeo | -0.582704447 | 0.651608543 | 746 | -0.894255382 | 0.999988259 |
| 200mM_NaCl,New_Dongjin - 200mM_NaCl,Chucheongbyeo | 0.504935295 | 0.644736942 | 746 | 0.783164826 | 0.999998362 |
| 200mM_NaCl,New_Dongjin - Mock,Chilbo | 0.08271421 | 0.648606116 | 746 | 0.127526102 | 1 |
| 200mM_NaCl,New_Dongjin - Mock_DMSO,Chilbo | -0.461115252 | 0.648636449 | 746 | -0.710899384 | 0.999999627 |
| 200mM_NaCl,New_Dongjin - 1uM_GA3,Chilbo | -5.239683395 | 0.648608745 | 746 | -8.078342198 | 0.00E+00 |
| 200mM_NaCl,New_Dongjin - 100mM_NaCl,Chilbo | -0.055912114 | 0.64476593 | 746 | -0.086716917 | 1 |
| 200mM_NaCl,New_Dongjin - 150mM_NaCl,Chilbo | 0.051447714 | 0.641041896 | 746 | 0.080256398 | 1 |
| 200mM_NaCl,New_Dongjin - 200mM_NaCl,Chilbo | 1.742118133 | 0.644735938 | 746 | 2.702064567 | 0.380741334 |
| Mock,Chucheongbyeo - Mock_DMSO,Chucheongbyeo | -0.564831223 | 0.628203128 | 746 | -0.899121952 | 0.999987294 |
| Mock,Chucheongbyeo - 1uM_GA3,Chucheongbyeo | -3.083592697 | 0.664083041 | 746 | -4.643384196 | 5.70E-04 |
| Mock,Chucheongbyeo - 100mM_NaCl,Chucheongbyeo | -0.125714482 | 0.656292597 | 746 | -0.19155249 | 1 |
| Mock,Chucheongbyeo - 150mM_NaCl,Chucheongbyeo | 0.266001604 | 0.662559392 | 746 | 0.401475864 | 1 |
| Mock,Chucheongbyeo - 200mM_NaCl,Chucheongbyeo | 1.353641346 | 0.656327213 | 746 | 2.062448912 | 0.836821227 |
| Mock,Chucheongbyeo - Mock,Chilbo | 0.931420261 | 0.660125449 | 746 | 1.410974629 | 0.995201684 |
| Mock,Chucheongbyeo - Mock_DMSO,Chilbo | 0.387590799 | 0.660221807 | 746 | 0.587061492 | 0.999999981 |
| Mock,Chucheongbyeo - 1uM_GA3,Chilbo | -4.390977344 | 0.660163621 | 746 | -6.651347034 | 3.20E-09 |
| Mock,Chucheongbyeo - 100mM_NaCl,Chilbo | 0.792793937 | 0.656351151 | 746 | 1.207880776 | 0.999270113 |
| Mock,Chucheongbyeo - 150mM_NaCl,Chilbo | 0.900153765 | 0.652661869 | 746 | 1.379203853 | 0.996310036 |
| Mock,Chucheongbyeo - 200mM_NaCl,Chilbo | 2.590824184 | 0.656356651 | 746 | 3.947281068 | 0.010540828 |
| Mock_DMSO,Chucheongbyeo - 1uM_GA3,Chucheongbyeo | -2.518761474 | 0.628199535 | 746 | -4.009492737 | 8.31E-03 |
| Mock_DMSO,Chucheongbyeo - 100mM_NaCl,Chucheongbyeo | 0.439116742 | 0.620110639 | 746 | 0.708126444 | 0.999999648 |
| Mock_DMSO,Chucheongbyeo - 150mM_NaCl,Chucheongbyeo | 0.830832827 | 0.62619451 | 746 | 1.326796728 | 0.997665311 |
| Mock_DMSO,Chucheongbyeo - 200mM_NaCl,Chucheongbyeo | 1.918472569 | 0.620186585 | 746 | 3.093379665 | 0.161101714 |
| Mock_DMSO,Chucheongbyeo - Mock,Chilbo | 1.496251484 | 0.624266226 | 746 | 2.396816329 | 0.610589304 |
| Mock_DMSO,Chucheongbyeo - Mock_DMSO,Chilbo | 0.952422022 | 0.6245424 | 746 | 1.524991772 | 0.988685527 |
| Mock_DMSO,Chucheongbyeo - 1uM_GA3,Chilbo | -3.826146121 | 0.624346569 | 746 | -6.128240806 | 2.13E-07 |
| Mock_DMSO,Chucheongbyeo - 100mM_NaCl,Chilbo | 1.35762516 | 0.620304689 | 746 | 2.188642428 | 0.760782025 |
| Mock_DMSO,Chucheongbyeo - 150mM_NaCl,Chilbo | 1.464984988 | 0.616299512 | 746 | 2.377066604 | 0.625654108 |
| Mock_DMSO,Chucheongbyeo - 200mM_NaCl,Chilbo | 3.155655407 | 0.620349809 | 746 | 5.086896715 | 6.76E-05 |
| 1uM_GA3,Chucheongbyeo - 100mM_NaCl,Chucheongbyeo | 2.957878216 | 0.656330554 | 746 | 4.506689808 | 1.05E-03 |
| 1uM_GA3,Chucheongbyeo - 150mM_NaCl,Chucheongbyeo | 3.349594302 | 0.66165706 | 746 | 5.06243265 | 7.64E-05 |
| 1uM_GA3,Chucheongbyeo - 200mM_NaCl,Chucheongbyeo | 4.437234044 | 0.656432891 | 746 | 6.759615652 | 0.00E+00 |
| 1uM_GA3,Chucheongbyeo - Mock,Chilbo | 4.015012958 | 0.660165393 | 746 | 6.081828888 | 2.83E-07 |
| 1uM_GA3,Chucheongbyeo - Mock_DMSO,Chilbo | 3.471183496 | 0.6602996 | 746 | 5.256982585 | 2.84E-05 |
| 1uM_GA3,Chucheongbyeo - 1uM_GA3,Chilbo | -1.307384647 | 0.660272496 | 746 | -1.980068313 | 0.878062916 |
| 1uM_GA3,Chucheongbyeo - 100mM_NaCl,Chilbo | 3.876386635 | 0.656358572 | 746 | 5.905897786 | 8.02E-07 |
| 1uM_GA3,Chucheongbyeo - 150mM_NaCl,Chilbo | 3.983746462 | 0.652667898 | 746 | 6.103787966 | 2.48E-07 |
| 1uM_GA3,Chucheongbyeo - 200mM_NaCl,Chilbo | 5.674416881 | 0.656431796 | 746 | 8.644335809 | 0.00E+00 |
| 100mM_NaCl,Chucheongbyeo - 150mM_NaCl,Chucheongbyeo | 0.391716086 | 0.654795847 | 746 | 0.598226284 | 0.999999975 |
| 100mM_NaCl,Chucheongbyeo - 200mM_NaCl,Chucheongbyeo | 1.479355828 | 0.648453774 | 746 | 2.281358959 | 0.696726887 |
| 100mM_NaCl,Chucheongbyeo - Mock,Chilbo | 1.057134742 | 0.652278985 | 746 | 1.620678831 | 0.978812933 |
| 100mM_NaCl,Chucheongbyeo - Mock_DMSO,Chilbo | 0.51330528 | 0.652342732 | 746 | 0.786864412 | 0.999998241 |
| 100mM_NaCl,Chucheongbyeo - 1uM_GA3,Chilbo | -4.265262863 | 0.652315222 | 746 | -6.538652972 | 1.22E-08 |
| 100mM_NaCl,Chucheongbyeo - 100mM_NaCl,Chilbo | 0.918508419 | 0.648449526 | 746 | 1.416468641 | 0.994984208 |
| 100mM_NaCl,Chucheongbyeo - 150mM_NaCl,Chilbo | 1.025868246 | 0.64473622 | 746 | 1.591144121 | 0.982395803 |
| 100mM_NaCl,Chucheongbyeo - 200mM_NaCl,Chilbo | 2.716538665 | 0.648452741 | 746 | 4.189262368 | 0.004071904 |
| 150mM_NaCl,Chucheongbyeo - 200mM_NaCl,Chucheongbyeo | 1.087639742 | 0.655733787 | 746 | 1.658660517 | 0.973392064 |
| 150mM_NaCl,Chucheongbyeo - Mock,Chilbo | 0.665418656 | 0.658645945 | 746 | 1.01028278 | 0.999933103 |
| 150mM_NaCl,Chucheongbyeo - Mock_DMSO,Chilbo | 0.121589195 | 0.659215404 | 746 | 0.184445318 | 1 |
| 150mM_NaCl,Chucheongbyeo - 1uM_GA3,Chilbo | -4.656978948 | 0.659603537 | 746 | -7.06026983 | 0.00E+00 |
| 150mM_NaCl,Chucheongbyeo - 100mM_NaCl,Chilbo | 0.526792333 | 0.654415347 | 746 | 0.80498163 | 0.999997522 |
| 150mM_NaCl,Chucheongbyeo - 150mM_NaCl,Chilbo | 0.63415216 | 0.650716885 | 746 | 0.974543884 | 0.999959654 |
| 150mM_NaCl,Chucheongbyeo - 200mM_NaCl,Chilbo | 2.324822579 | 0.655324675 | 746 | 3.547588958 | 0.043060813 |
| 200mM_NaCl,Chucheongbyeo - Mock,Chilbo | -0.422221086 | 0.652309344 | 746 | -0.647271252 | 0.999999913 |
| 200mM_NaCl,Chucheongbyeo - Mock_DMSO,Chilbo | -0.966050547 | 0.652336556 | 746 | -1.480908188 | 0.991756131 |
| 200mM_NaCl,Chucheongbyeo - 1uM_GA3,Chilbo | -5.74461869 | 0.652279054 | 746 | -8.806995499 | 0.00E+00 |
| 200mM_NaCl,Chucheongbyeo - 100mM_NaCl,Chilbo | -0.560847409 | 0.648511673 | 746 | -0.864822381 | 0.999992807 |
| 200mM_NaCl,Chucheongbyeo - 150mM_NaCl,Chilbo | -0.453487582 | 0.644800109 | 746 | -0.70329948 | 0.999999684 |
| 200mM_NaCl,Chucheongbyeo - 200mM_NaCl,Chilbo | 1.237182837 | 0.648449526 | 746 | 1.907909233 | 0.908322037 |
| Mock,Chilbo - Mock_DMSO,Chilbo | -0.543829462 | 0.656127767 | 746 | -0.828846894 | 0.999996165 |
| Mock,Chilbo - 1uM_GA3,Chilbo | -5.322397605 | 0.656128838 | 746 | -8.111817827 | 0.00E+00 |
| Mock,Chilbo - 100mM_NaCl,Chilbo | -0.138626323 | 0.652279327 | 746 | -0.212526011 | 1 |
| Mock,Chilbo - 150mM_NaCl,Chilbo | -0.031266496 | 0.64860714 | 746 | -0.0482056 | 1 |
| Mock,Chilbo - 200mM_NaCl,Chilbo | 1.659403923 | 0.652280393 | 746 | 2.544003993 | 0.497252522 |
| Mock_DMSO,Chilbo - 1uM_GA3,Chilbo | -4.778568143 | 0.656124437 | 746 | -7.283021139 | 0.00E+00 |
| Mock_DMSO,Chilbo - 100mM_NaCl,Chilbo | 0.405203138 | 0.652315188 | 746 | 0.621176918 | 0.999999955 |
| Mock_DMSO,Chilbo - 150mM_NaCl,Chilbo | 0.512562966 | 0.648673378 | 746 | 0.790171115 | 0.999998126 |
| Mock_DMSO,Chilbo - 200mM_NaCl,Chilbo | 2.203233385 | 0.652279718 | 746 | 3.377743204 | 0.07320256 |
| 1uM_GA3,Chilbo - 100mM_NaCl,Chilbo | 5.183771281 | 0.652345554 | 746 | 7.946357957 | 0.00E+00 |
| 1uM_GA3,Chilbo - 150mM_NaCl,Chilbo | 5.291131109 | 0.64867515 | 746 | 8.156827206 | 0.00E+00 |
| 1uM_GA3,Chilbo - 200mM_NaCl,Chilbo | 6.981801528 | 0.652280094 | 746 | 10.70368634 | 0.00E+00 |
| 100mM_NaCl,Chilbo - 150mM_NaCl,Chilbo | 0.107359827 | 0.644735889 | 746 | 0.166517529 | 1 |
| 100mM_NaCl,Chilbo - 200mM_NaCl,Chilbo | 1.798030246 | 0.648453774 | 746 | 2.772796331 | 0.332710954 |
| 150mM_NaCl,Chilbo - 200mM_NaCl,Chilbo | 1.690670419 | 0.644770144 | 746 | 2.622128886 | 0.438395168 |

Seminal Root Length (mm)

| contrast | estimate | SE | df | t.ratio | p.value |
| --- | --- | --- | --- | --- | --- |
| Mock,New_Dongjin - Mock_DMSO,New_Dongjin | -0.702120475 | 1.752940295 | 869 | -0.400538727 | 1 |
| Mock,New_Dongjin - 1uM_GA3,New_Dongjin | -6.929753876 | 1.877229714 | 869 | -3.691478897 | 2.64E-02 |
| Mock,New_Dongjin - 100mM_NaCl,New_Dongjin | 9.391812871 | 1.89774126 | 869 | 4.94894276 | 0.000130259 |
| Mock,New_Dongjin - 150mM_NaCl,New_Dongjin | 18.13673121 | 1.958837099 | 869 | 9.258927773 | 8.97E-13 |
| Mock,New_Dongjin - 200mM_NaCl,New_Dongjin | 21.09734743 | 1.886312649 | 869 | 11.1844383 | 7.96E-13 |
| Mock,New_Dongjin - Mock,Chucheongbyeo | 5.742753189 | 1.782332835 | 869 | 3.222043087 | 0.114184326 |
| Mock,New_Dongjin - Mock_DMSO,Chucheongbyeo | 7.3673648 | 1.839459232 | 869 | 4.005179713 | 0.008355501 |
| Mock,New_Dongjin - 1uM_GA3,Chucheongbyeo | 2.277115659 | 1.912233728 | 869 | 1.190814504 | 9.99E-01 |
| Mock,New_Dongjin - 100mM_NaCl,Chucheongbyeo | 9.900471537 | 1.7595955 | 869 | 5.626561068 | 3.73E-06 |
| Mock,New_Dongjin - 150mM_NaCl,Chucheongbyeo | 16.63401952 | 1.78316424 | 869 | 9.328372086 | 9.01E-13 |
| Mock,New_Dongjin - 200mM_NaCl,Chucheongbyeo | 23.20213018 | 1.924755914 | 869 | 12.05458313 | 7.27E-13 |
| Mock,New_Dongjin - Mock,Chilbo | 15.60548213 | 1.799047111 | 869 | 8.674304322 | 8.89E-13 |
| Mock,New_Dongjin - Mock_DMSO,Chilbo | 16.23368977 | 1.826006234 | 869 | 8.890270723 | 8.81E-13 |
| Mock,New_Dongjin - 1uM_GA3,Chilbo | 13.52240567 | 1.845801655 | 869 | 7.32603399 | 8.36E-11 |
| Mock,New_Dongjin - 100mM_NaCl,Chilbo | 19.85226465 | 1.752615439 | 869 | 11.32722228 | 7.67E-13 |
| Mock,New_Dongjin - 150mM_NaCl,Chilbo | 22.79894428 | 1.782806736 | 869 | 12.78823095 | 7.27E-13 |
| Mock,New_Dongjin - 200mM_NaCl,Chilbo | 24.38419274 | 1.79885479 | 869 | 13.55539806 | 7.27E-13 |
| Mock_DMSO,New_Dongjin - 1uM_GA3,New_Dongjin | -6.227633401 | 1.81627875 | 869 | -3.42878724 | 6.24E-02 |
| Mock_DMSO,New_Dongjin - 100mM_NaCl,New_Dongjin | 10.09393335 | 1.841436475 | 869 | 5.48155393 | 8.28E-06 |
| Mock_DMSO,New_Dongjin - 150mM_NaCl,New_Dongjin | 18.83885169 | 1.903549397 | 869 | 9.896697044 | 8.96E-13 |
| Mock_DMSO,New_Dongjin - 200mM_NaCl,New_Dongjin | 21.7994679 | 1.829690667 | 869 | 11.91429147 | 7.28E-13 |
| Mock_DMSO,New_Dongjin - Mock,Chucheongbyeo | 6.444873664 | 1.717770934 | 869 | 3.751881892 | 0.021365773 |
| Mock_DMSO,New_Dongjin - Mock_DMSO,Chucheongbyeo | 8.069485275 | 1.776725285 | 869 | 4.541774321 | 0.00088432 |
| Mock_DMSO,New_Dongjin - 1uM_GA3,Chucheongbyeo | 2.979236134 | 1.850527068 | 869 | 1.609939236 | 9.80E-01 |
| Mock_DMSO,New_Dongjin - 100mM_NaCl,Chucheongbyeo | 10.60259201 | 1.694086156 | 869 | 6.25859079 | 9.28E-08 |
| Mock_DMSO,New_Dongjin - 150mM_NaCl,Chucheongbyeo | 17.33614 | 1.717818305 | 869 | 10.09195207 | 8.97E-13 |
| Mock_DMSO,New_Dongjin - 200mM_NaCl,Chucheongbyeo | 23.90425066 | 1.863345586 | 869 | 12.82867271 | 7.27E-13 |
| Mock_DMSO,New_Dongjin - Mock,Chilbo | 16.3076026 | 1.736020829 | 869 | 9.393667591 | 9.03E-13 |
| Mock_DMSO,New_Dongjin - Mock_DMSO,Chilbo | 16.93581024 | 1.764132012 | 869 | 9.600081016 | 8.99E-13 |
| Mock_DMSO,New_Dongjin - 1uM_GA3,Chilbo | 14.22452614 | 1.783175573 | 869 | 7.977075481 | 1.65E-12 |
| Mock_DMSO,New_Dongjin - 100mM_NaCl,Chilbo | 20.55438512 | 1.686579586 | 869 | 12.18702355 | 7.27E-13 |
| Mock_DMSO,New_Dongjin - 150mM_NaCl,Chilbo | 23.50106475 | 1.71928815 | 869 | 13.66906691 | 7.27E-13 |
| Mock_DMSO,New_Dongjin - 200mM_NaCl,Chilbo | 25.08631321 | 1.735477328 | 869 | 14.45499334 | 7.27E-13 |
| 1uM_GA3,New_Dongjin - 100mM_NaCl,New_Dongjin | 16.32156675 | 1.955346786 | 869 | 8.347146841 | 9.59E-13 |
| 1uM_GA3,New_Dongjin - 150mM_NaCl,New_Dongjin | 25.06648509 | 2.011966974 | 869 | 12.4586961 | 7.27E-13 |
| 1uM_GA3,New_Dongjin - 200mM_NaCl,New_Dongjin | 28.02710131 | 1.944326842 | 869 | 14.41480964 | 7.27E-13 |
| 1uM_GA3,New_Dongjin - Mock,Chucheongbyeo | 12.67250706 | 1.844454553 | 869 | 6.870598706 | 1.86E-09 |
| 1uM_GA3,New_Dongjin - Mock_DMSO,Chucheongbyeo | 14.29711868 | 1.898924774 | 869 | 7.529060063 | 2.04E-11 |
| 1uM_GA3,New_Dongjin - 1uM_GA3,Chucheongbyeo | 9.206869534 | 1.969445496 | 869 | 4.674853685 | 0.000481983 |
| 1uM_GA3,New_Dongjin - 100mM_NaCl,Chucheongbyeo | 16.83022541 | 1.822470482 | 869 | 9.234841154 | 9.02E-13 |
| 1uM_GA3,New_Dongjin - 150mM_NaCl,Chucheongbyeo | 23.5637734 | 1.843063067 | 869 | 12.78511507 | 7.27E-13 |
| 1uM_GA3,New_Dongjin - 200mM_NaCl,Chucheongbyeo | 30.13188406 | 1.981728721 | 869 | 15.20484804 | 7.27E-13 |
| 1uM_GA3,New_Dongjin - Mock,Chilbo | 22.535236 | 1.860361363 | 869 | 12.11336488 | 7.27E-13 |
| 1uM_GA3,New_Dongjin - Mock_DMSO,Chilbo | 23.16344364 | 1.886479395 | 869 | 12.27866241 | 7.27E-13 |
| 1uM_GA3,New_Dongjin - 1uM_GA3,Chilbo | 20.45215954 | 1.905714454 | 869 | 10.73201681 | 8.84E-13 |
| 1uM_GA3,New_Dongjin - 100mM_NaCl,Chilbo | 26.78201852 | 1.815651131 | 869 | 14.75064128 | 7.27E-13 |
| 1uM_GA3,New_Dongjin - 150mM_NaCl,Chilbo | 29.72869815 | 1.844573881 | 869 | 16.11683785 | 7.27E-13 |
| 1uM_GA3,New_Dongjin - 200mM_NaCl,Chilbo | 31.31394661 | 1.860206367 | 869 | 16.83358749 | 7.27E-13 |
| 100mM_NaCl,New_Dongjin - 150mM_NaCl,New_Dongjin | 8.744918344 | 2.018732293 | 869 | 4.331886091 | 0.002214391 |
| 100mM_NaCl,New_Dongjin - 200mM_NaCl,New_Dongjin | 11.70553456 | 1.947341421 | 869 | 6.01103352 | 4.11E-07 |
| 100mM_NaCl,New_Dongjin - Mock,Chucheongbyeo | -3.649059682 | 1.865466758 | 869 | -1.956110805 | 0.888873171 |
| 100mM_NaCl,New_Dongjin - Mock_DMSO,Chucheongbyeo | -2.02444807 | 1.919619016 | 869 | -1.054609302 | 0.999879804 |
| 100mM_NaCl,New_Dongjin - 1uM_GA3,Chucheongbyeo | -7.114697212 | 1.995534973 | 869 | -3.565308205 | 4.04E-02 |
| 100mM_NaCl,New_Dongjin - 100mM_NaCl,Chucheongbyeo | 0.508658667 | 1.844118417 | 869 | 0.275827551 | 1 |
| 100mM_NaCl,New_Dongjin - 150mM_NaCl,Chucheongbyeo | 7.242206649 | 1.867777054 | 869 | 3.877447062 | 0.013554443 |
| 100mM_NaCl,New_Dongjin - 200mM_NaCl,Chucheongbyeo | 13.81031731 | 2.008064266 | 869 | 6.877427949 | 1.78E-09 |
| 100mM_NaCl,New_Dongjin - Mock,Chilbo | 6.213669257 | 1.877716668 | 869 | 3.309162326 | 0.089228147 |
| 100mM_NaCl,New_Dongjin - Mock_DMSO,Chilbo | 6.841876895 | 1.902869789 | 869 | 3.595557056 | 0.036549691 |
| 100mM_NaCl,New_Dongjin - 1uM_GA3,Chilbo | 4.130592795 | 1.927415621 | 869 | 2.143073217 | 7.90E-01 |
| 100mM_NaCl,New_Dongjin - 100mM_NaCl,Chilbo | 10.46045178 | 1.838482821 | 869 | 5.68971962 | 2.62E-06 |
| 100mM_NaCl,New_Dongjin - 150mM_NaCl,Chilbo | 13.40713141 | 1.861652049 | 869 | 7.201738594 | 1.98E-10 |
| 100mM_NaCl,New_Dongjin - 200mM_NaCl,Chilbo | 14.99237987 | 1.878986105 | 869 | 7.978973248 | 1.64E-12 |
| 150mM_NaCl,New_Dongjin - 200mM_NaCl,New_Dongjin | 2.960616215 | 2.009658881 | 869 | 1.473193408 | 0.992247856 |
| 150mM_NaCl,New_Dongjin - Mock,Chucheongbyeo | -12.39397803 | 1.925871982 | 869 | -6.435514999 | 3.10E-08 |
| 150mM_NaCl,New_Dongjin - Mock_DMSO,Chucheongbyeo | -10.76936641 | 1.954049587 | 869 | -5.511306615 | 7.04E-06 |
| 150mM_NaCl,New_Dongjin - 1uM_GA3,Chucheongbyeo | -15.85961556 | 2.026849654 | 869 | -7.824761706 | 3.15E-12 |
| 150mM_NaCl,New_Dongjin - 100mM_NaCl,Chucheongbyeo | -8.236259677 | 1.905308241 | 869 | -4.322796437 | 0.002301623 |
| 150mM_NaCl,New_Dongjin - 150mM_NaCl,Chucheongbyeo | -1.502711694 | 1.890898229 | 869 | -0.794707865 | 0.999997976 |
| 150mM_NaCl,New_Dongjin - 200mM_NaCl,Chucheongbyeo | 5.065398966 | 2.040115914 | 869 | 2.482897629 | 0.544155739 |
| 150mM_NaCl,New_Dongjin - Mock,Chilbo | -2.531249087 | 1.938575668 | 869 | -1.305726224 | 0.998085506 |
| 150mM_NaCl,New_Dongjin - Mock_DMSO,Chilbo | -1.903041448 | 1.964610149 | 869 | -0.96866111 | 0.999963257 |
| 150mM_NaCl,New_Dongjin - 1uM_GA3,Chilbo | -4.614325548 | 1.980883185 | 869 | -2.3294284 | 6.62E-01 |
| 150mM_NaCl,New_Dongjin - 100mM_NaCl,Chilbo | 1.715533433 | 1.898629332 | 869 | 0.903564168 | 0.999986464 |
| 150mM_NaCl,New_Dongjin - 150mM_NaCl,Chilbo | 4.662213063 | 1.921645656 | 869 | 2.426156482 | 0.588010042 |
| 150mM_NaCl,New_Dongjin - 200mM_NaCl,Chilbo | 6.247461521 | 1.939751046 | 869 | 3.220754299 | 0.114591223 |
| 200mM_NaCl,New_Dongjin - Mock,Chucheongbyeo | -15.35459424 | 1.853948891 | 869 | -8.28210223 | 9.94E-13 |
| 200mM_NaCl,New_Dongjin - Mock_DMSO,Chucheongbyeo | -13.72998263 | 1.909791096 | 869 | -7.189258899 | 2.16E-10 |
| 200mM_NaCl,New_Dongjin - 1uM_GA3,Chucheongbyeo | -18.82023177 | 1.985970805 | 869 | -9.476590352 | 9.02E-13 |
| 200mM_NaCl,New_Dongjin - 100mM_NaCl,Chucheongbyeo | -11.19687589 | 1.832430875 | 869 | -6.110394693 | 2.28E-07 |
| 200mM_NaCl,New_Dongjin - 150mM_NaCl,Chucheongbyeo | -4.463327909 | 1.857628775 | 869 | -2.402701751 | 0.606050887 |
| 200mM_NaCl,New_Dongjin - 200mM_NaCl,Chucheongbyeo | 2.104782751 | 1.998551136 | 869 | 1.053154314 | 0.999882056 |
| 200mM_NaCl,New_Dongjin - Mock,Chilbo | -5.491865302 | 1.866177068 | 869 | -2.942842561 | 0.231270477 |
| 200mM_NaCl,New_Dongjin - Mock_DMSO,Chilbo | -4.863657663 | 1.891402722 | 869 | -2.571455358 | 0.476178482 |
| 200mM_NaCl,New_Dongjin - 1uM_GA3,Chilbo | -7.574941763 | 1.916523758 | 869 | -3.952438226 | 1.02E-02 |
| 200mM_NaCl,New_Dongjin - 100mM_NaCl,Chilbo | -1.245082782 | 1.826764831 | 869 | -0.681578034 | 0.999999807 |
| 200mM_NaCl,New_Dongjin - 150mM_NaCl,Chilbo | 1.701596848 | 1.850079187 | 869 | 0.919742712 | 0.999982497 |
| 200mM_NaCl,New_Dongjin - 200mM_NaCl,Chilbo | 3.286845307 | 1.867427761 | 869 | 1.760092344 | 0.953779521 |
| Mock,Chucheongbyeo - Mock_DMSO,Chucheongbyeo | 1.624611612 | 1.803896671 | 869 | 0.900612345 | 0.999987092 |
| Mock,Chucheongbyeo - 1uM_GA3,Chucheongbyeo | -3.46563753 | 1.87836832 | 869 | -1.845025543 | 9.30E-01 |
| Mock,Chucheongbyeo - 100mM_NaCl,Chucheongbyeo | 4.157718349 | 1.724519528 | 869 | 2.410943037 | 0.599722213 |
| Mock,Chucheongbyeo - 150mM_NaCl,Chucheongbyeo | 10.89126633 | 1.747121401 | 869 | 6.233834882 | 1.08E-07 |
| Mock,Chucheongbyeo - 200mM_NaCl,Chucheongbyeo | 17.45937699 | 1.891090569 | 869 | 9.232438297 | 9.03E-13 |
| Mock,Chucheongbyeo - Mock,Chilbo | 9.862728939 | 1.764876675 | 869 | 5.588338878 | 4.61E-06 |
| Mock,Chucheongbyeo - Mock_DMSO,Chilbo | 10.49093658 | 1.792458033 | 869 | 5.852821313 | 1.04E-06 |
| Mock,Chucheongbyeo - 1uM_GA3,Chilbo | 7.779652478 | 1.811979176 | 869 | 4.293455787 | 2.61E-03 |
| Mock,Chucheongbyeo - 100mM_NaCl,Chilbo | 14.10951146 | 1.717438454 | 869 | 8.215439351 | 1.05E-12 |
| Mock,Chucheongbyeo - 150mM_NaCl,Chilbo | 17.05619109 | 1.748232777 | 869 | 9.756247174 | 9.02E-13 |
| Mock,Chucheongbyeo - 200mM_NaCl,Chilbo | 18.64143955 | 1.764738855 | 869 | 10.56328504 | 8.92E-13 |
| Mock_DMSO,Chucheongbyeo - 1uM_GA3,Chucheongbyeo | -5.090249142 | 1.91155191 | 869 | -2.662888262 | 4.08E-01 |
| Mock_DMSO,Chucheongbyeo - 100mM_NaCl,Chucheongbyeo | 2.533106737 | 1.78231821 | 869 | 1.421242695 | 0.994812259 |
| Mock_DMSO,Chucheongbyeo - 150mM_NaCl,Chucheongbyeo | 9.26665472 | 1.784058668 | 869 | 5.19414237 | 3.79E-05 |
| Mock_DMSO,Chucheongbyeo - 200mM_NaCl,Chucheongbyeo | 15.83476538 | 1.923999178 | 869 | 8.230131052 | 1.04E-12 |
| Mock_DMSO,Chucheongbyeo - Mock,Chilbo | 8.238117327 | 1.822312044 | 869 | 4.520695209 | 0.00097185 |
| Mock_DMSO,Chucheongbyeo - Mock_DMSO,Chilbo | 8.866324966 | 1.850310649 | 869 | 4.791803457 | 0.000278413 |
| Mock_DMSO,Chucheongbyeo - 1uM_GA3,Chilbo | 6.155040866 | 1.862163601 | 869 | 3.305316925 | 9.02E-02 |
| Mock_DMSO,Chucheongbyeo - 100mM_NaCl,Chilbo | 12.48489985 | 1.77581018 | 869 | 7.030537377 | 6.37E-10 |
| Mock_DMSO,Chucheongbyeo - 150mM_NaCl,Chilbo | 15.43157948 | 1.805066493 | 869 | 8.549036581 | 9.02E-13 |
| Mock_DMSO,Chucheongbyeo - 200mM_NaCl,Chilbo | 17.01682794 | 1.822910362 | 869 | 9.334977898 | 9.00E-13 |
| 1uM_GA3,Chucheongbyeo - 100mM_NaCl,Chucheongbyeo | 7.623355879 | 1.857247652 | 869 | 4.104652316 | 5.65E-03 |
| 1uM_GA3,Chucheongbyeo - 150mM_NaCl,Chucheongbyeo | 14.35690386 | 1.85745165 | 869 | 7.729355356 | 5.44E-12 |
| 1uM_GA3,Chucheongbyeo - 200mM_NaCl,Chucheongbyeo | 20.92501452 | 1.993717246 | 869 | 10.49547751 | 8.91E-13 |
| 1uM_GA3,Chucheongbyeo - Mock,Chilbo | 13.32836647 | 1.896801517 | 869 | 7.02675865 | 6.54E-10 |
| 1uM_GA3,Chucheongbyeo - Mock_DMSO,Chilbo | 13.95657411 | 1.92384023 | 869 | 7.254539066 | 1.37E-10 |
| 1uM_GA3,Chucheongbyeo - 1uM_GA3,Chilbo | 11.24529001 | 1.934399964 | 869 | 5.813322073 | 1.30E-06 |
| 1uM_GA3,Chucheongbyeo - 100mM_NaCl,Chilbo | 17.57514899 | 1.850243893 | 869 | 9.498828265 | 8.97E-13 |
| 1uM_GA3,Chucheongbyeo - 150mM_NaCl,Chilbo | 20.52182862 | 1.880516251 | 869 | 10.9128696 | 8.57E-13 |
| 1uM_GA3,Chucheongbyeo - 200mM_NaCl,Chilbo | 22.10707708 | 1.896608035 | 869 | 11.65611274 | 7.34E-13 |
| 100mM_NaCl,Chucheongbyeo - 150mM_NaCl,Chucheongbyeo | 6.733547983 | 1.724042005 | 869 | 3.90567513 | 0.012201781 |
| 100mM_NaCl,Chucheongbyeo - 200mM_NaCl,Chucheongbyeo | 13.30165864 | 1.870138779 | 869 | 7.112658585 | 3.64E-10 |
| 100mM_NaCl,Chucheongbyeo - Mock,Chilbo | 5.70501059 | 1.74196072 | 869 | 3.275051225 | 0.098415172 |
| 100mM_NaCl,Chucheongbyeo - Mock_DMSO,Chilbo | 6.333218229 | 1.769879625 | 869 | 3.578332751 | 0.038696442 |
| 100mM_NaCl,Chucheongbyeo - 1uM_GA3,Chilbo | 3.621934129 | 1.789782281 | 869 | 2.023673028 | 8.57E-01 |
| 100mM_NaCl,Chucheongbyeo - 100mM_NaCl,Chilbo | 9.95179311 | 1.693765758 | 869 | 5.875542745 | 9.09E-07 |
| 100mM_NaCl,Chucheongbyeo - 150mM_NaCl,Chilbo | 12.89847274 | 1.725135687 | 869 | 7.476787386 | 2.93E-11 |
| 100mM_NaCl,Chucheongbyeo - 200mM_NaCl,Chilbo | 14.4837212 | 1.741744439 | 869 | 8.315640843 | 9.74E-13 |
| 150mM_NaCl,Chucheongbyeo - 200mM_NaCl,Chucheongbyeo | 6.56811066 | 1.871064902 | 869 | 3.510359611 | 0.048271567 |
| 150mM_NaCl,Chucheongbyeo - Mock,Chilbo | -1.028537393 | 1.765142612 | 869 | -0.582693651 | 0.999999984 |
| 150mM_NaCl,Chucheongbyeo - Mock_DMSO,Chilbo | -0.400329754 | 1.794079188 | 869 | -0.2231394 | 1 |
| 150mM_NaCl,Chucheongbyeo - 1uM_GA3,Chilbo | -3.111613854 | 1.807381929 | 869 | -1.721613902 | 9.62E-01 |
| 150mM_NaCl,Chucheongbyeo - 100mM_NaCl,Chilbo | 3.218245127 | 1.715880932 | 869 | 1.875564363 | 0.920248925 |
| 150mM_NaCl,Chucheongbyeo - 150mM_NaCl,Chilbo | 6.164924758 | 1.747416547 | 869 | 3.528022421 | 0.045605661 |
| 150mM_NaCl,Chucheongbyeo - 200mM_NaCl,Chilbo | 7.750173216 | 1.764877896 | 869 | 4.391336779 | 0.001715944 |
| 200mM_NaCl,Chucheongbyeo - Mock,Chilbo | -7.596648053 | 1.909580197 | 869 | -3.978177019 | 0.009271159 |
| 200mM_NaCl,Chucheongbyeo - Mock_DMSO,Chilbo | -6.968440414 | 1.936450893 | 869 | -3.598562937 | 0.036185892 |
| 200mM_NaCl,Chucheongbyeo - 1uM_GA3,Chilbo | -9.679724514 | 1.946741717 | 869 | -4.972269526 | 1.16E-04 |
| 200mM_NaCl,Chucheongbyeo - 100mM_NaCl,Chilbo | -3.349865533 | 1.863226477 | 869 | -1.797884248 | 0.944211707 |
| 200mM_NaCl,Chucheongbyeo - 150mM_NaCl,Chilbo | -0.403185903 | 1.893429796 | 869 | -0.212939452 | 1 |
| 200mM_NaCl,Chucheongbyeo - 200mM_NaCl,Chilbo | 1.182062556 | 1.909379255 | 869 | 0.619082119 | 0.999999957 |
| Mock,Chilbo - Mock_DMSO,Chilbo | 0.628207639 | 1.808036607 | 869 | 0.347452942 | 1 |
| Mock,Chilbo - 1uM_GA3,Chilbo | -2.083076461 | 1.829149153 | 869 | -1.138822637 | 1.00E+00 |
| Mock,Chilbo - 100mM_NaCl,Chilbo | 4.24678252 | 1.735057996 | 869 | 2.447631451 | 0.57143033 |
| Mock,Chilbo - 150mM_NaCl,Chilbo | 7.19346215 | 1.764398954 | 869 | 4.077004315 | 0.006308729 |
| Mock,Chilbo - 200mM_NaCl,Chilbo | 8.778710608 | 1.781012942 | 869 | 4.929054923 | 0.000143602 |
| Mock_DMSO,Chilbo - 1uM_GA3,Chilbo | -2.7112841 | 1.856062147 | 869 | -1.460772261 | 9.93E-01 |
| Mock_DMSO,Chilbo - 100mM_NaCl,Chilbo | 3.618574881 | 1.763131096 | 869 | 2.052357247 | 0.842400523 |
| Mock_DMSO,Chilbo - 150mM_NaCl,Chilbo | 6.565254512 | 1.791799459 | 869 | 3.664056533 | 0.029007784 |
| Mock_DMSO,Chilbo - 200mM_NaCl,Chilbo | 8.15050297 | 1.80814713 | 869 | 4.507654734 | 0.001030036 |
| 1uM_GA3,Chilbo - 100mM_NaCl,Chilbo | 6.329858981 | 1.782892141 | 869 | 3.550331977 | 4.24E-02 |
| 1uM_GA3,Chilbo - 150mM_NaCl,Chilbo | 9.276538612 | 1.812925531 | 869 | 5.116888947 | 5.63E-05 |
| 1uM_GA3,Chilbo - 200mM_NaCl,Chilbo | 10.86178707 | 1.829012518 | 869 | 5.93860729 | 6.30E-07 |
| 100mM_NaCl,Chilbo - 150mM_NaCl,Chilbo | 2.94667963 | 1.7181916 | 869 | 1.714988963 | 0.963552487 |
| 100mM_NaCl,Chilbo - 200mM_NaCl,Chilbo | 4.531928089 | 1.734696603 | 869 | 2.612519147 | 0.445307621 |
| 150mM_NaCl,Chilbo - 200mM_NaCl,Chilbo | 1.585248458 | 1.764548771 | 869 | 0.898387443 | 0.999987548 |

Number of Crown Roots

| contrast | estimate | SE | df | t.ratio | p.value |
| --- | --- | --- | --- | --- | --- |
| Mock,New_Dongjin - Mock_DMSO,New_Dongjin | -0.355002228 | 0.295496693 | 737 | -1.201374622 | 0.999318428 |
| Mock,New_Dongjin - 1uM_GA3,New_Dongjin | 0.810190874 | 0.297273879 | 737 | 2.725402166 | 3.65E-01 |
| Mock,New_Dongjin - 100mM_NaCl,New_Dongjin | 1.810524271 | 0.292178006 | 737 | 6.196648044 | 1.41E-07 |
| Mock,New_Dongjin - 150mM_NaCl,New_Dongjin | 2.606815785 | 0.300395064 | 737 | 8.677958133 | 0.00E+00 |
| Mock,New_Dongjin - 200mM_NaCl,New_Dongjin | 3.67931403 | 0.290610771 | 737 | 12.66062514 | 0.00E+00 |
| Mock,New_Dongjin - Mock,Chucheongbyeo | 0.951986764 | 0.29551167 | 737 | 3.221486194 | 0.114780286 |
| Mock,New_Dongjin - Mock_DMSO,Chucheongbyeo | 0.954364137 | 0.280261398 | 737 | 3.40526432 | 0.067394852 |
| Mock,New_Dongjin - 1uM_GA3,Chucheongbyeo | 1.77507308 | 0.29554531 | 737 | 6.006094554 | 4.48E-07 |
| Mock,New_Dongjin - 100mM_NaCl,Chucheongbyeo | 2.810524271 | 0.292178006 | 737 | 9.619219143 | 0.00E+00 |
| Mock,New_Dongjin - 150mM_NaCl,Chucheongbyeo | 3.681490436 | 0.295161293 | 737 | 12.47280904 | 0.00E+00 |
| Mock,New_Dongjin - 200mM_NaCl,Chucheongbyeo | 4.158982773 | 0.303274915 | 737 | 13.71357329 | 0.00E+00 |
| Mock,New_Dongjin - Mock,Chilbo | 0.814969554 | 0.293805149 | 737 | 2.773843677 | 3.32E-01 |
| Mock,New_Dongjin - Mock_DMSO,Chilbo | 1.205766014 | 0.293817794 | 737 | 4.10378826 | 5.75E-03 |
| Mock,New_Dongjin - 1uM_GA3,Chilbo | 2.114594384 | 0.295510887 | 737 | 7.155724133 | 0.00E+00 |
| Mock,New_Dongjin - 100mM_NaCl,Chilbo | 2.884012416 | 0.292191966 | 737 | 9.87026597 | 0.00E+00 |
| Mock,New_Dongjin - 150mM_NaCl,Chilbo | 4.059917188 | 0.290626392 | 737 | 13.96954063 | 0.00E+00 |
| Mock,New_Dongjin - 200mM_NaCl,Chilbo | 4.338779078 | 0.293805149 | 737 | 14.76753929 | 0.00E+00 |
| Mock_DMSO,New_Dongjin - 1uM_GA3,New_Dongjin | 1.165193102 | 0.291642949 | 737 | 3.99527267 | 8.79E-03 |
| Mock_DMSO,New_Dongjin - 100mM_NaCl,New_Dongjin | 2.165526498 | 0.286446896 | 737 | 7.559957976 | 0.00E+00 |
| Mock_DMSO,New_Dongjin - 150mM_NaCl,New_Dongjin | 2.961818013 | 0.294807096 | 737 | 10.04663066 | 0.00E+00 |
| Mock_DMSO,New_Dongjin - 200mM_NaCl,New_Dongjin | 4.034316258 | 0.284848537 | 737 | 14.16302256 | 0.00E+00 |
| Mock_DMSO,New_Dongjin - Mock,Chucheongbyeo | 1.306988992 | 0.289846814 | 737 | 4.509240501 | 0.001044356 |
| Mock_DMSO,New_Dongjin - Mock_DMSO,Chucheongbyeo | 1.309366365 | 0.274282825 | 737 | 4.773781831 | 0.000311427 |
| Mock_DMSO,New_Dongjin - 1uM_GA3,Chucheongbyeo | 2.130075308 | 0.289879923 | 737 | 7.348129826 | 0.00E+00 |
| Mock_DMSO,New_Dongjin - 100mM_NaCl,Chucheongbyeo | 3.165526498 | 0.286446896 | 737 | 11.05100645 | 0.00E+00 |
| Mock_DMSO,New_Dongjin - 150mM_NaCl,Chucheongbyeo | 4.036492664 | 0.289473355 | 737 | 13.94426323 | 0.00E+00 |
| Mock_DMSO,New_Dongjin - 200mM_NaCl,Chucheongbyeo | 4.513985001 | 0.297755974 | 737 | 15.16001491 | 0.00E+00 |
| Mock_DMSO,New_Dongjin - Mock,Chilbo | 1.169971782 | 0.288106106 | 737 | 4.060905892 | 6.81E-03 |
| Mock_DMSO,New_Dongjin - Mock_DMSO,Chilbo | 1.560768242 | 0.28811912 | 737 | 5.417093601 | 1.23E-05 |
| Mock_DMSO,New_Dongjin - 1uM_GA3,Chilbo | 2.469596611 | 0.289847326 | 737 | 8.520336021 | 0.00E+00 |
| Mock_DMSO,New_Dongjin - 100mM_NaCl,Chilbo | 3.239014644 | 0.286460105 | 737 | 11.30703574 | 0.00E+00 |
| Mock_DMSO,New_Dongjin - 150mM_NaCl,Chilbo | 4.414919416 | 0.284863347 | 737 | 15.49837657 | 0.00E+00 |
| Mock_DMSO,New_Dongjin - 200mM_NaCl,Chilbo | 4.693781306 | 0.288106106 | 737 | 16.29184947 | 0.00E+00 |
| 1uM_GA3,New_Dongjin - 100mM_NaCl,New_Dongjin | 1.000333396 | 0.288268787 | 737 | 3.470141202 | 5.52E-02 |
| 1uM_GA3,New_Dongjin - 150mM_NaCl,New_Dongjin | 1.796624911 | 0.296365071 | 737 | 6.06220195 | 3.21E-07 |
| 1uM_GA3,New_Dongjin - 200mM_NaCl,New_Dongjin | 2.869123156 | 0.28669093 | 737 | 10.00772208 | 0.00E+00 |
| 1uM_GA3,New_Dongjin - Mock,Chucheongbyeo | 0.14179589 | 0.291641828 | 737 | 0.486198741 | 1.00E+00 |
| 1uM_GA3,New_Dongjin - Mock_DMSO,Chucheongbyeo | 0.144173263 | 0.276145523 | 737 | 0.522091617 | 1.00E+00 |
| 1uM_GA3,New_Dongjin - 1uM_GA3,Chucheongbyeo | 0.964882205 | 0.291658549 | 737 | 3.308259642 | 0.08985212 |
| 1uM_GA3,New_Dongjin - 100mM_NaCl,Chucheongbyeo | 2.000333396 | 0.288268787 | 737 | 6.939125857 | 0.00E+00 |
| 1uM_GA3,New_Dongjin - 150mM_NaCl,Chucheongbyeo | 2.871299562 | 0.291059893 | 737 | 9.864978413 | 0.00E+00 |
| 1uM_GA3,New_Dongjin - 200mM_NaCl,Chucheongbyeo | 3.348791899 | 0.299455893 | 737 | 11.182922 | 0.00E+00 |
| 1uM_GA3,New_Dongjin - Mock,Chilbo | 0.00477868 | 0.289922738 | 737 | 0.016482598 | 1.00E+00 |
| 1uM_GA3,New_Dongjin - Mock_DMSO,Chilbo | 0.39557514 | 0.289951553 | 737 | 1.36428012 | 9.97E-01 |
| 1uM_GA3,New_Dongjin - 1uM_GA3,Chilbo | 1.304403509 | 0.291674697 | 737 | 4.472117473 | 1.23E-03 |
| 1uM_GA3,New_Dongjin - 100mM_NaCl,Chilbo | 2.073821542 | 0.288281901 | 737 | 7.193727852 | 0.00E+00 |
| 1uM_GA3,New_Dongjin - 150mM_NaCl,Chilbo | 3.249726313 | 0.286690302 | 737 | 11.33532001 | 0.00E+00 |
| 1uM_GA3,New_Dongjin - 200mM_NaCl,Chilbo | 3.528588204 | 0.289922738 | 737 | 12.17078808 | 0.00E+00 |
| 100mM_NaCl,New_Dongjin - 150mM_NaCl,New_Dongjin | 0.796291514 | 0.291253831 | 737 | 2.734012156 | 0.358687328 |
| 100mM_NaCl,New_Dongjin - 200mM_NaCl,New_Dongjin | 1.868789759 | 0.281403435 | 737 | 6.640962856 | 4.39E-09 |
| 100mM_NaCl,New_Dongjin - Mock,Chucheongbyeo | -0.858537506 | 0.286446796 | 737 | -2.997197096 | 0.20414967 |
| 100mM_NaCl,New_Dongjin - Mock_DMSO,Chucheongbyeo | -0.856160133 | 0.270656557 | 737 | -3.163271356 | 0.134360158 |
| 100mM_NaCl,New_Dongjin - 1uM_GA3,Chucheongbyeo | -0.035451191 | 0.286463906 | 737 | -0.123754477 | 1.00E+00 |
| 100mM_NaCl,New_Dongjin - 100mM_NaCl,Chucheongbyeo | 1 | 0.283011141 | 737 | 3.533429797 | 0.045103836 |
| 100mM_NaCl,New_Dongjin - 150mM_NaCl,Chucheongbyeo | 1.870966165 | 0.285854744 | 737 | 6.545163961 | 1.21E-08 |
| 100mM_NaCl,New_Dongjin - 200mM_NaCl,Chucheongbyeo | 2.348458502 | 0.294400542 | 737 | 7.977086193 | 0.00E+00 |
| 100mM_NaCl,New_Dongjin - Mock,Chilbo | -0.995554716 | 0.284695 | 737 | -3.496916756 | 0.050695131 |
| 100mM_NaCl,New_Dongjin - Mock_DMSO,Chilbo | -0.604758256 | 0.284723323 | 737 | -2.124020789 | 0.801524929 |
| 100mM_NaCl,New_Dongjin - 1uM_GA3,Chilbo | 0.304070113 | 0.286479134 | 737 | 1.061404048 | 1.00E+00 |
| 100mM_NaCl,New_Dongjin - 100mM_NaCl,Chilbo | 1.073488146 | 0.283023578 | 737 | 3.792928324 | 1.86E-02 |
| 100mM_NaCl,New_Dongjin - 150mM_NaCl,Chilbo | 2.249392917 | 0.281402876 | 737 | 7.993496537 | 0.00E+00 |
| 100mM_NaCl,New_Dongjin - 200mM_NaCl,Chilbo | 2.528254807 | 0.284695 | 737 | 8.880573265 | 0.00E+00 |
| 150mM_NaCl,New_Dongjin - 200mM_NaCl,New_Dongjin | 1.072498245 | 0.289894503 | 737 | 3.699615667 | 0.025874353 |
| 150mM_NaCl,New_Dongjin - Mock,Chucheongbyeo | -1.654829021 | 0.294590232 | 737 | -5.617392699 | 4.13E-06 |
| 150mM_NaCl,New_Dongjin - Mock_DMSO,Chucheongbyeo | -1.652451648 | 0.279069262 | 737 | -5.921295789 | 7.37E-07 |
| 150mM_NaCl,New_Dongjin - 1uM_GA3,Chucheongbyeo | -0.831742705 | 0.294181895 | 737 | -2.827307592 | 2.98E-01 |
| 150mM_NaCl,New_Dongjin - 100mM_NaCl,Chucheongbyeo | 0.203708486 | 0.291253831 | 737 | 0.699419077 | 0.999999709 |
| 150mM_NaCl,New_Dongjin - 150mM_NaCl,Chucheongbyeo | 1.074674651 | 0.288281592 | 737 | 3.727864289 | 0.023453223 |
| 150mM_NaCl,New_Dongjin - 200mM_NaCl,Chucheongbyeo | 1.552166988 | 0.301404173 | 737 | 5.149785989 | 4.93E-05 |
| 150mM_NaCl,New_Dongjin - Mock,Chilbo | -1.791846231 | 0.292893183 | 737 | -6.117746453 | 2.29E-07 |
| 150mM_NaCl,New_Dongjin - Mock_DMSO,Chilbo | -1.401049771 | 0.293133525 | 737 | -4.7795617 | 0.000303055 |
| 150mM_NaCl,New_Dongjin - 1uM_GA3,Chilbo | -0.492221402 | 0.295262449 | 737 | -1.667064007 | 9.72E-01 |
| 150mM_NaCl,New_Dongjin - 100mM_NaCl,Chilbo | 0.277196631 | 0.291065407 | 737 | 0.952351687 | 0.999970889 |
| 150mM_NaCl,New_Dongjin - 150mM_NaCl,Chilbo | 1.453101403 | 0.289491535 | 737 | 5.019495311 | 9.49E-05 |
| 150mM_NaCl,New_Dongjin - 200mM_NaCl,Chilbo | 1.731963293 | 0.292893183 | 737 | 5.913293289 | 7.72E-07 |
| 200mM_NaCl,New_Dongjin - Mock,Chucheongbyeo | -2.727327266 | 0.284863844 | 737 | -9.574143304 | 0.00E+00 |
| 200mM_NaCl,New_Dongjin - Mock_DMSO,Chucheongbyeo | -2.724949893 | 0.269012658 | 737 | -10.12944859 | 0.00E+00 |
| 200mM_NaCl,New_Dongjin - 1uM_GA3,Chucheongbyeo | -1.90424095 | 0.284896487 | 737 | -6.683974827 | 2.16E-09 |
| 200mM_NaCl,New_Dongjin - 100mM_NaCl,Chucheongbyeo | -0.868789759 | 0.281403435 | 737 | -3.087345964 | 1.64E-01 |
| 200mM_NaCl,New_Dongjin - 150mM_NaCl,Chucheongbyeo | 0.002176406 | 0.284469585 | 737 | 0.007650751 | 1 |
| 200mM_NaCl,New_Dongjin - 200mM_NaCl,Chucheongbyeo | 0.479668743 | 0.29290599 | 737 | 1.637620123 | 0.97650715 |
| 200mM_NaCl,New_Dongjin - Mock,Chilbo | -2.864344476 | 0.28309194 | 737 | -10.11807146 | 0 |
| 200mM_NaCl,New_Dongjin - Mock_DMSO,Chilbo | -2.473548016 | 0.283105289 | 737 | -8.737201704 | 0 |
| 200mM_NaCl,New_Dongjin - 1uM_GA3,Chilbo | -1.564719647 | 0.284865517 | 737 | -5.492836288 | 8.16E-06 |
| 200mM_NaCl,New_Dongjin - 100mM_NaCl,Chilbo | -0.795301614 | 0.281415974 | 737 | -2.826071319 | 0.29876431 |
| 200mM_NaCl,New_Dongjin - 150mM_NaCl,Chilbo | 0.380603158 | 0.279790709 | 737 | 1.360313784 | 0.996858662 |
| 200mM_NaCl,New_Dongjin - 200mM_NaCl,Chilbo | 0.659465048 | 0.28309194 | 737 | 2.329508388 | 0.661445588 |
| Mock,Chucheongbyeo - Mock_DMSO,Chucheongbyeo | 0.002377373 | 0.274188078 | 737 | 0.008670592 | 1 |
| Mock,Chucheongbyeo - 1uM_GA3,Chucheongbyeo | 0.823086316 | 0.289847326 | 737 | 2.839723682 | 2.90E-01 |
| Mock,Chucheongbyeo - 100mM_NaCl,Chucheongbyeo | 1.858537506 | 0.286446796 | 737 | 6.488246787 | 1.94E-08 |
| Mock,Chucheongbyeo - 150mM_NaCl,Chucheongbyeo | 2.729503672 | 0.289239913 | 737 | 9.436815436 | 0.00E+00 |
| Mock,Chucheongbyeo - 200mM_NaCl,Chucheongbyeo | 3.206996009 | 0.297669517 | 737 | 10.7736796 | 0.00E+00 |
| Mock,Chucheongbyeo - Mock,Chilbo | -0.13701721 | 0.2881198 | 737 | -0.475556383 | 1.00E+00 |
| Mock,Chucheongbyeo - Mock_DMSO,Chilbo | 0.25377925 | 0.288162563 | 737 | 0.880680845 | 1.00E+00 |
| Mock,Chucheongbyeo - 1uM_GA3,Chilbo | 1.162607619 | 0.289879923 | 737 | 4.010652436 | 8.28E-03 |
| Mock,Chucheongbyeo - 100mM_NaCl,Chilbo | 1.932025652 | 0.286472397 | 737 | 6.744194802 | 0.00E+00 |
| Mock,Chucheongbyeo - 150mM_NaCl,Chilbo | 3.107930423 | 0.284862108 | 737 | 10.91029779 | 0.00E+00 |
| Mock,Chucheongbyeo - 200mM_NaCl,Chilbo | 3.386792314 | 0.2881198 | 737 | 11.75480584 | 0.00E+00 |
| Mock_DMSO,Chucheongbyeo - 1uM_GA3,Chucheongbyeo | 0.820708943 | 0.274185646 | 737 | 2.99326006 | 2.06E-01 |
| Mock_DMSO,Chucheongbyeo - 100mM_NaCl,Chucheongbyeo | 1.856160133 | 0.270656557 | 737 | 6.857990641 | 0 |
| Mock_DMSO,Chucheongbyeo - 150mM_NaCl,Chucheongbyeo | 2.727126299 | 0.27335681 | 737 | 9.976434457 | 0.00E+00 |
| Mock_DMSO,Chucheongbyeo - 200mM_NaCl,Chucheongbyeo | 3.204618636 | 0.282310446 | 737 | 11.35139945 | 0.00E+00 |
| Mock_DMSO,Chucheongbyeo - Mock,Chilbo | -0.139394583 | 0.272470852 | 737 | -0.511594476 | 0.999999998 |
| Mock_DMSO,Chucheongbyeo - Mock_DMSO,Chilbo | 0.251401877 | 0.272593407 | 737 | 0.922259566 | 0.99998163 |
| Mock_DMSO,Chucheongbyeo - 1uM_GA3,Chilbo | 1.160230246 | 0.274336789 | 737 | 4.229218578 | 3.46E-03 |
| Mock_DMSO,Chucheongbyeo - 100mM_NaCl,Chilbo | 1.929648279 | 0.270741366 | 737 | 7.127275403 | 0.00E+00 |
| Mock_DMSO,Chucheongbyeo - 150mM_NaCl,Chilbo | 3.10555305 | 0.268992786 | 737 | 11.54511649 | 0.00E+00 |
| Mock_DMSO,Chucheongbyeo - 200mM_NaCl,Chilbo | 3.384414941 | 0.272470852 | 737 | 12.42119996 | 0.00E+00 |
| 1uM_GA3,Chucheongbyeo - 100mM_NaCl,Chucheongbyeo | 1.035451191 | 0.286463906 | 737 | 3.614595658 | 3.45E-02 |
| 1uM_GA3,Chucheongbyeo - 150mM_NaCl,Chucheongbyeo | 1.906417356 | 0.288837613 | 737 | 6.600308504 | 7.12E-09 |
| 1uM_GA3,Chucheongbyeo - 200mM_NaCl,Chucheongbyeo | 2.383909693 | 0.297614345 | 737 | 8.010063133 | 0.00E+00 |
| 1uM_GA3,Chucheongbyeo - Mock,Chilbo | -0.960103526 | 0.288137956 | 737 | -3.332096678 | 8.38E-02 |
| 1uM_GA3,Chucheongbyeo - Mock_DMSO,Chilbo | -0.569307065 | 0.28819785 | 737 | -1.975403586 | 8.80E-01 |
| 1uM_GA3,Chucheongbyeo - 1uM_GA3,Chilbo | 0.339521304 | 0.289944339 | 737 | 1.170987867 | 1.00E+00 |
| 1uM_GA3,Chucheongbyeo - 100mM_NaCl,Chilbo | 1.108939336 | 0.286476126 | 737 | 3.870965976 | 1.40E-02 |
| 1uM_GA3,Chucheongbyeo - 150mM_NaCl,Chilbo | 2.284844108 | 0.284865058 | 737 | 8.020794572 | 0.00E+00 |
| 1uM_GA3,Chucheongbyeo - 200mM_NaCl,Chilbo | 2.563705998 | 0.288137956 | 737 | 8.897494919 | 0.00E+00 |
| 100mM_NaCl,Chucheongbyeo - 150mM_NaCl,Chucheongbyeo | 0.870966165 | 0.285854744 | 737 | 3.046883723 | 0.181020174 |
| 100mM_NaCl,Chucheongbyeo - 200mM_NaCl,Chucheongbyeo | 1.348458502 | 0.294400542 | 737 | 4.580353321 | 7.60E-04 |
| 100mM_NaCl,Chucheongbyeo - Mock,Chilbo | -1.995554716 | 0.284695 | 737 | -7.009447707 | 0 |
| 100mM_NaCl,Chucheongbyeo - Mock_DMSO,Chilbo | -1.604758256 | 0.284723323 | 737 | -5.636202336 | 3.72E-06 |
| 100mM_NaCl,Chucheongbyeo - 1uM_GA3,Chilbo | -0.695929887 | 0.286479134 | 737 | -2.429251572 | 5.86E-01 |
| 100mM_NaCl,Chucheongbyeo - 100mM_NaCl,Chilbo | 0.073488146 | 0.283023578 | 737 | 0.259653793 | 1.00E+00 |
| 100mM_NaCl,Chucheongbyeo - 150mM_NaCl,Chilbo | 1.249392917 | 0.281402876 | 737 | 4.439872589 | 1.42E-03 |
| 100mM_NaCl,Chucheongbyeo - 200mM_NaCl,Chilbo | 1.528254807 | 0.284695 | 737 | 5.368042313 | 1.59E-05 |
| 150mM_NaCl,Chucheongbyeo - 200mM_NaCl,Chucheongbyeo | 0.477492337 | 0.29617569 | 737 | 1.612192874 | 0.979890668 |
| 150mM_NaCl,Chucheongbyeo - Mock,Chilbo | -2.866520882 | 0.287537268 | 737 | -9.969215141 | 0 |
| 150mM_NaCl,Chucheongbyeo - Mock_DMSO,Chilbo | -2.475724422 | 0.287794426 | 737 | -8.602405744 | 0 |
| 150mM_NaCl,Chucheongbyeo - 1uM_GA3,Chilbo | -1.566896052 | 0.289923534 | 737 | -5.40451487 | 1.31E-05 |
| 150mM_NaCl,Chucheongbyeo - 100mM_NaCl,Chilbo | -0.79747802 | 0.285688016 | 737 | -2.791429725 | 0.32062951 |
| 150mM_NaCl,Chucheongbyeo - 150mM_NaCl,Chilbo | 0.378426752 | 0.284071809 | 737 | 1.33215173 | 0.997548878 |
| 150mM_NaCl,Chucheongbyeo - 200mM_NaCl,Chilbo | 0.657288642 | 0.287537268 | 737 | 2.285925048 | 0.693426634 |
| 200mM_NaCl,Chucheongbyeo - Mock,Chilbo | -3.344013219 | 0.296051936 | 737 | -11.29536009 | 0 |
| 200mM_NaCl,Chucheongbyeo - Mock_DMSO,Chilbo | -2.953216759 | 0.296163929 | 737 | -9.971561268 | 0 |
| 200mM_NaCl,Chucheongbyeo - 1uM_GA3,Chilbo | -2.04438839 | 0.297858583 | 737 | -6.863620887 | 0.00E+00 |
| 200mM_NaCl,Chucheongbyeo - 100mM_NaCl,Chilbo | -1.274970357 | 0.294426708 | 737 | -4.330348855 | 0.002268228 |
| 200mM_NaCl,Chucheongbyeo - 150mM_NaCl,Chilbo | -0.099065585 | 0.292837611 | 737 | -0.338295293 | 1 |
| 200mM_NaCl,Chucheongbyeo - 200mM_NaCl,Chilbo | 0.179796305 | 0.296051936 | 737 | 0.607313391 | 0.999999968 |
| Mock,Chilbo - Mock_DMSO,Chilbo | 0.39079646 | 0.286375113 | 737 | 1.364631361 | 0.996739479 |
| Mock,Chilbo - 1uM_GA3,Chilbo | 1.299624829 | 0.288137013 | 737 | 4.510440421 | 1.04E-03 |
| Mock,Chilbo - 100mM_NaCl,Chilbo | 2.069042862 | 0.284695158 | 737 | 7.267573061 | 0 |
| Mock,Chilbo - 150mM_NaCl,Chilbo | 3.244947633 | 0.28309247 | 737 | 11.46250068 | 0 |
| Mock,Chilbo - 200mM_NaCl,Chilbo | 3.523809524 | 0.286360502 | 737 | 12.30550127 | 0 |
| Mock_DMSO,Chilbo - 1uM_GA3,Chilbo | 0.908828369 | 0.28813289 | 737 | 3.154198641 | 1.38E-01 |
| Mock_DMSO,Chilbo - 100mM_NaCl,Chilbo | 1.678246402 | 0.284711309 | 737 | 5.89455476 | 8.61E-07 |
| Mock_DMSO,Chilbo - 150mM_NaCl,Chilbo | 2.854151173 | 0.283122071 | 737 | 10.08099143 | 0 |
| Mock_DMSO,Chilbo - 200mM_NaCl,Chilbo | 3.133013064 | 0.286375113 | 737 | 10.94024209 | 0 |
| 1uM_GA3,Chilbo - 100mM_NaCl,Chilbo | 0.769418033 | 0.28650572 | 737 | 2.68552416 | 3.92E-01 |
| 1uM_GA3,Chilbo - 150mM_NaCl,Chilbo | 1.945322804 | 0.284910017 | 737 | 6.827849806 | 0.00E+00 |
| 1uM_GA3,Chilbo - 200mM_NaCl,Chilbo | 2.224184695 | 0.288137013 | 737 | 7.719191204 | 0.00E+00 |
| 100mM_NaCl,Chilbo - 150mM_NaCl,Chilbo | 1.175904771 | 0.281402724 | 737 | 4.178725621 | 0.004255308 |
| 100mM_NaCl,Chilbo - 200mM_NaCl,Chilbo | 1.454766662 | 0.284695158 | 737 | 5.109910092 | 6.04E-05 |
| 150mM_NaCl,Chilbo - 200mM_NaCl,Chilbo | 0.27886189 | 0.28309247 | 737 | 0.985055837 | 0.999953029 |

Crown Root Length (mm)

| contrast | estimate | SE | df | t.ratio | p.value |
| --- | --- | --- | --- | --- | --- |
| Mock,New_Dongjin - Mock_DMSO,New_Dongjin | 1.797128233 | 0.932431936 | 789 | 1.927355943 | 0.900766725 |
| Mock,New_Dongjin - 1uM_GA3,New_Dongjin | 3.17013649 | 0.960534899 | 789 | 3.30038658 | 0.091745011 |
| Mock,New_Dongjin - 100mM_NaCl,New_Dongjin | -0.969888863 | 0.96360143 | 789 | -1.006524931 | 0.999936677 |
| Mock,New_Dongjin - 150mM_NaCl,New_Dongjin | -0.334687982 | 0.994521381 | 789 | -0.336531711 | 1 |
| Mock,New_Dongjin - 200mM_NaCl,New_Dongjin | 11.37337268 | 1.00678179 | 789 | 11.29676042 | 0 |
| Mock,New_Dongjin - Mock,Chucheongbyeo | -3.721012477 | 0.964673219 | 789 | -3.857277679 | 0.014687093 |
| Mock,New_Dongjin - Mock_DMSO,Chucheongbyeo | -3.210721881 | 0.936392142 | 789 | -3.428821898 | 0.062566464 |
| Mock,New_Dongjin - 1uM_GA3,Chucheongbyeo | -0.756039194 | 0.987717656 | 789 | -0.765440598 | 0.999998846 |
| Mock,New_Dongjin - 100mM_NaCl,Chucheongbyeo | -6.141022064 | 0.98102248 | 789 | -6.259817881 | 8.90E-08 |
| Mock,New_Dongjin - 150mM_NaCl,Chucheongbyeo | -2.552324341 | 1.022982598 | 789 | -2.494983145 | 0.534884187 |
| Mock,New_Dongjin - 200mM_NaCl,Chucheongbyeo | 7.540326647 | 1.097784467 | 789 | 6.868676755 | 0 |
| Mock,New_Dongjin - Mock,Chilbo | 2.127492313 | 0.916693698 | 789 | 2.320832265 | 0.667898344 |
| Mock,New_Dongjin - Mock_DMSO,Chilbo | 3.379410623 | 0.926293516 | 789 | 3.6483151 | 0.030740571 |
| Mock,New_Dongjin - 1uM_GA3,Chilbo | 5.676881274 | 0.947470948 | 789 | 5.991615136 | 4.72E-07 |
| Mock,New_Dongjin - 100mM_NaCl,Chilbo | 0.529498791 | 1.063358303 | 789 | 0.497949552 | 0.999999999 |
| Mock,New_Dongjin - 150mM_NaCl,Chilbo | 1.771986829 | 0.968147225 | 789 | 1.830286533 | 0.934860895 |
| Mock,New_Dongjin - 200mM_NaCl,Chilbo | 8.606625685 | 1.009038298 | 789 | 8.52953322 | 0 |
| Mock_DMSO,New_Dongjin - 1uM_GA3,New_Dongjin | 1.373008258 | 0.933771986 | 789 | 1.470389214 | 0.992389211 |
| Mock_DMSO,New_Dongjin - 100mM_NaCl,New_Dongjin | -2.767017096 | 0.937151104 | 789 | -2.952583724 | 0.226404275 |
| Mock_DMSO,New_Dongjin - 150mM_NaCl,New_Dongjin | -2.131816215 | 0.965404047 | 789 | -2.208211392 | 0.747803352 |
| Mock_DMSO,New_Dongjin - 200mM_NaCl,New_Dongjin | 9.576244443 | 0.98157398 | 789 | 9.756008854 | 0 |
| Mock_DMSO,New_Dongjin - Mock,Chucheongbyeo | -5.51814071 | 0.938191701 | 789 | -5.881677174 | 9.01E-07 |
| Mock_DMSO,New_Dongjin - Mock_DMSO,Chucheongbyeo | -5.007850114 | 0.907217163 | 789 | -5.520012538 | 6.89E-06 |
| Mock_DMSO,New_Dongjin - 1uM_GA3,Chucheongbyeo | -2.553167427 | 0.959873648 | 789 | -2.659899491 | 0.410664025 |
| Mock_DMSO,New_Dongjin - 100mM_NaCl,Chucheongbyeo | -7.938150297 | 0.955149425 | 789 | -8.310898895 | 0 |
| Mock_DMSO,New_Dongjin - 150mM_NaCl,Chucheongbyeo | -4.349452574 | 0.994726909 | 789 | -4.372509211 | 0.001880424 |
| Mock_DMSO,New_Dongjin - 200mM_NaCl,Chucheongbyeo | 5.743198414 | 1.072634854 | 789 | 5.354290317 | 1.68E-05 |
| Mock_DMSO,New_Dongjin - Mock,Chilbo | 0.33036408 | 0.888739015 | 789 | 0.371722265 | 1 |
| Mock_DMSO,New_Dongjin - Mock_DMSO,Chilbo | 1.58228239 | 0.898835701 | 789 | 1.760368873 | 0.953646515 |
| Mock_DMSO,New_Dongjin - 1uM_GA3,Chilbo | 3.879753041 | 0.921154314 | 789 | 4.211838323 | 0.003692645 |
| Mock_DMSO,New_Dongjin - 100mM_NaCl,Chilbo | -1.267629442 | 1.037125631 | 789 | -1.222252545 | 0.999153635 |
| Mock_DMSO,New_Dongjin - 150mM_NaCl,Chilbo | -0.025141404 | 0.941798708 | 789 | -0.026695093 | 1 |
| Mock_DMSO,New_Dongjin - 200mM_NaCl,Chilbo | 6.809497452 | 0.983578086 | 789 | 6.923189476 | 0 |
| 1uM_GA3,New_Dongjin - 100mM_NaCl,New_Dongjin | -4.140025353 | 0.965076925 | 789 | -4.289839747 | 0.002670887 |
| 1uM_GA3,New_Dongjin - 150mM_NaCl,New_Dongjin | -3.504824472 | 0.992266322 | 789 | -3.532140912 | 0.045166247 |
| 1uM_GA3,New_Dongjin - 200mM_NaCl,New_Dongjin | 8.203236186 | 1.008287209 | 789 | 8.135813004 | 0 |
| 1uM_GA3,New_Dongjin - Mock,Chucheongbyeo | -6.891148967 | 0.966063814 | 789 | -7.133223358 | 0 |
| 1uM_GA3,New_Dongjin - Mock_DMSO,Chucheongbyeo | -6.380858371 | 0.935698661 | 789 | -6.819351828 | 0 |
| 1uM_GA3,New_Dongjin - 1uM_GA3,Chucheongbyeo | -3.926175684 | 0.986888356 | 789 | -3.978338239 | 0.009327742 |
| 1uM_GA3,New_Dongjin - 100mM_NaCl,Chucheongbyeo | -9.311158555 | 0.982569692 | 789 | -9.476333978 | 0 |
| 1uM_GA3,New_Dongjin - 150mM_NaCl,Chucheongbyeo | -5.722460832 | 1.020815454 | 789 | -5.6057741 | 4.30E-06 |
| 1uM_GA3,New_Dongjin - 200mM_NaCl,Chucheongbyeo | 4.370190156 | 1.096870956 | 789 | 3.984233635 | 0.009119489 |
| 1uM_GA3,New_Dongjin - Mock,Chilbo | -1.042644178 | 0.918126892 | 789 | -1.135620999 | 0.999673218 |
| 1uM_GA3,New_Dongjin - Mock_DMSO,Chilbo | 0.209274132 | 0.927940428 | 789 | 0.225525396 | 1 |
| 1uM_GA3,New_Dongjin - 1uM_GA3,Chilbo | 2.506744784 | 0.949566519 | 789 | 2.639883288 | 0.425234455 |
| 1uM_GA3,New_Dongjin - 100mM_NaCl,Chilbo | -2.640637699 | 1.062240467 | 789 | -2.485913296 | 0.541888526 |
| 1uM_GA3,New_Dongjin - 150mM_NaCl,Chilbo | -1.398149662 | 0.969581985 | 789 | -1.44201283 | 0.993871984 |
| 1uM_GA3,New_Dongjin - 200mM_NaCl,Chilbo | 5.436489195 | 1.010205725 | 789 | 5.38156641 | 1.45E-05 |
| 100mM_NaCl,New_Dongjin - 150mM_NaCl,New_Dongjin | 0.635200881 | 0.996578332 | 789 | 0.63738179 | 0.999999932 |
| 100mM_NaCl,New_Dongjin - 200mM_NaCl,New_Dongjin | 12.34326154 | 1.011324277 | 789 | 12.20504819 | 0 |
| 100mM_NaCl,New_Dongjin - Mock,Chucheongbyeo | -2.751123614 | 0.969248036 | 789 | -2.838410305 | 0.291014138 |
| 100mM_NaCl,New_Dongjin - Mock_DMSO,Chucheongbyeo | -2.240833018 | 0.939435468 | 789 | -2.385297442 | 0.619380697 |
| 100mM_NaCl,New_Dongjin - 1uM_GA3,Chucheongbyeo | 0.213849669 | 0.99058948 | 789 | 0.215881224 | 1 |
| 100mM_NaCl,New_Dongjin - 100mM_NaCl,Chucheongbyeo | -5.171133201 | 0.985666705 | 789 | -5.246330404 | 2.96E-05 |
| 100mM_NaCl,New_Dongjin - 150mM_NaCl,Chucheongbyeo | -1.582435478 | 1.024992513 | 789 | -1.543850768 | 0.98713362 |
| 100mM_NaCl,New_Dongjin - 200mM_NaCl,Chucheongbyeo | 8.510215509 | 1.10026854 | 789 | 7.734671313 | 0 |
| 100mM_NaCl,New_Dongjin - Mock,Chilbo | 3.097381175 | 0.9215109 | 789 | 3.361198632 | 0.076778004 |
| 100mM_NaCl,New_Dongjin - Mock_DMSO,Chilbo | 4.349299485 | 0.931245074 | 789 | 4.670413414 | 0.000498795 |
| 100mM_NaCl,New_Dongjin - 1uM_GA3,Chilbo | 6.646770137 | 0.952567611 | 789 | 6.977741066 | 0 |
| 100mM_NaCl,New_Dongjin - 100mM_NaCl,Chilbo | 1.499387654 | 1.065932979 | 789 | 1.40664346 | 0.995375793 |
| 100mM_NaCl,New_Dongjin - 150mM_NaCl,Chilbo | 2.741875691 | 0.972756262 | 789 | 2.818666707 | 0.303197512 |
| 100mM_NaCl,New_Dongjin - 200mM_NaCl,Chilbo | 9.576514548 | 1.013334032 | 789 | 9.45050126 | 0 |
| 150mM_NaCl,New_Dongjin - 200mM_NaCl,New_Dongjin | 11.70806066 | 1.038999855 | 789 | 11.26858739 | 0 |
| 150mM_NaCl,New_Dongjin - Mock,Chucheongbyeo | -3.386324495 | 0.997036178 | 789 | -3.396390791 | 0.069079524 |
| 150mM_NaCl,New_Dongjin - Mock_DMSO,Chucheongbyeo | -2.876033899 | 0.956000662 | 789 | -3.008401577 | 0.19856909 |
| 150mM_NaCl,New_Dongjin - 1uM_GA3,Chucheongbyeo | -0.421351212 | 1.005514307 | 789 | -0.419040494 | 1 |
| 150mM_NaCl,New_Dongjin - 100mM_NaCl,Chucheongbyeo | -5.806334082 | 1.014027512 | 789 | -5.726012377 | 2.20E-06 |
| 150mM_NaCl,New_Dongjin - 150mM_NaCl,Chucheongbyeo | -2.217636359 | 1.03203608 | 789 | -2.148797316 | 0.786386216 |
| 150mM_NaCl,New_Dongjin - 200mM_NaCl,Chucheongbyeo | 7.875014629 | 1.112809598 | 789 | 7.076695461 | 0 |
| 150mM_NaCl,New_Dongjin - Mock,Chilbo | 2.462180295 | 0.950567755 | 789 | 2.590220719 | 0.462121801 |
| 150mM_NaCl,New_Dongjin - Mock_DMSO,Chilbo | 3.714098604 | 0.961302225 | 789 | 3.863611783 | 0.014348992 |
| 150mM_NaCl,New_Dongjin - 1uM_GA3,Chilbo | 6.011569256 | 0.984517175 | 789 | 6.106109077 | 2.36E-07 |
| 150mM_NaCl,New_Dongjin - 100mM_NaCl,Chilbo | 0.864186773 | 1.078095709 | 789 | 0.801586321 | 0.999997683 |
| 150mM_NaCl,New_Dongjin - 150mM_NaCl,Chilbo | 2.10667481 | 1.000742908 | 789 | 2.105110906 | 0.812814518 |
| 150mM_NaCl,New_Dongjin - 200mM_NaCl,Chilbo | 8.941313667 | 1.039099052 | 789 | 8.604871355 | 0 |
| 200mM_NaCl,New_Dongjin - Mock,Chucheongbyeo | -15.09438515 | 1.012311248 | 789 | -14.91081441 | 0 |
| 200mM_NaCl,New_Dongjin - Mock_DMSO,Chucheongbyeo | -14.58409456 | 0.984411436 | 789 | -14.81503976 | 0 |
| 200mM_NaCl,New_Dongjin - 1uM_GA3,Chucheongbyeo | -12.12941187 | 1.033244038 | 789 | -11.73915496 | 0 |
| 200mM_NaCl,New_Dongjin - 100mM_NaCl,Chucheongbyeo | -17.51439474 | 1.027985905 | 789 | -17.03758258 | 0 |
| 200mM_NaCl,New_Dongjin - 150mM_NaCl,Chucheongbyeo | -13.92569702 | 1.066288617 | 789 | -13.05996969 | 0 |
| 200mM_NaCl,New_Dongjin - 200mM_NaCl,Chucheongbyeo | -3.83304603 | 1.13883302 | 789 | -3.365766502 | 0.0757403 |
| 200mM_NaCl,New_Dongjin - Mock,Chilbo | -9.245880363 | 0.966678125 | 789 | -9.564590448 | 0 |
| 200mM_NaCl,New_Dongjin - Mock_DMSO,Chilbo | -7.993962054 | 0.97589123 | 789 | -8.191447788 | 0 |
| 200mM_NaCl,New_Dongjin - 1uM_GA3,Chilbo | -5.696491402 | 0.996272059 | 789 | -5.717807047 | 2.30E-06 |
| 200mM_NaCl,New_Dongjin - 100mM_NaCl,Chilbo | -10.84387389 | 1.105566388 | 789 | -9.808433033 | 0 |
| 200mM_NaCl,New_Dongjin - 150mM_NaCl,Chilbo | -9.601385848 | 1.015642211 | 789 | -9.453512019 | 0 |
| 200mM_NaCl,New_Dongjin - 200mM_NaCl,Chilbo | -2.766746991 | 1.054586296 | 789 | -2.623537781 | 0.437266691 |
| Mock,Chucheongbyeo - Mock_DMSO,Chucheongbyeo | 0.510290596 | 0.93958447 | 789 | 0.543102416 | 0.999999995 |
| Mock,Chucheongbyeo - 1uM_GA3,Chucheongbyeo | 2.964973283 | 0.990913881 | 789 | 2.992160409 | 0.206411301 |
| Mock,Chucheongbyeo - 100mM_NaCl,Chucheongbyeo | -2.420009587 | 0.986641833 | 789 | -2.452774154 | 0.567500906 |
| Mock,Chucheongbyeo - 150mM_NaCl,Chucheongbyeo | 1.168688136 | 1.025427484 | 789 | 1.139708223 | 0.999657352 |
| Mock,Chucheongbyeo - 200mM_NaCl,Chucheongbyeo | 11.26133912 | 1.100561297 | 789 | 10.23235976 | 0 |
| Mock,Chucheongbyeo - Mock,Chilbo | 5.84850479 | 0.922518504 | 789 | 6.339715426 | 5.17E-08 |
| Mock,Chucheongbyeo - Mock_DMSO,Chilbo | 7.100423099 | 0.932336825 | 789 | 7.615727391 | 0 |
| Mock,Chucheongbyeo - 1uM_GA3,Chilbo | 9.397893751 | 0.953559128 | 789 | 9.855596233 | 0 |
| Mock,Chucheongbyeo - 100mM_NaCl,Chilbo | 4.250511268 | 1.066471205 | 789 | 3.985584654 | 0.009072368 |
| Mock,Chucheongbyeo - 150mM_NaCl,Chilbo | 5.492999305 | 0.973710559 | 789 | 5.641306092 | 3.53E-06 |
| Mock,Chucheongbyeo - 200mM_NaCl,Chilbo | 12.32763816 | 1.014247545 | 789 | 12.15446684 | 0 |
| Mock_DMSO,Chucheongbyeo - 1uM_GA3,Chucheongbyeo | 2.454682687 | 0.933790057 | 789 | 2.628730802 | 0.433431621 |
| Mock_DMSO,Chucheongbyeo - 100mM_NaCl,Chucheongbyeo | -2.930300183 | 0.957651539 | 789 | -3.059881453 | 0.175097782 |
| Mock_DMSO,Chucheongbyeo - 150mM_NaCl,Chucheongbyeo | 0.65839754 | 0.985529977 | 789 | 0.668064448 | 0.999999858 |
| Mock_DMSO,Chucheongbyeo - 200mM_NaCl,Chucheongbyeo | 10.75104853 | 1.05169887 | 789 | 10.22255404 | 0 |
| Mock_DMSO,Chucheongbyeo - Mock,Chilbo | 5.338214194 | 0.879633453 | 789 | 6.068680283 | 2.97E-07 |
| Mock_DMSO,Chucheongbyeo - Mock_DMSO,Chilbo | 6.590132503 | 0.89235557 | 789 | 7.385097069 | 0 |
| Mock_DMSO,Chucheongbyeo - 1uM_GA3,Chilbo | 8.887603155 | 0.910530104 | 789 | 9.760910836 | 0 |
| Mock_DMSO,Chucheongbyeo - 100mM_NaCl,Chilbo | 3.740220672 | 1.011447539 | 789 | 3.697888946 | 0.025936925 |
| Mock_DMSO,Chucheongbyeo - 150mM_NaCl,Chilbo | 4.982708709 | 0.930816505 | 789 | 5.35305152 | 1.69E-05 |
| Mock_DMSO,Chucheongbyeo - 200mM_NaCl,Chilbo | 11.81734757 | 0.977415559 | 789 | 12.09040254 | 0 |
| 1uM_GA3,Chucheongbyeo - 100mM_NaCl,Chucheongbyeo | -5.38498287 | 1.007917025 | 789 | -5.342684701 | 1.79E-05 |
| 1uM_GA3,Chucheongbyeo - 150mM_NaCl,Chucheongbyeo | -1.796285147 | 1.033684505 | 789 | -1.737749903 | 0.958772668 |
| 1uM_GA3,Chucheongbyeo - 200mM_NaCl,Chucheongbyeo | 8.29636584 | 1.093951081 | 789 | 7.583854509 | 0 |
| 1uM_GA3,Chucheongbyeo - Mock,Chilbo | 2.883531507 | 0.930562796 | 789 | 3.098696313 | 0.158777251 |
| 1uM_GA3,Chucheongbyeo - Mock_DMSO,Chilbo | 4.135449816 | 0.942757791 | 789 | 4.386545363 | 0.001770305 |
| 1uM_GA3,Chucheongbyeo - 1uM_GA3,Chilbo | 6.432920468 | 0.959239782 | 789 | 6.706269475 | 0 |
| 1uM_GA3,Chucheongbyeo - 100mM_NaCl,Chilbo | 1.285537985 | 1.052250864 | 789 | 1.22170295 | 0.999158445 |
| 1uM_GA3,Chucheongbyeo - 150mM_NaCl,Chilbo | 2.528026022 | 0.978337864 | 789 | 2.584001003 | 0.466805477 |
| 1uM_GA3,Chucheongbyeo - 200mM_NaCl,Chilbo | 9.362664879 | 1.024145744 | 789 | 9.141926266 | 0 |
| 100mM_NaCl,Chucheongbyeo - 150mM_NaCl,Chucheongbyeo | 3.588697723 | 1.041961638 | 789 | 3.444174518 | 0.059668282 |
| 100mM_NaCl,Chucheongbyeo - 200mM_NaCl,Chucheongbyeo | 13.68134871 | 1.115919794 | 789 | 12.26015416 | 0 |
| 100mM_NaCl,Chucheongbyeo - Mock,Chilbo | 8.268514377 | 0.939795552 | 789 | 8.798205485 | 0 |
| 100mM_NaCl,Chucheongbyeo - Mock_DMSO,Chilbo | 9.520432687 | 0.949312626 | 789 | 10.02876442 | 0 |
| 100mM_NaCl,Chucheongbyeo - 1uM_GA3,Chilbo | 11.81790334 | 0.970160733 | 789 | 12.181387 | 0 |
| 100mM_NaCl,Chucheongbyeo - 100mM_NaCl,Chilbo | 6.670520855 | 1.082119856 | 789 | 6.164308713 | 1.64E-07 |
| 100mM_NaCl,Chucheongbyeo - 150mM_NaCl,Chilbo | 7.913008893 | 0.990083212 | 789 | 7.992266509 | 0 |
| 100mM_NaCl,Chucheongbyeo - 200mM_NaCl,Chilbo | 14.74764775 | 1.03001021 | 789 | 14.31796268 | 0 |
| 150mM_NaCl,Chucheongbyeo - 200mM_NaCl,Chucheongbyeo | 10.09265099 | 1.138339062 | 789 | 8.86612023 | 0 |
| 150mM_NaCl,Chucheongbyeo - Mock,Chilbo | 4.679816654 | 0.980318229 | 789 | 4.773772959 | 0.000307874 |
| 150mM_NaCl,Chucheongbyeo - Mock_DMSO,Chilbo | 5.931734964 | 0.990737307 | 789 | 5.987192489 | 4.84E-07 |
| 150mM_NaCl,Chucheongbyeo - 1uM_GA3,Chilbo | 8.229205615 | 1.013228897 | 789 | 8.12176364 | 0 |
| 150mM_NaCl,Chucheongbyeo - 100mM_NaCl,Chilbo | 3.081823132 | 1.104495457 | 789 | 2.790254239 | 0.321225437 |
| 150mM_NaCl,Chucheongbyeo - 150mM_NaCl,Chilbo | 4.32431117 | 1.029038633 | 789 | 4.202282625 | 0.003840265 |
| 150mM_NaCl,Chucheongbyeo - 200mM_NaCl,Chilbo | 11.15895003 | 1.066390725 | 789 | 10.46422269 | 0 |
| 200mM_NaCl,Chucheongbyeo - Mock,Chilbo | -5.412834334 | 1.048601395 | 789 | -5.161956069 | 4.56E-05 |
| 200mM_NaCl,Chucheongbyeo - Mock_DMSO,Chilbo | -4.160916024 | 1.059269345 | 789 | -3.928100105 | 0.011286865 |
| 200mM_NaCl,Chucheongbyeo - 1uM_GA3,Chilbo | -1.863445373 | 1.074939987 | 789 | -1.733534332 | 0.959679509 |
| 200mM_NaCl,Chucheongbyeo - 100mM_NaCl,Chilbo | -7.010827856 | 1.159360775 | 789 | -6.047149435 | 3.38E-07 |
| 200mM_NaCl,Chucheongbyeo - 150mM_NaCl,Chilbo | -5.768339818 | 1.091722727 | 789 | -5.283704074 | 2.43E-05 |
| 200mM_NaCl,Chucheongbyeo - 200mM_NaCl,Chilbo | 1.066299038 | 1.131989064 | 789 | 0.941969381 | 0.999975203 |
| Mock,Chilbo - Mock_DMSO,Chilbo | 1.25191831 | 0.874616259 | 789 | 1.431391535 | 0.994361168 |
| Mock,Chilbo - 1uM_GA3,Chilbo | 3.549388961 | 0.893914031 | 789 | 3.970615561 | 0.009607126 |
| Mock,Chilbo - 100mM_NaCl,Chilbo | -1.597993522 | 1.007443664 | 789 | -1.586186482 | 0.982969513 |
| Mock,Chilbo - 150mM_NaCl,Chilbo | -0.355505484 | 0.9157948 | 789 | -0.388193386 | 1 |
| Mock,Chilbo - 200mM_NaCl,Chilbo | 6.479133372 | 0.962552508 | 789 | 6.731199931 | 0 |
| Mock_DMSO,Chilbo - 1uM_GA3,Chilbo | 2.297470652 | 0.904926666 | 789 | 2.538847331 | 0.501138544 |
| Mock_DMSO,Chilbo - 100mM_NaCl,Chilbo | -2.849911831 | 1.018796254 | 789 | -2.79733246 | 0.316680641 |
| Mock_DMSO,Chilbo - 150mM_NaCl,Chilbo | -1.607423794 | 0.926640597 | 789 | -1.734678795 | 0.959434798 |
| Mock_DMSO,Chilbo - 200mM_NaCl,Chilbo | 5.227215063 | 0.972553531 | 789 | 5.374732493 | 1.51E-05 |
| 1uM_GA3,Chilbo - 100mM_NaCl,Chilbo | -5.147382483 | 1.033852683 | 789 | -4.97883554 | 0.000114442 |
| 1uM_GA3,Chilbo - 150mM_NaCl,Chilbo | -3.904894446 | 0.944014107 | 789 | -4.136478911 | 0.005015634 |
| 1uM_GA3,Chilbo - 200mM_NaCl,Chilbo | 2.929744411 | 0.990791051 | 789 | 2.956975043 | 0.224124163 |
| 100mM_NaCl,Chilbo - 150mM_NaCl,Chilbo | 1.242488037 | 1.051143174 | 789 | 1.182035015 | 0.999448439 |
| 100mM_NaCl,Chilbo - 200mM_NaCl,Chilbo | 8.077126894 | 1.094959718 | 789 | 7.376642957 | 0 |
| 150mM_NaCl,Chilbo - 200mM_NaCl,Chilbo | 6.834638857 | 1.010334437 | 789 | 6.764729191 | 0 |
