# Supplementary material for: Development of a novel and rapid phenotype-based screening method to assess rice seedling growth
Source: Plant Methods. 2020 Oct 15;16:139. doi: 10.1186/s13007-020-00682-6 (PMC7560306; doi:10.1186/s13007-020-00682-6)
Supplement: Supplementary file 14 — Additional file 14: Figure S12. Illustration of the use of Plength in another cereal crop, such as wheat. [file 13007_2020_682_MOESM14_ESM.pdf]

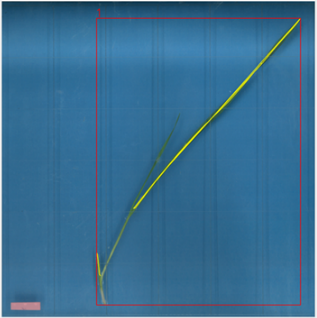


```
Calibration: 20mm=306 pixels; 1 pixel=0.065mm
#1, Length: 254.22;
    Internode 1: 71.32;
    Leaf 1: 33.05;
    Leaf 2: 178.75
==============================
Length: Mean, 254.22
    SD, nan
    SE, nan
Internode 1: Mean, 71.32
    SD, nan
    SE, nan
Leaf 1: Mean, 33.05
    SD, nan
    SE, nan
Leaf 2: Mean, 178.75
    SD, nan
    SE, nan
==============================
```
