# Supplementary material for: Development of a novel and rapid phenotype-based screening method to assess rice seedling growth
Source: Plant Methods. 2020 Oct 15;16:139. doi: 10.1186/s13007-020-00682-6 (PMC7560306; doi:10.1186/s13007-020-00682-6)
Supplement: Supplementary file 15 — Additional file 15. Python source code used to make Plength. [file 13007_2020_682_MOESM15_ESM.docx]

Additional file 15: Source code Plength

**import** numpy **as** np **import** networkx **as** nx **import** cv2

**import** sknw

**from** skimage**.**morphology **import** skeletonize

**from** scipy **import** stats

**from** tkinter **import * from** tkinter **import** font

**from** tkinter **import** filedialog

**from** tkinter **import** ttk

**from** PIL **import** Image**,** ImageTk

**from** os **import** path

**from** sys **import** stdout

**class Window(**Frame**):**

**def** init **(***self***,** master**=None):** Frame**.** init **(***self***,** master**)** *self***.**master **=** master *self***.**init_window**()**

**def** init_window**(***self***):**

'''Creates the main window for the program'''

*self***.**master**.**title**(**"PLENGTH"**)** *self***.**pack**(**fill**=**BOTH**,** expand**=**1**)**

# Initialization # General stuff

*self***.**filepath **= None** *self***.**disp **= None** *self***.**analyzed **= False** *self***.**cropping **= False** *self***.**thresholding **= False** *self***.**grouping **= False**

# Editable

*self***.**c_entry **=** 20

*self***.**min_area **=** 500

*self***.**plant_label **=** StringVar**()** *self***.**plant_label**.**set**(**"seedling"**)** *self***.**plant **=** *self***.**plant_label**.**get**()**

*self***.**hue_lower**,** *self***.**hue_upper **=** 0**,** 95

*self***.**sat_lower**,** *self***.**sat_upper **=** 0**,** 255

*self***.**val_lower**,** *self***.**val_upper **=** 0**,** 255

*self***.**side_label **=** StringVar**()** *self***.**side_label**.**set**(**"right"**)** *self***.**side **=** *self***.**side_label**.**get**()**

# Text

*self***.**imagename **=** StringVar**()**

*self***.**imagename_label **=** Label**(***self***,** textvariable**=***self***.**imagename**)**

*self***.**imagesize **=** StringVar**()**

*self***.**imagesize_label **=** Label**(***self***,** textvariable**=***self***.**imagesize**)**

*self***.**exported **=** StringVar**()**

*self***.**exported_label **=** Label**(***self***,** textvariable**=***self***.**exported**)**

# Buttons

uploadImage **=** Button**(***self***,** text**=**"Upload Image"**,** command**=***self***.**askopenfile**)** crop **=** Button**(***self***,** text**=**"Crop"**,** command**=***self***.**cropImgStart**)**

threshold **=** Button**(***self***,** text**=**"Threshold"**,** command**=***self***.**thresholdStart**)** start **=** Button**(***self***,** text**=**"Start"**,** command**=***self***.**analyze**)**

Button**(***self***,**text**=**"Quit"**,** command**=***self***.**master**.**quit**).**grid**(**\

column**=**0**,** row**=**2**,** padx**=(**20**,**10**),** pady**=(**83**,** 0**),** sticky**=(**W**+**E**,** N**))**

# Layout

uploadImage**.**grid**(**row**=**0**,** sticky**=**W**+**E**,** padx**=(**20**,**10**),** pady**=(**20**,**10**))**

*self***.**imagename_label**.**grid**(**row**=**0**,** column**=**1**,** sticky**=**W**,** padx**=**10**,**\

pady**=(**20**,**10**),** columnspan**=**7**)** crop**.**grid**(**row**=**1**,** sticky**=(**W**+**E**,** N**),** padx**=(**20**,**10**))** threshold**.**grid**(**row**=**2**,** sticky**=(**W**+**E**,** N**),** padx**=(**20**,**10**),** pady**=(**10**,**0**))** *self***.**imagesize_label**.**grid**(**row**=**1**,** column**=**1**,** sticky**=**W**,** padx**=**10**)** start**.**grid**(**row**=**2**,** sticky**=(**W**+**E**,** N**),** padx**=(**20**,**10**),** pady**=(**47**,**0**))**

*self***.**exported_label**.**grid**(**row**=**5**,** column**=**2**,** padx**=**20**,** pady**=(**5**,**15**),** sticky**=**E**)**

# Menu

menu **=** Menu**(***self***.**master**,** tearoff**=False)**

*self***.**master**.**config**(**menu**=**menu**)**

# File

file **=** Menu**(**menu**,** tearoff**=False)**

file**.**add_command**(**label**=**"Upload Image"**,** command**=***self***.**askopenfile**)** file**.**add_command**(**label**=**"Start"**,** command**=***self***.**analyze**)** file**.**add_command**(**label**=**"Exit"**,** command**=***self***.**master**.**quit**)** menu**.**add_cascade**(**label**=**"File"**,** menu**=**file**)**

# Edit

edit **=** Menu**(**menu**,** tearoff**=False)** edit**.**add_command**(**label**=**"Crop"**,** command**=***self***.**cropImgStart**)**

edit**.**add_command**(**label**=**"Threshold"**,** command**=***self***.**thresholdStart**)** edit**.**add_command**(**label**=**"Settings"**,** command**=***self***.**calibrate**)** menu**.**add_cascade**(**label**=**"Edit"**,** menu**=**edit**)**

# Help

helpmenu **=** Menu**(**menu**,** tearoff**=False)** helpmenu**.**add_command**(**label**=**"Guidelines"**,** command**=***self***.**guideline**)** helpmenu**.**add_command**(**label**=**"About"**,** command**=***self***.**about**)** menu**.**add_cascade**(**label**=**"Help"**,** menu**=**helpmenu**)**

**def** askopenfile**(***self***):**

'''Opens file dialog, gets the file directory, and shows the image'''

**if** *self***.**cropping **or** *self***.**thresholding**: return None**

filepath **=** filedialog**.**askopenfilename**(**filetypes**=((**"JPEG"**,** "*.jpg"**),**\

**(**"PNG"**,** "*.png"**)))**

**if** filepath**:**

*self***.**filepath **=** filepath

# If it has been analyzed, reset things

**if** *self***.**analyzed**:** *self***.**reset**(**full**=False)**

# More initialization

*self***.**img **=** cv2**.**imread**(***self***.**filepath**)**

*self***.**height**,** *self***.**width**,** _ **=** *self***.**img**.**shape

# Show image, file path, and file size load **=** Image**.**open**(***self***.**filepath**)** *self***.**showImg**(**load**)** *self***.**imagename**.**set**(***self***.**filepath**)**

*self***.**imagesize**.**set**(**"Image size: {} x {}"**.**format**(***self***.**width**,** *self***.**height**))**

**def** showImg**(***self***,** load**):** '''Shows the image'''

height**,** width **=** *self***.**height**,** *self***.**width times **=** 0

# Reduces the width until it is maximum 460 pixels

**while** width **>=** 460**:** width **=** width**//**2 times **+=** 1

# Maintain aspect ratio with this height **=** height**//(**2******times**)**

disp **=** load**.**resize**((**width**,** height**),** Image**.**ANTIALIAS**)** render **=** ImageTk**.**PhotoImage**(**disp**)**

# If picture already exists, destroy it

**if** *self***.**disp**:**

*self***.**disp**.**destroy**()**

# Finally show the image

*self***.**disp **=** Label**(***self***,** image**=**render**)**

*self***.**disp**.**image **=**r ender

*self***.**disp**.**grid**(**row**=**2**,** column**=**1**,** padx**=**10**,** pady**=**10**,** sticky**=**N**+**W**)**

**def** analyze**(***self***):**

'''MAIN FUNCTION: Does image analysis on the file and returns the lengths'''

# Some conditions to get it working right

**if not** *self***.**filepath **or** *self***.**cropping **or** *self***.**thresholding**: return None**

**if** *self***.**analyzed**:** *self***.**reset**(**full**=False)**

*self***.**overlay **=** *self***.**img**.**copy**()** # For later # Blur & Smoothen & Threshold

**if** *self***.**plant **==** "coleoptile"**:**

img2 **=** cv2**.**cvtColor**(***self***.**img**,** cv2**.**COLOR_BGR2GRAY**)** median **=** cv2**.**medianBlur**(**img2**,** 5**)**

blur **=** cv2**.**bilateralFilter**(**median**,**9**,**75**,**75**)**

_**,**mask **=** cv2**.**threshold**(**blur**,**0**,**255**,**cv2**.**THRESH_BINARY**+**cv2**.**THRESH_OTSU**)**

**elif** *self***.**plant **==** "seedling"**:**

img2 **=** cv2**.**cvtColor**(***self***.**img**,** cv2**.**COLOR_BGR2HSV**)** median **=** cv2**.**medianBlur**(**img2**,** 5**)**

blur **=** cv2**.**bilateralFilter**(**median**,**9**,**75**,**75**)**

lower_thres **=** np**.**array**([***self***.**hue_lower**,** *self***.**sat_lower**,** *self***.**val_lower**])** upper_thres **=** np**.**array**([***self***.**hue_upper**,** *self***.**sat_upper**,** *self***.**val_upper**])** mask **=** cv2**.**inRange**(**blur**,** lower_thres**,** upper_thres**)**

# Calibration: crop image to lower right or left corner

**if** *self***.**side **==** "right"**:**

cal **=** blur**[***self***.**img**.**shape**[**0**]-***self***.**img**.**shape**[**0**]//**5**:,**\

*self***.**img**.**shape**[**1**]-***self***.**img**.**shape**[**1**]//**5**:]**

**elif** *self***.**side **==** "left"**:**

cal **=** blur**[***self***.**img**.**shape**[**0**]-***self***.**img**.**shape**[**0**]//**5**:,**\

**:***self***.**img**.**shape**[**1**]//**5**]**

# Thresholding and stuff for scale bar

**if** *self***.**plant **==** "seedling"**:**

cal **=** cv2**.**split**(**cal**)[**2**]** # Get grayscale

_**,** calth **=** cv2**.**threshold**(**cal**,**0**,**255**,**cv2**.**THRESH_BINARY**+**cv2**.**THRESH_OTSU**)** calcont **=** cv2**.**findContours**(**calth**,**cv2**.**RETR_TREE**,**cv2**.**CHAIN_APPROX_SIMPLE**)[**1**]**

maxArea **=** 0

**for** contour **in** calcont**:**

# Scale bar is the biggest contour in that region -> get the width

**if** cv2**.**contourArea**(**contour**) >** maxArea**:** scale **=** cv2**.**boundingRect**(**contour**)[**2**]** maxArea **=** cv2**.**contourArea**(**contour**)**

# For displaying the results

*self***.**result **=** Text**(***self***,** height**=**10**,** width**=**50**,** wrap**=**WORD**)**

*self***.**firstline **=** "Calibration: {}mm={} pixels; 1 pixel={:.3f}mm\n\n"**.**format**(**\

*self***.**c_entry**,** scale**,** *self***.**c_entry**/**scale**)** *self***.**result**.**insert**(**END**,** *self***.**firstline**)**

# Get contours into a list

contours **=** cv2**.**findContours**(**mask**,**cv2**.**RETR_TREE**,**cv2**.**CHAIN_APPROX_SIMPLE**)[**1**]**

# Progress bar (shown in terminal)

progress **=** len**([**x **for** x **in** contours **if** cv2**.**contourArea**(**x**) >** *self***.**min_area**]) print(**"Progress: [{}]"**.**format**(**" "*****30**),** end**=**''**)**

**print(**"\b"*****31**,** end**=**''**)** stdout**.**flush**()**

# Initialization cc **=** 0 all_results **= []**

**for** contour **in** contours**:**

# Detects only large enough contours

**if** cv2**.**contourArea**(**contour**) <** *self***.**min_area**: continue**

cc **+=** 1

temp_results **= [**str**(**cc**)]**

# Draw just one contour

temp_array **=** np**.**zeros**(**mask**.**shape**,** dtype**=**np**.**uint8**)** cv2**.**fillPoly**(**temp_array**, [**contour**],** color**=(**255**,**255**,**255**))**

skeltest **=** skeletonize**(**temp_array**/**255**)** # Skeletonize graph **=** sknw**.**build_sknw**(**skeltest**)** # Build graph # Prune the graph only for seedlings

**if** *self***.**plant **==** "seedling"**:**

degree_list **= [**node **for** node**,**degree **in** graph**.**degree**().**items**()**\

**if** degree **==** 1**]** # List of terminal nodes

**while** degree_list**:**

node **=** degree_list**[**0**]**

neighbor **=** graph**.**neighbors**(**node**)[**0**]**

# If the edge is shorter than threshold, is not the lowest node, # and there isn't just two nodes left...

**if** graph**[**node**][**neighbor**][**'weight'**] <** 50 **and** \

node **!=** graph**.**nodes**()[-**1**] and** graph**.**degree**(**neighbor**) !=** 1**:** graph**.**remove_node**(**node**)**

# If neighbor becomes terminal node, add to the list

**if** graph**.**degree**(**neighbor**) ==** 1**:** degree_list**.**append**(**neighbor**)**

degree_list**.**pop**(**0**)**

# Pruning again - remove repeat edges (RARE)

**for** x**,** y **in** graph**.**edges**(): if** x **==** y**:**

graph**.**remove_edge**(**x**,** y**)**

# Some useful values

node**,** nodes **=** graph**.**node**,** graph**.**nodes**()**

branch_pts **= [**key **for** key**,**value **in** graph**.**degree**().**items**() if** value**==**3**]** branch_pts**.**sort**(**reverse**=True)**

# TOTAL SHOOT LENGTH

# For coleoptile, get the longest path possible

**if** *self***.**plant **==** "coleoptile"**:**

length **=** max**(**nx**.**single_source_dijkstra_path_length**(**graph**,**\ nodes**[-**1**]).**values**())**

branch_pts **= []**

# For seedling, get length from lowest to highest node

**elif** *self***.**plant **==** "seedling"**:**

length **=** nx**.**dijkstra_path_length**(**graph**,** nodes**[-**1**],** nodes**[**0**])** temp_results**.**append**(**length******self***.**c_entry**/**scale**)**

# LEAF LENGTHS AND INTERNODES

**for** i **in** range**(**len**(**branch_pts**)):** branch_pt **=** branch_pts**[**i**]**

# Select the two "higher" nodes from the neighbors n1**,** n2**,** _ **=** sorted**(**graph**.**neighbors**(**branch_pt**))**

# If it's not the last branch

**if** i **!=** len**(**branch_pts**)-**1**:**

# Internode length

interLength **=** nx**.**dijkstra_path_length**(\** graph**,** branch_pt**,** branch_pts**[**i**+**1**])**

# Determine leaf node - it's the one NOT in the longest path

**if** n1 **in** nx**.**dijkstra_path**(**graph**,** nodes**[-**1**],** nodes**[**0**]):** leaf_node **=** *self***.**findTerminalNode**(**graph**,** branch_pt**,** n2**)**

**else:**

leaf_node **=** *self***.**findTerminalNode**(**graph**,** branch_pt**,** n1**)**

# Append results without correction

**if** i **!=** 0**:**

temp_results**.**append**(**interLength******self***.**c_entry**/**scale**)**

# Terminal branching point

**else:**

# Approximate orientation by using points below the branching point # Main one: if there is more than one leaf

**if** len**(**branch_pts**) !=** 1**:**

# Get all the internode points

inter **=** *self***.**mergePoints**(**graph**,** branch_pt**,** branch_pts**[**i**-**1**])**

# If the internode is really short, use the first 100 points

**if** len**(**inter**) <** 150**:** inter **=** inter**[:**100**]**

# This one uses points a bit further down -> more accurate

**else:**

inter **=** inter**[**49**:**149**]**

interApprox1 **=** inter**[**0**][::-**1**]** interApprox2 **=** inter**[-**1**][::-**1**]**

# Single-leaf case, use points above the branching point

**else:**

path **=** nx**.**dijkstra_path**(**graph**,** nodes**[-**1**],** nodes**[**0**])**

interApprox1 **=** node**[**branch_pt**][**'o'**][::-**1**]**

interApprox2 **=** graph**[**branch_pt**][**path**[**path**.**index**(**branch_pt**)+**1**]]**\

**[**'pts'**][-**100**:][**0**][::-**1**]**

# Determine long and short node -> compare until same y-coordinate l_node**,** l_pts**,** s_node**,** s_pts **=** *self***.**compareNodes**(\**

n1**,** n2**,** graph**,** branch_pt**)**

# Starting point for longer node

**try:**

start_pt **=** list**(**l_pts**[:,**0**]).**index**(**round**(**node**[**s_node**][**'o'**][**0**]))**

**except** ValueError**:** start_pt **=** 0

# Get the maximum distance from all points on node-branch point # node-branch point edge to the internode line

s_dist **=** max**([***self***.**distance**(**interApprox1**,** interApprox2**,**\ each_pt**[::-**1**]) for** each_pt **in** s_pts**])**

l_dist **=** max**([***self***.**distance**(**interApprox1**,** interApprox2**,**\ each_pt**[::-**1**]) for** each_pt **in** l_pts**[**start_pt**:]])**

# The one with the larger maximum distance is the leaf

**if** l_dist **>** s_dist**:** leaf_node **=** l_node

**else:**

leaf_node **=** s_node

leafLength **=** nx**.**dijkstra_path_length**(**graph**,** branch_pt**,** leaf_node**)** # Draw leaf

leaf_pts **=** *self***.**mergePoints**(**graph**,** branch_pt**,** leaf_node**)** leaf_pts **=** np**.**concatenate**((**leaf_pts**[:,[**1**]],** leaf_pts**[:,[**0**]]),** 1**)** cv2**.**polylines**(***self***.**overlay**,** np**.**int32**([**leaf_pts**]),**\

**False, (**0**,**255**,**255**),** 5**)**

# For the first branching point, correct the distance using the # distance from the lowest node to the first branch point

**if** i **==** 0**:**

correction **=** nx**.**dijkstra_path_length**(**graph**,** nodes**[-**1**],** branch_pt**)**

# More than one branching point, there is internode

**if** len**(**branch_pts**) !=** 1**:**

# Fixed weight for correction interLength **+=** 0.8*****correction

temp_results**.**append**(**interLength******self***.**c_entry**/**scale**)**

# Also for leaf, same weight leafLength **+=** 0.8*****correction

temp_results**.**append**(**leafLength******self***.**c_entry**/**scale**)** # Drawing bounding box and put text

x**,**y**,**w**,**h **=** cv2**.**boundingRect**(**contour**)** cv2**.**rectangle**(***self***.**overlay**,(**x**,**y**),(**x**+**w**,**y**+**h**),(**0**,**0**,**255**),**5**)** cv2**.**putText**(***self***.**overlay**,**str**(**cc**),(**x**-**15**,**y**-**15**),**cv2**.**FONT_HERSHEY_SIMPLEX**,**\

4**, (**0**,**0**,**255**),**5**)**

# Results order: total length, internode 1, leaf 1, leaf 2 all_results**.**append**(**temp_results**)**

# Progress bar (look at terminal)

**print(**'#'***(**int**(**cc**/**progress*****30**)-**int**((**cc**-**1**)/**progress*****30**)),** end**=**''**)** stdout**.**flush**()**

**print()**

*self***.**results **= {**1**:{**'val'**:**all_results**}}** # For post-processing

*self***.**calcStatistics**()** *self***.**showText**()**

# Display new image with bounding box

temp = cv2**.**cvtColor**(***self***.**overlay**,** cv2**.**COLOR_BGR2RGB**)** temp **=** Image**.**fromarray**(**temp**)**

*self***.**showImg**(**temp**)**

# Scrollbar

*self***.**scroll **=** Scrollbar**(***self***)** *self***.**result**.**config**(**yscrollcommand**=***self***.**scroll**.**set**)** *self***.**scroll**.**config**(**command**=***self***.**result**.**yview**)**

# Entrybox

*self***.**e3 **=** Entry**(***self***)**

# Separator

*self***.**divider **=** ttk**.**Separator**(***self***,** orient**=**"vertical"**)**

# Buttons

*self***.**exportfile_b **=** Button**(***self***,** text**=**'Export File'**,** command**=***self***.**exportFile**)** *self***.**exportimg_b **=** Button**(***self***,** text**=**'Export Image'**,** command**=***self***.**exportImg**)** *self***.**reset_button **=** Button**(***self***,** text**=**'Reset'**,** command**=\**

**lambda:***self***.**reset**(**full**=True))**

*self***.**merge_button **=** Button**(***self***,** text**=**'Merge'**,** command**=***self***.**mergeRegions**)** *self***.**remove_button **=** Button**(***self***,** text**=**'Remove'**,** command**=***self***.**removeRegions**)** *self***.**select_button **=** Button**(***self***,** text**=**'Select'**,** command**=***self***.**selectRegions**)** *self***.**group_button **=** Button**(***self***,** text**=**'Group'**,** command**=***self***.**groupRegions**)** *self***.**apply_button **=** Button**(***self***,** text**=**'Apply'**,** command**=***self***.**applyGroups**)**

# Layout

*self***.**result**.**grid**(**row**=**2**,** column**=**2**,** padx**=(**10**,**20**),** pady**=**10**,** sticky**=(**W**+**E**,** N**+**S**))**

*self***.**scroll**.**grid**(**row**=**2**,** column**=**2**,** padx**=(**10**,**20**),** pady**=**10**,** sticky**=(**N**+**S**,**E**))**

*self***.**e3**.**grid**(**row**=**3**,** column**=**2**,** padx**=**10**,** pady**=(**5**,**3**),** sticky**=**W**)** *self***.**merge_button**.**grid**(**row**=**3**,** column**=**2**,** padx**=(**10**,**245**),** pady**=(**5**,**3**),** sticky**=**E**)** *self***.**select_button**.**grid**(**row**=**3**,** column**=**2**,** padx**=(**10**,**194**),** pady**=(**5**,**3**),** sticky**=**E**)** *self***.**remove_button**.**grid**(**row**=**3**,** column**=**2**,** padx**=(**10**,**131**),** pady**=(**5**,**3**),** sticky**=**E**)**

*self***.**divider**.**grid**(**row**=**3**,** column**=**2**,** padx**=(**10**,**121**),** pady**=(**5**,**3**),**sticky**=(**N**+**S**,** E**))**

*self***.**group_button**.**grid**(**row**=**3**,** column**=**2**,** padx**=(**10**,**70**),** pady**=(**5**,**3**),** sticky**=**E**)**

*self***.**apply_button**.**grid**(**row**=**3**,** column**=**2**,** padx**=(**10**,**20**),** pady**=(**5**,**3**),** sticky**=**E**)**

*self***.**exportfile_b**.**grid**(**row**=**4**,** column**=**2**,** padx**=(**10**,**160**),** pady**=(**10**,**3**),** sticky**=**E**)** *self***.**exportimg_b**.**grid**(**row**=**4**,** column**=**2**,** padx**=(**10**,**70**),** pady**=(**10**,**3**),** sticky**=**E**)** *self***.**reset_button**.**grid**(**row**=**4**,** column**=**2**,** padx**=(**10**,**20**),** pady**=(**10**,**3**),** sticky**=**E**)**

# Checkpoint

*self***.**analyzed **= True**

**def** distance**(***self***,** P1**,** P2**,** P3**):**

'''Returns the distance between point P3 and line containing points P1, P2'''

**return** np**.**linalg**.**norm**(**np**.**cross**(**np**.**subtract**(**P2**,** P1**),** np**.**subtract**(**P1**,**P3**))/**\ np**.**linalg**.**norm**(**np**.**subtract**(**P2**,**P1**)))**

**def** findTerminalNode**(***self***,** graph**,** branch_pt**,** node**):** '''Trace the path until the terminal node of a leaf'''

# Initialization all_neighbors **= {**branch_pt**}** x **=** node

neighbors_set **=** set**(**graph**.**neighbors**(**x**))**

# set becomes empty at the terminal node

**while** neighbors_set**:** all_neighbors**.**add**(**x**)**

# Get neighbors of x and remove duplicates with main set neighbors_set **=** set**(**graph**.**neighbors**(**x**))**

neighbors_set **-= (**all_neighbors**&**neighbors_set**)**

# If x has new neighbors, update x as the neighbor

**if** neighbors_set**:**

x **=** sorted**(**list**(**neighbors_set**))[-**1**] return** x

**def** mergePoints**(***self***,** graph**,** n1**,** n2**):**

'''Returns all the points from point n1 to n2'''

# Straightforward path

**if** len**(**nx**.**dijkstra_path**(**graph**,** n1**,** n2**)) ==** 2**:** pts **=** graph**[**n1**][**n2**][**'pts'**]**

# Some obstacles...merge all the edges connecting them

**else:**

path **=** sorted**(**nx**.**dijkstra_path**(**graph**,** n1**,** n2**))**

pts **=** np**.**concatenate**(**tuple**(**graph**[**path**[**j**]][**path**[**j**+**1**]][**'pts'**]**\

**for** j **in** range**(**len**(**path**)-**1**)))**

**return** pts

**def** compareNodes**(***self***,** n1**,** n2**,** graph**,** branch_pt**):** '''Takes two nodes and the graph as argument, returns

(lower node, points, higher node, points), with points being

the x,y coordinates from the branch point to the terminal node'''

# Get the end node and all the points in between t1 **=** *self***.**findTerminalNode**(**graph**,** branch_pt**,** n1**)** p1 **=** *self***.**mergePoints**(**graph**,** branch_pt**,** t1**)**

# Do the same for n2

t2 **=** *self***.**findTerminalNode**(**graph**,** branch_pt**,** n2**)** p2 **=** *self***.**mergePoints**(**graph**,** branch_pt**,** t2**)**

# Compare the two end nodes -> shorter node has greater y

**if** graph**.**node**[**t1**][**'o'**][**0**] >** graph**.**node**[**t2**][**'o'**][**0**]: return (**t2**,** p2**,** t1**,** p1**)**

**else:**

**return (**t1**,** p1**,** t2**,** p2**)**

**def** calcStatistics**(***self***):**

'''Calculate mean, standard deviation, standard error for each group'''

*self***.**checkGroups**()** # Remove empty groups and rearranges

**for** group_no **in** *self***.**results**:**

*self***.**results**[**group_no**] = {**'val'**:***self***.**results**[**group_no**][**'val'**]}** # Reset group **=** *self***.**results**[**group_no**]**

max_len **=** max**([**len**(**plant**) for** plant **in** group**[**'val'**]]) for** i **in** range**(**1**,** max_len**):**

# First one is always the total length

**if** i **==** 1**:**

intresult **= [**plant**[**1**] for** plant **in** group**[**'val'**]]** group**[**'lengthmean'**] =** np**.**mean**(**intresult**)** group**[**'lengthsd'**] =** np**.**std**(**intresult**,** ddof**=**1**)** group**[**'lengthse'**] =** stats**.**sem**(**intresult**)**

# Last one is always the leaf

**elif** i **==** max_len**-**1**:**

# even and odd indices have different guidelines...

**if** i**%**2 **==** 0**:**

intresult **= [**plant**[-**1**] for** plant **in** group**[**'val'**]**\

**if** len**(**plant**) >** i**]**

**elif** i**%**2 **==** 1**:**

intresult **= [**plant**[-**1**] for** plant **in** group**[**'val'**]**\

**if** len**(**plant**) >=** i**]**

group**[**'leaf{}mean'**.**format**(**i**//**2**)] =** np**.**mean**(**intresult**)** group**[**'leaf{}sd'**.**format**(**i**//**2**)] =** np**.**std**(**intresult**,** ddof**=**1**)** group**[**'leaf{}se'**.**format**(**i**//**2**)] =** stats**.**sem**(**intresult**)**

# In-between can be leaf or internode, can get kind of complicated

**else:**

key **= [**'internode'**,** 'leaf'**]** # if i is even -> internode **if** i**%**2 **==** 0**:**

intresult **= [**plant**[**i**] for** plant **in** group**[**'val'**]**\

**if** len**(**plant**) >** i**+**1**]**

# if i is odd -> leaf

**elif** i**%**2 **==** 1**:**

intresult **= [**plant**[**i**] for** plant **in** group**[**'val'**]**\

**if** len**(**plant**) >** i**]**

intresult **+= [**plant**[-**1**] for** plant **in** group**[**'val'**]**\

**if** len**(**plant**) ==** i**]**

group**[**'{}{}mean'**.**format**(**key**[**i**%**2**],** i**//**2**)] =** np**.**mean**(**intresult**)** group**[**'{}{}sd'**.**format**(**key**[**i**%**2**],** i**//**2**)] =** np**.**std**(**intresult**,** ddof**=**1**)** group**[**'{}{}se'**.**format**(**key**[**i**%**2**],** i**//**2**)] =** stats**.**sem**(**intresult**)**

**def** showText**(***self***):**

'''Displays the results in a nice fashion'''

*self***.**result**.**config**(**state**=**"normal"**)** *self***.**result**.**delete**(**2.0**,** END**)**

**for** group_no **in** sorted**(***self***.**results**.**keys**()):** group **=** *self***.**results**[**group_no**]**

# If there is only one group, don't print group number

**if** len**(***self***.**results**) !=** 1**:**

*self***.**result**.**insert**(**END**,** '\n#Group {}\n'**.**format**(**group_no**)) else:**

*self***.**result**.**insert**(**END**,** '\n'**)**

# Print each result except the mean, SD, and stderr key **= [**'Internode'**,** 'Leaf'**]**

**for** plant **in** group**[**'val'**]:**

**for** j **in** range**(**1**,** len**(**plant**)):**

# First value is plant no., second value is the total length

**if** j **==** 1**:**

*self***.**result**.**insert**(**END**,**\

"#{}, Length: {:.2f};\n"**.**format**(**plant**[**0**],** plant**[**1**]))**

# Final value is always the leaf length

**elif** j **==** len**(**plant**)-**1**:**

*self***.**result**.**insert**(**END**,**\

" Leaf {}: {:.2f}\n"**.**format**(**j**//**2**,** plant**[**j**]))**

# The in-between ones switch between leaf and internode

**else:**

*self***.**result**.**insert**(**END**,**" {} {}: {:.2f};\n"**.**format**(**\

key**[**j**%**2**],** int**(**j**/**2**),** plant**[**j**]))**

# Separator for the Mean, SD, and SE values

*self***.**result**.**insert**(**END**,** "="*****30 **+** "\n"**)**

# For each parameter, there exists Mean, SD, and SE # Divide by 3 to get # of parameters

key **= [**'internode'**,** 'leaf'**]**

**for** q **in** range**(**1**, ((**len**(**group**)-**1**)//**3**)+**1**):**

# First one is the total length

**if** q **==** 1**:**

*self***.**result**.**insert**(**END**,** "Length: Mean, {:.2f}\n"**.**format**(**\

group**[**'lengthmean'**]))**

*self***.**result**.**insert**(**END**,** " SD, {:.2f}\n"**.**format**(**\

group**[**'lengthsd'**]))**

*self***.**result**.**insert**(**END**,** " SE, {:.2f}\n"**.**format**(**\

group**[**'lengthse'**]))**

# Last one is the leaf

**elif** q **== (**len**(**group**)-**1**)//**3**:**

*self***.**result**.**insert**(**END**,** "Leaf {}: Mean, {:.2f}\n"**.**format**(**\

q**//**2**,** group**[**'leaf{}mean'**.**format**(**q**//**2**)]))**

*self***.**result**.**insert**(**END**,** " SD, {:.2f}\n"**.**format**(**\

group**[**'leaf{}sd'**.**format**(**q**//**2**)]))**

*self***.**result**.**insert**(**END**,** " SE, {:.2f}\n"**.**format**(**\

group**[**'leaf{}se'**.**format**(**q**//**2**)]))**

# In-between can be either leaf or internode

**else:**

*self***.**result**.**insert**(**END**,** "{} {}: Mean, {:.2f}\n"**.**format**(**\

key**[**q**%**2**].**capitalize**(),** q**//**2**,**\ group**[**'{}{}mean'**.**format**(**key**[**q**%**2**],** q**//**2**)]))**

*self***.**result**.**insert**(**END**,** " SD, {:.2f}\n"**.**format**(**\

group**[**'{}{}sd'**.**format**(**key**[**q**%**2**],** q**//**2**)]))**

*self***.**result**.**insert**(**END**,** " SE, {:.2f}\n"**.**format**(**\

group**[**'{}{}se'**.**format**(**key**[**q**%**2**],** q**//**2**)]))**

*self***.**result**.**insert**(**END**,** "="*****30**)**

*self***.**result**.**config**(**state**=**"disabled"**) def** checkGroups**(***self***):**

'''Removes empty groups and rearranges the groups so they are in chronological order'''

# Remove empty groups

keys **=** sorted**(***self***.**results**.**keys**())**

**for** group_no **in** keys**:**

**if** *self***.**results**[**group_no**][**'val'**] == []:**

*self***.**results**.**pop**(**group_no**)**

# Initialize again

keys **=** sorted**(***self***.**results**.**keys**())** len_keys **=** len**(**keys**)**

# If something was removed

**if** len**(***self***.**results**) !=** keys**[-**1**]: for** i **in** range**(**1**,** len_keys**):**

# If the keys are not in order, rearrange them

**if** keys**[**i**] -** keys**[**i**-**1**] !=** 1**:**

*self***.**results**[**i**+**1**] =** *self***.**results**.**pop**(**keys**[**i**])**

keys **=** sorted**(***self***.**results**.**keys**())**

**def** mergeRegions**(***self***):**

'''Merges two or more regions together'''

# Get text and clear entry box merge_text **=** *self***.**e3**.**get**()** *self***.**e3**.**delete**(**0**,** END**)**

**if not** merge_text **or** *self***.**grouping**: return None**

merge_list **=** merge_text**.**split**(**','**)** # For each merging

**for** element **in** merge_list**:**

# Some initialization merge_sum **=** 0 pos_list**=[]**

temp_merge **=** element**.**split**(**'+'**)**

# Check within each group (no cross-group merging!)

**for** group_no **in** *self***.**results**.**keys**():**

# For each number you have to merge

**for** element2 **in** temp_merge**:**

# Add the leaf length or total length (default = total) code **=** 't'

**if** 'l' **in** element2**:**

code **=** element2**[-**2**:]** element2 **=** element2**[:-**2**]**

# Find the plant to be merged

**for** pos**,** value **in** enumerate**(***self***.**results**[**group_no**][**'val'**]): if** value**[**0**] ==** element2**:**

# If it's the leaf, indexing is a little complicated

**if** code**[**0**] ==** 'l'**:**

**if** len**(**value**) == (**int**(**code**[**1**])***2**)+**1**:**

i **=** int**(**code**[**1**])***2

**else:**

i **= (**int**(**code**[**1**])***2**)+**1

# Total length is the second value of each plant

**else:**

i **=** 1

# Add up and store positions + plant part pos_list**.**append**((**pos**,** i**))**

merge_sum **+=** value**[**i**]**

# If the no. of values found is equal to no. that has to be merged

**if** len**(**pos_list**) ==** len**(**temp_merge**):**

# First number in the input gets updated

*self***.**results**[**group_no**][**'val'**][**pos_list**[**0**][**0**]][**0**] =**\ element

*self***.**results**[**group_no**][**'val'**][**pos_list**[**0**][**0**]][**pos_list**[**0**][**1**]] =**\ merge_sum

# The rest is removed pos_list**.**pop**(**0**)**

**for** i **in** pos_list**[::-**1**]:**

*self***.**results**[**group_no**][**'val'**].**pop**(**i**[**0**])**

# Reset everything pos_list**=[]** merge_sum **=** 0

# Update results *self***.**calcStatistics**()** *self***.**showText**()**

**def** selectRegions**(***self***):**

'''Select only certain regions and removes the rest'''

select_text **=** *self***.**e3**.**get**()**

*self***.**e3**.**delete**(**0**,** END**)** # Clear entry box

**if not** select_text **or** *self***.**grouping**: return None**

# Initialization

select_list **=** select_text**.**split**(**','**)** pos_list**,** select_list_new **= [],[]**

# This is if the user enters a range, i.e., 4-9

**for** element **in** select_list**:** temp **=** element**.**split**(**'-'**) if** len**(**temp**) ==** 2**:**

temp **= [**str**(**x**) for** x **in** range**(**int**(**temp**[**0**]),** int**(**temp**[**1**])+**1**)]** select_list_new **+=** temp

# Get the positions and group numbers of selected regions

**for** element **in** select_list_new**:**

**for** group_no **in** *self***.**results**.**keys**():**

**for** pos**,**value **in** enumerate**(***self***.**results**[**group_no**][**'val'**]): if** element **==** value**[**0**]:**

pos_list**.**append**((**group_no**,** pos**))**

# Get the values in those positions pos_list**.**sort**()**

new_results **= {**i**:{**'val'**:[]} for** i **in** *self***.**results**.**keys**()} for** group_no**,** pos **in** pos_list**:**

new_results**[**group_no**][**'val'**].**append**(***self***.**results**[**group_no**][**'val'**][**pos**])**

# Update results *self***.**results **=** new_results *self***.**calcStatistics**()** *self***.**showText**()**

**def** removeRegions**(***self***):**

'''Removes one or more region'''

# Get text from entry box and remove values remove_text **=** *self***.**e3**.**get**()** *self***.**e3**.**delete**(**0**,** END**)**

**if not** remove_text **or** *self***.**grouping**: return None**

# Initialization

remove_list **=** remove_text**.**split**(**','**)** pos_list**,** remove_list_new **= [],[]**

# This is if the user enters a range, i.e., 4-9

**for** element **in** remove_list**:** temp **=** element**.**split**(**'-'**) if** len**(**temp**) ==** 2**:**

temp **= [**str**(**x**) for** x **in** range**(**int**(**temp**[**0**]),** int**(**temp**[**1**])+**1**)]** remove_list_new **+=** temp

# Get the positions and group numbers of regions to be removed

**for** element **in** remove_list_new**:**

**for** group_no **in** *self***.**results**.**keys**():**

**for** pos**,**value **in** enumerate**(***self***.**results**[**group_no**][**'val'**]): if** element **==** value**[**0**]:**

pos_list**.**append**((**group_no**,** pos**))**

# Remove the regions from back to front pos_list**.**sort**(**reverse**=True)**

**for** group_no**,** pos **in** pos_list**:**

*self***.**results**[**group_no**][**'val'**].**pop**(**pos**)**

# Update results *self***.**calcStatistics**()** *self***.**showText**()**

**def** groupRegions**(***self***):**

'''Allows the user to group plant samples together'''

*self***.**grouping **= True**

*self***.**result**.**config**(**state**=**'normal'**)**

# Replaces the calibration text with instructions

*self***.**result**.**delete**(**1.0**,**2.0**)**

*self***.**result**.**insert**(**1.0**,** "Please enter asterisks between groups:\n"**)**

# Get text from second line onwards and convert to list all_text **=** *self***.**result**.**get**(**2.0**,** "end-1c"**)**

all_text **=** all_text**.**split**(**'\n'**)**

# Remove mean, standard deviation, and standard error lines # Get position of the lines

pos_list **= []**

**for** line_no**,** line **in** enumerate**(**all_text**): if** line**.**startswith**(**'='**):**

pos_list**.**append**(**line_no**)**

# Remove them pos_list**.**sort**(**reverse**=True)**

**for** pos **in** range**(**0**,**len**(**pos_list**),**2**):**

*self***.**result**.**delete**(**"{}.0+2l"**.**format**(**pos_list**[**pos**+**1**]),**\

"{}.0+3l"**.**format**(**pos_list**[**pos**]))**

**def** applyGroups**(***self***):** '''Apply the groups'''

# Get second line onwards and convert to list all_text **=** *self***.**result**.**get**(**2.0**,** "end-1c"**)** all_text **=** all_text**.**split**(**'#'**)**

# Initialization *self***.**results **= {}** all_results**,** temp **= [], []** group_no **=** 1

# First one will be empty from the splitting

**for** element **in** all_text**[**1**:]:**

# Keep existing groups

**if** element**.**startswith**(**"G"**):**

# Except group 1, don't create a new group yet

**if** element**.**split**()[**1**] !=** "1"**:** *self***.**results**[**group_no**] = {**'val'**:**all_results**}** group_no **+=** 1

all_results **= [] continue**

index**,** values **=** element**.**split**(**','**)**

# Found a new group

**if** values**.**strip**().**endswith**(**'*'**):**

values **=** values**.**strip**().**rstrip**(**'*'**)** temp **= [**index**]**

values **= [**val**.**strip**() for** val **in** values**.**split**(**';'**)]** temp **+= [**float**(**length**.**split**(**':'**)[**1**].**strip**())**\

**for** length **in** values **if** length **!=** ''**]** all_results**.**append**(**temp**)**

# Create new group

*self***.**results**[**group_no**] = {**'val'**:**all_results**}**

group_no **+=** 1 all_results **= []**

# Keep on collecting values until new group is found

**else:**

temp **= [**index**]**

values **= [**val**.**strip**() for** val **in** values**.**split**(**';'**)]** temp **+= [**float**(**length**.**split**(**':'**)[**1**].**strip**())**\

**for** length **in** values **if** length **!=** ''**]** all_results**.**append**(**temp**)**

# Final group

*self***.**results**[**group_no**] = {**'val'**:**all_results**}**

# Update results *self***.**calcStatistics**()** *self***.**result**.**delete**(**1.0**,**2.0**)**

*self***.**result**.**insert**(**1.0**,***self***.**firstline**)** *self***.**showText**()**

*self***.**grouping **= False def** cropImgStart**(***self***):**

''' Extends the crop option'''

**if not** *self***.**filepath **or** *self***.**cropping **or** *self***.**analyzed **or** *self***.**thresholding**: return None**

*self***.**cropping **= True**

# Create sliders and set initial values

*self***.**cropleft **=** Scale**(***self***,**from_**=**0**,**length**=**275**,**to**=***self***.**width**,**orient**=**HORIZONTAL**)** *self***.**cropright **=** Scale**(***self***,**from_**=**0**,**length**=**275**,**to**=***self***.**width**,**orient**=**HORIZONTAL**)** *self***.**cropright**.**set**(***self***.**width**)**

*self***.**croptop **=** Scale**(***self***,**from_**=**0**,**length**=**275**,**to**=***self***.**height**,**orient**=**HORIZONTAL**)** *self***.**cropdown **=** Scale**(***self***,**from_**=**0**,**length**=**275**,**to**=***self***.**height**,**orient**=**HORIZONTAL**)** *self***.**cropdown**.**set**(***self***.**height**)**

# Labels

*self***.**toplabel **=** Label**(***self***,** text**=**'Top'**)** *self***.**downlabel **=** Label**(***self***,** text**=**'Bottom'**)** *self***.**leftlabel **=** Label**(***self***,** text**=**'Left'**)** *self***.**rightlabel **=** Label**(***self***,** text**=**'Right'**)**

# Buttons

*self***.**check **=** Button**(***self***,** text**=**"Check"**,** command**=***self***.**cropImgCheck**)**

*self***.**confirm **=** Button**(***self***,** text**=**"Confirm"**,** command**=***self***.**cropImgEnd**)**

# Layout

*self***.**toplabel**.**grid**(**row**=**2**,** column**=**2**,** sticky**=**NE**,** padx**=**10**,** pady**=(**28**,**0**))**

*self***.**downlabel**.**grid**(**row**=**2**,** column**=**2**,** sticky**=**NE**,** padx**=**10**,** pady**=(**73**,**0**))**

*self***.**leftlabel**.**grid**(**row**=**2**,** column**=**2**,** sticky**=**NE**,** padx**=**10**,** pady**=(**118**,**0**))** *self***.**rightlabel**.**grid**(**row**=**2**,** column**=**2**,** sticky**=**NE**,** padx**=**10**,** pady**=(**163**,**0**))** *self***.**croptop**.**grid**(**row**=**2**,** column**=**3**,** sticky**=(**W**+**E**,**NW**),** padx**=**0**,** pady**=(**10**,**0**))** *self***.**cropdown**.**grid**(**row**=**2**,** column**=**3**,** sticky**=(**W**+**E**,**NW**),** padx**=**0**,** pady**=(**55**,**0**))** *self***.**cropleft**.**grid**(**row**=**2**,** column**=**3**,** sticky**=(**W**+**E**,**NW**),** padx**=**0**,** pady**=(**100**,**0**))** *self***.**cropright**.**grid**(**row**=**2**,** column**=**3**,** sticky**=(**W**+**E**,**NW**),** padx**=**0**,** pady**=(**145**,**0**))** *self***.**check**.**grid**(**row**=**2**,** column**=**3**,** sticky**=**NW**,** padx**=(**3**,**0**),** pady**=(**200**,**0**))** *self***.**confirm**.**grid**(**row**=**2**,** column**=**3**,** sticky**=**NW**,** padx**=(**60**,**10**),** pady**=(**200**,**0**))**

**def** cropImgCheck**(***self***):**

'''Checks region being cropped''' tempImg **=** *self***.**img**.**copy**()**

# Get values from the sliders top **=** *self***.**croptop**.**get**()** bottom **=** *self***.**cropdown**.**get**()** left **=** *self***.**cropleft**.**get**()** right **=** *self***.**cropright**.**get**()**

# Add black rectangle over the picture

cv2**.**rectangle**(**tempImg**, (**0**,**0**), (***self***.**width**,***self***.**height**), (**0**,** 0**,** 0**), -**1**)**

# Add white rectangle over selected area

cv2**.**rectangle**(**tempImg**, (**left**,**bottom**), (**right**,** top**), (**255**,** 255**,** 255**), -**1**)**

# Overlay the rectangles with original image opacity **=** 0.5

tempImg **=** cv2**.**addWeighted**(**tempImg**,** opacity**,** *self***.**img**,** 1**-**opacity**,** 0**)**

# Show the image

tempImg **=** cv2.cvtColor(tempImg, cv2.COLOR_BGR2RGB) tempImg **=** Image**.**fromarray**(**tempImg**)** *self***.**showImg**(**tempImg**)**

**def** cropImgEnd**(***self***):** '''Crops the image'''

# Get values from slider top **=** *self***.**croptop**.**get**()**

bottom **=** *self***.**cropdown**.**get**()** left **=** *self***.**cropleft**.**get**()** right **=** *self***.**cropright**.**get**()**

# Remove everything! *self***.**toplabel**.**grid_remove**()** *self***.**downlabel**.**grid_remove**()** *self***.**leftlabel**.**grid_remove**()** *self***.**rightlabel**.**grid_remove**()** *self***.**croptop**.**grid_remove**()** *self***.**cropdown**.**grid_remove**()** *self***.**cropleft**.**grid_remove**()** *self***.**cropright**.**grid_remove**()** *self***.**check**.**grid_remove**()** *self***.**confirm**.**grid_remove**()**

# Actual cropping

*self***.**img **=** *self***.**img**[**top**:**bottom**,** left**:**right**]**

# Update information

*self***.**height**,** *self***.**width**,** _ **=** *self***.**img**.**shape

*self***.**imagesize**.**set**(**"Image size: {} x {}"**.**format**(***self***.**width**,** *self***.**height**))**

# Show new image

temp **=** cv2.cvtColor(*self***.**img, cv2.COLOR_BGR2RGB) temp **=** Image**.**fromarray**(**temp**)**

*self***.**showImg**(**temp**)**

# Now analysis can begin!

*self***.**cropping **= False**

**def** thresholdStart**(***self***):**

'''Extends the threshold option'''

**if not** *self***.**filepath **or** *self***.**cropping **or** *self***.**analyzed **or**\

*self***.**thresholding **or** *self***.**plant **==** 'coleoptile'**: return None**

*self***.**thresholding **= True**

# Create sliders and set initial values

*self***.**HL**=**Scale**(***self***,** from_**=**0**,** length**=**255**,** to**=**255**,** orient**=**HORIZONTAL**)** *self***.**HR **=**Scale**(***self***,** from_**=**0**,** length**=**255**,** to**=**255**,** orient**=**HORIZONTAL**)** *self***.**HL**.**set**(***self***.**hue_lower**)**

*self***.**HR**.**set**(***self***.**hue_upper**)**

*self***.**SL **=**Scale**(***self***,** from_**=**0**,** length**=**255**,** to**=**255**,** orient**=**HORIZONTAL**)** *self***.**SR **=**Scale**(***self***,** from_**=**0**,** length**=**255**,** to**=**255**,** orient**=**HORIZONTAL**)** *self***.**SL**.**set**(***self***.**sat_lower**)**

*self***.**SR**.**set**(***self***.**sat_upper**)**

*self***.**VL **=**Scale**(***self***,** from_**=**0**,** length**=**255**,** to**=**255**,** orient**=**HORIZONTAL**)** *self***.**VR **=**Scale**(***self***,** from_**=**0**,** length**=**255**,** to**=**255**,** orient**=**HORIZONTAL**)** *self***.**VL**.**set**(***self***.**val_lower**)**

*self***.**VR**.**set**(***self***.**val_upper**)**

# Labels

*self***.**hueLabel **=** Label**(***self***,** text**=**'Hue'**)** *self***.**satLabel **=** Label**(***self***,** text**=**'Sat'**)** *self***.**valLabel **=** Label**(***self***,** text**=**'Value'**)**

# Buttons

*self***.**check **=** Button**(***self***,** text**=**"Check"**,** command**=***self***.**thresholdCheck**)** *self***.**default **=** Button**(***self***,** text**=**"Reset"**,** command**=***self***.**thresholdDefault**)** *self***.**confirm **=** Button**(***self***,** text**=**"Confirm"**,** command**=***self***.**thresholdEnd**)**

# Layout

*self***.**hueLabel**.**grid**(**row**=**2**,** column**=**2**,** sticky**=**NE**,** padx**=**10**,** pady**=(**28**,**0**))** *self***.**satLabel**.**grid**(**row**=**2**,** column**=**2**,** sticky**=**NE**,** padx**=**10**,** pady**=(**118**,**0**))** *self***.**valLabel**.**grid**(**row**=**2**,** column**=**2**,** sticky**=**NE**,** padx**=**10**,** pady**=(**208**,**0**))**

*self***.**HL**.**grid**(**row**=**2**,** column**=**3**,** sticky**=(**W**+**E**,**NW**),** padx**=**0**,** pady**=(**10**,**0**))** *self***.**HR**.**grid**(**row**=**2**,** column**=**3**,** sticky**=(**W**+**E**,**NW**),** padx**=**0**,** pady**=(**55**,**0**))** *self***.**SL**.**grid**(**row**=**2**,** column**=**3**,** sticky**=(**W**+**E**,**NW**),** padx**=**0**,** pady**=(**100**,**0**))** *self***.**SR**.**grid**(**row**=**2**,** column**=**3**,** sticky**=(**W**+**E**,**NW**),** padx**=**0**,** pady**=(**145**,**0**))** *self***.**VL**.**grid**(**row**=**2**,** column**=**3**,** sticky**=(**W**+**E**,**NW**),** padx**=**0**,** pady**=(**190**,**0**))** *self***.**VR**.**grid**(**row**=**2**,** column**=**3**,** sticky**=(**W**+**E**,**NW**),** padx**=**0**,** pady**=(**235**,**0**))**

*self***.**check**.**grid**(**row**=**2**,** column**=**3**,** sticky**=**NW**,** padx**=(**3**,**0**),** pady**=(**290**,**0**))** *self***.**default**.**grid**(**row**=**2**,** column**=**3**,** sticky**=**NW**,** padx**=(**57**,**10**),** pady**=(**290**,**0**))** *self***.**confirm**.**grid**(**row**=**2**,** column**=**3**,** sticky**=**NW**,** padx**=(**107**,**10**),** pady**=(**290**,**0**))**

**def** thresholdCheck**(***self***):**

'''Checks the thresholding with given values'''

tempImg **=** *self***.**img**.**copy**()**

tempImg2 **=** cv2**.**cvtColor**(**tempImg**,** cv2**.**COLOR_BGR2HSV**)** median **=** cv2**.**medianBlur**(**tempImg2**,** 5**)**

blur **=** cv2**.**bilateralFilter**(**median**,**9**,**75**,**75**)**

# Get values from the sliders

hue_lower**,** hue_upper **=** *self***.**HL**.**get**(),** *self***.**HR**.**get**()** sat_lower**,** sat_upper **=** *self***.**SL**.**get**(),** *self***.**SR**.**get**()** val_lower**,** val_upper **=** *self***.**VL**.**get**(),** *self***.**VR**.**get**()**

lower_thres **=** np**.**array**([**hue_lower**,** sat_lower**,** val_lower**])** upper_thres **=** np**.**array**([**hue_upper**,** sat_upper**,** val_upper**])**

mask **=** cv2**.**inRange**(**blur**,** lower_thres**,** upper_thres**)** res **=** cv2**.**bitwise_and**(**blur**,** blur**,** mask**=**mask**)**

# Show the image

tempImg2 **=** cv2**.**cvtColor**(**res**,** cv2**.**COLOR_HSV2RGB**)** tempImg2 **=** Image**.**fromarray**(**tempImg2**)** *self***.**showImg**(**tempImg2**)**

**def** thresholdDefault**(***self***):**

'''Returns the values to default'''

*self***.**HL**.**set**(***self***.**hue_lower**)** *self***.**HR**.**set**(***self***.**hue_upper**)** *self***.**SL**.**set**(***self***.**sat_lower**)** *self***.**SR**.**set**(***self***.**sat_upper**)** *self***.**VL**.**set**(***self***.**val_lower**)** *self***.**VR**.**set**(***self***.**val_upper**)**

**def** thresholdEnd**(***self***):** '''Applies the value'''

# Get values from slider

*self***.**hue_lower**,** *self***.**hue_upper **=** *self***.**HL**.**get**(),** *self***.**HR**.**get**()** *self***.**sat_lower**,** *self***.**sat_upper **=** *self***.**SL**.**get**(),** *self***.**SR**.**get**()** *self***.**val_lower**,** *self***.**val_upper **=** *self***.**VL**.**get**(),** *self***.**VR**.**get**()**

# Remove everything! *self***.**HL**.**grid_remove**()** *self***.**HR**.**grid_remove**()** *self***.**SL**.**grid_remove**()** *self***.**SR**.**grid_remove**()** *self***.**VL**.**grid_remove**()** *self***.**VR**.**grid_remove**()** *self***.**hueLabel**.**grid_remove**()** *self***.**satLabel**.**grid_remove**()** *self***.**valLabel**.**grid_remove**()** *self***.**check**.**grid_remove**()** *self***.**default**.**grid_remove**()** *self***.**confirm**.**grid_remove**()**

# Show original image

temp **=** cv2.cvtColor(*self***.**img, cv2.COLOR_BGR2RGB) temp **=** Image**.**fromarray**(**temp**)**

*self***.**showImg**(**temp**)**

# Analysis can begin

*self***.**thresholding **= False**

**def** exportFile**(***self***):**

'''Exports the results in a .csv file'''

# Get file path, name, and extension head**,** tail **=** path**.**split**(***self***.**filepath**)** name **=** tail**.**split**(**'.'**)[**0**]**

# Write the .csv file in the same directory as the image file **=** open**(**"{}/{}.csv"**.**format**(**head**,**name**),** 'w'**)**

# Maximum no. of parameters found in all groups

max_len **=** max**([**max**([**len**(**plant**) for** plant **in** *self***.**results**[**i**][**'val'**]])**\

**for** i **in** range**(**1**,** len**(***self***.**results**)+**1**)])**

# HEADINGS

# First column is total length

**print(**"Plant,Total"**,** end**=**''**,** file**=**file**)**

key **= [**'Internode'**,** 'Leaf'**] for** i **in** range**(**2**,** max_len**):**

# In between - either internode or leaf

**if** i **<** max_len**-**1**:**

**print(**",{} {}"**.**format**(**key**[**i**%**2**],** i**//**2**),** end**=**''**,** file**=**file**)**

# Final column is always leaf

**else:**

**print(**",Leaf {}"**.**format**((**i**)//**2**),** end**=**''**,** file**=**file**) print(**file**=**file**)**

i **=** 2 # To prevent errors with coleoptiles

**for** group_no **in** sorted**(***self***.**results**.**keys**()):** # Don't print group number for one group

**if** len**(***self***.**results**) !=** 1**:**

**print(**"Group {}"**.**format**(**group_no**),** file**=**file**)**

**for** plant **in** *self***.**results**[**group_no**][**'val'**]:**

# Initialize plant number and total length temp_str **=** plant**[**0**]**

temp_str **+=** ",{:.2f}"**.**format**(**plant**[**1**])**

**for** i **in** range**(**2**,** max_len**-**1**):**

# For plants with lesser branching points than maximum

**if** len**(**plant**) ==** i **and** i**%**2 **==** 1**:**

temp_str **+=** ",{:.2f}"**.**format**(**plant**[-**1**])**

# Plant doesn't have that parameter - diff for even and odd

**elif (**len**(**plant**) <=** i**+**1 **and** i**%**2 **==** 0**) or** \

**(**len**(**plant**) <** i **and** i**%**2 **==** 1**):**

temp_str **+=** ","

# Normal case

**else:**

temp_str **+=** ",{:.2f}"**.**format**(**plant**[**i**])**

# Last one - different conditions for even and odd

**if (**i**%**2 **==** 1 **and** len**(**plant**) >** i**+**1**) or (**i**%**2 **==** 0 **and** len**(**plant**) >=** i**+**1**):** temp_str **+=** ",{:.2f}"**.**format**(**plant**[-**1**])**

**print(**temp_str**,** file**=**file**)**

# Number of parameters in that group

group_len **= (**len**(***self***.**results**[**group_no**])-**1**)//**3

# For the statistics

key **= [**'internode'**,** 'leaf'**]**

**for** statistics **in [**'Mean'**,** 'SD'**,** 'SE'**]:**

# Initialize with heading and total length temp_str **=** statistics

temp_str **+=** ",{:.2f}"**.**format**(***self***.**results**[**group_no**]**\

**[**'length{}'**.**format**(**statistics**.**lower**())])**

# Same idea as before

**for** i **in** range**(**2**,** max_len**-**1**):**

# If the group as a whole has less params than the max param

**if (**group_len **<=** i **and** i**%**2**==**0**) or (**group_len**+**1 **<** i **and** i**%**2**==**1**):** temp_str **+=** ","

# Normal case

**else:**

temp_str **+=** ",{:.2f}"**.**format**(***self***.**results**[**group_no**]**\

**[**'{}{}{}'**.**format**(**key**[**i**%**2**],**i**//**2**,**statistics**.**lower**())])**

# Final value

**if (**group_len **==** i**+**1 **and** i**%**2**==**1**) or (**group_len **>=** i **and** i**%**2**==**0**):** temp_str **+=** ",{:.2f}"**.**format**(***self***.**results**[**group_no**][**'leaf{}{}'**.**\

format**((**i**+**1**)//**2**,** statistics**.**lower**())]) print(**temp_str**,** file**=**file**)**

*self***.**exported**.**set**(**"File has been exported as {}.csv"**.**format**(**name**))**

**def** exportImg**(***self***):**

'''Exports the labeled image'''

# Get file path, name, and extension head**,** tail **=** path**.**split**(***self***.**filepath**)** name**,** ext **=** tail**.**split**(**'.'**)**

# Write the labeled image in the same path as the original cv2**.**imwrite**(**"{}/{}_labeled.{}"**.**format**(**head**,** name**,** ext**),** *self***.**overlay**)**

*self***.**exported**.**set**(**"Image has been exported as {}_labeled.{}"**.**format**(**\

name**,** ext**))**

**def** reset**(***self***,** full**=True):** '''Resets after analysis'''

*self***.**disp**.**grid_remove**()** *self***.**result**.**grid_remove**()** *self***.**scroll**.**grid_remove**()** *self***.**e3**.**grid_remove**()** *self***.**merge_button**.**grid_remove**()** *self***.**select_button**.**grid_remove**()** *self***.**remove_button**.**grid_remove**()** *self***.**apply_button**.**grid_remove**()** *self***.**divider**.**grid_remove**()** *self***.**group_button**.**grid_remove**()** *self***.**exportfile_b**.**grid_remove**()** *self***.**exportimg_b**.**grid_remove**()** *self***.**reset_button**.**grid_remove**()** *self***.**exported**.**set**(**""**)** *self***.**analyzed **= False**

**if** full**:**

*self***.**imagename**.**set**(**""**)** *self***.**imagesize**.**set**(**""**)** *self***.**filepath **= None**

**def** calibrate**(***self***):**

'''Window for setting the scale bar position, scale, and detection limit'''

# New window

*self***.**t **=** Toplevel**()**

*self***.**t**.**bind**(**"<Return>"**,** *self***.**update**)** #Binds window to Enter key

*self***.**t**.**wm_title**(**"Settings"**)**

# Text

Label**(***self***.**t**,** text**=**"Plant type"**).**grid**(**row**=**0**,** column**=**0**,** padx**=**10**,**\ pady**=(**15**,**0**),** sticky**=**E**)**

Label**(***self***.**t**,** text**=**"Scale bar position"**).**grid**(**\

row**=**1**,** column**=**0**,** padx**=**10**,** pady**=(**5**,**0**),** sticky**=**E**)**

Label**(***self***.**t**,** text**=**"Known distance"**).**grid**(**\

row**=**2**,** column**=**0**,** padx**=**10**,** pady**=(**10**,**5**),** sticky**=**E**)**

Label**(***self***.**t**,** text**=**"mm"**).**grid**(**row**=**2**,** column**=**2**,** padx**=(**0**,**10**),**\ pady**=(**10**,**5**),** sticky**=**W**)**

Label**(***self***.**t**,** text**=**"Min detection area"**).**grid**(**\

row**=**3**,** column**=**0**,** padx**=**10**,** pady**=(**5**,**5**),** sticky**=**E**)**

Label**(***self***.**t**,** text**=**"pixels"**).**grid**(**row**=**3**,** column**=**2**,** \ padx**=(**0**,**10**),** pady**=(**5**,**5**),** sticky**=**W**)**

# Radiobuttons for type

Radiobutton**(***self***.**t**,** text**=**"Coleoptile"**,** variable**=***self***.**plant_label**,**\ value**=**"coleoptile"**).**grid**(**row**=**0**,** column**=**1**,** padx**=**5**,** pady**=(**15**,**0**),** sticky**=**W**)**

Radiobutton**(***self***.**t**,** text**=**"Seedling"**,** variable**=***self***.**plant_label**,**\ value**=**"seedling"**).**grid**(**row**=**0**,** column**=**1**,** padx**=(**90**,**0**),** pady**=(**15**,**0**),** sticky**=**W**)**

# Radiobuttons for scale bar position

Radiobutton**(***self***.**t**,** text**=**"Left"**,**variable**=***self***.**side_label**,**value**=**"left"**).**grid**(**\ row**=**1**,** column**=**1**,** padx**=**5**,** pady**=(**5**,**0**),** sticky**=**W**)**

Radiobutton**(***self***.**t**,** text**=**"Right"**,**variable**=***self***.**side_label**,**value**=**"right"**).**grid**(**\ row**=**1**,** column**=**1**,** padx**=(**90**,**0**),** pady**=(**5**,**0**),** sticky**=**W**)**

# Entry box: scale

*self***.**e1 **=** Entry**(***self***.**t**)**

*self***.**e1**.**insert**(**0**,** *self***.**c_entry**)**

*self***.**e1**.**grid**(**row**=**2**,** column**=**1**,** padx**=**10**,** pady**=(**10**,**5**),** sticky**=**W**+**E**)**

# Entry box: min area

*self***.**e2 **=** Entry**(***self***.**t**)**

*self***.**e2**.**insert**(**0**,** *self***.**min_area**)**

*self***.**e2**.**grid**(**row**=**3**,** column**=**1**,** padx**=**10**,** pady**=(**5**,**5**),** sticky**=**W**+**E**)**

# Buttons

Button**(***self***.**t**,** text**=**'Set'**,** command**=***self***.**update**).**grid**(**\ row**=**4**,** column**=**0**,** padx**=**10**,** pady**=**10**,** sticky**=**E**)**

Button**(***self***.**t**,** text**=**'Cancel'**,** command**=***self***.**t**.**destroy**).**grid**(**\ row**=**4**,** column**=**1**,** padx**=**10**,** pady**=**10**,** sticky**=**W**)**

**def** update**(***self***,** event**=None):**

'''Updates the settings and closes the window'''

*self***.**c_entry **=** int**(***self***.**e1**.**get**())** *self***.**min_area**=** int**(***self***.**e2**.**get**())** *self***.**side **=** *self***.**side_label**.**get**()** *self***.**plant **=** *self***.**plant_label**.**get**()** *self***.**t**.**destroy**()**

**def** guideline**(***self***):**

'''Text on how to use the program'''

*self***.**g **=** Toplevel**()**

*self***.**g**.**wm_title**(**"Guidelines"**)**

# When clicked, shows a different text

**def** Click**(**event**,** text**):** *self***.**G**.**config**(**state**=**'normal'**)** *self***.**G**.**delete**(**1.0**,** END**)** *self***.**G**.**insert**(**END**,** text**)** *self***.**G**.**config**(**state**=**'disabled'**)**

# Texts

*self***.**G **=** Text**(***self***.**g**,** height**=**33**,** width**=**75**)**

gentext **=** "This program works by converting a plant to a graph, with branching points and end points as nodes. It works on both rice coleoptiles and seedlings.

Anything shorter than 2mm will be considered as noise and removed.\n\nSteps:\n1. Upload a scanned image of coleoptiles or seedlings on a plate. For optimum performance, the following conditions are recommended:\n• A black background for coleoptiles or a blue background for seedlings\n• The plate contains no scratches and smudges; ideally it would also be gridless\n• No overlapping plants\n• The scale bar is a white strip of known length placed on either the lower right or left corner of the plate\n• No plant is in the same grid box as the scale bar (~3cm for gridless plates)\n\n2. Crop the plate borders and surrounding areas.\n\n3. For seedlings, check if your plants are being detected using the Threshold button. Adjust values accordingly.\n\n4. Check the settings to see that the plant type being analyzed and scale bar position and length are correct.\n\n5. Run the program. Each detected region will be labeled with a red bounding box and a number. In case of seedlings, yellow lines are drawn over the branch determined to be a leaf. This could be incorrect if the leaf is too close to the main stem.\n\n6. Merge, remove, select, or group regions as necessary. The labeled image can be exported to see the numberings more clearly. The mean, standard deviation, and standard error will be recalculated for each parameter.\n\n7. Export to a csv file.\n\n"

croptext **=** "CROPPING\nUse the four sliders to crop the four sides as needed.

The 'Check' button will give an overview of your end result. Try to crop as much of the surroundings as possible. There is no undo button once you confirm the cropping, so if there was a mistake just upload the image again.\n\n"THRESHOLDING\nThis option is only available for seedling analysis, since the threshold is based on color. (The threshold used in coleoptile analysis is black and white). There are three parameters - hue, saturation, and value - and each has two sliders for their lower and upper limit.\n\nHue is the color. The default value of 0 to 95 encompasses red, orange and green.\n\nSaturation is the intensity of color. Zero means no color (i.e., gray or black) and 255 is intense color. The default value was set to 0.\n\nValue is the brightness of the color. Like saturation, zero is also black. The default value was also set to 0.\n\nThe 'Check' button will show the detected regions in color.

Undetected regions are colored black. If the plants are not detected, you can try the following: (1) increase the Hue upper limit, (2) decreasing the Saturation lower limit, or (3) decreasing the Value lower limit. Be careful with (1) because at values above

100 blue will be detected as well.\n\nIf a black background was used and some noise was detected, try increasing the lower limits of saturation and value."

srmtext **=** "SELECTING AND REMOVING\nType the number(s) of plant(s) you want to select or remove in the entry box. Separate the numbers with a comma (no space in between). A range of numbers will also be accepted. For example, to remove plants number 1, 4, 5, 6, and 10, type '1,4-6,10' and click 'Remove.' Selection and removing can be done after grouping, and the statistics will be recalculated accordingly.\n\nMERGING\nType the numbers of two or more plants to be merged in the entry box, e.g., '4+17' will merge plants 4 and 17. Separate each merging with a comma (no space in between). Merging can only happen once, so first typing '4+17' then

'4+17+23' will not work - to merge three plants you have to include all the numbers at once. Only plants from the same group can be merged.\n\nMerging leaf values is a little more complicated. You will have to use the letter 'l' to indicate the leaf, followed by the leaf number. (Leaves are numbered from bottom to top.) For instance, if the second leaf of plant 4 was cut off and the stray leaf was numbered as 8, you have to type '4l2+8' to merge them. Always make sure that the main plant is the first value (i.e., before the plus sign).\n\n"GROUPING\nWhile in grouping mode, type asterisks (*) in a new line to separate the plants into groups. You can shift the rows around (Ctrl+X then Ctrl+V) and even remove them during grouping. Click 'Apply' to create the groups, and the statistics will be calculated for each group. If you wish to ungroup them, go into the grouping mode, remove the group heading(s), then apply."

*self***.**G**.**insert**(**END**,** gentext**)** # Styling left column

# Make the frame and text

labels **=** Frame**(***self***.**g**,** width**=**20**,** height**=**33**)** general **=** Label**(**labels**,** text**=**"General"**)**

cropthres **=** Label**(**labels**,** text**=**"Crop, Threshold"**)**

srm **=** Label**(**labels**,** text**=**"Select, Remove,\nMerge, Group"**,** justify**=**RIGHT**)**

# Place the frame, and underline the text

labels**.**grid**(**row**=**0**,** column**=**0**,** padx**=(**20**,**10**),** pady**=**20**,** sticky**=**N**)** f **=** font**.**Font**(**general**,** general**.**cget**(**"font"**))** f**.**configure**(**underline**=True)**

# Place the other labels general**.**grid**(**sticky**=**E**,** pady**=(**0**,**10**))** general**.**configure**(**font**=**f**)**

general**.**bind**(**"<Button-1>"**, lambda** event**,** arg**=**gentext**:**Click**(**event**,** arg**))**

cropthres**.**grid**(**sticky**=**E**,** pady**=**10**)** cropthres**.**configure**(**font**=**f**)**

cropthres**.**bind**(**"<Button-1>"**, lambda** event**,** arg**=**croptext**:** Click**(**event**,** arg**))**

srm**.**grid**(**sticky**=**E**,** pady**=**10**)** srm**.**configure**(**font**=**f**)**

srm**.**bind**(**"<Button-1>"**, lambda** event**,** arg**=**srmtext**:** Click**(**event**,** arg**))**

# Textbox

*self***.**G**.**grid**(**row**=**0**,** column**=**1**,** padx**=(**10**,**20**),** pady**=**20**)**

*self***.**G**.**config**(**state**=**"disabled"**,** background**=**"#f0f0f0"**,** borderwidth**=**0**,**\ font**=**"TkDefaultFont"**,** wrap**=**"word"**)**

# Close Button

Button**(***self***.**g**,** text**=**"Close"**,** command**=***self***.**g**.**destroy**).**grid**(**\ row**=**1**,** column**=**1**,** sticky**=**E**,** padx**=**20**,** pady**=(**0**,**20**))**

**def** about**(***self***):** '''Acknowledgements and stuff'''

*self***.**a **=** Toplevel**()**

*self***.**a**.**wm_title**(**"About"**)**

#Text

A **=** Text**(***self***.**a**,** height**=**15**,** width**=**57**)** A**.**insert**(**END**,** "PLENGTH 1.1\n"**)**

A**.**insert**(**END**,**"A program for measuring plant length\n\n"**)** A**.**insert**(**END**,**"Lab of Plant Growth Analysis, "**)** A**.**insert**(**END**,**"Ghent University Global Campus\n\n"**)** A**.**insert**(**END**,**"Created by Chananchida Sang-aram (Sai)\n"**)**

A**.**insert**(**END**,**"Special thanks to Prof. Stephen Depuydt, Prof. Wesley De Neve, "**)** A**.**insert**(**END**,**"Prof. Arnout Van Messem, Breght Vandenberghe, Jonas de Saeger, "**)**

A**.**insert**(**END**,** "Lena Vlaminck, Fanny, and Ashley!\n\n"**)** A**.**insert**(**END**,**"For any questions, feature requests, or bug reports "**)** A**.**insert**(**END**,**"please contact chananchida.sangaram@ghent.ac.kr.\n\n"**)** A**.**insert**(**END**,**"©2017, Python 3.5.2"**)**

#Configure stuff A**.**tag_add**(**"title"**,** "1.0"**,** "1.11"**)**

A**.**tag_config**(**"title"**,** font**=(**"TkDefaultFont"**,** 11**,** "bold"**))**

A**.**config**(**state**=**"disabled"**,** background**=**"#f0f0f0"**,** borderwidth**=**0**,**\ font**=**"TkDefaultFont"**,** wrap**=**"word"**)**

A**.**grid**(**padx**=**20**,** pady**=(**20**,**0**))**

#Button

Button**(***self***.**a**,** text**=**"Close"**,** command**=***self***.**a**.**destroy**).**grid**(**\ row**=**1**,** sticky**=**E**,** padx**=**20**,** pady**=(**0**,**20**))**

# Creation of root window root **=** Tk**()**

root**.**geometry**(**"1000x655"**)** # widthxheight

# creation of instance app **=** Window**(**root**)**

root**.**mainloop**()**
