# Supplementary material for: Development of a novel and rapid phenotype-based screening method to assess rice seedling growth
Source: Plant Methods. 2020 Oct 15;16:139. doi: 10.1186/s13007-020-00682-6 (PMC7560306; doi:10.1186/s13007-020-00682-6)
Supplement: Supplementary file 16 — Additional file 16. Plength installation manual. [file 13007_2020_682_MOESM16_ESM.pdf]

Plength Installation Guide

1. Install Python through Anaconda

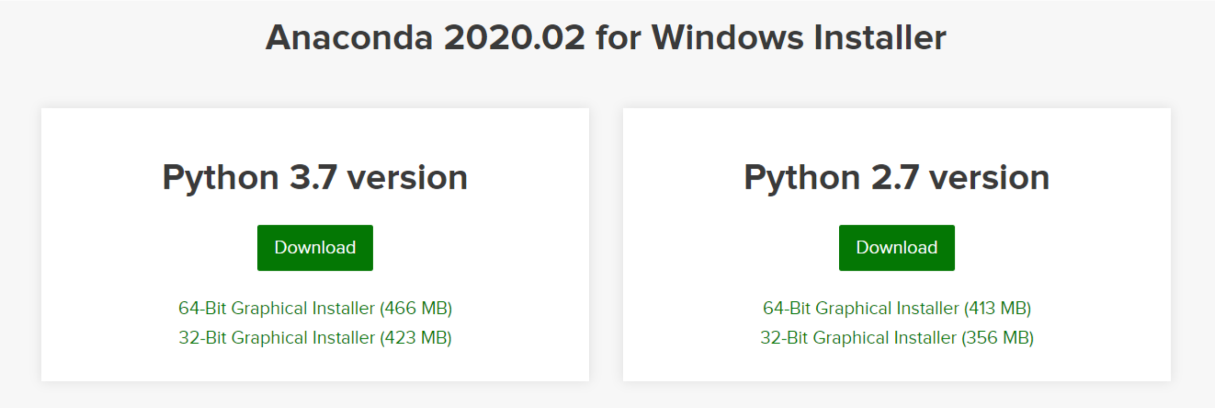

Download the Python 3.7 version. If you click the "Download" button it should automatically detect whether your computer is 32- or 64-Bit. It is recommended that you use Anaconda as your default Python and NOT add Anaconda to your PATH environment variable (see below).

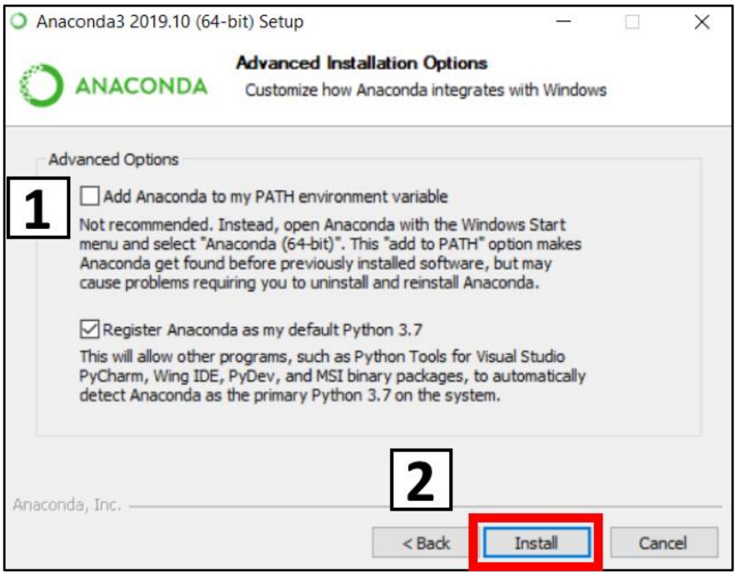

2. Install git and clone the repository

- Open "Anaconda prompt" (Using the "Command prompt" will not work!)

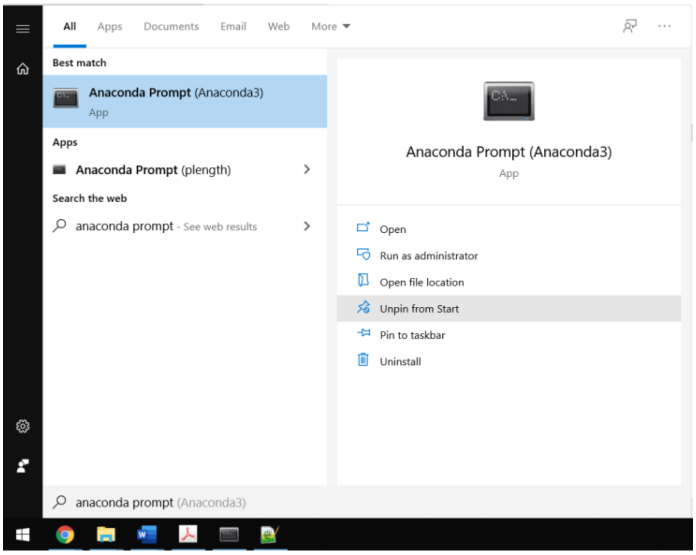

- Install git

```
conda install -c anaconda git
```

- When asked if you want to proceed, type "y" and press enter. Then type:

```
git clone https://github.com/csangara/plength.git
```

This will install the plength repository locally on your desktop.

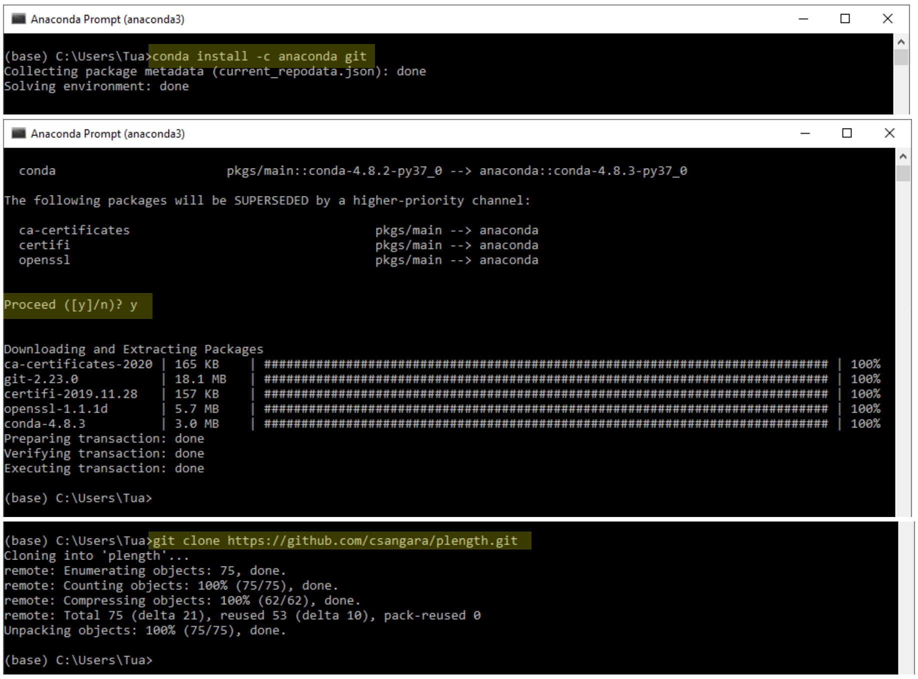

3. Create a virtual environment

- Create a virtual environment using the following command

```
conda create -n plength python=3.5
```

You will be asked if you want to install the following packages. Type “y” and press enter.

After a few moments, your virtual environment will be created, which will allow you to use specific versions of the libraries that were used to write Plength.

```
Anaconda Prompt (anaconda3)

(base) C:\Users\Tua>conda create -n plength python=3.5
Collecting package metadata (current_repodata.json): done
Solving environment: failed with repodata from current_repodata.json, will retry with next repodata source.
Collecting package metadata (repodata.json): done
Solving environment: done
```

```
Anaconda Prompt (anaconda3)

Proceed ([y]/n)? y

Downloading and Extracting Packages
certifi-2018.8.24    | 137 KB    | ############################################################################ | 100%
setuptools-40.2.0    | 497 KB    | ############################################################################ | 100%
python-3.5.6         | 14.5 MB   | ############################################################################ | 100%
wincertstore-0.2     | 14 KB     | ############################################################################ | 100%
wheel-0.31.1         | 82 KB     | ############################################################################ | 100%
pip-10.0.1           | 1.6 MB    | ############################################################################ | 100%
Preparing transaction: done
Verifying transaction: done
Executing transaction: done
#
# To activate this environment, use
#
#     $ conda activate plength
#
# To deactivate an active environment, use
#
#     $ conda deactivate

(base) C:\Users\Tua>
```

4. Activate the virtual environment

- In the Anaconda prompt, type the following command:

```
conda activate plength
```

It is very important to do this every time before running the Plength script. You will be able to tell that you are in a virtual environment based on the start of the line, which changes from `(base)` to `(plength)`

```
(base) C:\Users\Sai>conda activate plength

(plength) C:\Users\Sai>
```

5. Install the required libraries

First you will have to enter the plength folder with the `cd` command, then install a specific package with `conda`, then install the rest of the packages with `pip`.

- `cd plength`
- `conda install llvmlite=0.23.1` (again, type 'y' when prompted)
- `pip install .`

(Note that the "." is very important!)

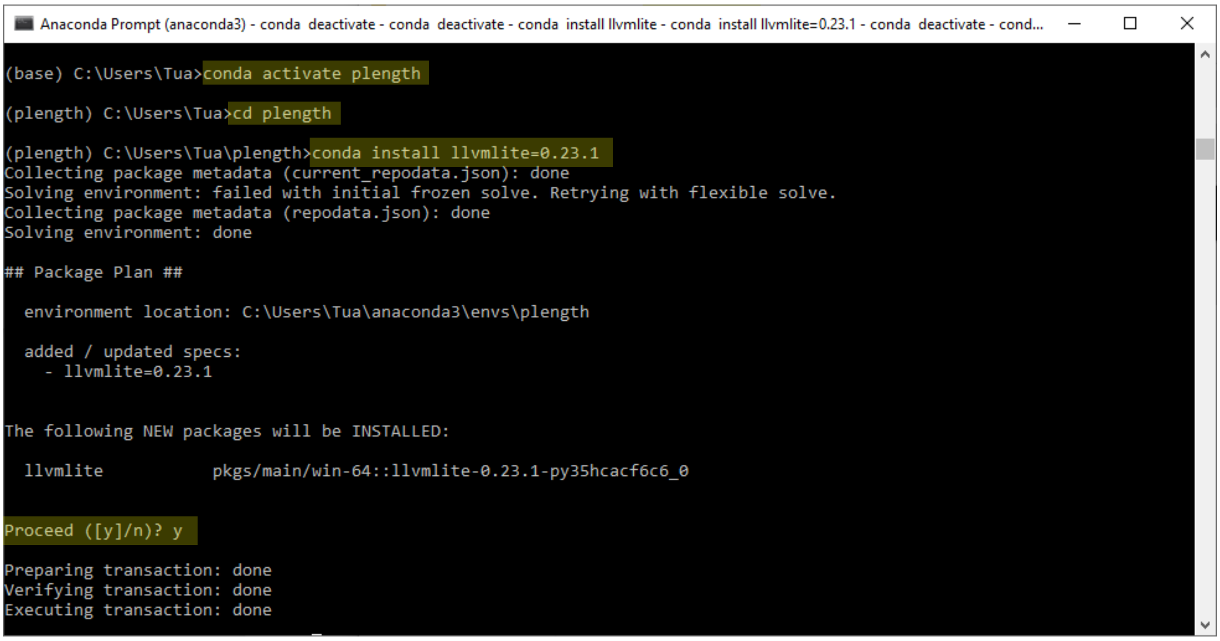

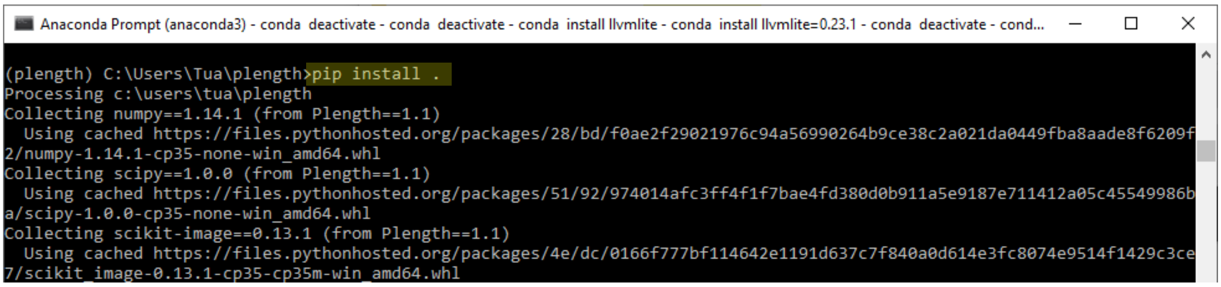

After all packages have been installed you should be able to run Plength with the command

`python plength.py`

(PLENGTH does not have to be capitalized in Windows computers.)

5. Using Plength afterwards

From now on, every time you would like to use Plength, you must

- Open “Anaconda Prompt”
- Type `conda activate plength`
- Type `cd plength`
- Type `python plength.py`

Alternatively, if you move the PLENGTH.py file to your home directory, you can skip the `cd plength` step.

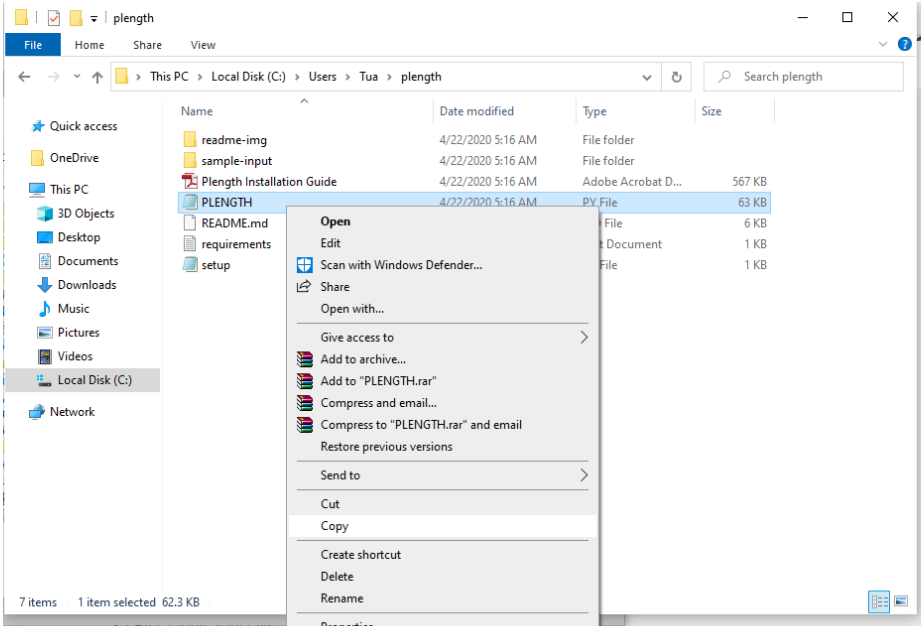

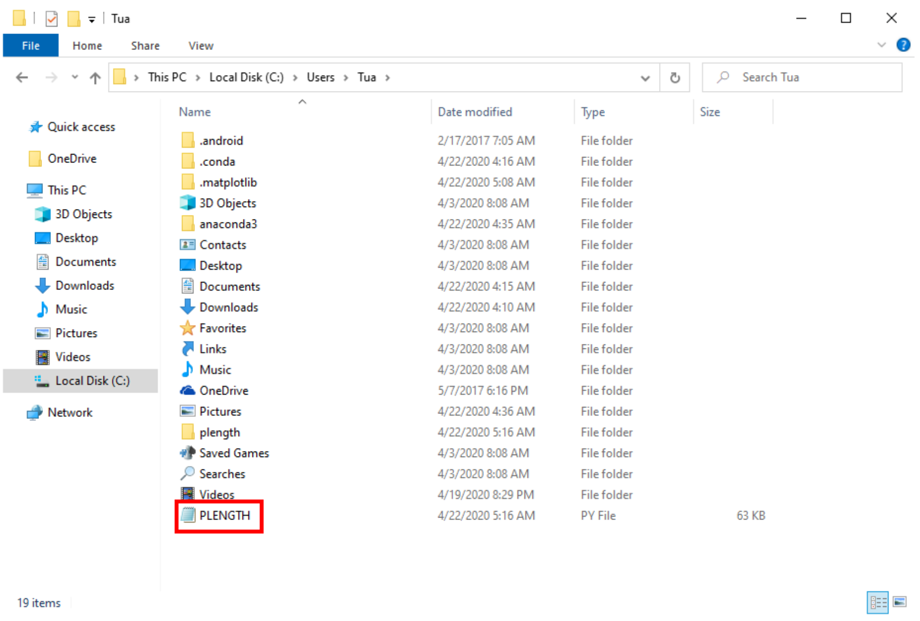

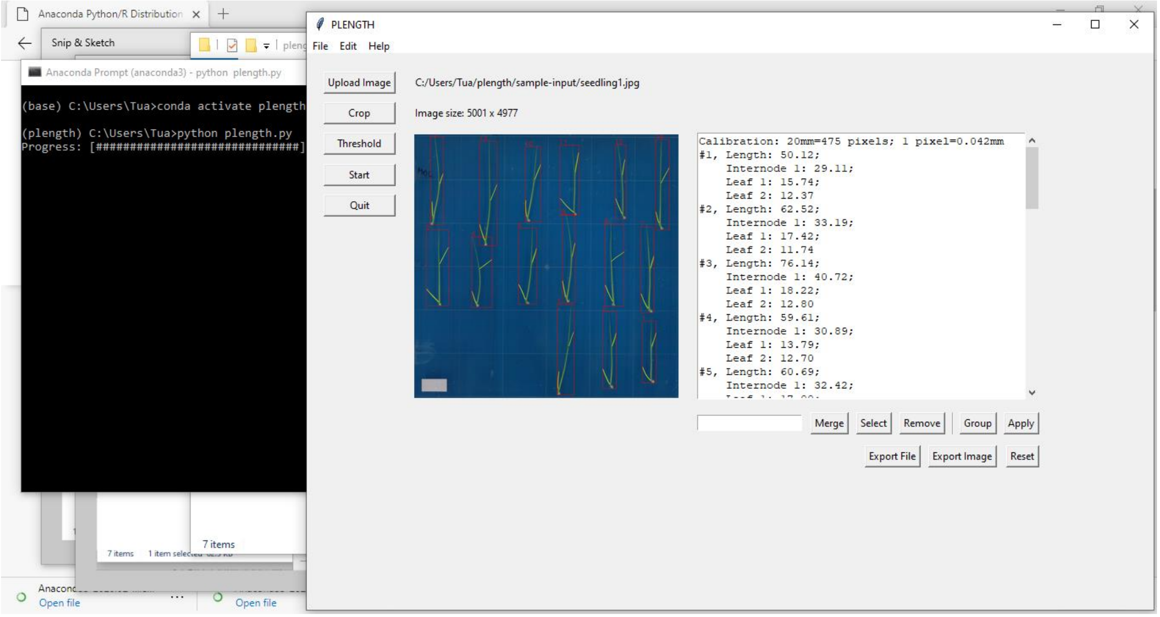

Happy measuring!
